# Cu-catalyzed oxygenation of alkene-tethered amides with O_2_*via* unactivated C=C bond cleavage: a direct approach to cyclic imides†
†Electronic supplementary information (ESI) available. CCDC 1912652. For ESI and crystallographic data in CIF or other electronic format see DOI: 10.1039/c9sc03175h


**DOI:** 10.1039/c9sc03175h

**Published:** 2019-08-06

**Authors:** Junhua Li, Jialiang Wei, Bencong Zhu, Teng Wang, Ning Jiao

**Affiliations:** a State Key Laboratory of Natural and Biomimetic Drugs , School of Pharmaceutical Sciences , Peking University , Xue Yuan Road 38 , Beijing 100191 , China . Email: jiaoning@pku.edu.cn; b School of Chemistry , Beihang University , Xue Yuan Road 37 , Beijing , 100191 , China; c State Key Laboratory of Organometallic Chemistry , Chinese Academy of Sciences , Shanghai 200032 , China

## Abstract

The transformations of unactivated alkenes through C=C bond double cleavage are always attractive but very challenging. We report herein a chemoselective approach to valuable cyclic imides by a novel Cu-catalyzed geminal amino-oxygenation of unactivated C=C bonds. O_2_ was successfully employed as the oxidant as well as the O-source and was incorporated into alkenyl amides *via* C=C bond cleavage for the efficient preparation of succinimide or glutarimide derivatives. Moreover, the present strategy under simple conditions can be used in the late-stage modification of biologically active compounds and the synthesis of pharmaceuticals, which demonstrated the potential application.

## Abstract 2

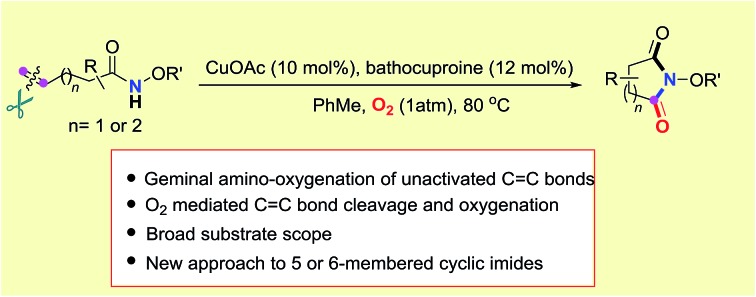
An efficient aerobic unactivated C=C bond cleavage process was achieved, in which the succinimide or glutarimide derivatives could be prepared directly from alkenyl amides.

5- and 6-membered cyclic imide moieties are frequently present as the key subunit in many pharmaceuticals and bioactive compounds^1^ (Fig. 1). For instance, 5-membered cyclic imides (succinimides) **i**, **ii** and **iii** are commonly used to treat petit mal epilepsy,^2^ while 6-membered cyclic imides (glutarimides) **iv**, **v**, and **vi** could be used as sedative-hypnotics^3^ and antineoplastic and immunomodulatory drugs.^4^ Interestingly, the penitential thalidomide **vi** returned to the market for the treatment of cancer under the brand name Immunoprin,^5^ since the crisis of thalidomide in the 1960s.^6^ The evolution of synthetic methods for cyclic imides is continuously driven forward by their importance in medicinal chemistry. Typical methods for the synthesis of simple imides include the ammonolysis of anhydrides at high temperature,^7^ the oxidation of lactams with strong or special oxidants,^8^ the reduction of unsaturated imides such as maleimide,^9^ and metal-catalyzed carbonylation of various precursors.^10^ However, the approach to polysubstituted cyclic imides remains challenging due to tedious transformations that are required for the preparation of anhydride precursors.

**Fig. 1:**
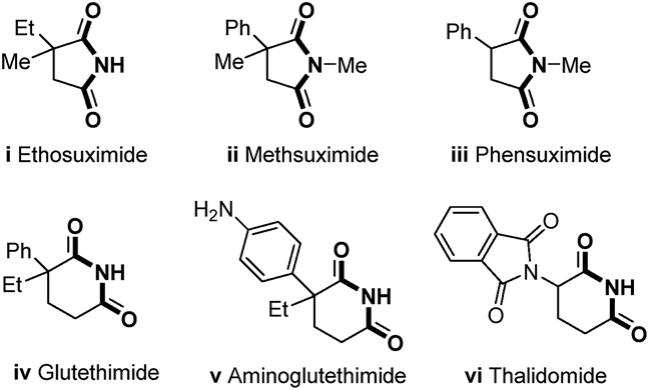
Some pharmaceuticals with succinimide and glutarimide moieties.

It is instructive that the difunctionalization of alkenes is such a versatile transformation for the assembly of two individual functional groups across C=C double bonds.^11^ Alkene-tethered amides, which serve as the substrates for intramolecular amidation of unactivated alkenes initiated by the photo-induced amidyl radical formation or metal-mediated amidocyclization, have emerged as the ideal precursor of γ-lactams in recent years with well-established transformations^12^ (Scheme 1(a1)).

**Scheme 1:**
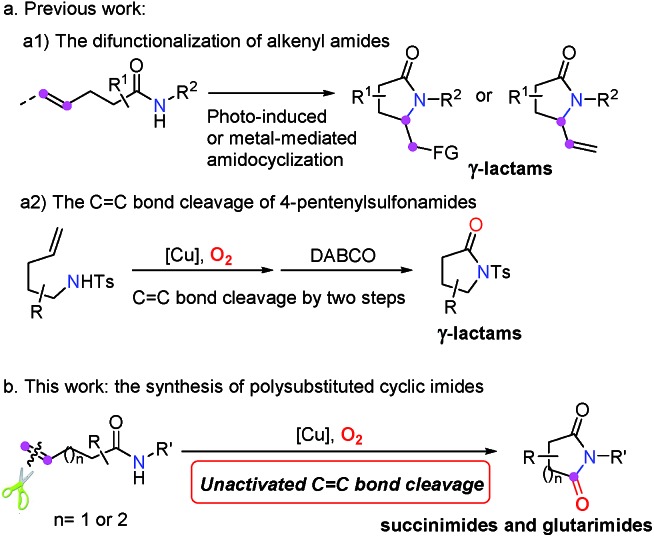
The strategies for the oxygenation of unactivated alkenyl amides.

Recently, O_2_ or air has been regarded as an ideal oxidant because of its inexpensive, environmentally benign and high atom economy characteristics, which attract academic and industrial attention.^13^ The oxygenations of olefins enable efficient protocols for the construction of epoxides,^14^ diols,^15^ and ketones^16^ with/without C=C double bond cleavage.^17^ Given what we discovered in our previous studies on the oxygen mediated C=C bond cleavage,^18^ we proposed that the geminal olefin amino-oxygenation of pent-4-enamides *via* chemoselective C=C double bond cleavage would be highly promising to produce succinimides in the presence of oxygen (Scheme 1b). To date, the aerobic oxidation of enamides has only been reported in the Pd(ii)-catalyzed intramolecular aza-Wacker-type cyclization.^19^ Recently, significant aminooxygenation of 4-pentenylsulfonamides was reported by Chemler and coworkers (Scheme 1(a 2)),^20^ in which the C=C bond cleavage was successfully achieved but in two steps. DABCO was required as a base with the formation of γ-lactam products. To the best of our knowledge, the chemoselective cleavage of C=C double bonds in alkene-tethered amides for cyclic imide synthesis has not been accomplished yet.

Our investigation commenced with *N*-methoxy alkenyl amide **1a**. After a lot of experiments, we were surprised to find that the unactivated C=C double bond could be cleaved with the incorporation of one oxygen atom using O_2_. Encouraged by the copper catalyzed olefin amino-oxygenation which delivered **2a** in 47% yield (Table 1, entry 1), a variety of conditions were screened (see the ESI†). The control experiments demonstrated that the reaction could not work in the absence of the copper catalyst, or oxygen atmosphere (Table 1, entries 2 and 3). Besides, different copper catalysts, solvents, additives and ligands were also screened (Table 1, entries 5–10). The yields sharply decreased when bases or acids were used as additives. Finally, we found that with copper acetate as the catalyst and bathocuproine (**Ligand II**) as the ligand the unactivated C=C double bond geminal amino-oxygenation reaction in toluene proceeded well and produced the desired succinimide product **2a** with excellent efficiency (83% isolated yield, Table 1, entry 10).

**Table 1:** Screening of reaction conditions^*a*^

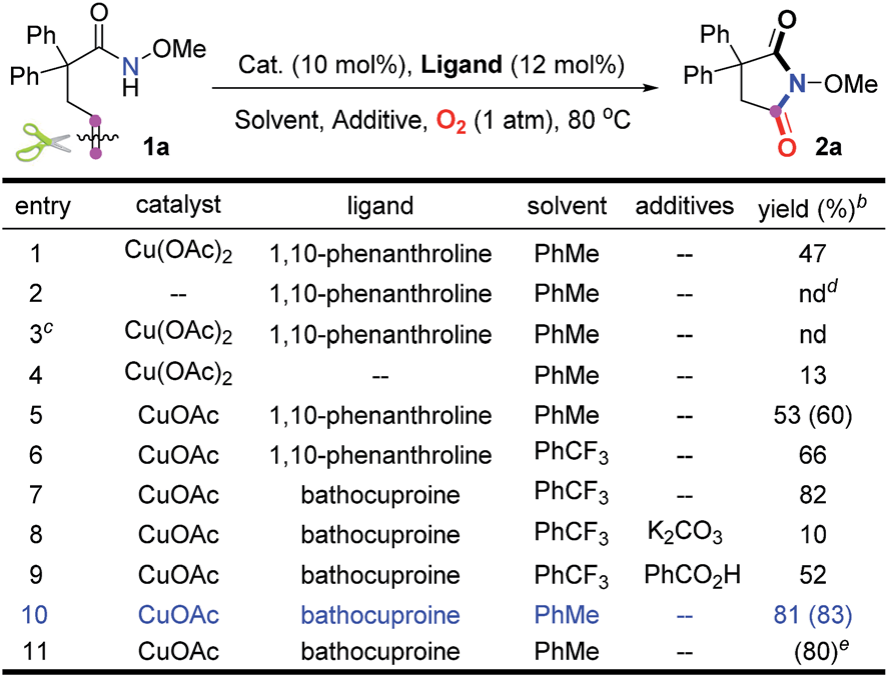

^*a*^Reaction conditions: **1a** (0.2 mmol), catalyst (0.02 mmol), ligand (0.024 mmol) and additive (1.0 equiv.) were stirred in solvent (2.0 mL) at 80 °C under O_2_.

^*b*^Determined by ^1^H NMR analysis using 1,1,2,2-tetrachloroethane as the internal standard. The numbers in parentheses are isolated yields.

^*c*^Under an argon atmosphere.

^*d*^Not detected.

^*e*^This reaction was carried out under air.

Subsequently, a good number of pent-4-enamides were smoothly converted to succinimides in moderate to good yields (Table 2). Several *N*-alkoxy protecting groups were well tolerated (up to 84%, **2a–2c**) while substrates bearing bulky groups showed poor conversion (**2d** and **2e**). When a hydrogen atom (**2f**) or benzyl group (**2g**) was attached to the amide nitrogen, the reaction did not work. The reason is that the alkyl-metal intermediate formation might be favored with the assistance of alkoxy protecting groups.12d,e α-Geminal substituted substrates worked well in this transformation (Table 2, **2h–2o**), producing polysubstituted and spiro-succinimides in moderate to good yields. It is noteworthy that the reaction could contain one of the identical allyl groups specifically to give the allylic imide in 44% yield (**2o**). The mono-methyl or benzyl substituted enamides were also tolerated, and the desired products could be obtained in fair yields (**2p** and **2q**). To our delight, the vinylcyclohexane derived enamide underwent the process smoothly to afford the corresponding imide **2s**. 2-Vinylbenzamide was also compatible to give the synthetically important phthalimide **2t** albeit the efficiency is a little bit low because the conjugated alkenes would undergo unwanted oxidation. Unfortunately, the alkene-tethered amide without alkylation of the backbone did not work. Notably, the glutarimide derivatives **4a–4d** were also obtained in moderate yields with hex-5-enamides (Table 3).

**Table 2:** Substrate scope for the synthesis of succinimides^*a*^

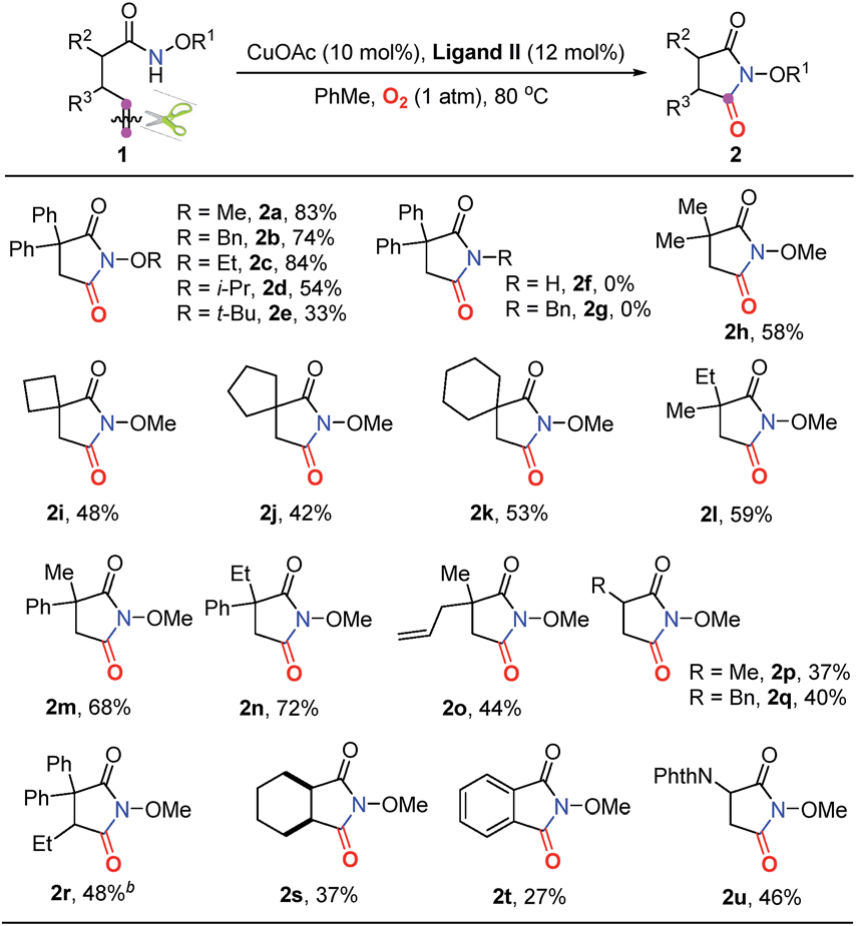

^*a*^Reaction conditions: see entry 10, Table 1. Isolated yields.

^*b*^Reaction for 48 hours.

**Table 3:** Substrate scope for the synthesis of glutarimides^*a*^

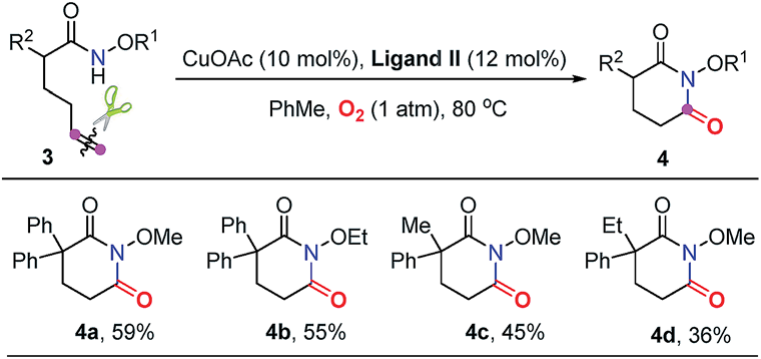

^*a*^Reaction conditions: see entry 10, Table 1. Isolated yields.

To demonstrate the synthetic value of our strategy, several late-stage modifications of biologically active compounds were carried out under standard conditions (Scheme 2).

**Scheme 2:**
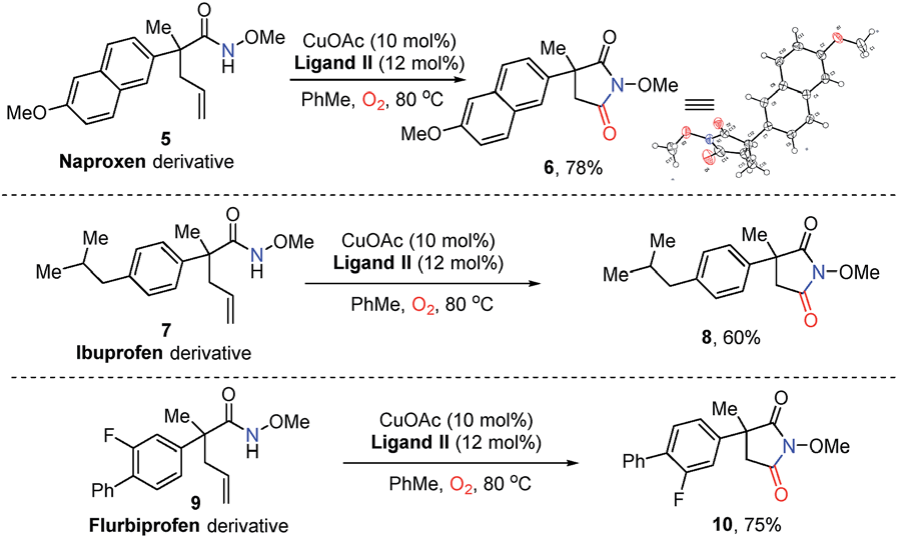
Application in the synthesis of drug analogues.

Naproxen, approved by the USA Food and Drug Administration (FDA) as an anti-inflammatory, antipyretic and analgesic agent, could deliver succinimide **6** in 78% yield *via* analogue **5** under standard conditions (Scheme 2). The structure of **6** was confirmed by X-ray single crystal structural analysis. Additionally, the derivatives of best-selling drugs ibuprofen and flurbiprofen could also undergo the present transformation to afford the desired product **8** in 60% yield and **10** in 75% yield respectively. These results provide efficient approaches to drug analogues for future medicinal chemistry studies.

Furthermore, our strategy can be applied to the synthesis of two pharmaceutical compounds ethosuximide **i** and methsuximide **ii** (Fig. 1). As shown in Scheme 3, the amidocyclization of **11** gave *N*-benzyloxy succinimide **12** in 69% yield under standard conditions, followed by the removal of the *N*-benzyloxy group by hydrogenation and treatment with 2-bromoacetophenone and triethylamine to furnish^21^ the ethosuximide in high yield (**13**, 83%), which possesses antiepileptic effects. This method avoids the use of highly toxic hydrocyanic acid in industrial production. Similarly, the methsuximide **17** could also be obtained from succinimide **15** in good overall yield.

**Scheme 3:**
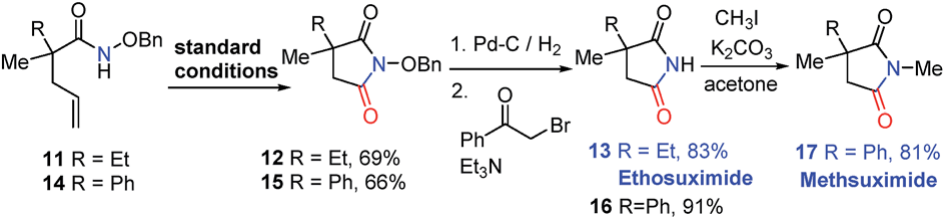
Synthesis of succinimide-containing medicines.

In order to probe the mechanism, some control experiments were designed and investigated (Scheme 4). Firstly, the reaction was conducted in the presence of TEMPO as a radical scavenger, and the difunctionalization product **18** could be obtained in 87% yield, with the formation of **2a** in only 10% yield (Scheme 4a). This result indicates that an alkyl radical intermediate was produced after the intramolecular amido-cyclization process. Then, to investigate the intermediacy of an aldehyde in the C=C bond cleavage, 2-pyrrolidinone **19** was employed under standard conditions. The formation of **2r** with some unconsumed raw materials compared with the results in Table 2 indicates that an aldehyde might be involved in this transformation (Scheme 4b). In addition, the isotopic labeling studies under ^18^O_2_ delivered the labeled succinimide [^18^O]-**2a** in 80% yield (67% ^18^O) due to the exchange with H_2_O (see the ESI†), which supports our expectation. We also studied the reaction kinetic profile, which showed the initial increase and later consumption of the aldehyde intermediate along with the formation of succinimide (Fig. 2). This result was in accordance with our aforementioned observation. Tentative studies on trapping intermediates were also carried out by EPR (see the ESI†).

**Scheme 4:**
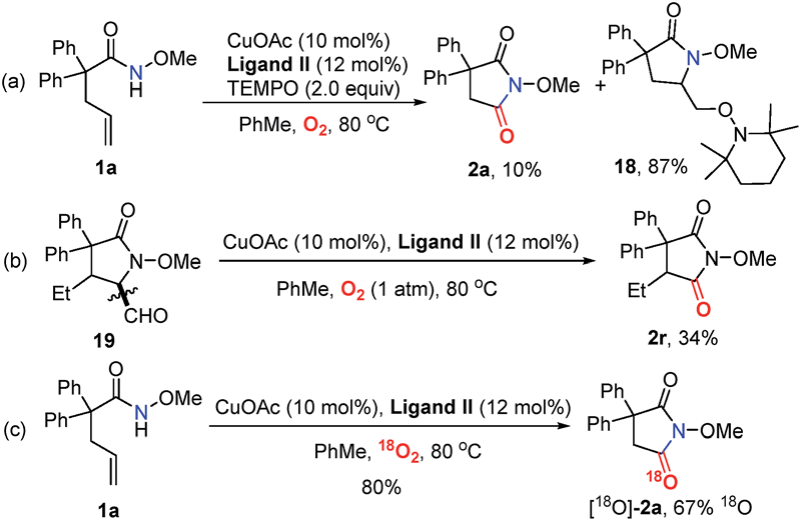
Mechanistic studies.

**Fig. 2:**
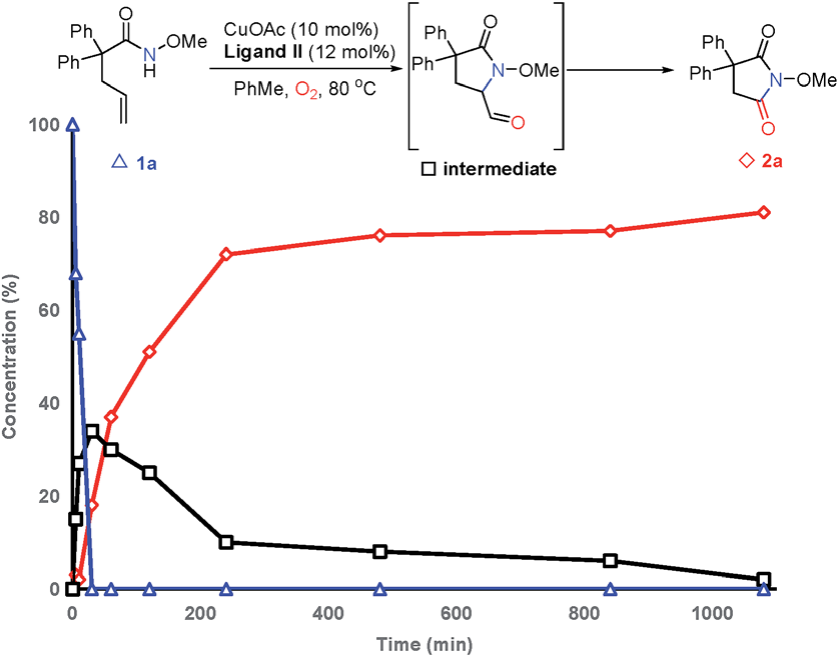
Kinetic profile of the C=C bond oxygenation reaction.

Based on previously reported12e,^20^ and our own mechanistic studies, a plausible mechanism is shown in Scheme 5. We proposed that copper(i) is oxidized to copper(ii) by O_2_ in the initial step. Then, copper(ii)-catalyzed alkene *cis*-amidocupration affords an unstable organocopper(ii) intermediate **B**. Primary radical **C**, which could be trapped by TEMPO (Scheme 4a), is subsequently generated by the C–Cu homolysis of the intermediate **B**.^22^ The mechanism is not completely clear yet. Alternatively, the lack of detection of the amidyl radical by EPR analysis (see the ESI†) could not fully disprove its presence under the reaction conditions. The primary radical **C** may also be generated by the addition of the amidyl radical to the double bond. Then, the radical species **C** is trapped by molecular oxygen and produces the superoxide radical **D**. Then, the intramolecular 1,3-hydrogen migration occurs to form the intermediate **E**, followed by the O–O homolysis to give the aldehyde **F** and hydroxyl radical which is unstable and easily reduced *in situ* to give the hydroxide anion. The intermediate aldehyde **F** could be directly transformed into copper(ii) enolate **G** which undergoes formal [2 + 2] cycloaddition with another molecule of oxygen to give the 1,2-dioxetane **J***via* radical species **H** and the cyclic peroxo intermediate **I**.^23^ Then the ring opening process occurs to form the succinimide **2a** and release carbon monoxide.

**Scheme 5:**
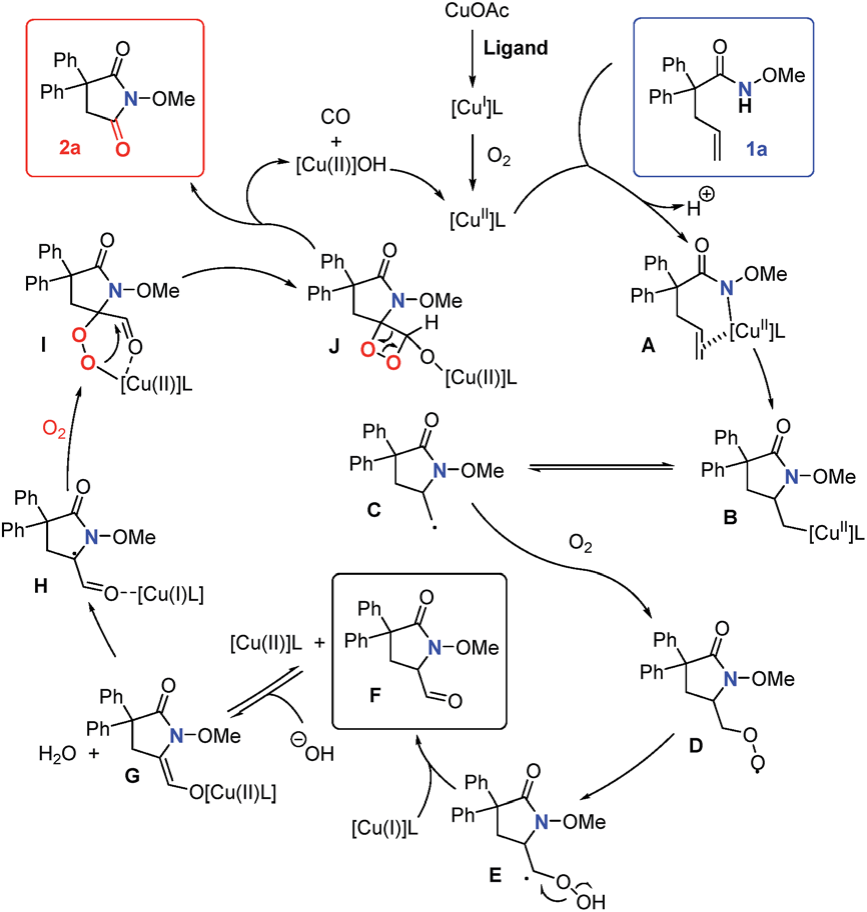
Proposed mechanism of the reaction.

In summary, we developed a novel molecular oxygen mediated geminal amino-oxygenation of unactivated olefins in alkene-tethered amides *via* a chemoselective cyclization/C=C bond cleavage processes that revealed an efficient approach to polysubstituted succinimides and glutarimides. Our reaction exhibited good functional group tolerance under simple conditions. The success of this protocol in the late-stage modification of biologically active compounds and the synthesis of pharmaceuticals would motivate further exploration of the transformations of unactivated alkenes.

## Conflicts of interest

The authors declare no competing financial interest.

## Supplementary Material

Supplementary information

Crystal structure data

## References

[cit1] Crider A. M., Kolczynski T. M., Yates K. M. (1980). J. Med. Chem..

[cit2] Sorel L. (1960). Acta Neurol. Psychiatr. Belg..

[cit3] Keberle H., Hoffmann K., Bernhard K. (1962). Experientia.

[cit4] Santen R. J., Santner S., Davis B., Veldhuis J., Samojlik E., Ruby E. (1978). J. Clin. Endocrinol. Metab..

[cit5] Kumar S. K., Rajkumar S. V., Dispenzieri A., Lacy M. Q., Hayman S. R., Buadi F. K., Zeldenrust S. R., Dingli D., Russell S. J., Lust J. A., Greipp P. R., Kyle R. A., Gertz M. A. (2008). Blood.

[cit6] Mcbride W. G. (1961). Lancet.

[cit7] Katoh T., Nishide K., Node M., Ogura H. (1999). Heterocycles.

[cit8] Zhang Y., Riemer D., Schilling W., Kollmann J., Das S. (2018). ACS Catal..

[cit9] Bayat M., Fox J. M. (2016). J. Heterocycl. Chem..

[cit10] Driller K. M., Klein H., Jackstell R., Beller M. (2009). Angew. Chem., Int. Ed..

[cit11] Yin G., Mu X., Liu G. (2016). Acc. Chem. Res..

[cit12] Nicolai S., Piemontesi C., Waser J. (2011). Angew. Chem., Int. Ed..

[cit13] Sigman M. S., Jensen D. R. (2006). Acc. Chem. Res..

[cit14] Hess J. S., Leelasubcharoen S., Rheingold A. L., Doren D. J., Theopold K. H. (2002). J. Am. Chem. Soc..

[cit15] Raja R., Sankar G., Thomas J. M. (1999). Chem. Commun..

[cit16] Drago R. S., Corden B. B., Barnes C. W. (1986). J. Am. Chem. Soc..

[cit17] Jun C.-H. (2004). Chem. Soc. Rev..

[cit18] Wang T., Jiao N. (2013). J. Am. Chem. Soc..

[cit19] Trend R. M., Ramtohul Y. K., Ferreira E. M., Stoltz B. M. (2003). Angew. Chem., Int. Ed..

[cit20] Wdowik T., Chemler S. R. (2017). J. Am. Chem. Soc..

[cit21] Silvers M. A., Robertson G. T., Taylor C. M., Waldrop G. L. (2014). J. Med. Chem..

[cit22] Jiao J.-W., Bi H.-Y., Zou P.-S., Wang Z.-X., Liang C., Mo D.-L. (2018). Adv. Synth. Catal..

[cit23] Cossy J., Belotti D., Bellosta V., Brocca D. (1994). Tetrahedron Lett..

