# Supplementary material for: Cu-catalyzed oxygenation of alkene-tethered amides with O2via unactivated C[double bond, length as m-dash]C bond cleavage: a direct approach to cyclic imides
Source: Chem Sci. 2019 Aug 6;10(39):9099–103. doi: 10.1039/c9sc03175h (PMC6889834; doi:10.1039/c9sc03175h)
Supplement: Supplementary file 1 [file SC-010-C9SC03175H-s001.pdf]

Electronic Supplementary Material (ESI) for Chemical Science.
This journal is © The Royal Society of Chemistry 2019

Supporting Information for:

Cu-catalyzed Oxygenation of Alkene-tethered Amides with O_2 via Unactivated C=C Bond Cleavage: A Direct Approach to Cyclic Imides

Junhua Li,[a] Jialiang Wei,[a] Bencong Zhu,[a] Teng Wang,[b] and Ning Jiao*[,ac]

[a] State Key Laboratory of Natural and Biomimetic Drugs, School of Pharmaceutical Sciences, Peking University, 100191 Beijing, China;

[b] School of Chemistry, Beihang University, 100191, Beijing, China;

[c] State Key Laboratory of Organometallic Chemistry, Chinese Academy of Sciences, 200032, Shanghai, China;

E-mail: jiaoning@bjmu.edu.cn;

Fax: (+86)10-82805297

Table of Contents

A. General information	S2
B. Optimization details for the unactivated C=C bond cleavage	S3-S9
C. The synthesis of substrates	S9-S24
D. The general experimental procedure and the characterization data of products	S24-S35
E. The crystal structure of compound 6	S35
F. Synthesis of succinimide-containing bioactive compounds	S35-S37
G. Mechanistic investigation	S37-S44
H. References	S44-S45
I. ^{1}H NMR and ^{13}C NMR spectra	S46-S172

A. General information

All commercially available compounds were purchased from Sigma-Aldrich, Alfa-Aesar, Acros, Beijing Ouhe and Beijing Chemical Works, Ltd. Unless otherwise noted, materials obtained from commercial suppliers were used without further purification. Analysis of crude reaction mixture was done on an Agilent 7890 GC System with an Agilent 5975 Mass Selective Detector. Products were purified by flash chromatography on silica gel. ^{1}H-NMR spectra were recorded on Bruker AVANCE III-400 spectrometers. Chemical shifts (in ppm) were referenced TMS (0.00 ppm). ^{13}C-NMR spectra were obtained by using the same NMR spectrometers and were calibrated with $CDCl_3$ (δ = 77.00 ppm). Mass spectra were recorded using a PE SCLEX QSTAR spectrometer. High resolution mass spectra were obtained with a Bruker APEX IV Fourier transform ion cyclotron resonance mass spectrometer.

B. Optimization details for the unactivated C=C bond cleavage.

Table S1. The control experiment of the reaction[a]

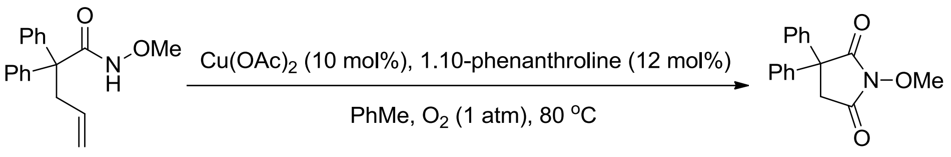

1a **2a**

Entry	Condition	Yield (%)[b]
1	Standard condition	47
2	Without $Cu(OAc)_2$	nd[c]
3	Without 1,10-phenanthroline	13
4	Without O_2	nd

[a] Unless noted otherwise, reaction conditions: **1a** (0.20 mmol), $Cu(OAc)_2$ (0.02 mmol), 1,10-phenanthroline (0.024 mmol), in toluene (2.0 mL) was stirred at 80 °C under O_2 atmosphere. [b] Determined by ^{1}H NMR analysis using 1,1,2,2-tetrachloroethane as internal standard. [c] Not determined.

Table S2. Optimization of copper catalysts[a]

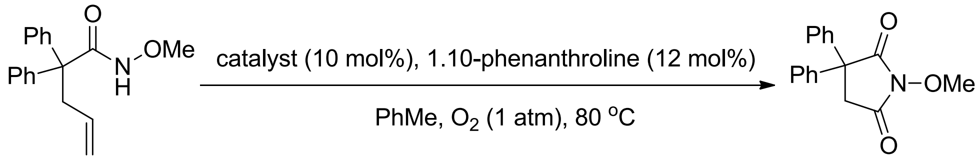

1a **2a**

Entry	[Cu] catalyst	Yield (%)[b]
1	$Cu(OAc)_2$	47
2	CuOAc	53 (60)[c]
3	CuBr	34
4	CuBr2	16
5	CuCl	44
6	$Cu(OTf)_2$	19
7	$Cu(CH_3CN)_4PF_6$	14
8	$CuCl_2$	19
9	$Cu(acac)_2$	54 (59)[c]
10	CuI	44
11	CuCN	51

[a] Unless noted otherwise, reaction conditions: **1a** (0.20 mmol), copper catalyst (0.02 mmol), 1,10-phenanthroline (0.024 mmol), in toluene (2.0 mL) was stirred at 80 °C under O_2. [b] Determined by ^{1}H NMR analysis using 1,1,2,2-tetrachloroethane as internal standard. [c] Isolated yield.

Table S3. Optimization of solvents[a]

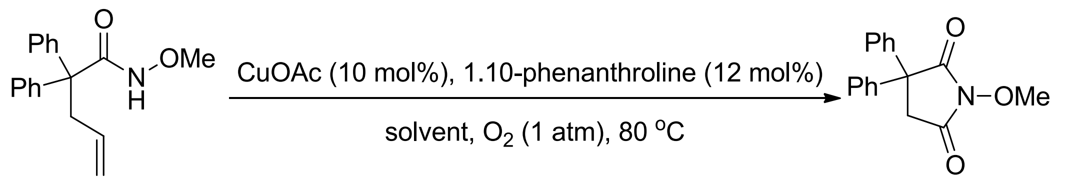

Entry	Solvent	Yield (%)[b]
1	PhMe	53
2	CH_3CN	48
3	CH_3OH	22
4	PhCl	63
5	1.2-DCE	50
6	1.4-dioxane	26
7	acetone	nd
8	ethyl acetate	50
9	DMF	nd
10	DMSO	nd
11	THF	nd
12	CH_3NO_2	9
13	cyclohexane	3
14	1,2-dimethoxyethane	16
15	$PhCF_3$	66

[a] Unless noted otherwise, reaction conditions: **1a** (0.20 mmol), CuOAc (0.02 mmol), 1,10-phenanthroline (0.024 mmol), in solvent (2.0 mL) was stirred at 80°C under O_2. [b] Determined by ^{1}H NMR analysis using 1,1,2,2-tetrachloroethane as internal standard.

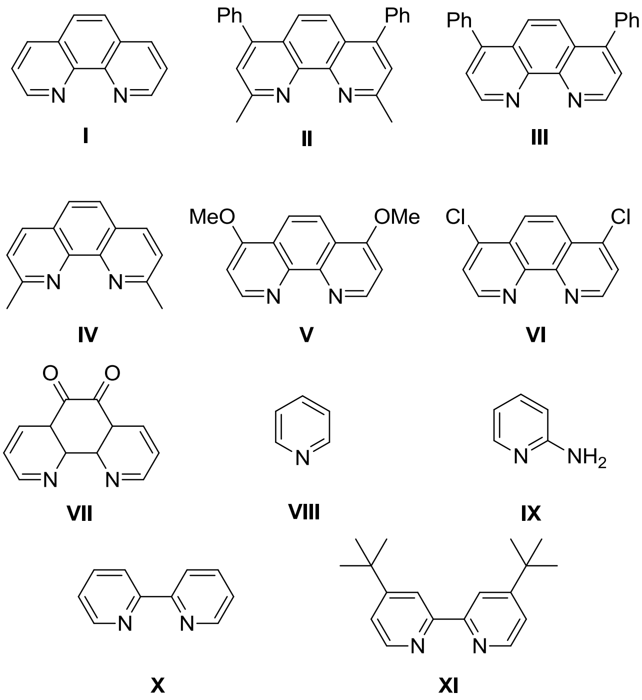

Fig. S1 The structures of screened ligand.

Table S4. Optimization of ligands[a]

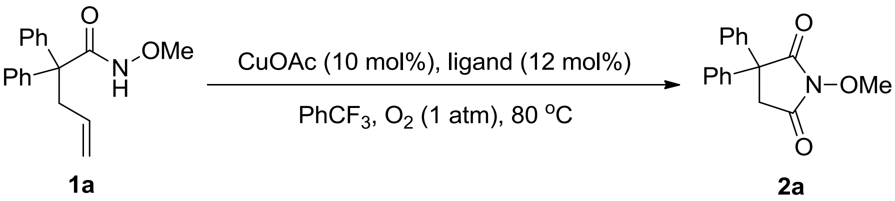

Entry	Ligand	Yield (%)[b]
1	**I**	66
2	**II**	82
3	**III**	69
4	**IV**	76
5	**V**	67
6	**VI**	33
7	**VII**	32
8[c]	**VIII**	29
9	**IX**	32
10	**X**	61
11	**XI**	66

[a] Unless noted otherwise, reaction conditions: **1a** (0.20 mmol), CuOAc (0.02 mmol), ligand (0.024 mmol), in $PhCF_3$ (2.0 mL) was stirred at 80°C under O_2. [b] Determined by ^1H NMR analysis using 1,1,2,2-tetrachloroethane as internal standard. [c] 1.0 equiv ligand was used.

Table S5. Optimization of temperature[a]

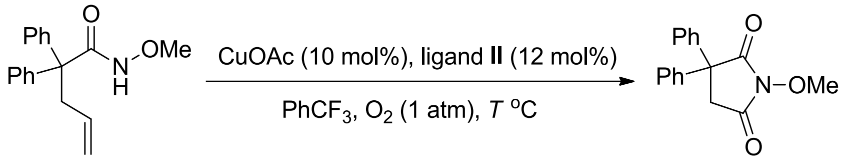

Entry	Temperature (°C)	Yield (%)[b]
1	rt	Trace
2	50	50
3	100	76
4	120	5
5	70	82
6	80	82
7	90	82

[a] Unless noted otherwise, reaction conditions: **1a** (0.20 mmol), CuOAc (0.02 mmol), ligand (0.024 mmol), in $PhCF_3$ (2.0 mL) was stirred at specified temperature under O_2. [b] Determined by ^{1}H NMR analysis using 1,1,2,2-tetrachloroethane as internal standard.

Table S6. Optimization of additives[a]

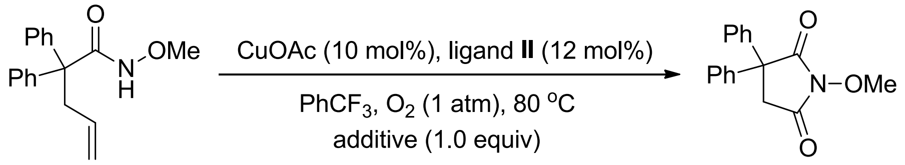

Entry	Additive	Yield (%)[b]
1	10 equiv H_2O	79
2	20 mg 4Å M.S.	78
3	0.2 equiv NABARF[c]	19
4	CsOAc	64
5	K_3PO_4	2
6	K_2CO_3	10
7	Cs_2CO_3	trace
8	DABCO	64
9	PhCOOH	52
10	Pivalic acid	75

[a] Unless noted otherwise, reaction conditions: **1a** (0.20 mmol), CuOAc (0.02 mmol), ligand **II** (0.024 mmol), additives (0.2 mmol) in $PhCF_3$ (2.0 mL) was stirred at 80°C under O_2. [b] Determined by ^{1}H NMR analysis using 1,1,2,2-tetrachloroethane as internal standard. [c] NABARF: Sodium tetrakis[3,5-bis(trifluoromethyl)phenyl]borate.

Table S7. Optimization of the catalyst and ligand loadings[a]

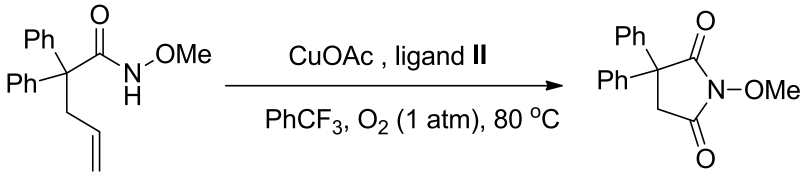

Entry	Loadings	Yield (%)[b]
1	2 mol% CuOAc, 2.4 mol% ligand **II**	47
2	5 mol% CuOAc, 6 mol% ligand **II**	77
3	15 mol% CuOAc, 18 mol% ligand **II**	78
4	20 mol% CuOAc, 24 mol% ligand **II**	81

[a] Unless noted otherwise, reaction conditions: **1a** (0.20 mmol), corresponding CuOAc and ligand **II**, in $PhCF_3$ (2.0 mL) was stirred at 80°C under O_2. [b] Determined by ^{1}H NMR analysis using 1,1,2,2-tetrachloroethane as internal standard.

Table S8. Optimization of reaction concentrations[a]

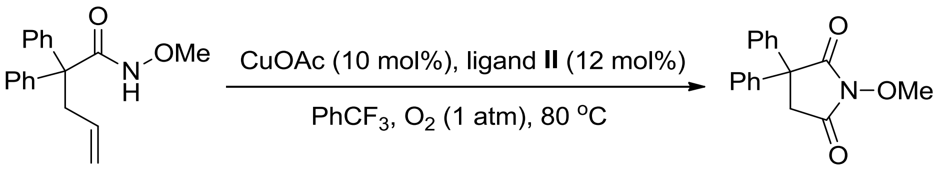

Entry	$PhCF_3$ (mL)	Yield (%)[b]
1[c]	2	81
2	1	76
3	3	84

[a] Unless noted otherwise, reaction conditions: **1a** (0.20 mmol), CuOAc (0.02 mmol), ligand **II** (0.024 mmol), in corresponding volume $PhCF_3$ was stirred at 80°C under O_2. [b] Determined by ^{1}H NMR analysis using 1,1,2,2-tetrachloroethane as internal standard. [c] Air atmosphere instead of O_2.

Table S9. Optimization of solvent, temperature and ligand[a]

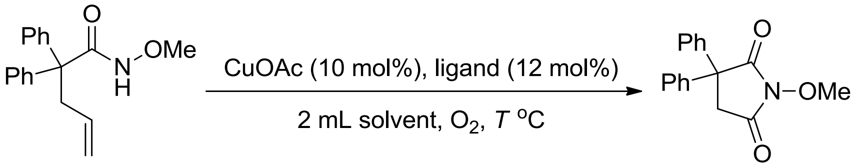

1a **2a**

Entry	Solvent	*T* (°C)	Ligand	Yield (%)[b]
1	PhMe	80	**IV**	67
2	PhCl	80	**IV**	80
3	PhMe	80	**II**	81
4	PhCl	80	**II**	79
5	PhMe	100	**II**	74
6	PhCl	100	**II**	75
7	PhMe	120	**II**	23
8	PhCl	120	**II**	50

[a] Reaction conditions: **1a** (0.20 mmol), CuOAc (0.02 mmol), ligand (0.024 mmol), in solvent (2.0 mL) was stirred at specified temperature under O_2. [b] Determined by ^1H NMR analysis using 1,1,2,2-tetrachloroethane as internal standard.

Table S10. Optimization of reaction concentrations under different solvents[a]

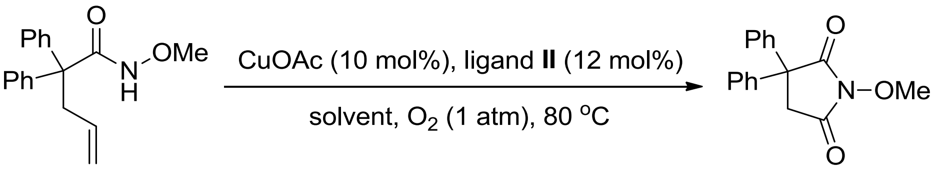

1a **2a**

Entry	Solvent	Yield (%)[b]
1	2 mL PhMe	81
2	2 mL PhCl	79
3	1 mL PhMe	78
4	1 mL PhCl	74
5	3 mL PhMe	70
6	3 mL PhCl	79

[a] Reaction conditions: **1a** (0.20 mmol), CuOAc (0.02 mmol), ligand (0.024 mmol), in solvent was stirred at 80°C under O_2. [b] Determined by ^1H NMR analysis using 1,1,2,2-tetrachloroethane as internal standard.

C. The synthesis of substrates

The synthesis of the substrates were described as follows:

1) *N*-Methoxy-2,2-diphenylpent-4-enamide (1a):

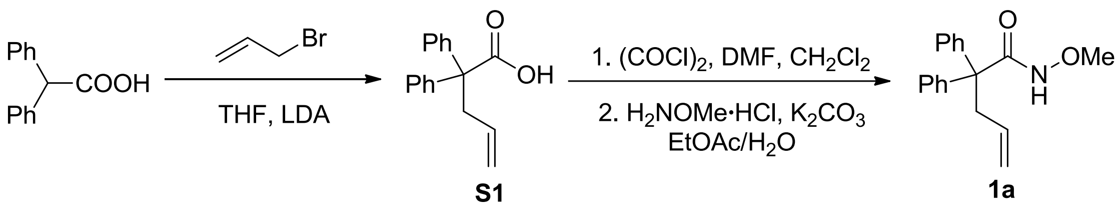

Following a reported procedure with appropriate modifications,[1,2,3] A solution on diphenylacetic acid (4.24 g, 20 mmol, 1.0 equiv) in 16 mL of anhydrous THF was added to freshly prepared LDA (44 mmol, 2.2 equiv) at 0 ℃. The suspension was stirred for 1 h at 25 ℃ and 0.5 h at 60 ℃. The red reaction mixture was cooled to room temperature, then allyl bromide (3.5 mL, 40.0 mmol, 2.0 equiv) was added dropwise. The color dissipated and the reaction mixture was heated at 45 ℃ for 4 hours. Then the reaction mixture was cooled to room temperature and 80 mL of Et_2O and 95 mL of H_2O were added. The layers were separated and the aqueous layer was acidified with 2M HCl and extracted with Et_2O (3 x 70 mL). The organic layer was dried with anhydrous Na_2SO_4, filtered, and the solvent was removed. Purification of the residue by flash chromatography (PE: EA = 4:1) on silica gel to afforded crude 2,2-diphenylpent-4-enoic acid as a white solid.

To a solution of 2,2-diphenylpent-4-enoic acid (20 mmol) in CH_2Cl_2 (20 mL) was added dropwise oxalyl chloride (2.2 mL, 26 mmol) followed by a catalytic amount of DMF. The mixture was stirred at room temperature for 1 h, and then was concentrated under reduced pressure to remove the solvent. The resulting residue was added dropwise to a biphasic mixture of $MeONH_2·HCl$ (2.51 g, 30 mmol) and K_2CO_3 (5.52 g, 40 mmol) in EtOAc (36 mL) and H_2O (18 mL). The reaction was stirred at room temperature for 2 h. The organic layer was separated and the aqueous layer was extracted with EtOAc. The combined extracts were washed with brine, dried over anhydrous Na_2SO_4, and filtrated. The filtrate was concentrated. Purification by column chromatography (PE: EA = 3:1) gave **1a** as a white solid; **^{1}H NMR ($CDCl_3$, 400 MHz)**: δ 8.12 (s, 1H), 7.34–7.25 (m, 10H), 5.82–5.72 (m, 1H), 5.01 (d, *J* = 18.0 Hz, 1H), 4.98 (d, *J* = 11.2 Hz, 1H), 3.69 (s, 3H), 3.22 (d, *J* = 6.8 Hz, 2H); **^{13}C NMR ($CDCl_3$, 100 MHz)**: δ 171.9, 142.0, 134.5, 128.8, 128.3, 127.2, 118.2, 64.1, 58.9, 43.2 ppm; HRMS *m/z* (ESI) calcd for $C_{18}H_{20}NO_2$ $(M + H)^+$,

282.1494, found 282.1496.

2) *N*-(Benzyloxy)-2,2-diphenylpent-4-enamide (1b):

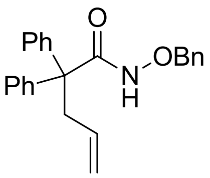

1b

Follow the same procedure with **1a**, using benzylhydroxylamine hydrochloride instead of methoxyammonium chloride as the raw material. Purification by column chromatography (PE: EA = 3:1) gave **1b** as a white solid; **^{1}H NMR ($CDCl_3$, 400 MHz)**: δ 7.98 (s, 1H), 7.30–7.20 (m, 15H), 5.76–5.66 (m, 1H), 4.97–4.92 (m, 2H), 4.83 (s, 2H), 3.18 (d, *J* = 6.8 Hz, 2H); **^{13}C NMR ($CDCl_3$, 100 MHz)**: δ 171.6, 141.9, 134.9, 134.5, 129.2, 128.7, 128.6, 128.4, 128.2, 127.0, 118.1, 77.9, 59.0, 43.1 ppm; HRMS *m/z* (ESI) calcd for $C_{24}H_{24}NO_2$ $(M + H)^+$, 358.1807, found 358.1799.

3) *N*-Ethoxy-2,2-diphenylpent-4-enamide (1c):

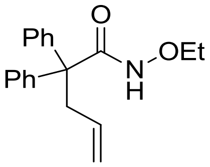

1c

Follow the same procedure with **1a**, using ethoxyamine hydrochloride instead of methoxyammonium chloride as the raw material. Purification by column chromatography (PE: EA = 5:1) gave **1c** as a white solid; **^{1}H NMR ($CDCl_3$, 400 MHz)**: δ 8.06 (s, 1H), 7.34–7.24 (m, 10 H), 5.82–5.71 (m, 1H), 5.00 (d, *J* = 18.1 Hz, 1H), 4.97 (d, *J* = 10.8 Hz, 1H), 3.90 (q, *J* = 7.2 Hz, 2H), 3.22 (d, *J* = 6.8 Hz, 2H), 1.17 (t, *J* = 7.0 Hz, 3H); **^{13}C NMR ($CDCl_3$, 100 MHz)**: δ 171.9, 142.1, 134.6, 128.8, 128.3, 127.1, 118.1, 71.9, 59.0, 43.2, 13.3 ppm; HRMS *m/z* (ESI) calcd for $C_{19}H_{22}NO_2$ $(M + H)^+$, 196.1651, found 296.1652.

4) *N*-isopropoxy-2,2-diphenylpent-4-enamide (1d):

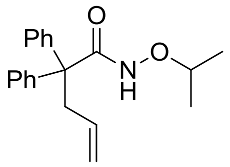

1d

Follow the same procedure with **1a**, using 2-(aminooxy)propane hydrochloride instead of methoxyammonium chloride as the raw material. Purification by column chromatography (PE: EA = 10:1) gave **1d** as a white solid; **^{1}H NMR (CDCl$_3$, 400 MHz)**: δ 7.87 (s, 1H), 7.35–7.25 (m, 10H), 5.81–5.71 (m, 1H), 4.99 (d, *J* = 16.8 Hz, 1H), 4.96 (d, *J* = 10.0 Hz, 1H), 4.14–4.05 (m, 1H), 3.22 (d, *J* = 6.4 Hz, 2H), 1.13 (d, *J* = 6.0 Hz, 6H); **^{13}C NMR (CDCl$_3$, 100 MHz)**: δ 172.1, 142.2, 134.6, 128.8, 128.3, 127.2, 118.1, 77.8, 59.2, 43.3, 20.4 ppm; HRMS *m/z* (ESI) calcd for $C_{20}H_{24}NO_2$ $(M + H)^+$, 310.1807, found 310.1806.

5) *N*-(Tert-butoxy)-2,2-diphenylpent-4-enamide (1e):

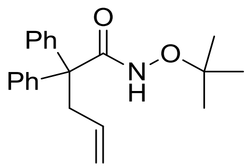

1e

Follow the same procedure with **1a**, using *o*-(tert-butyl)hydroxylamine hydrochloride instead of methoxyammonium chloride as the raw material. Purification by column chromatography (PE: EA = 10:1) gave **1e** as a white solid; **^{1}H NMR (CDCl$_3$, 400 MHz)**: δ 7.57 (s, 1H), 7.36–7.27 (m, 10 H), 5.81–5.71 (m, 1H), 4.98 (d, *J* = 15.2 Hz, 1H), 4.95 (d, *J* = 8.8 Hz, 1H), 3.23 (d, *J* = 6.8 Hz, 2H), 1.12 (s, 9H); **^{13}C NMR (CDCl$_3$, 100 MHz)**: δ 172.7, 142.2, 134.6, 128.9, 128.3, 127.1, 118.1, 82.4, 59.5, 43.4, 26.1 ppm; HRMS *m/z* (ESI) calcd for $C_{21}H_{26}NO_2$ $(M + H)^+$, 324.1964, found 324.1963.

6) 2,2-Diphenylpent-4-enamide (1f):

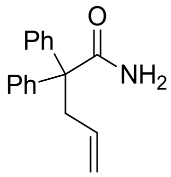

1f

Following a reported procedure.[4] To a solution of 2,2-diphenylpent-4-enoic acid (5.04 g, 20 mmol)

in dry CH_2Cl_2 (20 mL) with dry tube packed with anhydrous calcium chloride was added oxalyl chloride (2.2 mL, 26 mmol) dropwise at room temperature, followed by the addition of 2 drops of DMF. After 8 h, the resulting mixture was concentrated in vacuo and the residue was taken up with CH_2Cl_2 (15 mL). The mixture was added aqueous ammonia solution (28%, 35 mL) dropwise through a funnel in an iced-water bath. After 22 h, the organic layer was separated and the aqueous layer was extracted with CH_2Cl_2 (35 mL × 3). The combined organic layers were dried over anhydrous Na_2SO_4, filtered and concentrated in vacuo. Purification by column chromatography (PE: EA = 2:1) gave **1f** as a white solid; **^{1}H NMR ($CDCl_3$, 400 MHz)**: δ 7.32-7.23 (m, 10H), 6.36 (s, br, 1H), 5.79–5.69 (m, 1H), 5.54 (s, br, 1H), 5.00–4.93 (m, 2H), 3.20 (d, *J* = 6.8 Hz); **^{13}C NMR ($CDCl_3$, 100 MHz)**: δ 176.8 142.8, 135.0, 128.9, 128.2, 127.0, 117.8, 60.4, 43.1 ppm; HRMS *m/z* (ESI) calcd for $C_{17}H_{18}NO$ $(M + H)^+$, 252.1388, found 252.1391.

7) *N*-Benzyl-2,2-diphenylpent-4-enamide (1g):

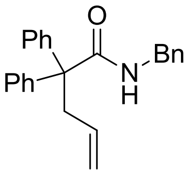

1g

Follow the same procedure with **1a**, using benzylamine hydrochloride instead of methoxyammonium chloride as the raw material. Purification by column chromatography (PE: EA = 10:1) gave **1g** as a white solid; **^{1}H NMR ($CDCl_3$, 400 MHz)**: δ 7.32–7.19 (m, 13H), 7.06 (d, *J* = 7.6 Hz, 2H), 5.84 (s, 1H), 5.80–5.69 (m, 1H), 4.99–4.92 (m, 2H), 4.43 (d, *J* = 6.0 Hz, 2H), 3.25 (d, *J* = 6.8 Hz, 2H); **^{13}C NMR ($CDCl_3$, 100 MHz)**: δ 173.9, 142.8, 138.1, 135.1, 129.0, 128.5, 128.3, 127.33, 127.25, 127.0, 117.8, 60.7, 43.8, 43.4 ppm; HRMS *m/z* (ESI) calcd for $C_{24}H_{24}NO$ $(M + H)^+$, 342.1858, found 342.1856.

8) *N*-Methoxy-2,2-dimethylpent-4-enamide (1h):

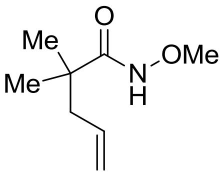

1h

Follow the same procedure with **1a**, using isobutyric acid instead of diphenylacetic acid as the raw

material. Purification by column chromatography (PE: EA = 3:1) gave **1h** as a colorless oil; **^{1}H NMR ($CDCl_3$, 400 MHz)**: δ 8.74 (s, 1H), 5.81–5.70 (m, 1H), 5.12–5.06 (m, 2H), 3.74 (s, 3H), 2.30–2.27 (m, 2H), 1.18 (s, 6H); **^{13}C NMR ($CDCl_3$, 100 MHz)**: δ 175.0, 133.8, 118.3, 64.1, 44.8, 41.2, 24.6 ppm; HRMS *m/z* (ESI) calcd for $C_{24}H_{24}NO$ $(M + H)^+$, 158.1181, found 158.1179.

9) 1-Allyl-*N*-methoxycyclobutanecarboxamide (1i):

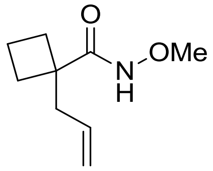

1i

Follow the same procedure with **1a**, using cyclobutanecarboxylic acid as the raw material. Purification by column chromatography (PE: EA = 3:1) gave **1i** as a colorless oil; **^{1}H NMR ($CDCl_3$, 400 MHz)**: δ 8.77 (s, 1H), 5.82–5.71 (m, 1H), 5.16–5.09 (m, 2H), 3.74 (s, 3H), 2.51 (d, *J* = 7.2 Hz, 2H), 2.42–2.35 (m, 2H), 1.99–1.81 (m, 4H); **^{13}C NMR ($CDCl_3$, 100 MHz)**: δ 174.6, 133.4, 118.2, 64.1, 46.1, 42.1, 29.2, 15.3 ppm; HRMS *m/z* (ESI) calcd for $C_9H_{16}NO_2$ $(M + H)^+$, 170.1181, found 170.1180.

10) 1-Allyl-*N*-methoxycyclopentanecarboxamide (1j):

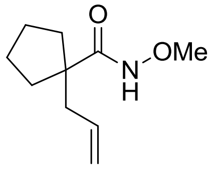

1j

Follow the same procedure with **1a**, using cyclopentanecarboxylic acid instead of diphenylacetic acid as the raw material. Purification by column chromatography (PE: EA = 3:1) gave **1j** as a colorless oil; **^{1}H NMR ($CDCl_3$, 400 MHz)**: δ 8.91 (s, 1H), 5.82–5.71 (m, 1H), 5.11–5.07 (m, 2H), 3.74 (s, 3H), 2.33 (d, *J* = 6.8 Hz, 2H), 2.02–1.99 (m, 2H), 1.67–1.58 (m, 6H); **^{13}C NMR ($CDCl_3$, 100 MHz)**: δ 174.9, 134.3, 117.9, 64.0, 52.7, 42.7, 34.9, 24.2 ppm; HRMS *m/z* (ESI) calcd for $C_{10}H_{18}NO_2$ $(M + H)^+$, 184.1338, found 184.1335.

11) 1-Allyl-*N*-methoxycyclohexanecarboxamide (1k):

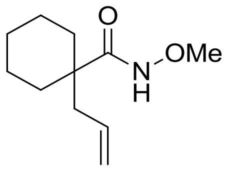

1k

Follow the same procedure with **1a**, using cyclohexanecarboxylic acid instead of diphenylacetic acid as the raw material. Purification by column chromatography (PE: EA = 3:1) gave **1k** as a colorless oil; **^{1}H NMR ($CDCl_3$, 400 MHz)**: δ 8.61 (s, 1H), 5.80–5.70 (m, 1H), 5.09–5.05 (m, 2H), 3.76 (s, 3H), 2.26 (d, J = 7.2 Hz, 2H), 1.95–1.90 (m, 2H), 1.58–1.26 (m, 8H); **^{13}C NMR ($CDCl_3$, 100 MHz)**: δ 173.8, 133.2, 118.3, 64.2, 45.6, 44.2, 33.4, 25.7, 22.6 ppm; HRMS *m/z* (ESI) calcd for $C_{11}H_{20}NO_2$ $(M + H)^+$, 198.1494, found 198.1497.

12) 2-Ethyl-*N*-methoxy-2-methylpent-4-enamide (1l):

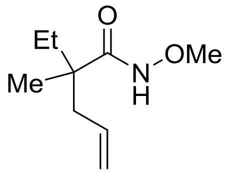

1l

Follow the same procedure with **1a**, using 2-methyl butyric acid instead of diphenylacetic acid as the raw material. Purification by column chromatography (PE: EA = 3:1) gave **1l** as a yellow oil; **^{1}H NMR ($CDCl_3$, 400 MHz)**: δ 9.58 (s, 1H), 5.81–5.70 (m, 1H), 5.11–5.05 (m, 2H), 3.74 (s, 3H), 2.45 (dd, J_1 = 7.2 Hz, J_2 = 14.0 Hz, 1H), 2.15 (dd, J_1 = 7.6 Hz, J_2 = 13.6 Hz, 1H), 1.79–1.70 (m, 1H), 1.49–1.40 (m, 1H), 1.13 (s, 3H), 0.87 (t, J = 7.6 Hz, 3H); **^{13}C NMR ($CDCl_3$, 100 MHz)**: δ 174.0, 133.7, 117.9, 63.7, 44.7, 43.2, 31.7, 19.7, 8.5 ppm; HRMS *m/z* (ESI) calcd for $C_9H_{18}NO_2$ $(M + H)^+$, 172.1338, found 172.1339.

13) *N*-Methoxy-2-methyl-2-phenylpent-4-enamide (1m):

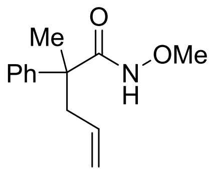

1m

Follow the same procedure with **1a**, using 2-phenylpropionic acid as the raw material. Purification by column chromatography (PE: EA = 3:1) gave **1m** as a colorless oil; **^{1}H NMR ($CDCl_3$, 400

MHz): δ 8.16 (s, 1H), 7.37–7.32 (m, 4H), 7.29–7.25 (m, 1H), 5.59–5.49 (m, 1H), 5.09–5.01 (m, 2H), 3.63 (s, 3H), 2.80 (dd, J_1 = 8.0 Hz, J_2 = 14.0 Hz, 1H), 2.71 (dd, J_1 = 6.8 Hz, J_2 = 14.0 Hz, 1H), 1.53 (s, 3H); **^{13}C NMR ($CDCl_3$, 100 MHz)**: δ 174.3, 142.3, 133.4, 128.6, 127.1, 126.6, 118.6, 63.9, 48.6, 43.3, 23.2 ppm; HRMS *m/z* (ESI) calcd for $C_{13}H_{18}NO_2$ $(M + H)^+$, 220.1338, found 220.1335.

14) 2-Ethyl-*N*-methoxy-2-phenylpent-4-enamide (1n):

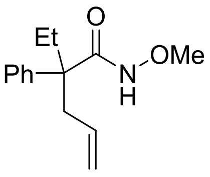

1n

Follow the same procedure with **1a**, using 2-phenylbutyric acid as the raw material. Purification by column chromatography (PE: EA = 3:1) gave **1n** as a colorless oil; **^{1}H NMR ($CDCl_3$, 400 MHz)**: δ 8.23 (s, 1H), 7.36–7.32 (m, 2H), 7.29–7.23 (m, 3H), 5.53–5.42 (m, 1H), 5.06 (d, J = 17.2 Hz, 1H), 5.02 (d, J = 10.4 Hz, 1H), 3.63 (s, 3H), 2.80 (dd, J_1 = 7.6 Hz, J_2 = 14.0 Hz, 1H), 2.70 (dd, J_1 = 6.4 Hz, J_2 = 14.0 Hz, 1H), 2.06–1.97 (m, 2H), 0.81 (t, J = 7.4 Hz, 3H); **^{13}C NMR ($CDCl_3$, 100 MHz)**: δ 173.7, 141.8, 133.1, 128.5, 127.0, 126.9, 118.2, 63.8, 52.4, 38.9, 27.4, 7.9 ppm; HRMS *m/z* (ESI) calcd for $C_{14}H_{20}NO_2$ $(M + H)^+$, 234.1494, found 234.1494.

15) 2-Allyl-*N*-methoxy-2-methylpent-4-enamide (1o):

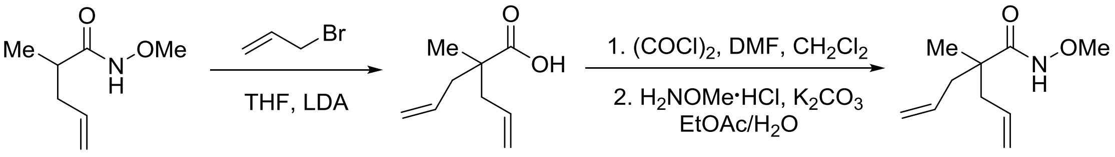

1o

A solution on *N*-methoxy-2-methylpent-4-enamide (2.3 g, 20 mmol, 1.0 equiv) in 16 mL of anhydrous THF was added to freshly prepared LDA (44 mmol, 2.2 equiv) at 0 ℃. The suspension was stirred for 1 h at 25 ℃ and 1.5 h at 60 ℃. The red reaction mixture was cooled to room temperature, then allyl bromide (3.5 mL, 40.0 mmol, 2.0 equiv) was added dropwise. The color dissipated and the reaction mixture was heated at 50 ℃ for 24 hours. Then the reaction mixture was cooled to room temperature and 80 mL of Et_2O and 95 mL of H_2O were added. The layers were separated and the aqueous layer was acidified with 2M HCl and extracted with Et_2O (3 x 70

mL). The organic layer was dried with anhydrous Na_2SO_4, filtered, and the solvent was removed. Purification of the residue by flash chromatography (PE: EA = 4:1) on silica gel to afforded crude 2-allyl-2-methylpent-4-enoic acid as a colorless oil.

To a solution of 2-allyl-2-methylpent-4-enoic acid (20 mmol) in CH_2Cl_2 (20 mL) was added dropwise oxalyl chloride (2.2 mL, 26 mmol) followed by a catalytic amount of DMF. The mixture was stirred at room temperature for 1 h, and then was concentrated under reduced pressure to remove the solvent. The resulting residue was added dropwise to a biphasic mixture of $MeONH_2 \cdot HCl$ (2.51 g, 30 mmol) and K_2CO_3 (5.52 g, 40 mmol) in EtOAc (36 mL) and H_2O (18 mL). The reaction was stirred at room temperature for 2 h. The organic layer was separated and the aqueous layer was extracted with EtOAc. The combined extracts were washed with brine, dried over anhydrous Na_2SO_4, and filtrated. The filtrate was concentrated. Purification by column chromatography (PE: EA = 3:1) gave **1o** as a white solid; **^{1}H NMR ($CDCl_3$, 400 MHz)**: δ 9.58 (s, 1H), 5.81–5.71 (m, 2H), 5.12–5.19 (m, 2H), 5.07 (s, 2H), 3.72 (s, 3H), 2.46 (dd, J_1 = 7.2 Hz, J_2 = 14.0 Hz, 2H), 2.17 (dd, J_1 = 7.6 Hz, J_2 = 13.6 Hz, 2H), 1.14 (s, 3H); **^{13}C NMR ($CDCl_3$, 100 MHz)**: δ 173.6, 133.4, 118.2, 63.7, 44.2, 43.1, 20.2 ppm; HRMS *m/z* (ESI) calcd for $C_{10}H_{18}NO_2$ $(M + H)^+$, 184.1338, found 184.1338.

16) *N*-Methoxy-2-methylpent-4-enamide (1p):

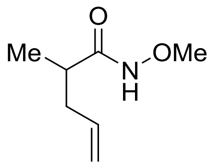

1p

Follow the same procedure with **1a**, using propionic acid instead of diphenylacetic acid as the raw material. Purification by column chromatography (PE: EA = 2:1) gave **1p** as a colorless oil; **^{1}H NMR ($CDCl_3$, 400 MHz)**: δ 9.68 (s, 1H), 5.80–5.70 (m, 1H), 5.10–5.06 (m, 2H), 3.74 (s, 3H), 2.43–2.36 (m, 1H), 2.29–2.24 (m, 1H), 2.19–2.13 (m, 1H), 1.16 (d, J = 6.4 Hz, 3H); **^{13}C NMR ($CDCl_3$, 100 MHz)**: δ 173.7, 135.4, 116.9, 64.0, 37.9, 37.5, 17.0 ppm; HRMS *m/z* (ESI) calcd for $C_7H_{14}NO_2$ $(M + H)^+$, 144.1025, found 144.1021.

17) 2-Benzyl-*N*-methoxypent-4-enamide (1q):

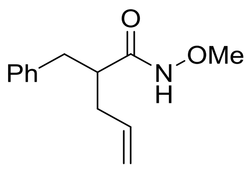

1q

Follow the same procedure with **1a**, using 3-phenylpropionic acid instead of diphenylacetic acid as the raw material. Purification by column chromatography (PE: EA = 3:1) gave **1q** as a colorless oil; **^{1}H NMR ($CDCl_3$, 400 MHz)**: δ 9.59 (s, 1H), 7.26–7.23 (m, 2H), 7.19–7.16 (m, 3H), 5.80–5.71 (m, 1H), 5.09 (d, *J* = 17.2 Hz, 1H), 5.03 (d, *J* = 10.4 Hz, 1H), 3.49 (s, 3H), 2.95–2.90 (m, 1H), 2.77–2.74 (m, 1H), 2.47–2.37 (m, 2H), 2.26–2.20 (m, 1H); **^{13}C NMR ($CDCl_3$, 100 MHz)**: δ 171.8, 139.1, 135.1, 128.9, 128.2, 126.2, 117.1, 63.8, 45.4, 38.1, 36.3 ppm; HRMS *m/z* (ESI) calcd for $C_{13}H_{18}NO_2$ $(M + H)^+$, 220.1338, found 220.1338.

18) 3-Ethyl-*N*-methoxy-2,2-diphenylpent-4-enamide (1r):

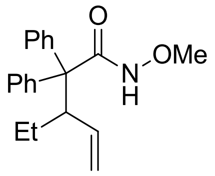

1r

The product **1r** was synthesized according to literature procedure.[3] Purification by column chromatography (PE: EA = 3:1) gave the product **1r** as a white solid; **^{1}H NMR ($CDCl_3$, 400 MHz)**: δ 8.16 (s, 1H), 7.36–7.23 (m, 10H), 5.50–5.41 (m, 1H), 5.11–5.07 (m, 2H), 3.58 (s, 3H), 3.37 (t, *J* = 9.6 Hz, 1H), 1.74–1.66 (m, 1H), 0.91 (t, *J* = 7.2 Hz, 3H), 0.80–0.71 (m, 1H); **^{13}C NMR ($CDCl_3$, 100 MHz)**: δ 171.5, 140.9, 140.5, 137.8, 130.1, 129.7, 127.9, 127.8, 127.1, 127.0, 118.5 63.8, 63.6, 50.4, 23.8, 12.1 ppm; HRMS *m/z* (ESI) calcd for $C_{20}H_{24}NO_2$ $(M + H)^+$, 310.1807, found 310.1805.

19) *N*-Methoxy-2-vinylcyclohexanecarboxamide (1s):

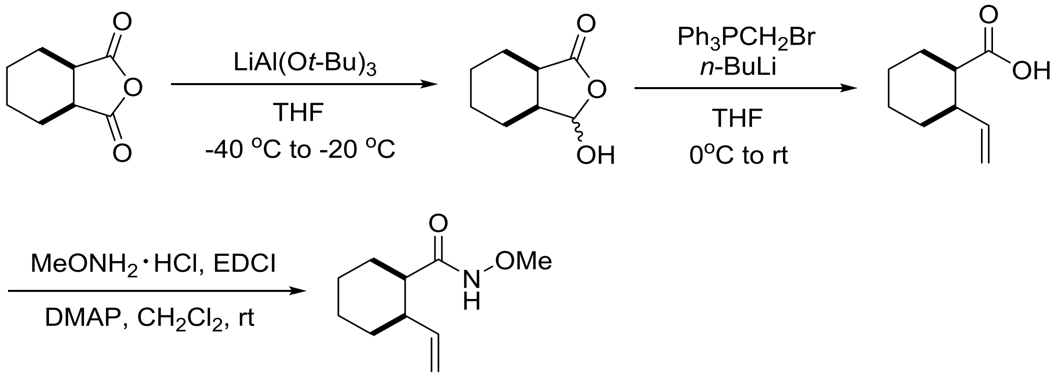

1s

The product **1s** was synthesized according to literature procedure.[5,6] Generally, 1,2-cyclohexanedicarboxylic anhydride (3.08 g, 20 mmol, 1.00 equiv) was dissolved in THF (25 mL) and the resulting solution was cooled to -40 ℃. $LiAl(O\textit{t}\text{-}Bu)_3$ (6.34 g, 25 mmol, 1.25 equiv) was dissolved in THF (40 mL) at -40 ℃ and the resulting off-white solution was cannulated into the flask containing the anhydride solution. The mixture was allowed to warm to -20 ℃ over 6 h (attention was paid not to go over this temperature). The reaction mixture was then quenched by addition of a 10% HCl solution until all solids were dissolved. The aqueous layer was extracted with Et_2O (4 x 30 mL) and the combined organic layers were washed with brine, dried over $MgSO_4$, filtered and concentrated in vacuo. Purification by column chromatography (SiO_2, PE/EtOAc 90/10) afforded lactol as a colorless viscous oil.

n-BuLi (1.6 M in hexanes) (20.7 mL) was added dropwise to a suspension of Ph_3PCH_2Br (11.8 g) in THF (53.7 mL) at 0 ℃. After 30 min, a solution of lactol 37 (2.34g, 15 mmol) in THF (35.3 mL) was cannulated into the ylide solution and the resulting mixture was allowed to warm to room temperature under stirring. The reaction was then quenched by adding a 10% NaOH solution until all solids were dissolved. The aqueous layer was washed with EtOAc (3 x 40 mL). The resulting aqueous solution was acidified with 4 N HCl until pH 3 and extracted with EtOAc (3 x 40 mL). The combined organic layers were dried over $MgSO_4$, filtered and concentrated in vacuo. Purification by column chromatography (SiO_2, PET/EtOAc 90/10) afforded 2-vinylcyclohexanecarboxylic acid as a yellow oil.

To a solution of 2-vinylcyclohexanecarboxylic acid (1.2 g, 8 mmol) in CH_2Cl_2 (40 mL) was added $MeONH_2{\cdot}HCl$ (1.0 mg, 12 mmol), EDCI (3.1 g, 16 mmol) and DMAP (1.96g, 16 mmol) successively. The resulting mixture was stirred at room temperature overnight. The reaction was

quenched by adding HCl (2M, 20 mL). The organic layer was separated and the aqueous layer was extracted with CH_2Cl_2. The combined extracts were washed with brine, dried over Na_2SO_4, and filtrated. The filtrate was concentrated. Purification by column chromatography (PE: EA = 2:1) gave **1s** as a white solid; ^{1}H NMR (400 MHz, $CDCl_3$): δ 8.78 (s, 1H), 6.05–5.96 (m, 1H), 5.12–5.06 (m, 2H), 3.72 (s, 3H), 2.56 (s, br, 1H), 2.36 (s, br, 1H), 1.89–1.73 (m, 3H), 1.66–1.55 (m, 3H), 1.46–1.32 (m, 2H); ^{13}C NMR (100 MHz, $CDCl_3$): δ 172.4, 138.7, 115.7, 64.3, 44.1, 41.9, 30.0, 25.2, 24.0, 22.4 ppm; HRMS *m/z* (ESI) calcd for $C_{10}H_{18}NO_2$ $(M + H)^+$, 184.1338, found 184.1336.

20) *N*-Methoxy-2-vinylbenzamide (1t):

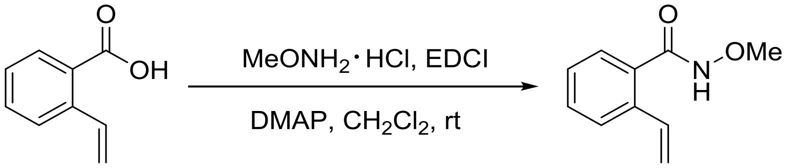

1t

The product **1t** was synthesized according to literature procedure.[5] To a solution of 2-vinylbenzoic acid (712 mg, 4 mmol) in CH_2Cl_2 (20 mL) was added $MeONH_2$·HCl (501 mg, 6 mmol), EDCI (1.54 g, 8 mmol) and DMAP (979 mg, 8 mmol) successively. The resulting mixture was stirred at room temperature overnight. The reaction was quenched by adding HCl (2M, 10 mL). The organic layer was separated and the aqueous layer was extracted with CH_2Cl_2. The combined extracts were washed with brine, dried over Na_2SO_4, and filtrated. The filtrate was concentrated. Purification by column chromatography (PE: EA = 2:1) gave the product as a white solid. **^{1}H NMR ($CDCl_3$, 400 MHz)**: δ 8.95 (s, 1H), 7.56 (d, J = 8.0 Hz, 1H), 7.42–7.35 (m, 2H), 7.25 (t, J = 7.6 Hz, 1H), 6.98 (dd, J_1 = 11.2 Hz, J_2 = 17.6 Hz, 1H), 5.71 (d, J = 17.2 Hz, 1H), 5.34 (d, J = 10.8 Hz, 1H), 3.82 (s, 3H); **^{13}C NMR ($CDCl_3$, 100 MHz)**: δ 167.2, 136.4, 133.8, 131.6, 130.7, 127.7, 127.5, 126.1, 117.1, 64.4 ppm; HRMS *m/z* (ESI) calcd for $C_{10}H_{12}NO_2$ $(M + H)^+$, 178.0868, found 178.0867.

21) 2-(1,3-Dioxoisoindolin-2-yl)-*N*-methoxypent-4-enamide (1u):

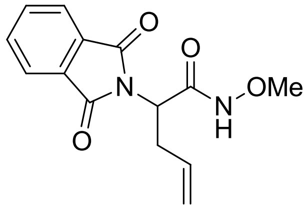

1u

The product **1u** was synthesized according to literature procedure,[7] Using 2-Allylglycine as the raw material. Purification by column chromatography (PE: EA = 2:1) gave **1u** as a white solid; **^{1}H NMR ($CDCl_3$, 400 MHz)**: δ 9.55 (s, 1H), 7.86–7.84 (m, 2H), 7.77–7.74 (m, 2H), 5.72–5.62 (m, 1H), 5.07 (d, *J* = 17.2 Hz, 1H), 5.00 (d, *J* = 10.0 Hz, 1H), 4.87 (s, br, 1H), 3.74 (s, 3H), 3.00–2.87 (m, 2H); **^{13}C NMR ($CDCl_3$, 100 MHz)**: δ 167.9, 166.5, 134.4, 132.8, 131.4, 123.6, 119.3, 64.4, 52.4, 33.2 ppm; HRMS *m/z* (ESI) calcd for $C_{14}H_{15}N_2O_4$ $(M + H)^+$, 275.1032, found 275.1033.

22) 2-(4-Isobutylphenyl)-*N*-methoxy-2-methylpent-4-enamide (5):

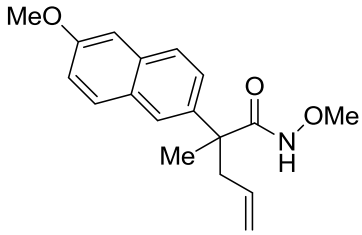

5

Follow the same procedure with **1a**, using naproxen instead of diphenylacetic acid as the raw material. Purification by column chromatography (PE: EA = 3:1) gave **7** as a colorless oil; **^{1}H NMR ($CDCl_3$, 400 MHz)**: δ 8.26 (s, 1H), 7.70 (d, *J* = 2.4 Hz, 1H), 7.68–7.66 (m, 2H), 7.36 (dd, J_1 = 2.0 Hz, J_2 = 8.8 Hz, 1H), 7.14 (dd, J_1 = 2.4 Hz, J_2 = 8.8 Hz, 1H), 7.10 (d, *J* = 2.4 Hz, 1H), 5.57–5.47 (m, 1H), 5.06 (d, *J* = 17.2 Hz, 1H), 5.00 (d, *J* = 10.0 Hz, 1H), 3.88 (s, 3H), 3.60 (s, 3H), 2.87–2.75 (m, 2H), 1.59 (s, 3H); **^{13}C NMR ($CDCl_3$, 100 MHz)**: δ 174.3, 157.8, 137.2, 133.4, 133.3, 129.3, 128.5, 127.2, 125.5, 124.9, 119.0, 118.5, 105.4, 63.8, 55.2, 48.4, 43.0, 23.1 ppm; HRMS *m/z* (ESI) calcd for $C_{17}H_{26}NO_2$ $(M + H)^+$, 276.1964, found 276.1960.

23) *N*-Methoxy-2-(6-methoxynaphthalen-2-yl)-2-methylpent-4-enamide (7):

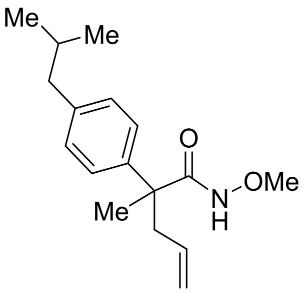

7

Follow the same procedure with **1a**, using ibuprofen instead of diphenylacetic acid as the raw material. Purification by column chromatography (PE: EA = 3:1) gave **5** as a colorless oil; **^{1}H NMR (CDCl$_3$, 400 MHz)**: δ 8.03 (s, 1H), 7.23 (d, J = 8.4 Hz, 2H), 7.13 (d, J = 8.0 Hz, 2H), 5.59–5.49 (m, 1H), 5.07 (d, J = 17.6 Hz, 1H), 5.02 (d, J = 10.4 Hz, 1H), 3.65 (s, 3H), 2.79 (dd, J_1 = 8.0 Hz, J_2 = 14.0 Hz, 1H), 2.70 (dd, J_1 = 6.4 Hz, J_2 = 14.0 Hz, 1H), 2.46 (d, J = 7.2 Hz, 2H), 1.91–1.81 (m, 1H), 1.52 (s, 3H), 0.90 (d, J = 6.4 Hz, 6H) ; **^{13}C NMR (CDCl$_3$, 100 MHz)**: δ 174.6, 140.7, 139.4, 133.6, 129.4, 126.4, 118.4, 64.0, 48.3, 44.8, 43.3, 30.0, 23.4, 22.3 ppm; HRMS *m/z* (ESI) calcd for $C_{18}H_{22}NO_3$ $(M + H)^+$, 300.1600, found 300.1598.

24) 2-(2-Fluoro-[1,1'-biphenyl]-4-yl)-*N*-methoxy-2-methylpent-4-enamide (9):

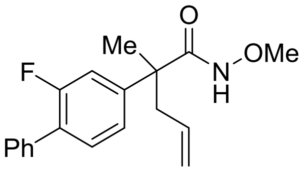

9

Follow the same procedure with **1a**, using flurbiprofen instead of diphenylacetic acid as the raw material. Purification by column chromatography (PE: EA = 3:1) gave **9** as a white solid; **^{1}H NMR (CDCl$_3$, 400 MHz)**: δ 8.27 (s, 1H), 7.55–7.53 (m, 2H), 7.46–7.35 (m, 4H), 7.20–7.13 (m, 2H), 5.63–5.53 (m, 1H), 5.14–5.07 (m, 2H), 3.69 (s, 3H), 2.83 (dd, J_1 = 8.0 Hz, J_2 = 14.0 Hz, 1H), 2.72 (dd, J_1 = 6.8 Hz, J_2 = 14.0 Hz, 1H), 1.56 (s, 3H); **^{13}C NMR (CDCl$_3$, 100 MHz)**: δ 173.5, 159.6 (d, J_{CF} = 247.3), 143.9 (d, J_{CF} = 7.1 Hz), 135.0, 133.0, 130.8 (d, J_{CF} = 4.1 Hz), 128.8 (d, J_{CF} = 2.9 Hz), 128.4, 127.8, 127.7, 122.7 (d, J_{CF} = 3.4 Hz), 119.1, 114.6 (d, J_{CF} = 24.1 Hz), 64.1, 48.5, 43.3, 23.1 ppm; HRMS *m/z* (ESI) calcd for $C_{19}H_{21}NO_2F$ $(M + H)^+$, 314.1556, found 314.1559.

25) *N*-(Benzyloxy)-2-ethyl-2-methylpent-4-enamide (11):

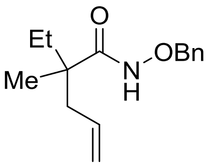

11

Follow the same procedure with **1a**, using 2-methyl butyric acid and benzylhydroxylamine hydrochloride instead of diphenylacetic acid and methoxyammonium chloride as the raw material. Purification by column chromatography (PE: EA = 5:1) gave **11** as a colorless oil; **^{1}H NMR ($CDCl_3$, 400 MHz)**: δ 9.03 (s, 1H), 7.41–7.38 (m, 2H), 7.35–7.28 (m, 3H), 5.74–5.64 (m, 1H), 5.05–5.00 (m, 2H), 4.89 (s, 2H), 2.40 (dd, J_1 = 7.2 Hz, J_2 = 14.0 Hz, 1H), 2.10 (dd, J_1 = 8.0 Hz, J_2 = 13.6 Hz, 1H), 1.73–1.64 (m, 1H), 1.45–1.36 (m, 1H), 1.06 (s, 3H), 0.83 (t, J = 7.2 Hz, 3H); **^{13}C NMR ($CDCl_3$, 100 MHz)**: δ 174.0, 135.2, 133.7, 129.0, 128.3, 128.2, 117.9, 77.7, 44.8, 43.1, 31.7, 19.8, 8.5 ppm; HRMS *m/z* (ESI) calcd for $C_{15}H_{22}NO_2$ $(M + H)^+$, 248.1651, found 248.1650.

26) *N*-(Benzyloxy)-2-methyl-2-phenylpent-4-enamide (14):

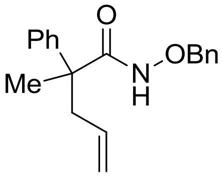

14

Follow the same procedure with **1a**, using 2-phenylpropionic acid and benzylhydroxylamine hydrochloride instead of diphenylacetic acid and methoxyammonium chloride as the raw material. Purification by column chromatography (PE: EA = 5:1) gave **14** as a colorless oil; **^{1}H NMR ($CDCl_3$, 400 MHz)**: δ 8.23 (s, 1H), 7.30–7.20 (m, 10H), 5.54–5.44 (m, 1H), 5.04–4.96 (m, 2H), 4.77 (s, 2H), 2.77 (dd. J_1 = 7.6 Hz, J_2 = 14.0 Hz, 1H), 2.66 (dd, J_1 = 6.8 Hz, J_2 = 14.0 Hz, 1H), 1.47 (s, 3H); **^{13}C NMR ($CDCl_3$, 100 MHz)**: δ 173.7, 142.1, 134.8, 133.4, 129.0, 128.4, 128.3, 128.1, 126.8, 126.3, 118.3, 77.5, 48.4, 43.1, 22.8 ppm; HRMS *m/z* (ESI) calcd for $C_{19}H_{22}NO_2$ $(M + H)^+$, 296.1651, found 296.1651.

27) *N*-Methoxy-2,2-diphenylhex-5-enamide (3a):

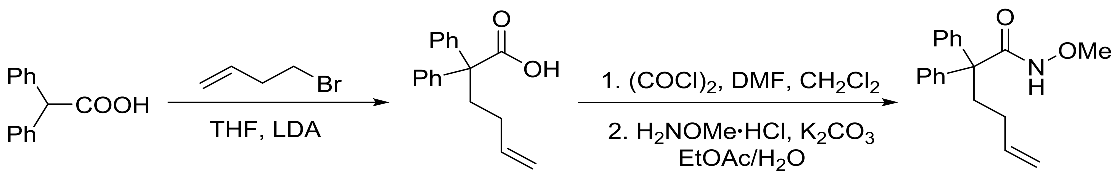

3a

Following a reported procedure with appropriate modifications,[1,5] A solution on diphenylacetic acid (4.24 g, 20 mmol, 1.0 equiv) in 16 mL of anhydrous THF was added to freshly prepared LDA (44 mmol, 2.2 equiv) at 0 ℃. The suspension was stirred for 1 h at 25 ℃ and 0.5 h at 60 ℃. The red reaction mixture was cooled to room temperature, then 4-bromo-1-butene (4.1 mL, 40.0 mmol, 2.0 equiv) was added dropwise. The color dissipated and the reaction mixture was heated at 45 ℃ for 4 hours. Then the reaction mixture was cooled to room temperature and 80 mL of Et_2O and 95 mL of H_2O were added. The layers were separated and the aqueous layer was acidified with 2M HCl and extracted with Et_2O (3 x 70 mL). The organic layer was dried with anhydrous Na_2SO_4, filtered, and the solvent was removed. Purification of the residue by flash chromatography (PE: EA = 4:1) on silica gel to afforded crude 2,2-diphenylhex-5-enoic acid.

To a solution of 2,2-diphenylhex-5-enoic acid (20 mmol) in CH_2Cl_2 (20 mL) was added dropwise oxalyl chloride (2.2 mL, 26 mmol) followed by a catalytic amount of DMF. The mixture was stirred at room temperature for 1 h, and then was concentrated under reduced pressure to remove the solvent. The resulting residue was added dropwise to a biphasic mixture of $MeONH_2 \cdot HCl$ (2.51 g, 30 mmol) and K_2CO_3 (5.52 g, 40 mmol) in EtOAc (36 mL) and H_2O (18 mL). The reaction was stirred at room temperature for 2 h. The organic layer was separated and the aqueous layer was extracted with EtOAc. The combined extracts were washed with brine, dried over anhydrous Na_2SO_4, and filtrated. The filtrate was concentrated. Purification by column chromatography (PE: EA = 3:1) gave **3a** as a white solid; **^{1}H NMR ($CDCl_3$, 400 MHz)**: δ 8.08 (s, 1H), 7.35–7.25 (m, 10H), 5.83–5.73 (m, 1H), 4.98 (d, *J* = 17.2 Hz, 1H), 4.92 (d, *J* = 10.4 Hz, 1H), 3.66 (s, 3H), 2.48 (t, *J* = 7.8 Hz, 2H), 2.02–1.96 (m, 2H); **^{13}C NMR ($CDCl_3$, 100 MHz)**: δ 172.2, 142.1, 138.2, 128.6, 128.4, 127.1, 114.4, 64.0, 58.8, 37.9, 29.6 ppm; HRMS *m/z* (ESI) calcd for $C_{19}H_{22}NO_2$ $(M + H)^+$, 296.1651, found 296.1644.

28) *N*-Ethoxy-2,2-diphenylhex-5-enamide (3b):

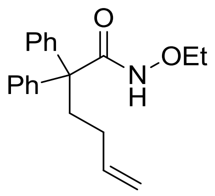

3b

Follow the same procedure with **3a**, using ethoxyamine hydrochloride instead of methoxyammonium chloride as the raw material. Purification by column chromatography (PE: EA = 3:1) gave **3b** as a white solid; **^{1}H NMR (CDCl$_3$, 400 MHz)**: δ 7.95 (s, 1H), 7.35–7.25 (m, 10H), 5.84–5.73 (m, 1H), 4.98 (d, *J* = 17.2 Hz, 1H), 4.91 (d, *J* = 10.0 Hz, 1H), 3.88 (q, *J* = 7.2 Hz, 2H), 2.51–2.46 (m, 2H), 1.99 (q, *J* = 7.2 Hz, 2H), 1.16 (t, *J* = 7.2 Hz, 3H) ; **^{13}C NMR (CDCl$_3$, 100 MHz)**: δ 172.2, 142.2, 138.3, 128.6, 128.4, 127.1, 114.4, 71.9, 58.9, 38.1, 29.6, 13.3 ppm; HRMS *m/z* (ESI) calcd for $C_{20}H_{24}NO_2$ $(M + H)^+$, 310.1807, found 310.1808.

29) *N*-Methoxy-2-methyl-2-phenylhex-5-enamide (3c):

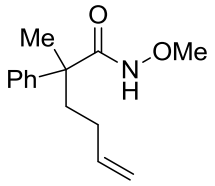

3c

Follow the same procedure with **3a**, using 2-phenylpropionic acid instead of diphenylacetic acid as the raw material. Purification by column chromatography (PE: EA = 3:1) gave **3c** as a colorless oil; **^{1}H NMR (CDCl$_3$, 400 MHz)**: δ 8.23 (s, 1H), 7.37–7.25 (m, 5H), 5.83–5.73 (m, 1H), 4.99 (d, *J* = 17.2 Hz, 1H), 4.93 (d, *J* = 10.4 Hz, 1H), 3.62 (s, 3H), 2.17–1.95 (m, 3H), 1.88–1.79 (m, 1H), 1.56 (s, 3H); **^{13}C NMR (CDCl$_3$, 100 MHz)**: δ 174.5, 142.6, 138.1, 128.6, 127.0, 126.5, 114.5, 63.8, 48.8, 37.8, 28.3, 23.1 ppm; HRMS *m/z* (ESI) calcd for $C_{14}H_{20}NO_2$ $(M + H)^+$, 234.1494, found 234.1493.

30) 2-Ethyl-*N*-methoxy-2-phenylhex-5-enamide (3d):

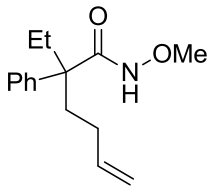

3d

Follow the same procedure with **3a**, using 2-phenylbutyric acid instead of diphenylacetic acid as

the raw material. Purification by column chromatography (PE: EA = 3:1) gave **3d** as a colorless oil; **^{1}H NMR ($CDCl_3$, 400 MHz)**: δ 8.26 (s, 1H), 7.35–7.23 (m, 5H), 5.83–5.73 (m, 1H), 4.99 (d, *J* = 17.2 Hz, 1H), 4.93 (d, *J* = 10.0 Hz, 1H), 3.62 (s, 3H), 2.12–1.98 (m, 4H), 1.95–1.86 (m, 1H), 1.83–1.73 (m, 1H), 0.78 (t, *J* = 7.4 Hz, 3H); **^{13}C NMR ($CDCl_3$, 100 MHz)**: δ 174.0, 142.1, 138.1, 128.5, 126.89, 126.87, 114.4, 63.7, 52.5, 33.3, 27.8, 27.3, 7.9 ppm; HRMS *m/z* (ESI) calcd for $C_{15}H_{22}NO_2$ $(M + H)^+$, 248.1651, found 248.1647.

D. The general experimental procedure and the characterization data of products.

The general experimental procedure:

The reaction of *N*-alkoxy enamide **1** (0.2 mmol), CuOAc (0.02 mmol, 2.5 mg) and ligand **II** (0.024 mmol, 8.7 mg) were stirred in 2 mL PhMe at 80 ℃ under oxygen (with a dioxygen ballon, 1 atm) for 18 h. After cooling down to room temperature, the mixture was concentrated and purified by silica gel column chromatography (with ethyl acetate/petroleum ether as the eluent) to afford the desired products **2**.

1) 1-Methoxy-3,3-diphenylpyrrolidine-2,5-dione (2a):

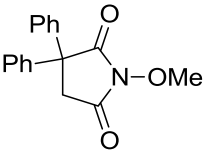

2a

The reaction of *N*-methoxy-2,2-diphenylpent-4-enamide **1a** (0.2 mmol, 56.3 mg), CuOAc (0.02 mmol, 2.5 mg) and ligand **II** (0.024 mmol, 8.7 mg) were stirred in 2 mL PhMe at 80 ℃ under oxygen for 18 h. The resulting mixture was purified by flash chromatography on silica gel (eluent: petroleum ether/ethyl acetate = 5:1) to afford 46.8 mg (83% yield) of **2a** as white solid: **^{1}H NMR ($CDCl_3$, 400 MHz)**: δ 7.38–7.28 (m, 10H), 3.96 (s, 3H), 3.44 (s, 2H); **^{13}C NMR ($CDCl_3$, 100 MHz)**: δ 172.5, 169.1, 140.9, 129.0, 127.9, 127.2, 64.7, 54.3, 42.5 ppm; HRMS *m/z* (ESI) calcd for $C_{17}H_{16}NO_3$ $(M + H)^+$, 282.1125, found 282.1123.

2) 1-(Benzyloxy)-3,3-diphenylpyrrolidine-2,5-dione (2b):

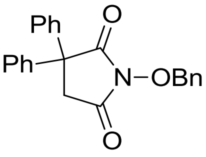

2b

The reaction of *N*-(benzyloxy)-2,2-diphenylpent-4-enamide **1b** (0.2 mmol, 71.5 mg), CuOAc

(0.02 mmol, 2.5 mg) and ligand **II** (0.024 mmol, 8.7 mg) were stirred in 2 mL PhMe at 80 ℃ under oxygen for 18 h to afford 53.1 mg (74% yield) of **2b** as white solid: **^{1}H NMR ($CDCl_3$, 400 MHz)**: δ 7.43–7.41 (m, 2H), 7.38–7.26 (m, 9H), 7.15–7.13 (m, 4H), 5.15 (s, 2H), 3.33 (s, 2H); **^{13}C NMR ($CDCl_3$, 100 MHz)**: δ 172.9, 169.3, 141.0, 133.1, 130.1, 129.4, 128.8, 128.5, 127.8, 127.3, 78.5, 54.2, 42.4 ppm; HRMS *m/z* (ESI) calcd for $C_{23}H_{20}NO_3$ $(M + H)^+$, 358.1438, found 358.1438.

3) 1-Ethoxy-3,3-diphenylpyrrolidine-2,5-dione (2c):

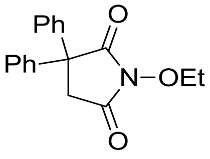

2c

The reaction of *N*-ethoxy-2,2-diphenylpent-4-enamide **1c** (0.2 mmol, 59.1 mg), CuOAc (0.02 mmol, 2.5 mg) and ligand **II** (0.024 mmol, 8.7 mg) were stirred in 2 mL PhMe at 80 ℃ under oxygen for 18 h to afford 49.6 mg (84 % yield) of **2c** as white solid: **^{1}H NMR ($CDCl_3$, 400 MHz)**: δ 7.37–7.28 (m, 10 H), 4.18 (q, J = 7.1 Hz, 2H), 3.44 (s, 2H), 1.33 (t, J = 7.2 Hz, 3H); **^{13}C NMR ($CDCl_3$, 100 MHz)**: δ 173.0, 169.5, 141.0, 128.9, 127.9, 127.2, 73.1, 54.2, 42.5, 13.4 ppm; HRMS *m/z* (ESI) calcd for $C_{18}H_{18}NO_3$ $(M + H)^+$, 296.1281, found 296.1281.

4) 1-Isopropoxy-3,3-diphenylpyrrolidine-2,5-dione (2d):

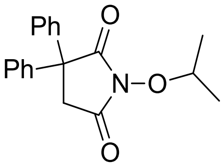

2d

The reaction of *N*-isopropoxy-2,2-diphenylpent-4-enamide **1d** (0.2 mmol, 61.9 mg), CuOAc (0.02 mmol, 2.5 mg) and ligand **II** (0.024 mmol, 8.7 mg) were stirred in 2 mL PhMe at 80 ℃ under oxygen for 18 h to afford 33.6 mg (54% yield) of **2d** as colorless oil: **^{1}H NMR ($CDCl_3$, 400 MHz)**: δ 7.38–7.28 (m, 10H), 4.56–4.47 (m, 1H), 3.44 (s, 2H), 1.27 (d, J = 6.4 Hz, 6H); **^{13}C NMR ($CDCl_3$, 100 MHz)**: δ 173.7, 170.1, 141.1, 128.9, 127.9, 127.3, 79.9, 54.3, 42.5, 20.7 ppm; HRMS *m/z* (ESI) calcd for $C_{19}H_{20}NO_3$ $(M + H)^+$, 310.1438, found 310.1438.

5) 1-(*tert*-Butoxy)-3,3-diphenylpyrrolidine-2,5-dione (2e):

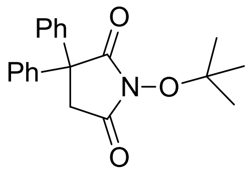

2e

The reaction of *N*-(tert-butoxy)-2,2-diphenylpent-4-enamide **1e** (0.2 mmol, 64.7 mg), CuOAc (0.02 mmol, 2.5 mg) and ligand **II** (0.024 mmol, 8.7 mg) were stirred in 2 mL PhMe at 80 ℃ under oxygen for 18 h to afford 21.4 mg (33% yield) of **2e** as colorless oil: **^{1}H NMR ($CDCl_3$, 400 MHz)**: δ 7.37–7.29 (m, 10H), 3.45 (s, 2H), 1.31 (s, 9H); **^{13}C NMR ($CDCl_3$, 100 MHz)**: δ 174.9, 171.2, 141.3, 128.9, 127.8, 127.3, 87.8, 54.4, 42.5, 27.3 ppm; HRMS *m/z* (ESI) calcd for $C_{20}H_{22}NO_3$ $(M + H)^+$, 324.1594, found 324.1595.

6) 1-Methoxy-3,3-dimethylpyrrolidine-2,5-dione (2h):

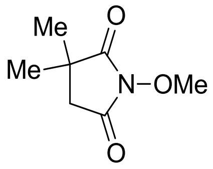

2h

The reaction of *N*-methoxy-2,2-dimethylpent-4-enamide **1h** (0.2 mmol, 31.4 mg), CuOAc (0.02 mmol, 2.5 mg) and ligand **II** (0.024 mmol, 8.7 mg) were stirred in 2 mL PhMe at 80 ℃ under oxygen for 18 h to afford 18.2 mg (58% yield) of **2h** as colorless oil: **^{1}H NMR ($CDCl_3$, 400 MHz)**: δ 3.95 (s, 3H), 2.54 (s, 2H), 1.35 (s, 6H); **^{13}C NMR ($CDCl_3$, 100 MHz)**: δ 177.1, 169.8, 64.5, 41.1, 37.6, 25.4 ppm; HRMS *m/z* (ESI) calcd for $C_7H_{12}NO_3$ $(M + H)^+$, 158.0812, found 158.0809.

7) 6-Methoxy-6-azaspiro[3.4]octane-5,7-dione (2i):

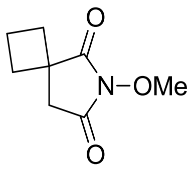

2i

The reaction of 1-allyl-*N*-methoxycyclobutanecarboxamide **1i** (0.2 mmol, 33.8 mg), CuOAc (0.02 mmol, 2.5 mg) and ligand **II** (0.024 mmol, 8.7 mg) were stirred in 2 mL PhMe at 80 ℃ under oxygen for 18 h to afford 16.2 mg (48% yield) of **2i** as colorless oil: **^{1}H NMR ($CDCl_3$, 400 MHz)**:

δ 3.94 (s, 3H), 2.82 (s, 2H), 2.69–2.62 (m, 2H), 2.25–2.14 (m, 1H), 2.12–2.02 (m, 3H); **^{13}C NMR ($CDCl_3$, 100 MHz)**: δ 175.9, 169.9, 64.6, 41.5, 40.4, 31.6, 16.2 ppm; HRMS *m/z* (ESI) calcd for $C_8H_{12}NO_3$ $(M + H)^+$, 170.0812, found 170.0812.

8) 2-Methoxy-2-azaspiro[4.4]nonane-1,3-dione (2j):

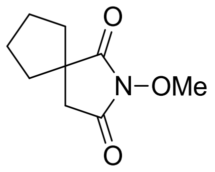

2j

The reaction of 1-allyl-*N*-methoxycyclopentanecarboxamide **1j** (0.2 mmol, 36.7 mg), CuOAc (0.02 mmol, 2.5 mg) and ligand **II** (0.024 mmol, 8.7 mg) were stirred in 2 mL PhMe at 80 ℃ under oxygen for 18 h to afford 15.4 mg (42% yield) of **2j** as colorless oil: **^{1}H NMR ($CDCl_3$, 400 MHz)**: δ 3.96 (s, 3H), 2.57 (s, 2H), 2.24–2.12 (m, 2H), 1.98–1.87 (m, 2H), 1.80–1.64 (m, 4H); **^{13}C NMR ($CDCl_3$, 100 MHz)**: δ 177.2, 170.3, 64.6, 47.6, 41.3, 38.3, 25.1 ppm; HRMS *m/z* (ESI) calcd for $C_9H_{14}NO_3$ $(M + H)^+$, 184.0968, found 184.0964.

9) 2-Methoxy-2-azaspiro[4.5]decane-1,3-dione (2k):

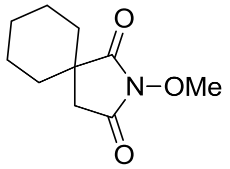

2k

The reaction of 1-allyl-*N*-methoxycyclohexanecarboxamide **1k** (0.2 mmol, 39.5 mg), CuOAc (0.02 mmol, 2.5 mg) and ligand **II** (0.024 mmol, 8.7 mg) were stirred in 2 mL PhMe at 80 ℃ under oxygen for 18 h to afford 20.9 mg (53% yield) of **2k** as white solid: **^{1}H NMR ($CDCl_3$, 400 MHz)**: δ 3.94 (s, 3H), 2.52 (s, 2H), 1.89–1.78 (m, 4H), 1.68 (s, 1H), 1.51 (d, *J* = 11.6 Hz, 2H), 1.37–1.30 (m, 3H); **^{13}C NMR ($CDCl_3$, 100 MHz)**: δ 176.9, 170.3, 64.6, 42.1, 37.7, 33.2, 24.7, 21.6 ppm; HRMS *m/z* (ESI) calcd for $C_{10}H_{16}NO_3$ $(M + H)^+$, 198.1125, found 198.1123.

10) 3-Ethyl-1-methoxy-3-methylpyrrolidine-2,5-dione (2l):

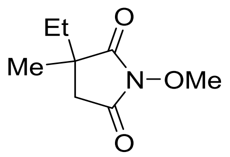

2l

The reaction of 2-ethyl-*N*-methoxy-2-methylpent-4-enamide **1l** (0.2 mmol, 34.2 mg), CuOAc (0.02 mmol, 2.5 mg) and ligand **II** (0.024 mmol, 8.7 mg) were stirred in 2 mL PhMe at 80 ℃ under oxygen for 18 h to afford 19.5 mg (57% yield) of **2l** as yellow oil: **^{1}H NMR (CDCl$_3$, 400 MHz)**: δ 3.94 (s, 3H), 2.61 (d, *J* = 18.0 Hz, 1H), 2.42 (d, *J* = 18.0 Hz, 1H), 1.82–1.73 (m, 1H), 1.67–1.58 (m, 1H), 1.33 (s, 3H), 0.92 (t, *J* = 7.6 Hz, 3H); **^{13}C NMR (CDCl$_3$, 100 MHz)**: δ 176.6, 170.0, 64.4, 41.4, 37.8, 30.9, 23.7, 8.3 ppm; HRMS *m/z* (ESI) calcd for $C_8H_{14}NO_3$ $(M + H)^+$, 172.0968, found 172.0968.

11) 1-Methoxy-3-methyl-3-phenylpyrrolidine-2,5-dione (2m):

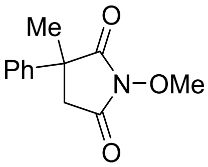

2m

The reaction of *N*-methoxy-2-methyl-2-phenylpent-4-enamide **1m** (0.2 mmol, 43.9 mg), CuOAc (0.02 mmol, 2.5 mg) and ligand **II** (0.024 mmol, 8.7 mg) were stirred in 2 mL PhMe at 80 ℃ under oxygen for 18 h to afford 29.9 mg (68% yield) of **2m** as colorless oil: **^{1}H NMR (CDCl$_3$, 400 MHz)**: δ 7.41–7.29 (m, 5H), 3.99 (s, 3H), 3.10 (d, *J* = 18.0 Hz, 1H), 2.84 (d, *J* = 18.0 Hz, 1H), 1.74 (s, 3H); **^{13}C NMR (CDCl$_3$, 100 MHz)**: δ 174.9, 169.5, 141.0, 129.1, 127.8, 125.4, 64.7, 45.2, 42.7, 25.7 ppm; HRMS *m/z* (ESI) calcd for $C_{12}H_{14}NO_3$ $(M + H)^+$, 220.0968, found 220.0968.

12) 3-Ethyl-1-methoxy-3-phenylpyrrolidine-2,5-dione (2n):

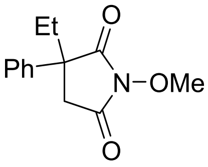

2n

The reaction of 2-ethyl-*N*-methoxy-2-phenylpent-4-enamide **1n** (0.2 mmol, 46.7 mg), CuOAc (0.02 mmol, 2.5 mg) and ligand **II** (0.024 mmol, 8.7 mg) were stirred in 2 mL PhMe at 80 ℃ under oxygen for 18 h to afford 33.6 mg (72% yield) of **2n** as colorless oil: **^{1}H NMR (CDCl$_3$, 400 MHz)**: δ 7.44–7.29 (m, 5H), 3.96 (s, 3H), 3.08 (d, *J* = 18.0 Hz, 1H), 2.90 (d, *J* = 18.0 Hz, 1H), 2.17–2.03 (m, 2H), 0.92 (t, *J* = 7.2 Hz, 3H); **^{13}C NMR (CDCl$_3$, 100 MHz)**: δ 174.2, 169.7, 139.9, 129.0, 127.7, 125.9, 49.5, 38.8, 32.7, 8.8 ppm; HRMS *m/z* (ESI) calcd for $C_{13}H_{16}NO_3$ $(M + H)^+$,

234.1125, found 234.1123.

13) 3-Allyl-1-methoxy-3-methylpyrrolidine-2,5-dione (2o):

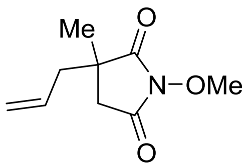

2o

The reaction of 2-allyl-*N*-methoxy-2-methylpent-4-enamide **1o** (0.2 mmol, 36.6 mg), CuOAc (0.02 mmol, 2.5 mg) and ligand **II** (0.024 mmol, 8.7 mg) were stirred in 2 mL PhMe at 80 ℃ under oxygen for 18 h to afford 15.0 mg (44% yield) of **2o** as colorless oil: **^{1}H NMR ($CDCl_3$, 400 MHz)**: δ 5.71–5.61 (m, 1H), 5.21 (s, 1H), 5.18–5.17 (m, 1H), 3.93 (s, 3H), 2.68 (d, J = 18.0 Hz, 1H), 2.51 (dd, J_1 = 7.2 Hz, J_2 = 14.0 Hz, 1H), 2.38 (d, J = 18.0 Hz, 1H), 2.26 (dd, J_1 = 8.0 Hz, J_2 = 13.6 Hz, 1H), 1.35 (s, 3H); **^{13}C NMR ($CDCl_3$, 100 MHz)**: δ 176.2, 169.9, 131.2, 120.9, 64.7, 42.3, 41.0, 37.7, 24.1 ppm; HRMS *m/z* (ESI) calcd for $C_9H_{14}NO_3$ $(M + H)^+$, 184.0968, found 184.0968.

14) 1-Methoxy-3-methylpyrrolidine-2,5-dione (2p):

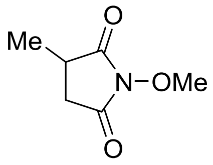

2p

The reaction of *N*-methoxy-2-methylpent-4-enamide **1p** (0.2 mmol, 28.6 mg), CuOAc (0.02 mmol, 2.5 mg) and ligand **II** (0.024 mmol, 8.7 mg) were stirred in 2 mL PhMe at 80 ℃ under oxygen for 18 h to afford 10.6 mg (37% yield) of **2p** as colorless oil: **^{1}H NMR ($CDCl_3$, 400 MHz)**: δ 3.95 (s, 3H), 2.93 (dd, J_1 = 9.2 Hz, J_2 = 17.6 Hz, 1H), 2.89–2.82 (m, 1H), 2.32 (dd, J_1 = 3.6 Hz, J_2 = 17.6 Hz, 1H), 1.38 (d, J = 7.2 Hz, 3H); **^{13}C NMR ($CDCl_3$, 100 MHz)**: δ 174.4, 170.3, 64.5, 33.8, 32.0, 16.5 ppm; HRMS *m/z* (ESI) calcd for $C_6H_{10}NO_3$ $(M + H)^+$, 144.0655, found 144.0654.

15) 3-Benzyl-1-methoxypyrrolidine-2,5-dione (2q):

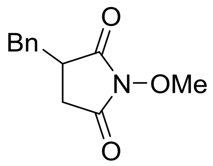

2q

The reaction of 2-benzyl-*N*-methoxypent-4-enamide **1q** (0.2 mmol, 43.9 mg), CuOAc (0.02 mmol, 2.5 mg) and ligand **II** (0.024 mmol, 8.7 mg) were stirred in 2 mL PhMe at 80 ℃ under oxygen for 18 h to afford 17.6 mg (40% yield) of **2q** as colorless oil: **^{1}H NMR ($CDCl_3$, 400 MHz)**: δ 7.33–7.24 (m, 3H), 7.17 (d, J = 7.2 Hz, 2H), 3.85 (s, 3H), 3.20–3.11 (m, 2H), 2.96 (dd, J_1 = 8.0 Hz, J_2 = 13.0 Hz, 1H), 2.69 (dd, J_1 = 8.8 Hz, J_2 = 18.4 Hz, 1H), 2.46 (d, J = 18.4 Hz, 1H); **^{13}C NMR ($CDCl_3$, 100 MHz)**: δ 173.2, 170.2, 136.1, 129.1, 128.9, 127.3, 64.6, 38.3, 36.2, 30.5 ppm; HRMS *m/z* (ESI) calcd for $C_{12}H_{14}NO_3$ $(M + H)^+$, 220.0968, found 220.0967.

16) 4-Ethyl-1-methoxy-3,3-diphenylpyrrolidine-2,5-dione (2r):

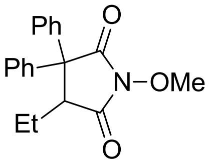

2r

The reaction of 3-ethyl-*N*-methoxy-2,2-diphenylpent-4-enamide **1r** (0.2 mmol, 61.9 mg), CuOAc (0.02 mmol, 2.5 mg) and ligand **II** (0.024 mmol, 8.7 mg) were stirred in 2 mL PhMe at 80 ℃ under oxygen for 48 h to afford 29.4 mg (48% yield) of **2r** as white solid: **^{1}H NMR ($CDCl_3$, 400 MHz)**: δ 7.48 (d, J = 7.2 Hz, 2H), 7.41–7.34 (m, 3H), 7.32–7.29 (m, 3H), 7.08–7.05 (m, 2H), 3.91 (s, 3H), 3.43 (dd, J_1 = 5.2 Hz, J_2 = 8.8 Hz, 1H), 1.39–1.27 (m, 2H), 1.02 (t, J = 7.4 Hz, 3H); **^{13}C NMR ($CDCl_3$, 100 MHz)**: δ 172.8, 172.6, 140.0, 139.2, 128.8, 128.7, 128.4, 128.2, 128.0, 127.7, 64.3, 58.9, 49.4, 23.2, 12.1 ppm; HRMS *m/z* (ESI) calcd for $C_{19}H_{20}NO_3$ $(M + H)^+$, 310.1438, found 310.1437.

17) 2-Methoxyhexahydro-1*H*-isoindole-1,3(2*H*)-dione (2s):

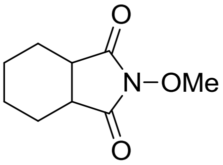

2s

The reaction of *N*-methoxy-2-vinylcyclohexanecarboxamide **1s** (0.2 mmol, 36.7 mg), CuOAc (0.02 mmol, 2.5 mg) and ligand **II** (0.024 mmol, 8.7 mg) were stirred in 2 mL PhMe at 80 ℃ under oxygen for 18 h to afford 13.6 mg (37% yield) of **2s** as colorless oil: **^{1}H NMR ($CDCl_3$, 400 MHz)**: δ 3.93 (s, 3H), 2.92–2.86 (m, 2H), 1.94–1.85 (m, 2H), 1.82–1.75 (m, 2H), 1.53–1.42 (m,

4H); **^{13}C NMR (CDCl$_3$, 100 MHz)**: δ 173.7, 64.3, 37.2, 23.4, 21.2 ppm; HRMS *m/z* (ESI) calcd for $C_9H_{14}NO_3$ (M + H)$^+$, 184.0968, found 184.0968.

18) 2-Methoxyisoindoline-1,3-dione (2t):[8]

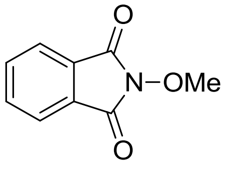

2t

The reaction of *N*-methoxy-2-vinylbenzamide **1t** (0.2 mmol, 35.4 mg), CuOAc (0.02 mmol, 2.5 mg) and ligand **II** (0.024 mmol, 8.7 mg) were stirred in 2 mL PhMe at 80 ℃ under oxygen for 18 h to afford 9.6 mg (27% yield) of **2t** as white solid: **^{1}H NMR (CDCl$_3$, 400 MHz)**: δ 7.86–7.83 (m, 2H), 7.79–7.74 (m, 2H), 4.08 (s, 3H); **^{13}C NMR (CDCl$_3$, 100 MHz)**: δ 163.2, 134.5, 128.9, 123.5, 65.8 ppm. HRMS *m/z* (ESI) calcd for $C_9H_{14}NO_3$ (M + H)$^+$, 178.0499, found 178.0495.

19) 2-(1-Methoxy-2,5-dioxopyrrolidin-3-yl)isoindoline-1,3-dione (2u):

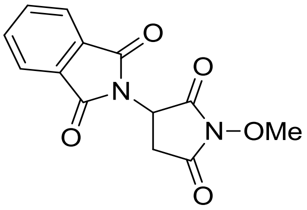

2u

The reaction of 2-(1,3-dioxoisoindolin-2-yl)-*N*-methoxypent-4-enamide **1u** (0.2 mmol, 54.9 mg), CuOAc (0.02 mmol, 2.5 mg) and ligand **II** (0.024 mmol, 8.7 mg) were stirred in 2 mL PhMe at 80 ℃ under oxygen for 18 h to afford 25.1 mg (46% yield) of **2u** as white solid: **^{1}H NMR (CDCl$_3$, 400 MHz)**: δ 7..92–7.88 (m, 2H), 7.82–7.78 (m, 2H), 5.21 (dd, J_1 = 5.2 Hz, J_2 = 9.6 Hz, 1H), 4.06 (s, 3H), 3.20 (dd, J_1 = 9.2 Hz, J_2 = 17.6 Hz, 1H), 2.94 (dd, J_1 = 5.2 Hz, J_2 = 18.0 Hz, 1H); **^{13}C NMR (CDCl$_3$, 100 MHz)**: δ 167.8, 167.5, 166.8, 134.8, 131.5, 124.0, 64.9, 44.2, 32.4 ppm; HRMS *m/z* (ESI) calcd for $C_{13}H_{211}N_2O_5$ (M + H)$^+$, 275.0668, found 275.0668.

20) 1-Methoxy-3,3-diphenylpiperidine-2,6-dione (4a):

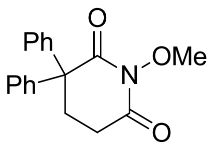

4a

The reaction of *N*-methoxy-2,2-diphenylhex-5-enamide **3a** (0.2 mmol, 59.1 mg), CuOAc (0.02

mmol, 2.5 mg) and ligand **II** (0.024 mmol, 8.7 mg) were stirred in 2 mL PhMe at 80 ℃ under oxygen for 18 h to afford 34.9 mg (59% yield) of **4a** as white solid: **^{1}H NMR ($CDCl_3$, 400 MHz)**: δ 7.37–7.30 (m, 6H), 7.16 (d, *J* = 7.2 Hz, 4H), 3.92 (s, 3H), 2.73 (t, *J* = 5.8 Hz, 2H), 2.67 (t, *J* = 5.8 Hz, 2H); **^{13}C NMR ($CDCl_3$, 100 MHz)**: δ 170.4, 167.4, 140.2, 128.7, 127.9, 127.8, 63.8, 57.4, 30.3, 29.5 ppm; HRMS *m/z* (ESI) calcd for $C_{18}H_{18}NO_3$ $(M + H)^+$, 296.1281, found 296.1279.

21) 1-Ethoxy-3,3-diphenylpiperidine-2,6-dione (4b):

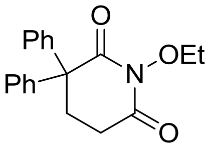

4b

The reaction of *N*-ethoxy-2,2-diphenylhex-5-enamide **3b** (0.2 mmol, 61.9 mg), CuOAc (0.02 mmol, 2.5 mg) and ligand **II** (0.024 mmol, 8.7 mg) were stirred in 2 mL PhMe at 80 ℃ under oxygen for 18 h to afford 34.1 mg (55% yield) of **4b** as white solid: **^{1}H NMR ($CDCl_3$, 400 MHz)**: δ 7.37–7.31 (m, 6H), 7.16 (d, *J* = 7.6 Hz, 4H), 4.11 (q, *J* = 7.2 Hz, 2H), 2.72 (d, *J* = 6.0 Hz, 2H), 2.66 (d, *J* = 5.6 Hz, 2H), 1.36 (t, *J* = 7.0 Hz, 3H); **^{13}C NMR ($CDCl_3$, 100 MHz)**: δ 170.8, 167.7, 140.3, 128.7, 128.0, 127.8, 72.1, 57.8, 30.3, 29.6, 13.3 ppm; HRMS *m/z* (ESI) calcd for $C_{19}H_{20}NO_3$ $(M + H)^+$, 310.1438, found 310.1435.

22) 1-Methoxy-3-methyl-3-phenylpiperidine-2,6-dione (4c):

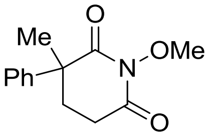

4c

The reaction of *N*-methoxy-2-methyl-2-phenylhex-5-enamide **3c** (0.2 mmol, 46.7 mg), CuOAc (0.02 mmol, 2.5 mg) and ligand **II** (0.024 mmol, 8.7 mg) were stirred in 2 mL PhMe at 80 ℃ under oxygen for 18 h to afford 22.5 mg (45% yield) of **4c** as colorless oil: **^{1}H NMR ($CDCl_3$, 400 MHz)**: δ 7.38 (t, *J* = 7.4 Hz, 2H), 7.30 (t, *J* = 7.2 Hz, 1H), 7.24 (d, *J* = 7.6 Hz, 2H), 3.92 (s, 3H), 2.71–2.65 (m, 1H), 2.51–2.35 (m, 2H), 2.18–2.10 (m, 1H), 1.61 (s, 3H); **^{13}C NMR ($CDCl_3$, 100 MHz)**: δ 171.8, 167.7, 140.3, 129.2, 127.7, 125.3, 63.8, 49.0, 30.4, 30.3, 27.5 ppm; HRMS *m/z* (ESI) calcd for $C_{13}H_{16}NO_3$ $(M + H)^+$, 234.1125, found 234.1124.

23) 3-Ethyl-1-methoxy-3-phenylpiperidine-2,6-dione (4d):

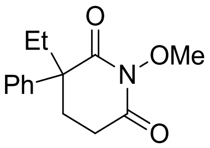

4d

The reaction of 2-ethyl-*N*-methoxy-2-phenylhex-5-enamide **3d** (0.2 mmol, 49.5 mg), CuOAc (0.02 mmol, 2.5 mg) and ligand **II** (0.024 mmol, 8.7 mg) were stirred in 2 mL PhMe at 80 ℃ under oxygen for 18 h to afford 17.8 mg (36% yield) of **4d** as colorless oil: **^{1}H NMR ($CDCl_3$, 400 MHz)**: δ 7.38 (t, J = 7.2 Hz, 2H), 7.32–7.24 (m, 3H), 3.89 (s, 3H), 2.74–2.69 (m, 1H), 2.58–2.49 (m, 1H), 2.34 (dd, J_1 = 2.0 Hz, J_2 = 14.0 Hz, 1H), 2.26–2.17 (m, 1H), 2.15–2.06 (m, 1H), 1.97–1.88 (m, 1H), 0.89 (t, J = 7.4 Hz, 3H); **^{13}C NMR ($CDCl_3$, 100 MHz)**: δ 171.1, 167.6, 138.6, 129.1, 127.7, 125.9, 63.7, 52.7, 33.6, 30.0, 25.7, 9.0 ppm; HRMS *m/z* (ESI) calcd for $C_{14}H_{18}NO_3$ (M + H)$^+$, 248.1281, found 248.1286.

24) 1-Methoxy-3-(6-methoxynaphthalen-2-yl)-3-methylpyrrolidine-2,5-dione (6):

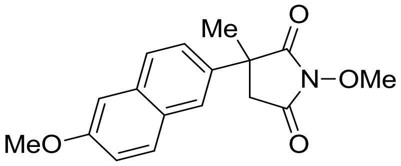

6

The reaction of 2-(4-isobutylphenyl)-*N*-methoxy-2-methylpent-4-enamide **5** (0.2 mmol, 59.9 mg), CuOAc (0.02 mmol, 2.5 mg) and ligand **II** (0.024 mmol, 8.7 mg) were stirred in 2 mL PhMe at 80 ℃ under oxygen for 18 h to afford 46.7 mg (78% yield) of **6** as white solid: **^{1}H NMR ($CDCl_3$, 400 MHz)**: δ 7.76–7.70 (m, 3H), 7.37 (dd, J_1 = 2.0 Hz, J_2 = 8.8 Hz, 1H), 7.17 (dd, J_1 = 2.4 Hz, J_2 = 8.8 Hz, 1H), 7.11 (d, J = 2.4 Hz, 1H), 4.00 (s, 3H), 3.91 (s, 3H), 3.18 (d, J = 18.0 Hz, 1H), 2.88 (d, J = 18.0 Hz, 1H), 1.81 (s, 3H); **^{13}C NMR ($CDCl_3$, 100 MHz)**: δ 175.1, 169.6, 158.1, 135.7, 133.6, 129.6, 128.5, 128.0, 124.1, 123.7, 119.5, 105.4, , 55.3, 45.2, 42.6, 25.6 ppm; HRMS *m/z* (ESI) calcd for $C_{17}H_{18}NO_4$ (M + H)$^+$, 300.1230, found 300.1221.

25) 3-(4-Isobutylphenyl)-1-methoxy-3-methylpyrrolidine-2,5-dione (8):

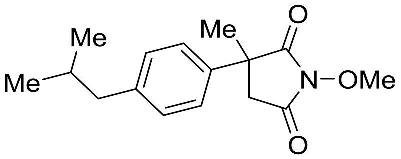

8

The reaction of *N*-methoxy-2-(6-methoxynaphthalen-2-yl)-2-methylpent-4-enamide **7** (0.2 mmol, 55.1 mg), CuOAc (0.02 mmol, 2.5 mg) and ligand **II** (0.024 mmol, 8.7 mg) were stirred in 2 mL PhMe at 80 ℃ under oxygen for 18 h to afford 32.9 mg (60% yield) of **8** as colorless oil: **^{1}H NMR ($CDCl_3$, 400 MHz)**: δ 7.27–7.24 (m, 2H), 7.17–7.14 (m, 2H), 3.98 (s, 3H), 3.09 (d, *J* = 18.0 Hz, 1H), 2.82 (d, *J* = 18.0 Hz, 1H), 2.46 (d, *J* = 7.2 Hz, 2H), 1.89–1.79 (m, 1H), 1.73 (s, 3H), 0.89 (d, J = 6.8 Hz, 6H); **^{13}C NMR ($CDCl_3$, 100 MHz)**: δ 175.2, 169.7, 141.4, 138.2, 129.8, 125.2, 64.7, 45.0, 44.8, 42.8, 30.1, 25.7, 22.3 ppm; HRMS *m/z* (ESI) calcd for $C_{16}H_{22}NO_3$ $(M + H)^+$, 276.1594, found 276.1591.

26) 3-(2-Fluoro-[1,1'-biphenyl]-4-yl)-1-methoxy-3-methylpyrrolidine-2,5-dione (10):

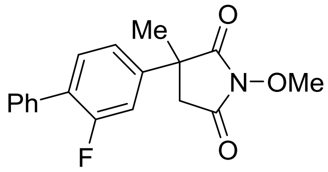

10

The reaction of 2-(2-fluoro-[1,1'-biphenyl]-4-yl)-*N*-methoxy-2-methylpent-4-enamide **9** (0.2 mmol, 62.6 mg), CuOAc (0.02 mmol, 2.5 mg) and ligand **II** (0.024 mmol, 8.7 mg) were stirred in 2 mL PhMe at 80 ℃ under oxygen for 18 h to afford 46.9 mg (75% yield) of **10** as white solid: **^{1}H NMR ($CDCl_3$, 400 MHz)**: δ 7.53–7.51 (m, 2H), 7.48–7.36 (m, 4H), 7.24–7.19 (m, 2H), 4.00 (s, 3H), 3.12 (d, *J* = 18.0 Hz, 1H), 2.87 (d, *J* = 18.0 Hz, 1H), 1.76 (s, 3H); **^{13}C NMR ($CDCl_3$, 100 MHz)**: δ 174.4, 169.1, 159.7 (d, J_{CF} = 247.9), 142.1 (d, J_{CF} = 7.5 Hz), 134.8, 131.3 (d, J_{CF} = 4.0 Hz), 128.8 (d, J_{CF} = 2.9 Hz), 128.6, 128.5, 128.0, 121.4 (d, J_{CF} = 3.5 Hz), 113.8 (d, J_{CF} = 24.7 Hz), 64.8, 44.9, 42.3, 25.8 ppm; **^{19}F NMR ($CDCl_3$, 375 MHz)**: δ -115.8 (s, 1F) ppm; HRMS *m/z* (ESI) calcd for $C_{18}H_{17}FNO_3$ $(M + H)^+$, 314.1187, found 314.1187.

E. The crystal structure of compound 6 (CCDC: 1912652).

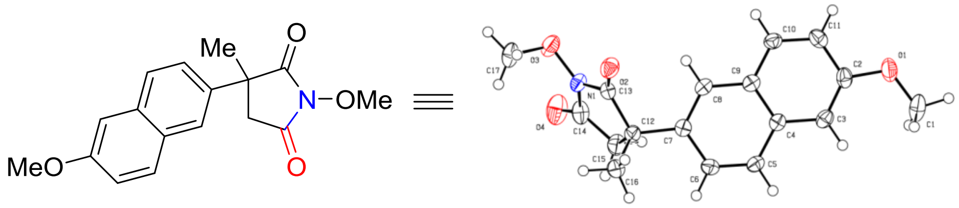

F. Synthesis of succinimide-containing bioactive compounds.

1) The synthesis of the ethosuximide 13:

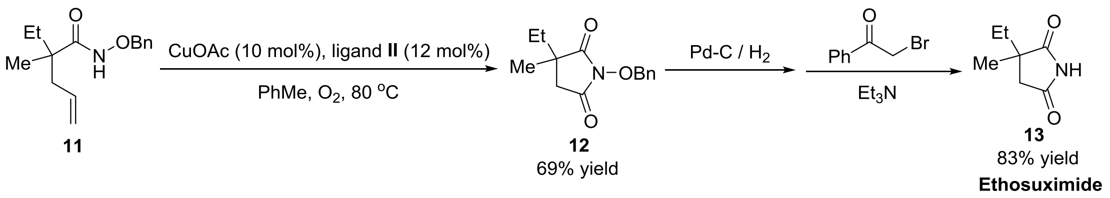

The reaction of *N*-(benzyloxy)-2-ethyl-2-methylpent-4-enamide **11** (0.8 mmol, 197.8 mg), CuOAc (0.08 mmol, 10.0 mg) and ligand **II** (0.096 mmol, 34.8 mg) were stirred in 4 mL PhMe at 80 ℃ under oxygen for 18 h. Then the mixture was concentrated and purified by silica gel column chromatography (with ethyl acetate/petroleum ether 1:4 as the eluent) to afford the desired products **12** as white solid (135.9 mg, 69% yield): **^{1}H NMR ($CDCl_3$, 400 MHz)**: δ 7.49–7.46 (m, 2H), 7.38–7.36 (m, 3H), 5.14 (s, 2H), 2.50 (d, *J* = 18.0 Hz, 1H), 2.29 (d, *J* = 18.0 Hz, 1H), 1.69–1.59 (m, 1H), 1.54–1.45 (m, 1H), 1.19 (s, 3H), 0.78 (t, *J* = 7.6 Hz, 3H); **^{13}C NMR ($CDCl_3$, 100 MHz)**: δ 176.9, 170.4, 133.2, 130.1, 129.4, 128.4, 78.3, 41.5, 37.9, 30.9, 23.9, 8.3 ppm; HRMS *m/z* (ESI) calcd for $C_{14}H_{18}NO_3$ $(M + H)^+$, 248.1281, found 248.1279.

Then, a catalytic amount of 5% Pd/C (27.2 mg, 20% w/w) was added to a solution of **12** (135.9 mg, 0.55 mmol, 1.00 equiv) in dry methanol (4 mL), and the mixture was stirred under H_2 for 1.5 h. The reaction mixture was filtered through a pad of Celite, washed with methanol, and concentrated. The residue was dissolved in acetonitrile (2 mL) and added dropwise to a stirred solution of 2-bromoacetophenone (109.6 mg, 0.55 mmol, 1.00 equiv) in acetonitrile (3 mL) at room temperature. A solution of triethylamine (115.1 μL, 83.6 mg, 0.825 mmol, 1.50 equiv) in acetonitrile (1 mL) was added dropwise over 2 h, and the reaction mixture was stirred at room temperature overnight.[9] The mixture was concentrated and then partitioned between CH_2Cl_2 (12.5 mL) and 5% hydrochloric acid (2 × 10 mL). The organic layer was washed with brine (10 mL), dried over $MgSO_4$, filtered, concentrated, and purified by flash chromatography on silica gel (with ethyl acetate/petroleum ether 1:3 as the eluent) to afford ethosuximide **13** as a white solid (64.2 mg, 83% yield). **^{1}H NMR ($CDCl_3$, 400 MHz)**: δ 8.78 (s, 1H), 2.67 (d, *J* = 18.4 Hz, 1H), 2.47 (d, *J* = 18.4 Hz, 1H), 1.80–1.71 (m, 1H), 1.65–1.56 (m, 1H), 1.32 (s, 3H), 0.93 (t, *J* = 7.4 Hz, 3H); **^{13}C NMR ($CDCl_3$, 100 MHz)**: δ 183.5, 176.6, 45.6, 41.5, 30.9, 23.7, 8.6 ppm.[10] MS (70 ev): m/z (%): 55.0 (100), 70.1 (50), 85.0 (12), 113.0 (84), 141.0 (M^+, 1).

2) The synthesis of the methsuximide 17:

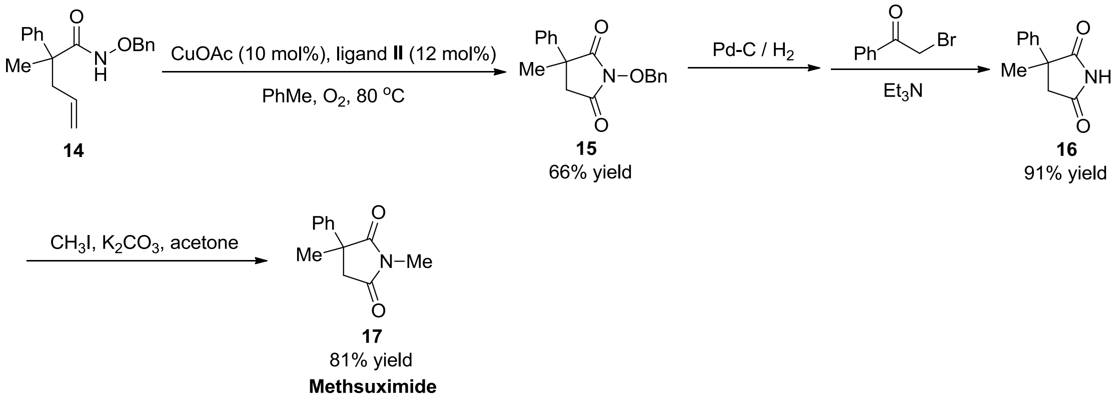

The reaction of *N*-(Benzyloxy)-2-methyl-2-phenylpent-4-enamide **14** (0.6 mmol, 177.2 mg), CuOAc (0.06 mmol, 7.5 mg) and ligand **II** (0.072 mmol, 8.7 mg) were stirred in 2 mL PhMe at 80 ℃ under oxygen for 18 h to afford 116.3 mg (66% yield) of **15** as yellow oil: **^{1}H NMR ($CDCl_3$, 400 MHz)**: δ 7.49–7.47 (m, 2H), 7.39–7.32 (m, 3H), 7.31–7.24 (m, 3H), 7.15–7.12 (m, 2H), 5.19 (s, 2H), 2.96 (d, *J* = 18.0 Hz, 1H), 2.72 (d, *J* = 18.0 Hz, 1H), 1.61 (s, 3H); **^{13}C NMR ($CDCl_3$, 100 MHz)**: δ 175.2, 169.8, 141.0, 133.1, 130.1, 129.5, 128.9, 128.5, 127.6, 125.4, 78.3, 45.1, 42.7, 25.4 ppm; HRMS *m/z* (ESI) calcd for $C_{18}H_{18}NO_3$ $(M + H)^+$, 296.1281, found 296.1283.

Then, a catalytic amount of 5% Pd/C (23.2 mg, 20% w/w) was added to a solution of **15** (116 mg, 0.39 mmol, 1.00 equiv) in dry methanol (4 mL), and the mixture was stirred under H_2 for 1.5 h. The reaction mixture was filtered through a pad of Celite, washed with methanol, and concentrated. The residue was dissolved in acetonitrile (2 mL) and added dropwise to a stirred solution of 2-bromoacetophenone (77.6 mg, 0.39 mmol, 1.00 equiv) in acetonitrile (3 mL) at room temperature. A solution of triethylamine (81.6 μL, 59.3 mg, 0.585 mmol, 1.50 equiv) in acetonitrile (1 mL) was added dropwise over 2 h, and the reaction mixture was stirred at room temperature overnight.[8] The mixture was concentrated and then partitioned between CH_2Cl_2 (10 mL) and 5% hydrochloric acid (2 × 7.5 mL). The organic layer was washed with brine (7.5 mL), dried over $MgSO_4$, filtered, concentrated, and purified by flash chromatography on silica gel (with ethyl acetate/petroleum ether 1:2 as the eluent) to afford **16** as a white solid (66.8 mg, 91% yield). **^{1}H NMR ($CDCl_3$, 400 MHz)**: δ 9.47 (s, 1H), 7.38–7.33 (m, 4H), 7.29–7.25 (m, 1H), 3.12 (d, *J* = 18.4 Hz, 1H), 2.87 (d, *J* = 18.4 Hz, 1H), 1.72 (s, 3H); **^{13}C NMR ($CDCl_3$, 100 MHz)**: δ 182.0, 176.5, 141.2, 128.8, 127.5, 125.5, 49.1, 46.0, 25.3 ppm.

A mixture of succinimide **16 (**65.5 mg, 0.346 mmol), methyl iodide (56.8 mg, 0.4 mmol) and anhydrous potassium cabonate (62.2 g, 0.45 mmol) was heated at reflux in anhydrous acetone 4

mL for 4 h. After cooling to 20 ^oC, the mixture was concentrated and purified by silica gel column chromatography (with ethyl acetate/petroleum ether 1:3) to afford methsuximide **17** as a white solid (56.7 mg, 81% yield). **^{1}H NMR ($CDCl_3$, 400 MHz)**: δ 7.38–7.33 (m, 4H), 7.31–7.25 (m, 1H), 3.11 (d, J = 18.0 Hz, 1H), 3.06 (s, 3H), 2.85 (d, J = 18.0 Hz, 1H), 1.71 (s, 3H); **^{13}C NMR ($CDCl_3$, 100 MHz)**: δ 180.9, 175.5, 141.7, 128.8, 127.4, 125.5, 47.8, 45.1, 25.6, 25.1 ppm.[11] MS (70 ev): m/z (%): 51.1 (17), 77.1 (40), 91.1 (30), 103.1 (58), 118.1 (100), 203.1 (M^+, 100).

G. Mechanistic investigation

1) The radical trapping experiment:

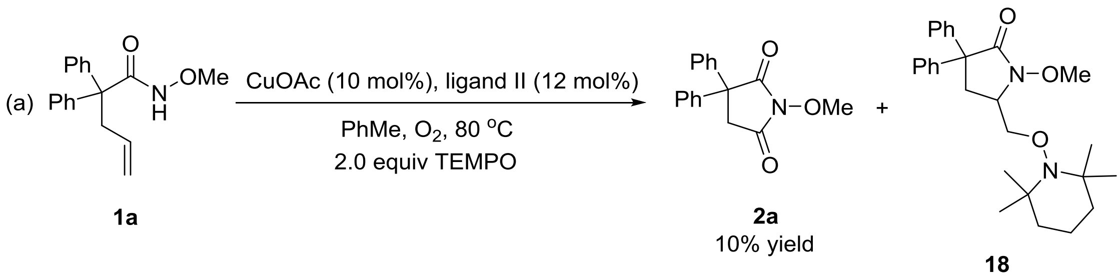

To a reaction tube charged with *N*-methoxy-2,2-diphenylpent-4-enamide **1a** (56.3 mg, 0.2 mmol) was added CuOAc (2.5 mg, 0.02 mmol), ligand **II** (0.024 mmol, 8.7 mg) and TEMPO (62.5 mg, 0.4 mmol). The resulting mixure was stirred in 2 mL PhMe at 80 ℃ under oxygen for 18 h. After cooling down to room temperature, the filtrate was concentrated in vacuo, and the crude mixture was subjected to flash column chromatography to afford the C=C bond cleavage product in only 10% yield (5.6 mg), and radical trapping product **18** in 87% yield (76.0 mg) as a white solid: **^{1}H NMR ($CDCl_3$, 400 MHz)**: δ 7.40–7.20 (m, 10H), 4.02–3.94 (m, 2H), 3.91–3.86 (m, 1H), 3.84 (s, 3H), 2.93 (dd, J_1 =6.2 Hz, J_2 = 12.9 Hz, 1H), 2.63 (dd, J_1 = 8.8 Hz, J_2 = 13.0 Hz, 1H), 1.60–1.43 (m, 5H), 1.33–1.30 (m, 1H), 1.17 (s, 3H), 1.15 (s, 3H), 1.11 (s, 3H), 1.08 (s, 3H); **^{13}C NMR ($CDCl_3$, 100 MHz)**: δ 171.7, 143.8, 141.3, 128.5, 128.3, 127.8, 127.7, 127.2, 126.8, 76.0, 62.3, 60.0, 54.3, 53.6, 39.7, 36.3, 33.0, 32.9, 20.1, 16.9 ppm; HRMS *m/z* (ESI) calcd for $C_{27}H_{37}N_2O_3$ $(M + H)^+$, 437.2799, found 437.2793.

2) The intermediate product isolation and further reaction.

In order to probe the mechanism, further control experiments were investigated. When the olefin ortho-substituted **1r** was used as the substrate and reaction for 18 hours, both the C=C

double-bond cleavage product (**2r**, 30% yield) and aldehyde product (**19**, 54% yield) could be detected, and the aldehyde product was separated. Next, we investigated the possibility that aldehyde **19** as an intermediates in the formation of succinimide **2r**. Submitting **19** to the standard reaction condition resulted in the formation of **2r**, which means the aldehyde might be the key intermediate in this transformation.

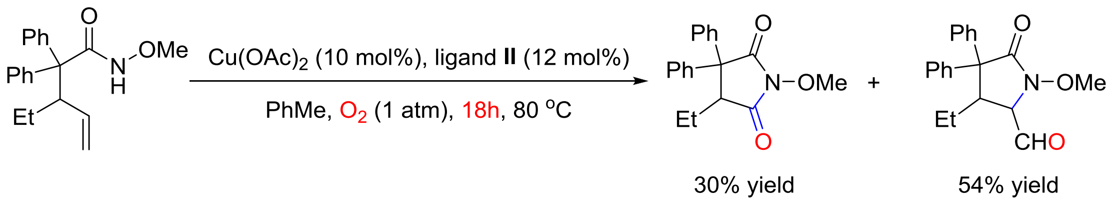

1r **2r** **19**

The reaction of 3-ethyl-*N*-methoxy-2,2-diphenylpent-4-enamide **2r** (0.2 mmol, 61.9 mg), CuOAc (0.02 mmol, 2.5 mg) and ligand **II** (0.024 mmol, 8.7 mg) were stirred in 2 mL PhMe at 80 ℃ under oxygen for 18 h. After cooling down to room temperature, the filtrate was concentrated in vacuo, and the crude mixture was subjected to flash column chromatography to afford the C=C bond cleavage product **2r** in 30% yield (18.6 mg), and aldehyde product 3-ethyl-1-methoxy-5-oxo-4,4-diphenylpyrrolidine-2-carbaldehyde **19** in 54% yield (33.4 mg, dr > 25:1) as colorless oil: **^{1}H NMR ($CDCl_3$, 400 MHz)**: δ 9.68 (d, J = 4.0 Hz, 1H), 7.57 (d, J = 7.6 Hz, 2H), 7.42–7.32 (m, 3H), 7.28–7.24 (m, 3H), 6.89–6.87 (m, 2H), 4.00 (dd, J_1 = 4.0 Hz, J_2 = 8.0 Hz, 1H), 3.95 (s, 3H), 3.15–3.11 (m, 1H), 1.49–1.40 (m, 1H), 1.01–0.92 (m, 1H), 0.89 (t, J = 6.8 Hz, 3H); **^{13}C NMR ($CDCl_3$, 100 MHz)**: δ 198.3, 174.1, 140.6, 139.8, 128.6, 128.4, 128.2, 128.1, 127.7, 127.3, 69.3, 63.9, 57.9, 42.1, 23.3, 12.5 ppm; HRMS *m/z* (ESI) calcd for $C_{20}H_{22}NO_3$ $(M + H)^+$, 324.1600, found 324.1596.

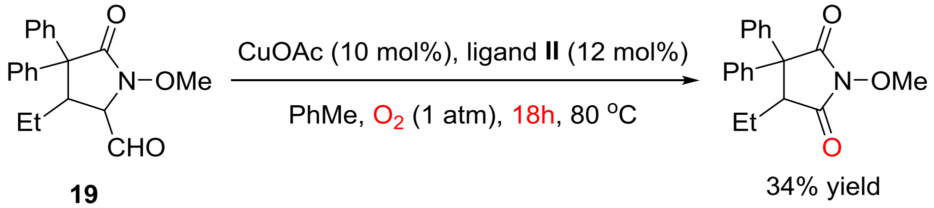

19 **2r**

The reaction of 3-ethyl-1-methoxy-5-oxo-4,4-diphenylpyrrolidine-2-carbaldehyde **19** (0.107 mmol, 34.7 mg), CuOAc (0.02 mmol, 2.5 mg) and ligand **II** (0.024 mmol, 8.7 mg) were stirred in 2 mL PhMe at 80 ℃ under oxygen for 18 h. After cooling down to room temperature, the mixture was concentrated and purified by silica gel column chromatography (with ethyl acetate/petroleum

ether as the eluent) to afford the desired product **2r** (11.3 mg, 34% yield).

3) [^{18}O]-Labeling experiments

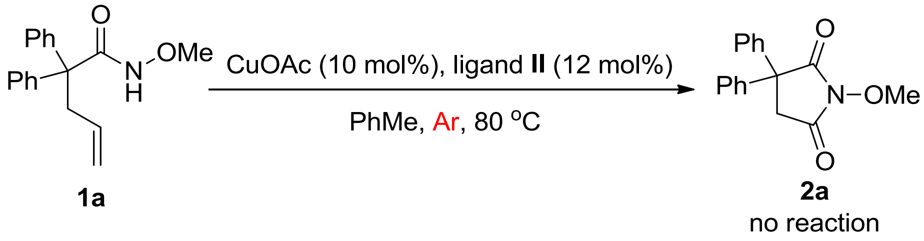

Reaction conditions: *N*-methoxy-2,2-diphenylpent-4-enamide **1a** (28.1 mg, 0.1 mmol), CuOAc (0.01 mmol, 1.3 mg), ligand **II** (0.012 mmol, 4.3 mg) and 2 mL PhMe were added to a 20 mL reaction tube with a stir bar. Then the reaction tube was placed into the liquid nitrogen and vacuum pumping. Next, the reaction mixture was warmed to the room temperature and filled with argon. This operation was repeated three times, then the reaction mixture was placed into the heating device which was 80 ℃ and reaction for 18 hours. After cooling down to room temperature, the reaction was monitored by TLC analysis, and the target product was not detected.

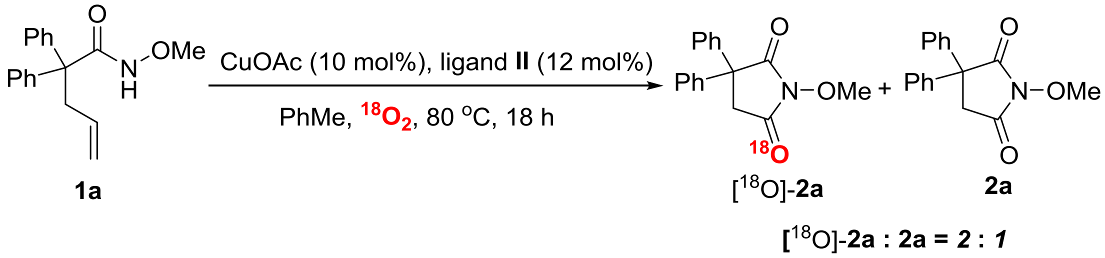

Reaction conditions: *N*-methoxy-2,2-diphenylpent-4-enamide **1a** (28.1 mg, 0.1 mmol), CuOAc (0.01 mmol, 1.3 mg) and ligand **II** (0.012 mmol, 4.3 mg) were stirred in 1 mL PhMe at 80 ℃ under $^{18}O_2$ for 18 h. After cooling down to room temperature, the mixture was concentrated and purified by silica gel column chromatography (with ethyl acetate/petroleum ether = 5:1 as the eluent) to afford the desired product [^{18}O-**2a**] (22.5 mg, 80% yield). The ^{18}O in product **2a** was determined by HRMS. Two products were detected on the base of HRMS analysis (see below): [^{18}O]-**2a** and **2a**, with the ratio 2:1. [^{18}O]-**2a**: HRMS *m/z* (ESI) calcd for $C_{17}H_{16}NO_2{}^{18}O$ (M + H)$^+$, 284.1167, found 284.1167. **2a**: HRMS *m/z* (ESI) calcd for $C_{17}H_{16}NO_3$ (M + H)$^+$, 282.1125, found 282.1122.

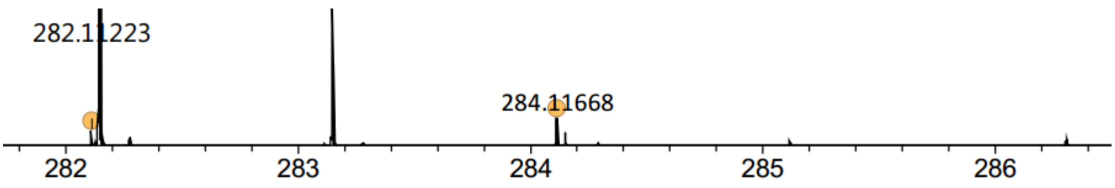

When 5.0 equiv of $H_2{}^{18}O$ was added into the reaction of **2a** under the standard conditions, the two products could also be detected with the ratio 1:11 on the base of GC-MS analysis (see below).

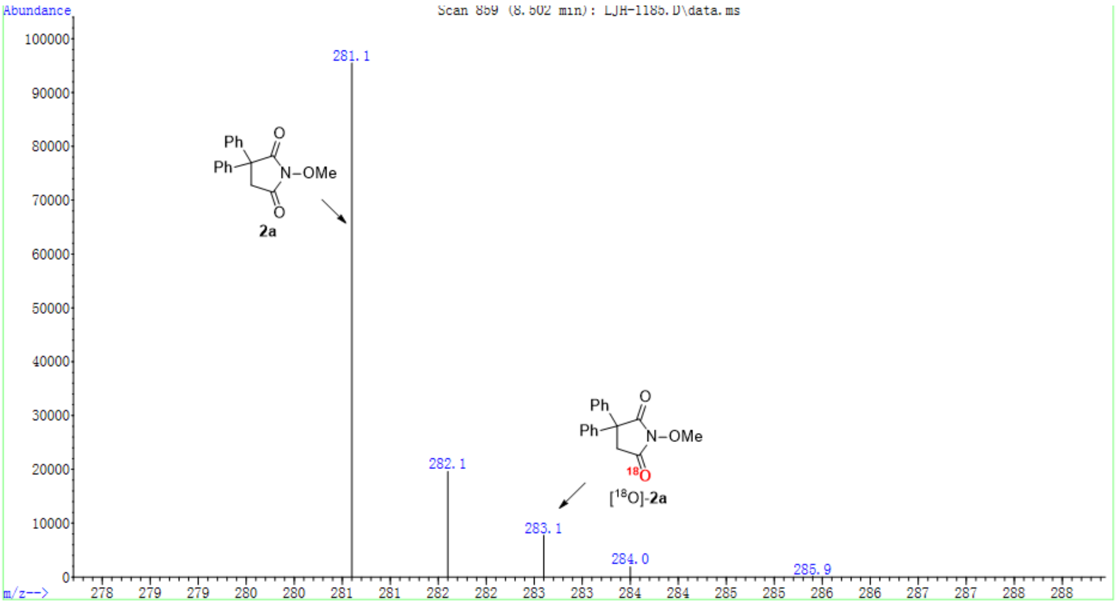

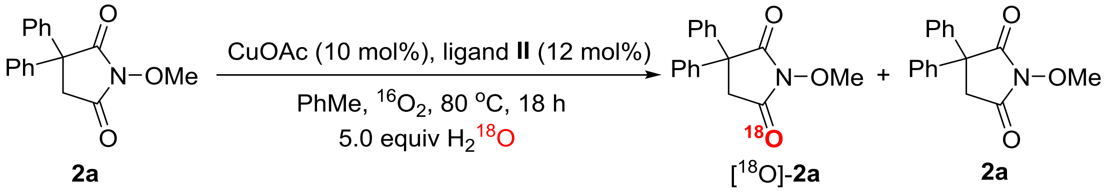

[^{18}O]-2a : 2a = *1* : *11*

Reaction conditions: **2a** (28.1 mg, 0.1 mmol), CuOAc (0.01 mmol, 1.3 mg, 10 mol%), ligand **II** (0.012 mmol, 4.3 mg, 12 mol%) and $H_2{}^{18}O$ (10 mg, 5.0 equiv) were stirred in 1 mL PhMe at 80 ℃ under O_2 for 18 h. After cooling down to room temperature, the mixture was concentrated and purified by silica gel column chromatography (with ethyl acetate/petroleum ether = 5:1 as the eluent) to afford the desired product. The [^{18}O]-**2a** was determined by GC-MS. The two products were detected on the base of GC-MS analysis: [^{18}O]-**2a** and **2a** with the ratio 1:11.

The above ^{18}O-labeling experiments suggest that the additional oxygen atom in imide product is original form O_2. The O-isotope exchange products are probably generated from exchange with $H_2{}^{18}O$, which was the byproduct in PhMe.

4) Kinetic studies:

Prepare nine reactions which was numbered as one to nine, to each of the reaction, the *N*-methoxy-2,2-diphenylpent-4-enamide **1a** (0.2 mmol, 56.3 mg), CuOAc (0.02 mmol, 2.5 mg) and ligand **II** (0.024 mmol, 8.7 mg) were stirred in 2 mL PhMe at 80 ℃ under oxygen. At time of 5 min, 10 min, 30 min, 1 h, 2 h, 4 h, 8 h, 14 h and 18 h, the reaction of 1 to 9 was stopped in turn, and filtration through quartz sand, then the NMR yield of each compound was determined by using 1,1,2,2-tetrachloroethane as internal standard (**Table S10**). The graph below represents the changes of relative concentration of analyses as a function of time (**Fig. S2**).

Table S10. Kinetic data and profile of the reaction.

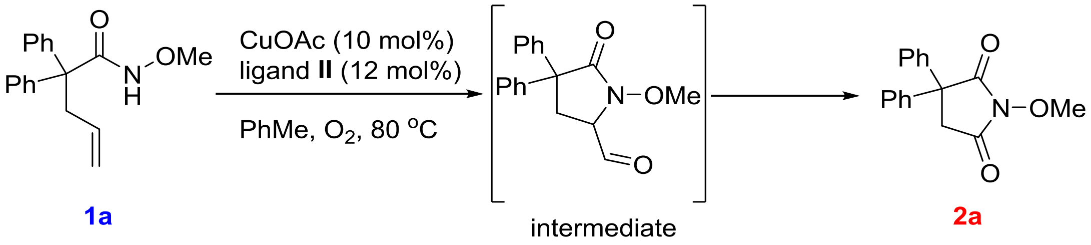

Entry	Time	**1a** (yield^b)	**2a** (yield^b)	Intermediate product (yield^b)
**1**	5 min	68%	Trace (3%)	15%
**2**	10 min	55%	Trace (2%)	27%
**3**	30 min	nd	18%	34%
**4**	1 h	nd	37%	30%
**5**	2h	nd	51%	25%
**6**	4h	nd	72%	10%
**7**	8h	nd	76%	8%
**8**	14h	nd	77%	6%
**9**	18h	nd	81%	2%

[a] Unless noted otherwise, reaction conditions: **1a** (0.20 mmol), CuOAc (0.02 mmol), ligand **II** (0.024 mmol), in PhMe (2.0 mL) was stirred at 80 °C under O_2. [b] Determined by ^{1}H NMR analysis using 1,1,2,2-tetrachloroethane as internal standard.

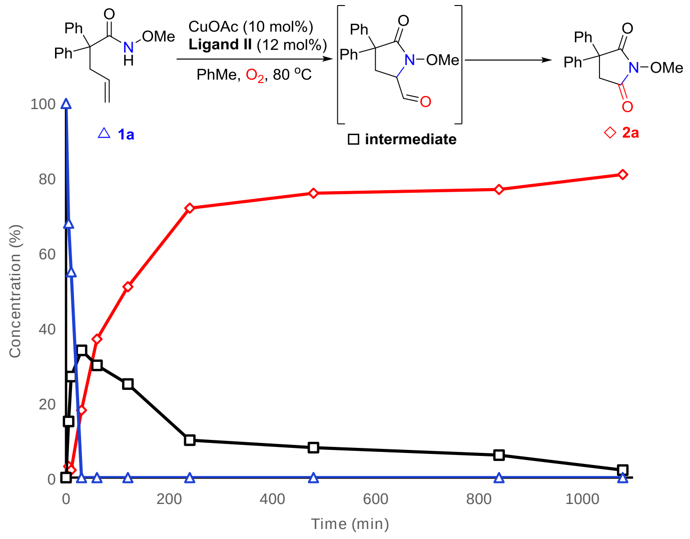

Figure S2. Kinetic profile of the reaction.

5) EPR spectra:

To get more information about the mechanism of this transformation, we also tried to catch some intermediates by EPR. EPR spectra were recorded at room temperature on a Bruker ESP-300E: Receiver Gain=1.00e+005; Phase=0deg; Harmonic=1; Mod. Frequency=100.000 KHz; Mod. Amplitude=1.944G; Canter Field=3485.000G; Sweep width 100.000G; Resolution=1024 points; Conversion=40.960ms; Time const=20.480m; Sweep time=41.943s; Power=1.00e+001 mw. DMPO (5-,5-dimethyl-1-pyrroline N-oxide) was employed as the radical trap.

When the reaction was carried out under the standard conditions (**1a**, CuOAc, ligand **II**, O_2 and DMPO) was recorded for the EPR spectra, the signals corresponding to (DMPO–O(H)) can beobviously identified, which are classical four peaks (**Fig. S3**). The hydroxyl radical maybe derived from a superoxide compound according to our previous report.[12]

When the reaction was carried out without ligand **II** (bathocuproine), complex signals of organic radical were detected (**Fig. S4**). Which indicate that a series of side effects were occurred in the reaction, and only 13% yield of the desired product could be obtained under this condition.

Furthermore, Cu^{II} complex signal were also detected by EPR in the reaction under standard conditions (**Fig. S5**). Meanwhile, the signal of nitrogen radicals was not detected, which demonstrate that this reaction was not going through the process of nitrogen radicals, but a kind of alkyl−metal intermediates.

In addition, there were no radical signals detected when the reaction was carried out under Ar (1 atm) (**Fig. S6**), which proves that molecular oxygen is very important for the initiate of this radical process.

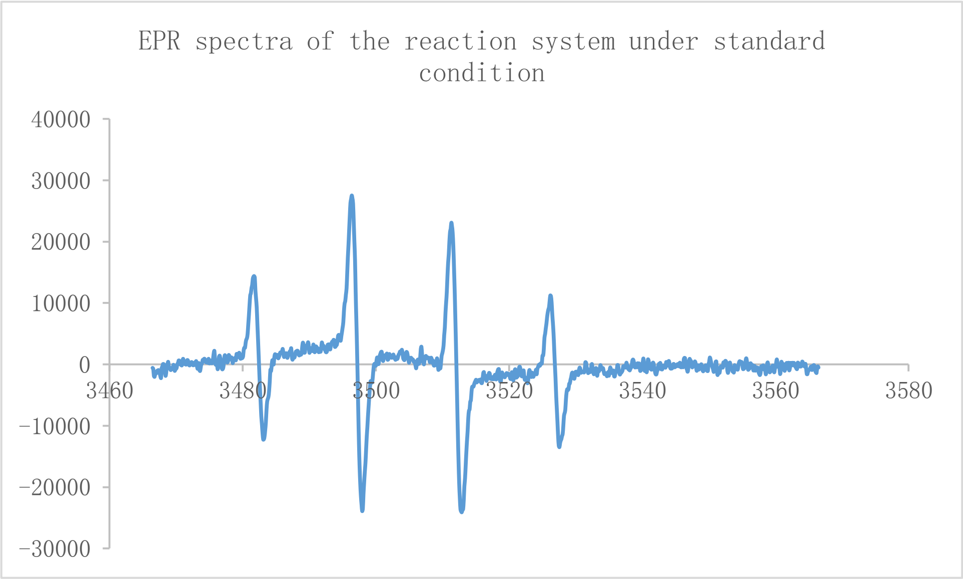

Figure S3. EPR spectra of reaction system: **1a** (0.2 mmol), CuOAc (0.02 mmol), ligand **II** (0.024 mmol) in toluene (2.0 mL), stirred at 80 ℃ under O_2 (1 atm), 1.5 h. 0.01 mL of this reaction solution was taken out into a small tube, followed by the addition of 0.01 mL DMPO ($5*10^{-2}$ M). Then, this mixture was analyzed by EPR. There are classical 4 peaks the signals corresponding to (DMPO–O(H)).

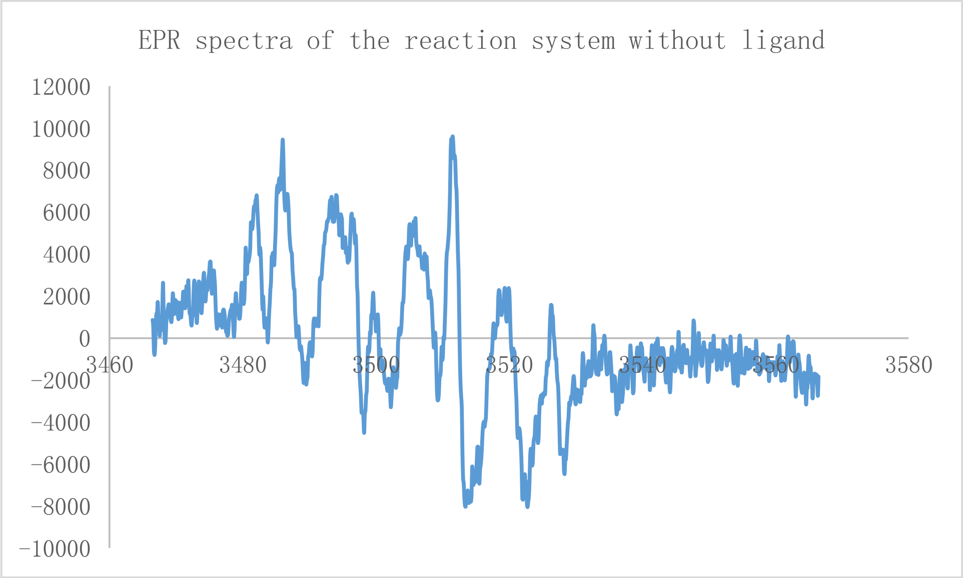

Figure S4. EPR spectra of reaction system: **1a** (0.2 mmol), CuOAc (0.02 mmol) in toluene (2.0 mL), stirred at 80 ℃ under O_2 (1 atm), 0.25 h. 0.01 mL of this reaction solution was taken out into a small tube, followed by the addition of 0.01 mL DMPO ($5*10^{-2}$ M). Then, this mixture was analyzed by EPR.

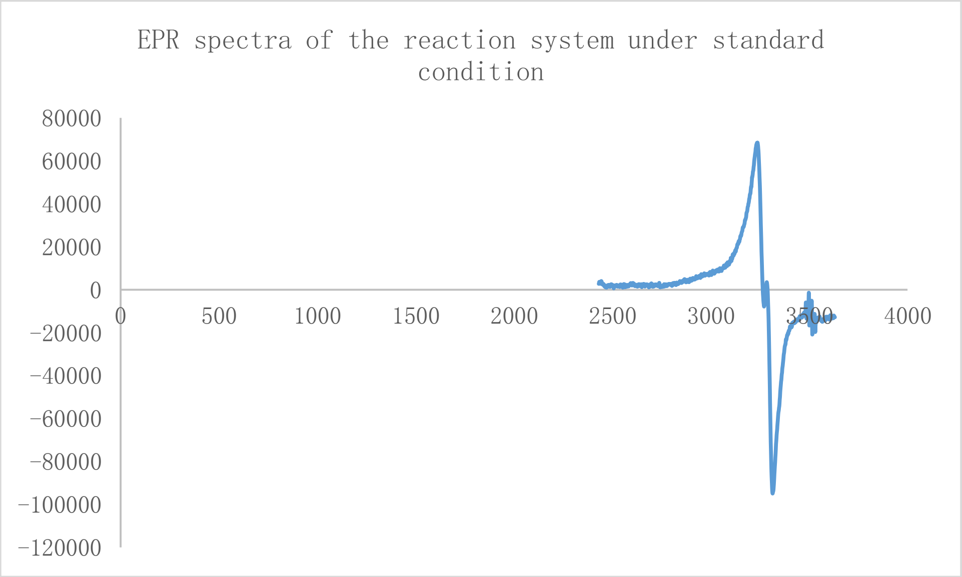

Figure S5. EPR spectra of reaction system: **1a** (0.2 mmol), CuOAc (0.02 mmol), ligand **II** (0.024 mmol) in toluene (2.0 mL), stirred at 80 ℃ under O_2 (1 atm), 5 min. 0.01 mL of this reaction solution was taken out into a small tube, followed by the addition of 0.01 mL DMPO ($5*10^{-2}$ M). Then, this mixture was analyzed by EPR.

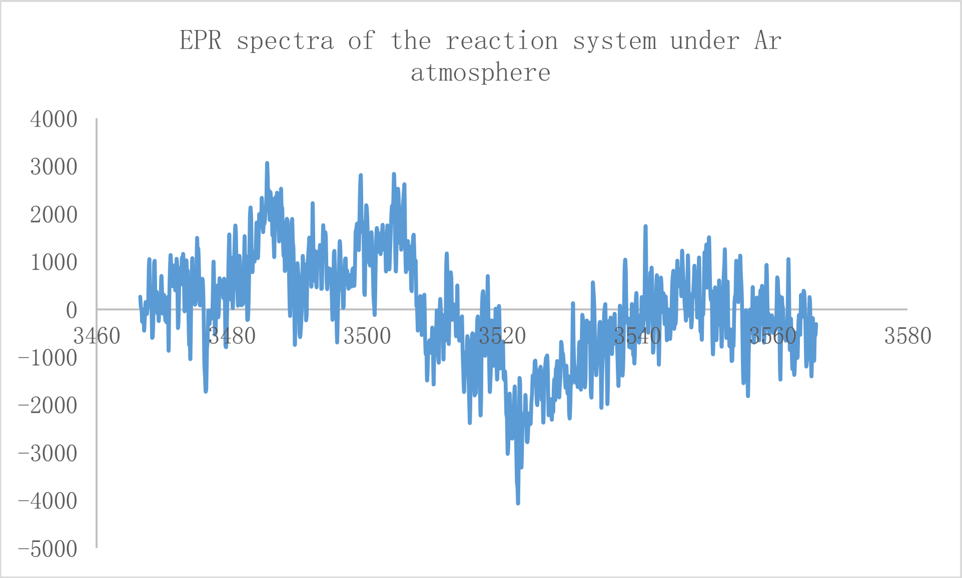

Figure S6. EPR spectra of reaction system: **1a** (0.2 mmol), CuOAc (0.02 mmol), ligand **II** (0.024 mmol) in toluene (2.0 mL), stirred at 80 ℃ under Ar (1 atm), 0.5 h. 0.01 mL of this reaction solution was taken out into a small tube, followed by the addition of 0.01 mL DMPO ($5*10^{-2}$ M). Then, this mixture was analyzed by EPR.

H. References:

1) N. T. Barczak and E. R. Jarvo, *Chem. Eur. J.*, 2011, **17**, 12912.
2) Y.-B. Kang, X.-M. Chen, C.-Z. Yao and X,-S. Ning, *Chem. Commun.*, 2016, **52**, 6193.
3) K. Shen and Q. Wang, *Chem. Sci.*, 2015, **6**, 4279.
4) K.-Y. Lo, L. Ye and D. Yang, *Synlett*, 2017, **28**, 1570.
5) K. Shen and Q. Wang, *Org. Chem. Front.*, 2016, **3**, 222.

6) S. Nicolai, C. Piemontesi and J. A Waser, *Angew. Chem. Int. Ed.*, 2011, **50**, 4680.
7) K. Shen and Q. Wang, *J. Am. Chem. Soc.*, 2017, **139**, 13110.
8) J. W. Wrigglesworth, B. Cox and G. C. Lloyd-Jones, *Org. Lett.*, 2011, **13**, 5326.
9) (a) M. A. Silvers, G. T. Robertson, C. M. Taylor and G. L. Waldrop, *J. Med. Chem.*, 2014, **57**, 8947; (b) J. Pohlmann, T. Lampe, M. Shimada, P. G. Nell, J. Pernerstorfer, N. Svenstrup, N. A. Brunner, G. Schiffer and C. Freiberg, *Bioorg. Med. Chem. Lett.*, 2015, **15**, 1189; (c) D. J. Dixon and S. G. Davies, *Chem. Commun.*, 1996, 1797; (d) K. V. Nikitin and N. P. Andryukhova, *Mendeleev Commun*., 2000, **10**, 32.
10) (a) K, Nishide, T. Katoh, H. Imazato and M. Node, *Heterocycles*, 1998, **47**, 839; (b) T. Katoh, K, Nishide, M. Node and H. Ogura, *Heterocycles*, 1999, **50**, 833.
11) M. J. Kornet and A. M. Crider, *J. Med. Chem.*, 1977, **20**, 1210.
12) (a) C. Zhang, L. Zhang and N. Jiao, *Adv. Synth. Catal.*, 2012, **354**, 1293; (b) C. Zhang, Z. Xu, T. Shen, G. Wu, L. Zhang and N. Jiao, *Org. Lett.*, 2012, **14**, 2362; (c) C. Zhang, P. Feng and N. Jiao, *J. Am. Chem. Soc.*, 2013, **135**, 15257.

I. ^{1}H NMR and ^{13}C NMR spectra

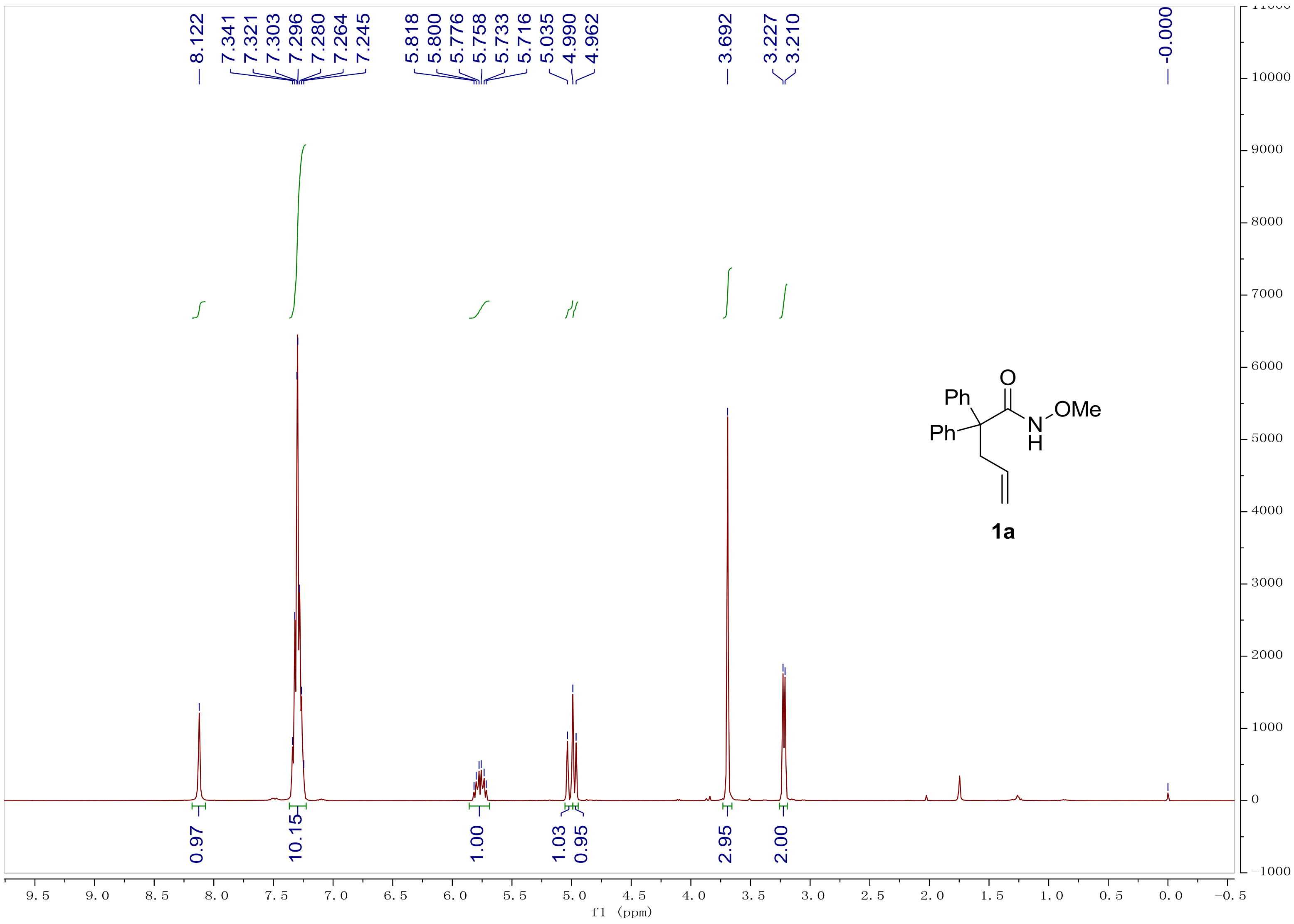

8.122
7.341
7.321
7.303
7.296
7.280
7.264
7.245
5.818
5.800
5.776
5.758
5.733
5.716
5.035
4.990
4.962
3.692
3.227
3.210
-0.000
0.97
10.15
1.00
1.03
0.95
2.95
2.00
Ph
Ph
O
N
OMe
H
1a
f1 (ppm)

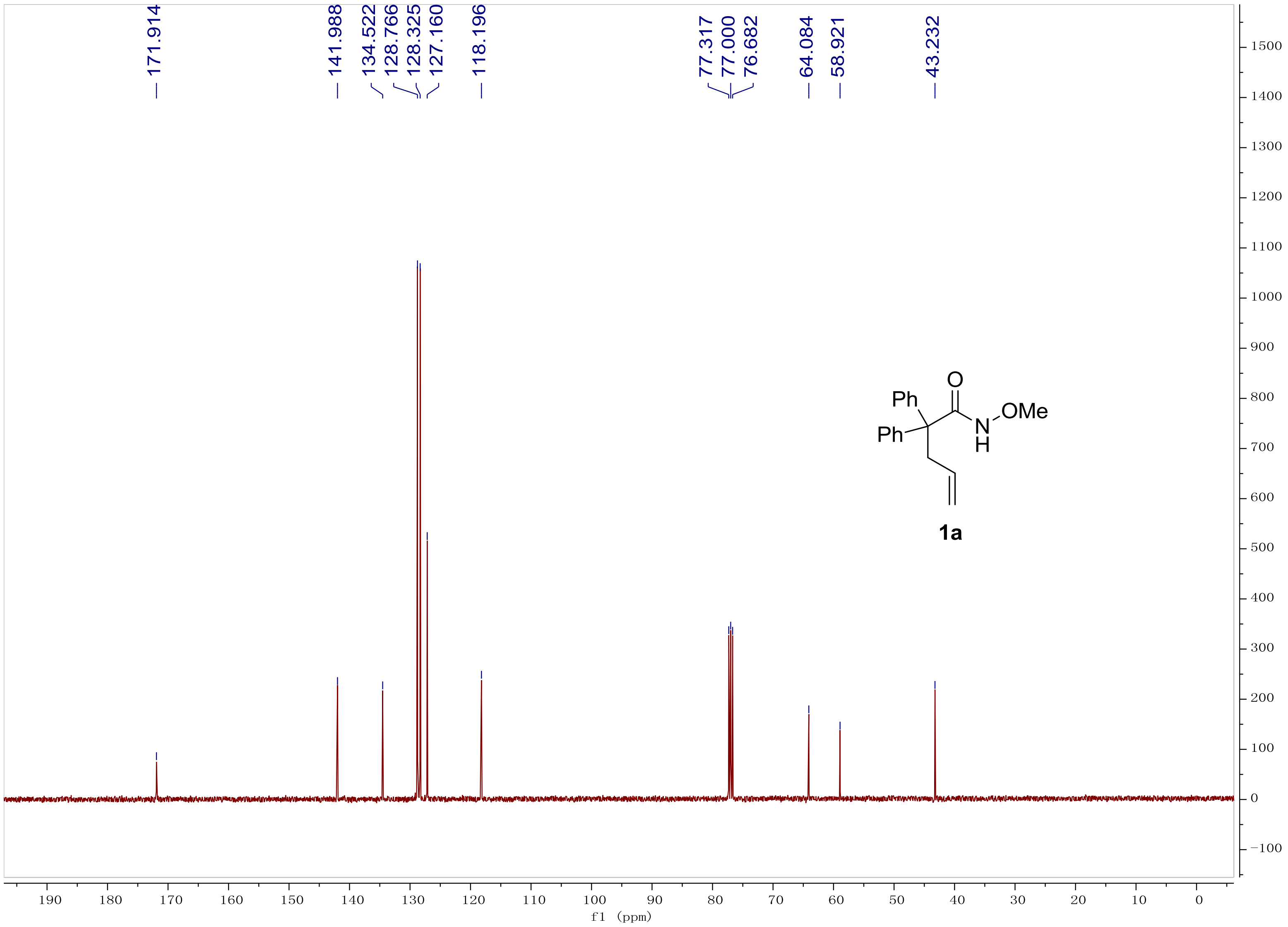
171.914
141.988
134.522
128.766
128.325
127.160
118.196
77.317
77.000
76.682
64.084
58.921
43.232
Ph
Ph
O
N
H
OMe
1a
f1 (ppm)

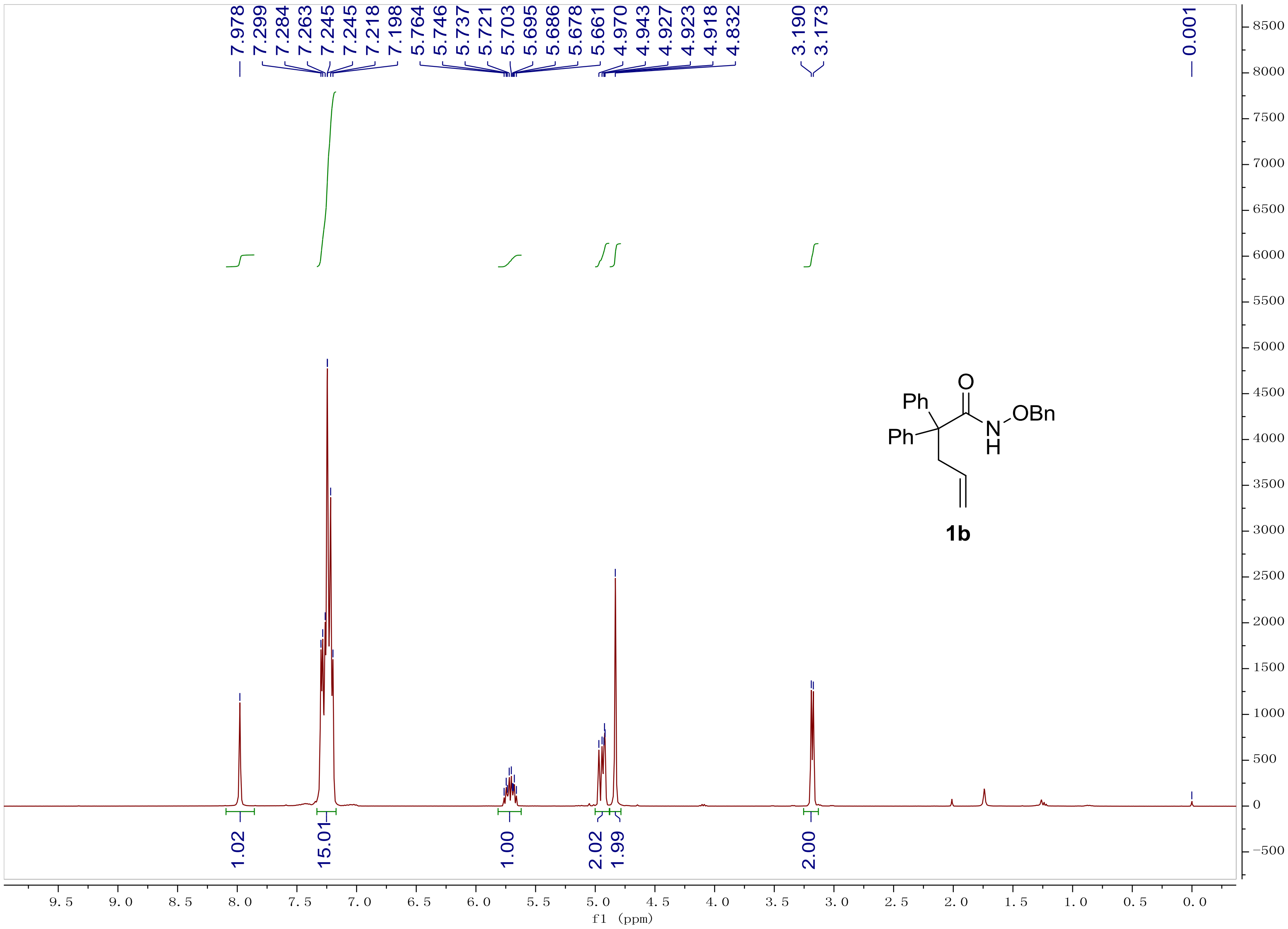
7.978
7.299
7.284
7.263
7.245
7.245
7.218
7.198
5.764
5.746
5.737
5.721
5.703
5.695
5.686
5.678
5.661
4.970
4.943
4.927
4.923
4.918
4.832
3.190
3.173
0.001
1.02
15.01
1.00
2.02
1.99
2.00
O
Ph
Ph
N
H
OBn
1b
f1 (ppm)

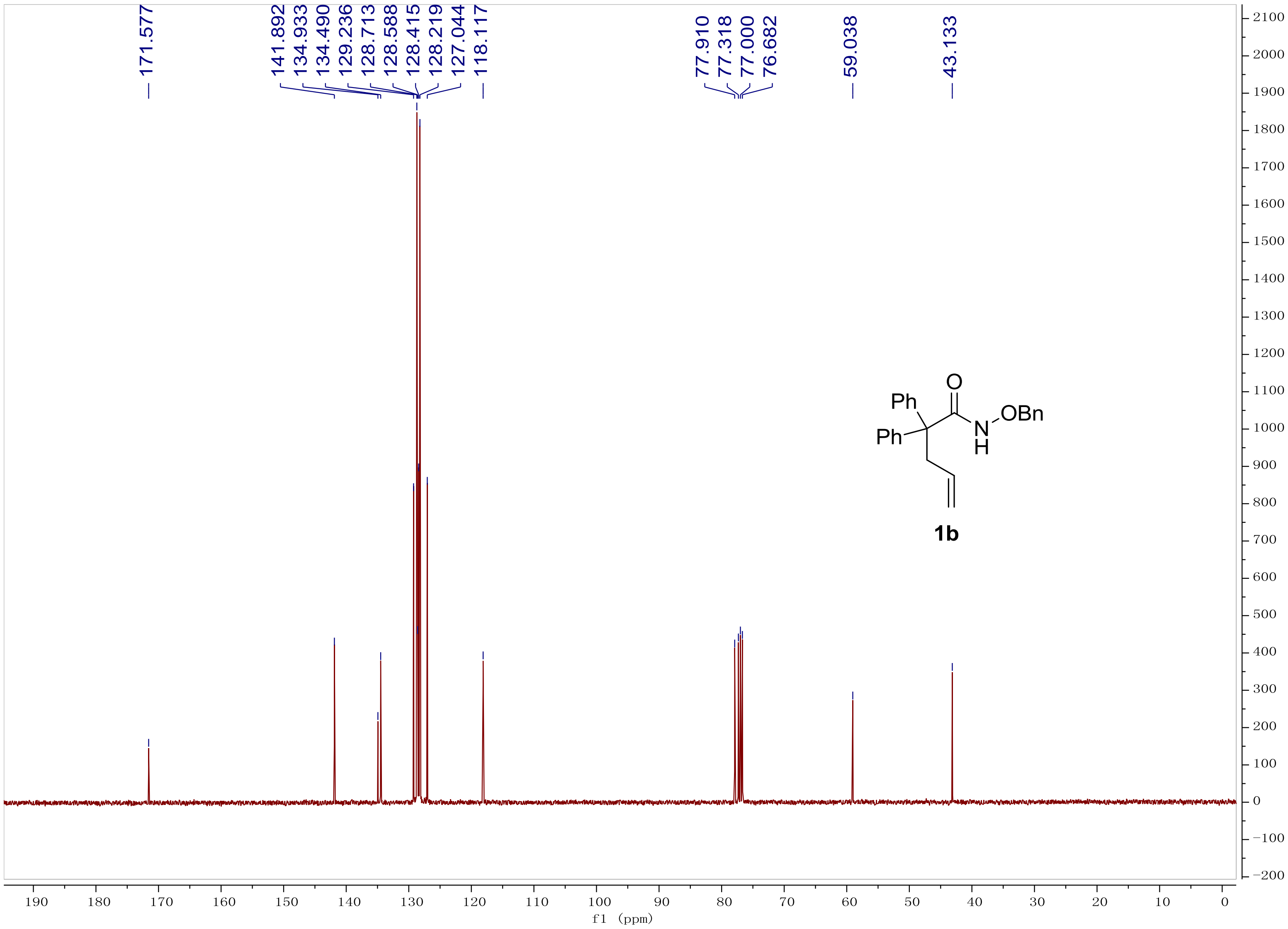
171.577
141.892
134.933
134.490
129.236
128.713
128.588
128.415
128.219
127.044
118.117
77.910
77.318
77.000
76.682
59.038
43.133
Ph
Ph
O
N
H
OBn
1b
f1 (ppm)

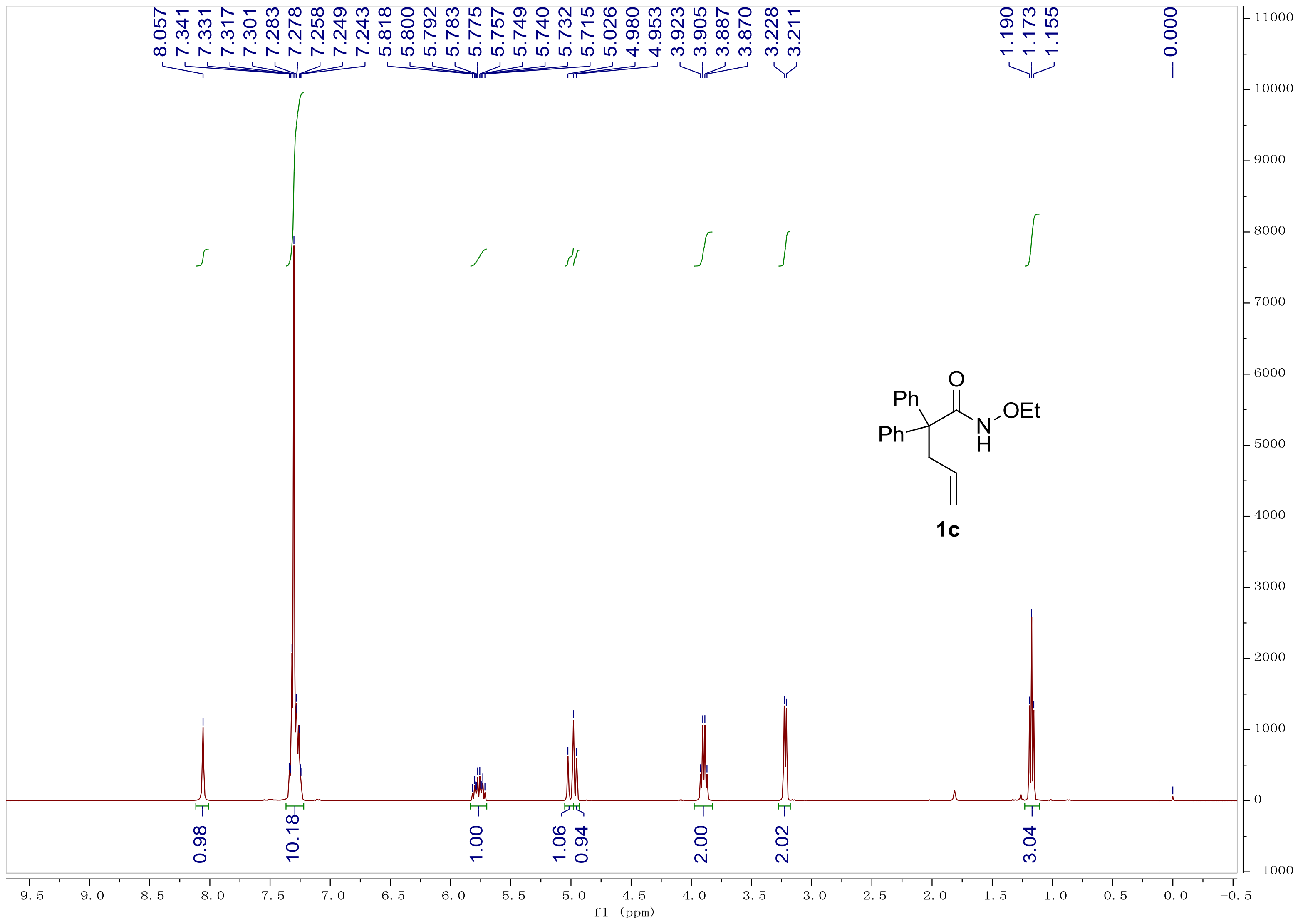
8.057
7.341
7.331
7.317
7.301
7.283
7.278
7.258
7.249
7.243
5.818
5.800
5.792
5.783
5.775
5.757
5.749
5.740
5.732
5.715
5.026
4.980
4.953
3.923
3.905
3.887
3.870
3.228
3.211
1.190
1.173
1.155
0.000
0.98
10.18
1.00
1.06
0.94
2.00
2.02
3.04
Ph
Ph
O
N
H
OEt
1c
f1 (ppm)

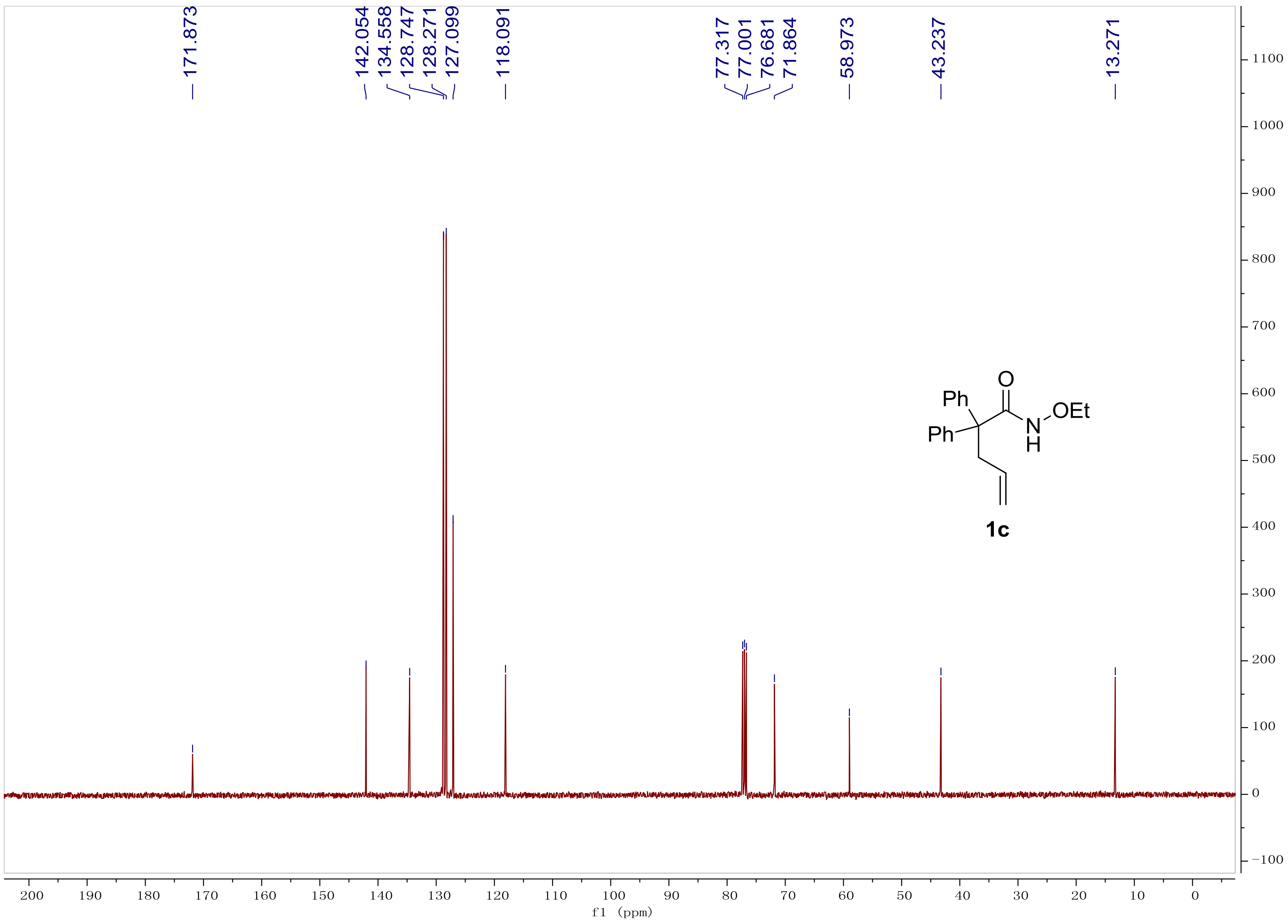
171.873
142.054
134.558
128.747
128.271
127.099
118.091
77.317
77.001
76.681
71.864
58.973
43.237
13.271
Ph
Ph
O
N
H
OEt
1c
f1 (ppm)

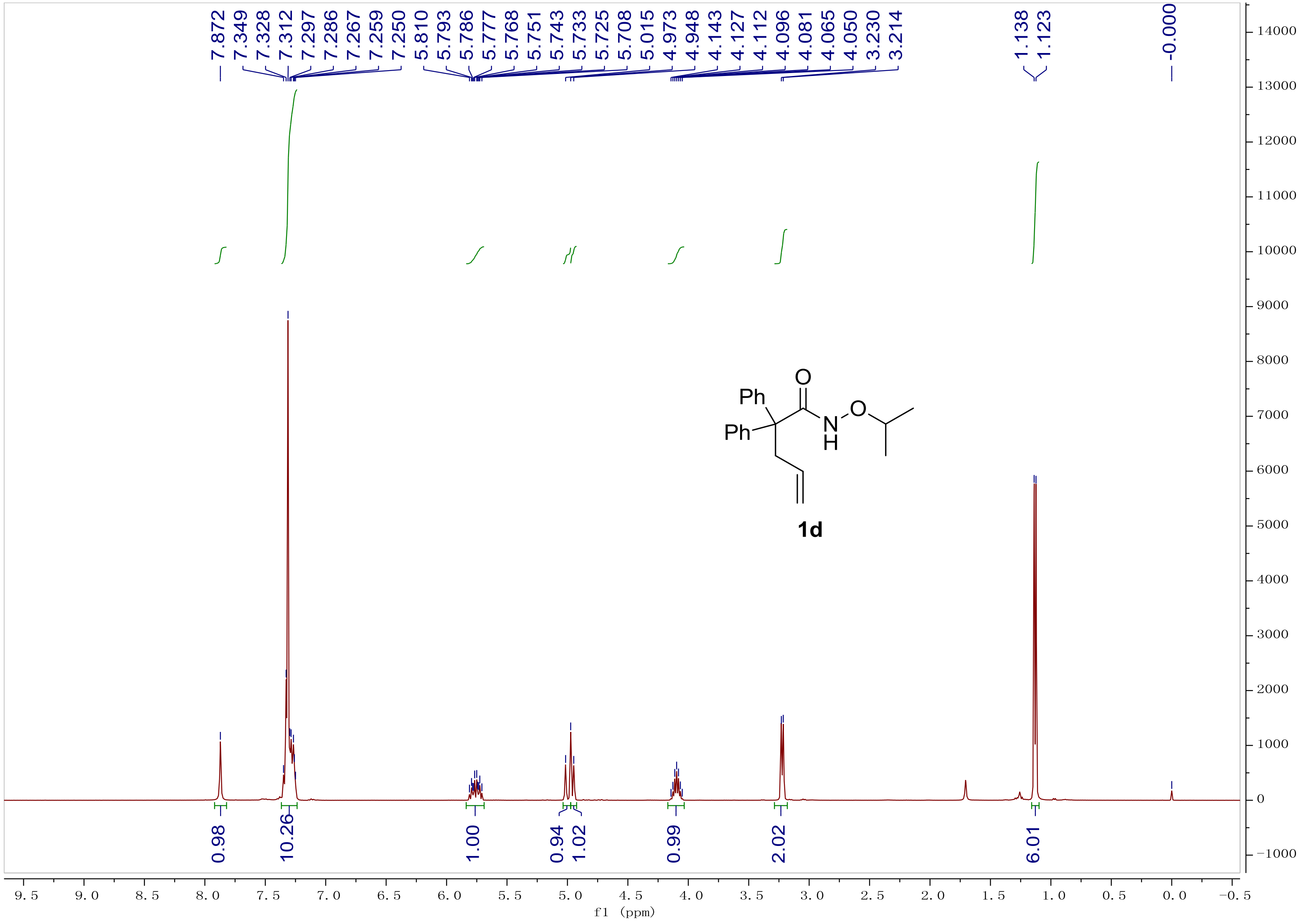

7.872
7.349
7.328
7.312
7.297
7.286
7.267
7.259
7.250
5.810
5.793
5.786
5.777
5.768
5.751
5.743
5.733
5.725
5.708
5.015
4.973
4.948
4.143
4.127
4.112
4.096
4.081
4.065
4.050
3.230
3.214
1.138
1.123
-0.000
0.98
10.26
1.00
0.94
1.02
0.99
2.02
6.01
Ph
Ph
O
N
H
O
1d
f1 (ppm)

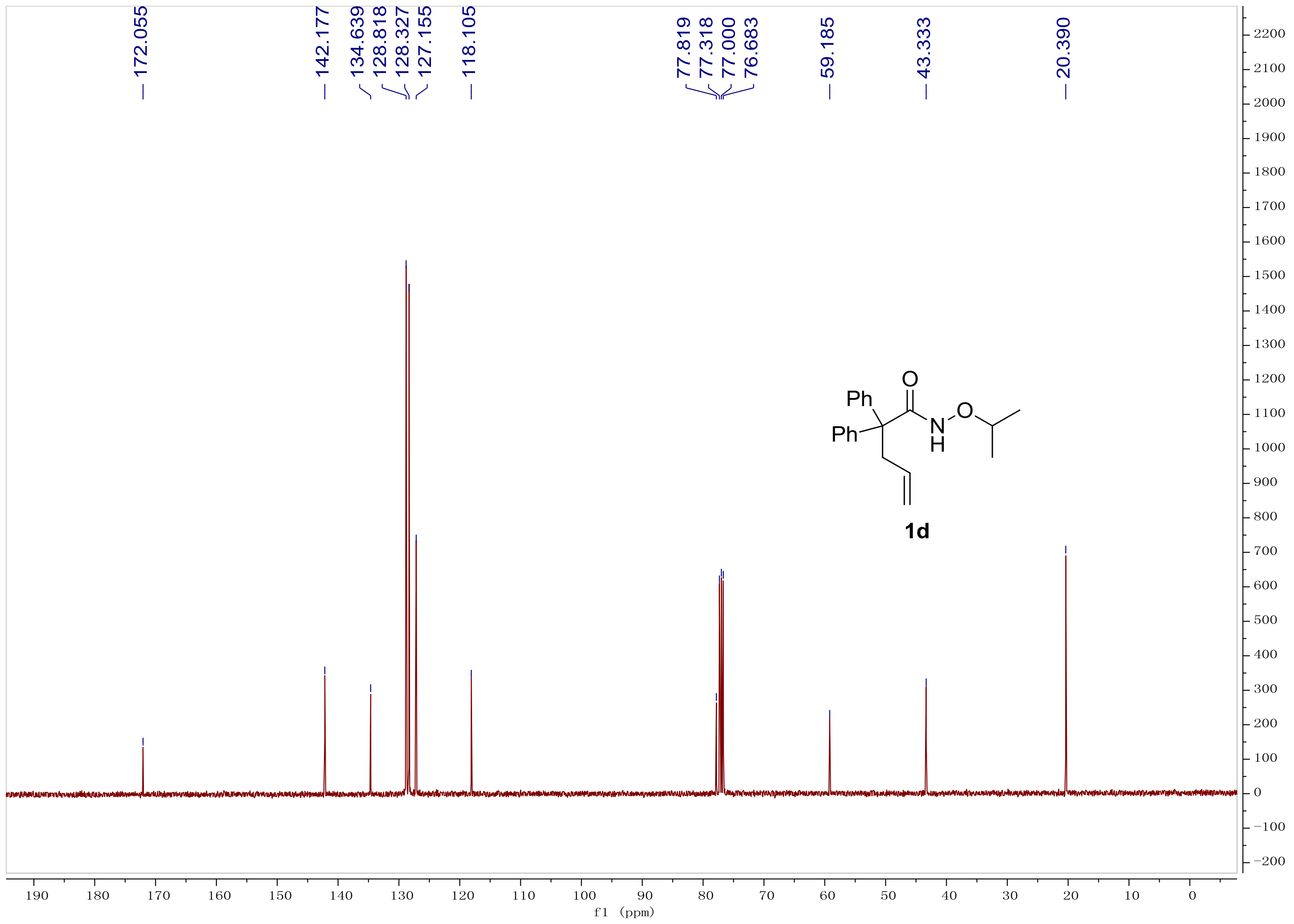
172.055
142.177
134.639
128.818
128.327
127.155
118.105
77.819
77.318
77.000
76.683
59.185
43.333
20.390
Ph
Ph
O
N
H
O
1d
f1 (ppm)

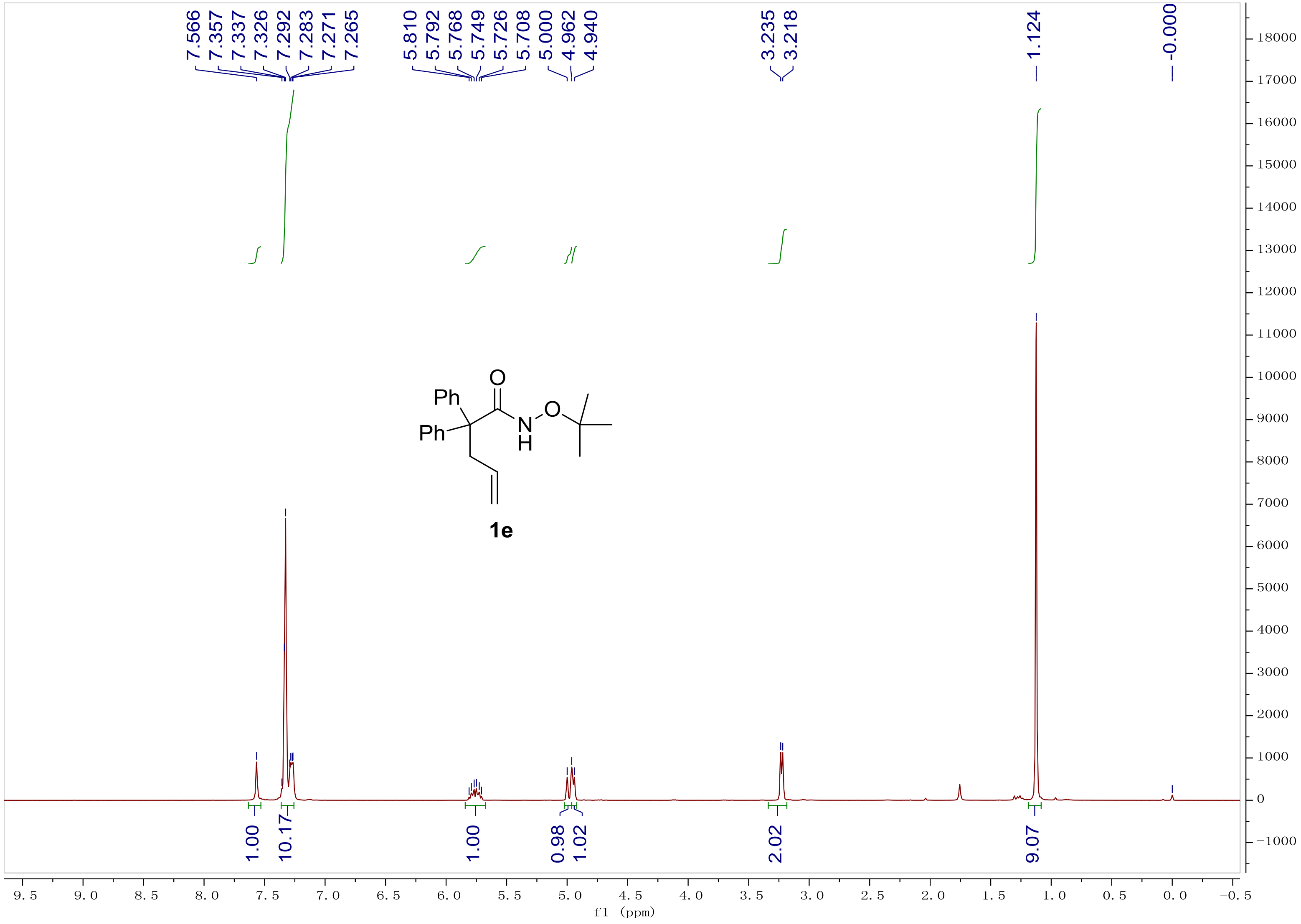

7.566
7.357
7.337
7.326
7.292
7.283
7.271
7.265
5.810
5.792
5.768
5.749
5.726
5.708
5.000
4.962
4.940
3.235
3.218
1.124
-0.000
Ph
Ph
O
N
H
O
1e
1.00
10.17
1.00
0.98
1.02
2.02
9.07
f1 (ppm)

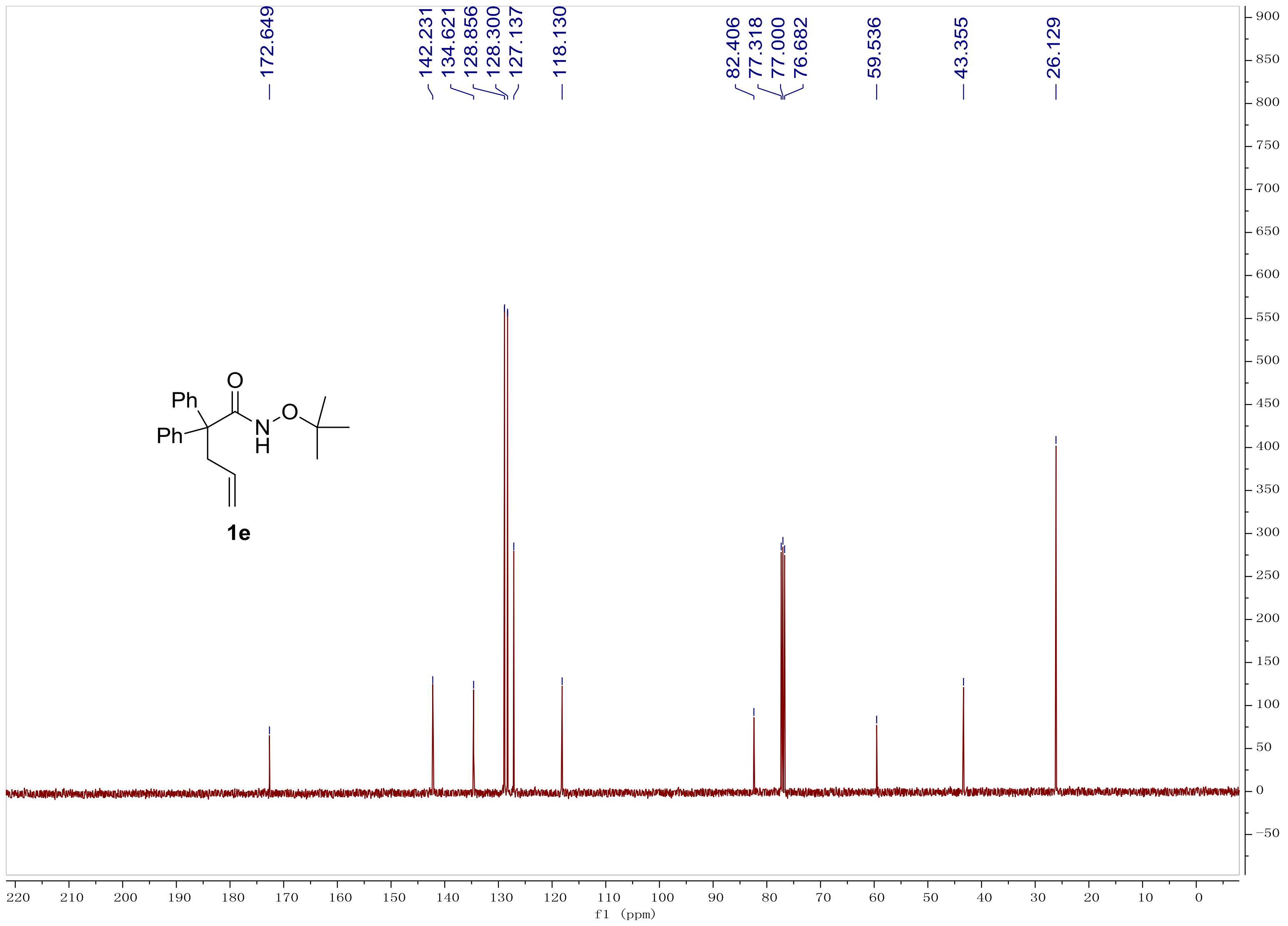

172.649
142.231
134.621
128.856
128.300
127.137
118.130
82.406
77.318
77.000
76.682
59.536
43.355
26.129
Ph
Ph
O
N
H
O
1e
f1 (ppm)

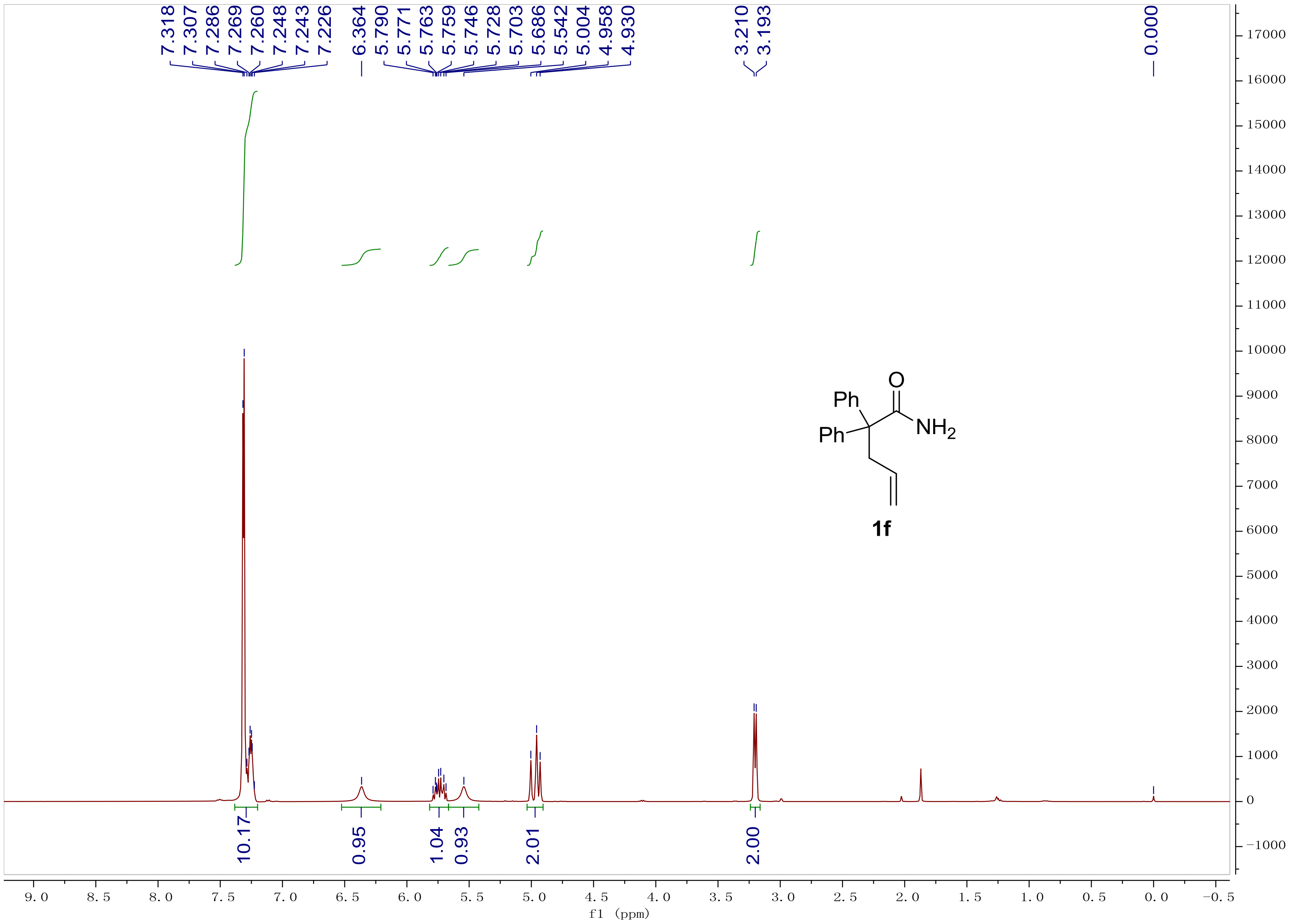

7.318
7.307
7.286
7.269
7.260
7.248
7.243
7.226
6.364
5.790
5.771
5.763
5.759
5.746
5.728
5.703
5.686
5.542
5.004
4.958
4.930
3.210
3.193
0.000
10.17
0.95
1.04
0.93
2.01
2.00
Ph
Ph
O
NH2
1f
f1 (ppm)

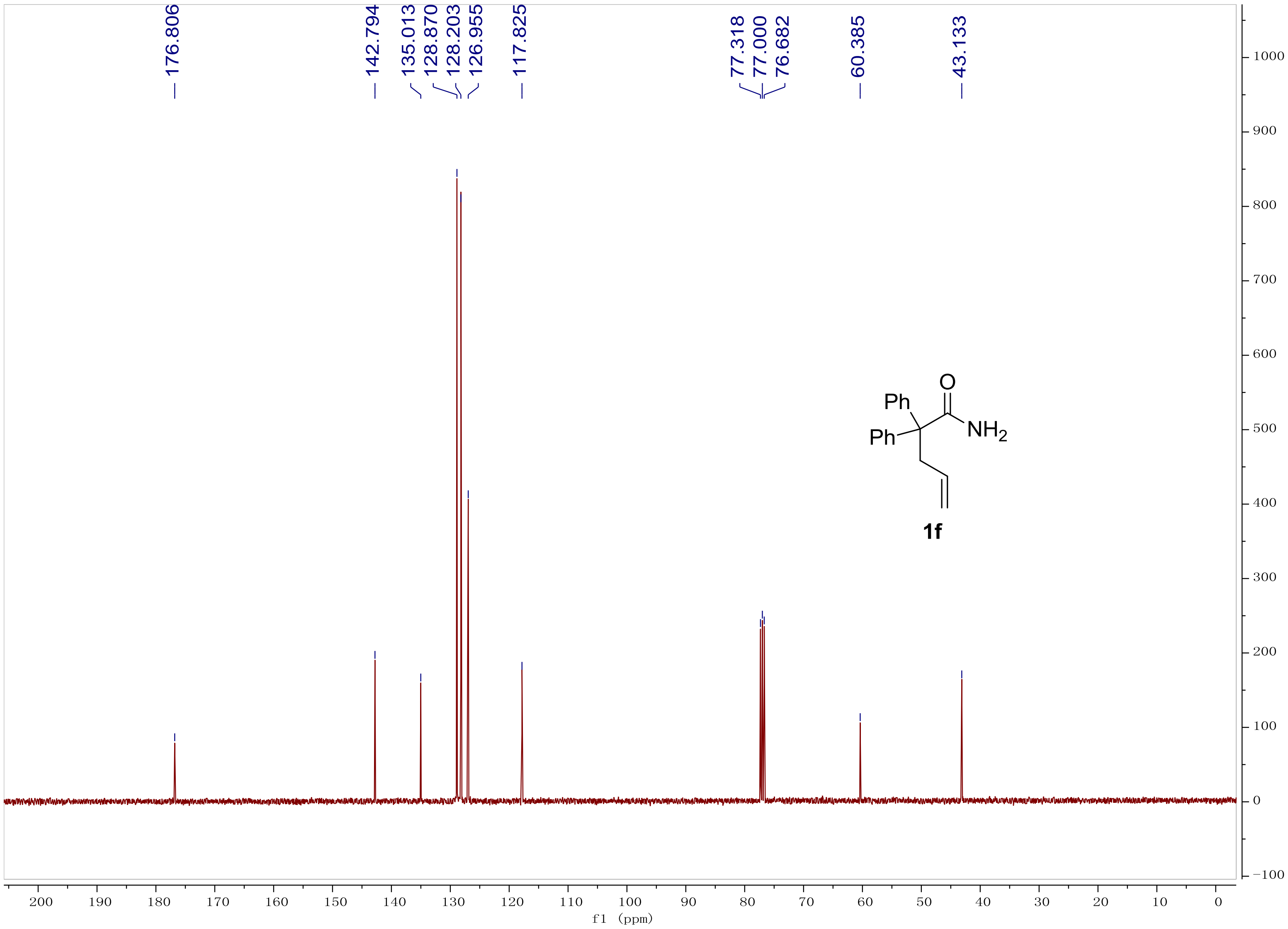

176.806
142.794
135.013
128.870
128.203
126.955
117.825
77.318
77.000
76.682
60.385
43.133
Ph
Ph
O
NH2
1f
f1 (ppm)

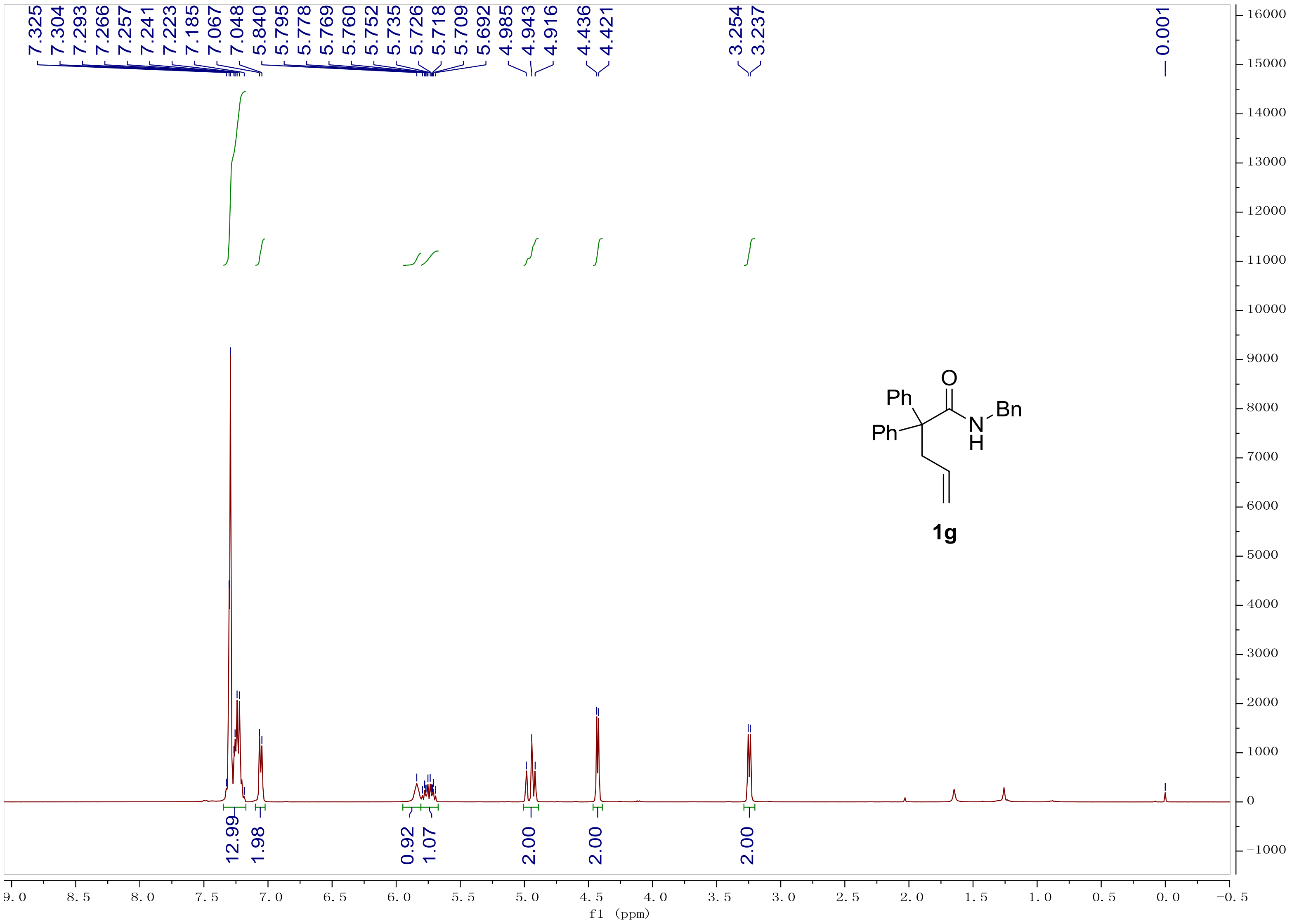
7.325
7.304
7.293
7.266
7.257
7.241
7.223
7.185
7.067
7.048
5.840
5.795
5.778
5.769
5.760
5.752
5.735
5.726
5.718
5.709
5.692
4.985
4.943
4.916
4.436
4.421
3.254
3.237
0.001
12.99
1.98
0.92
1.07
2.00
2.00
2.00
Ph
Ph
O
N
H
Bn
1g
f1 (ppm)

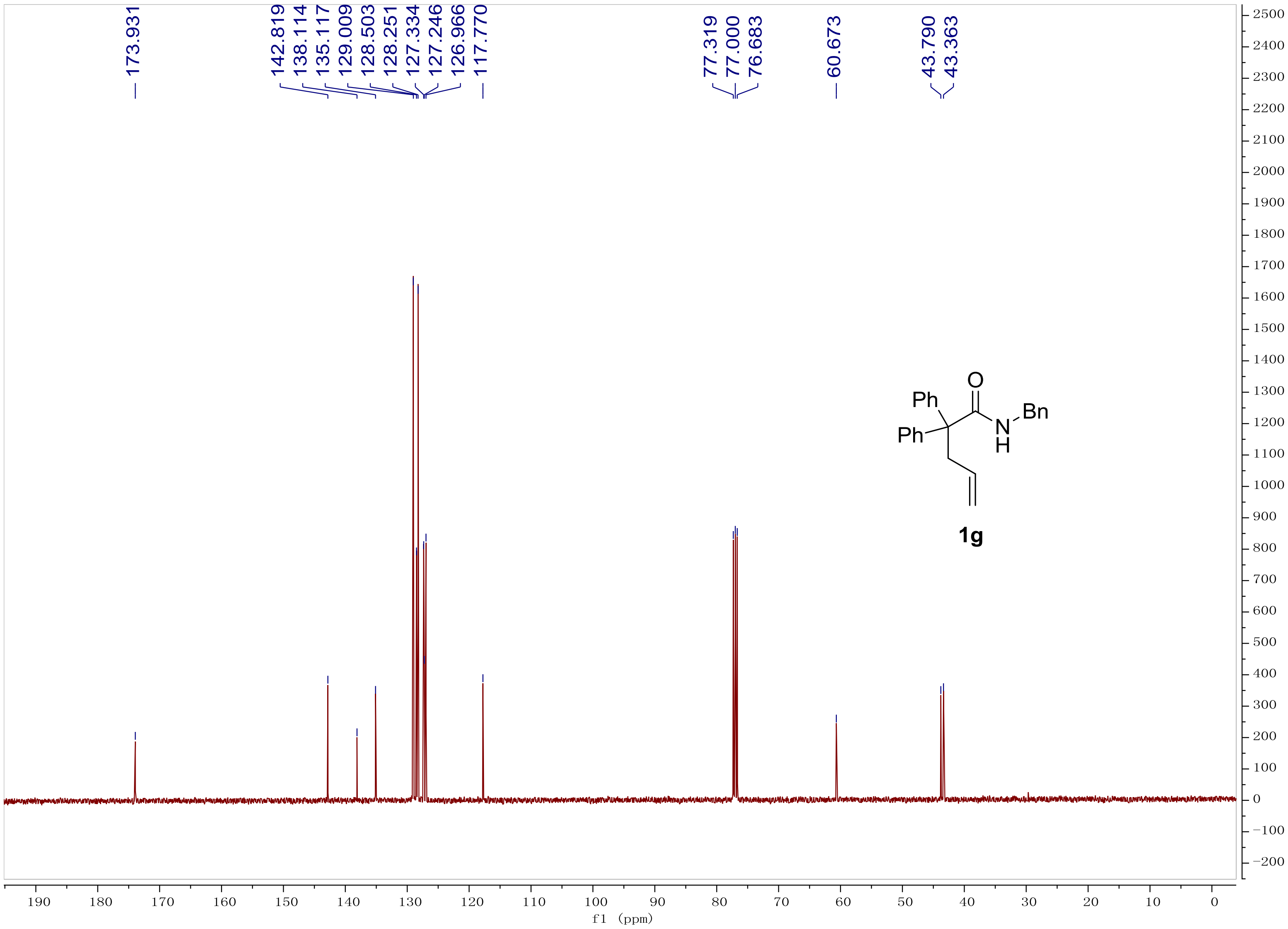
173.931
142.819
138.114
135.117
129.009
128.503
128.251
127.334
127.246
126.966
117.770
77.319
77.000
76.683
60.673
43.790
43.363
Ph
Ph
O
N
H
Bn
1g
f1 (ppm)

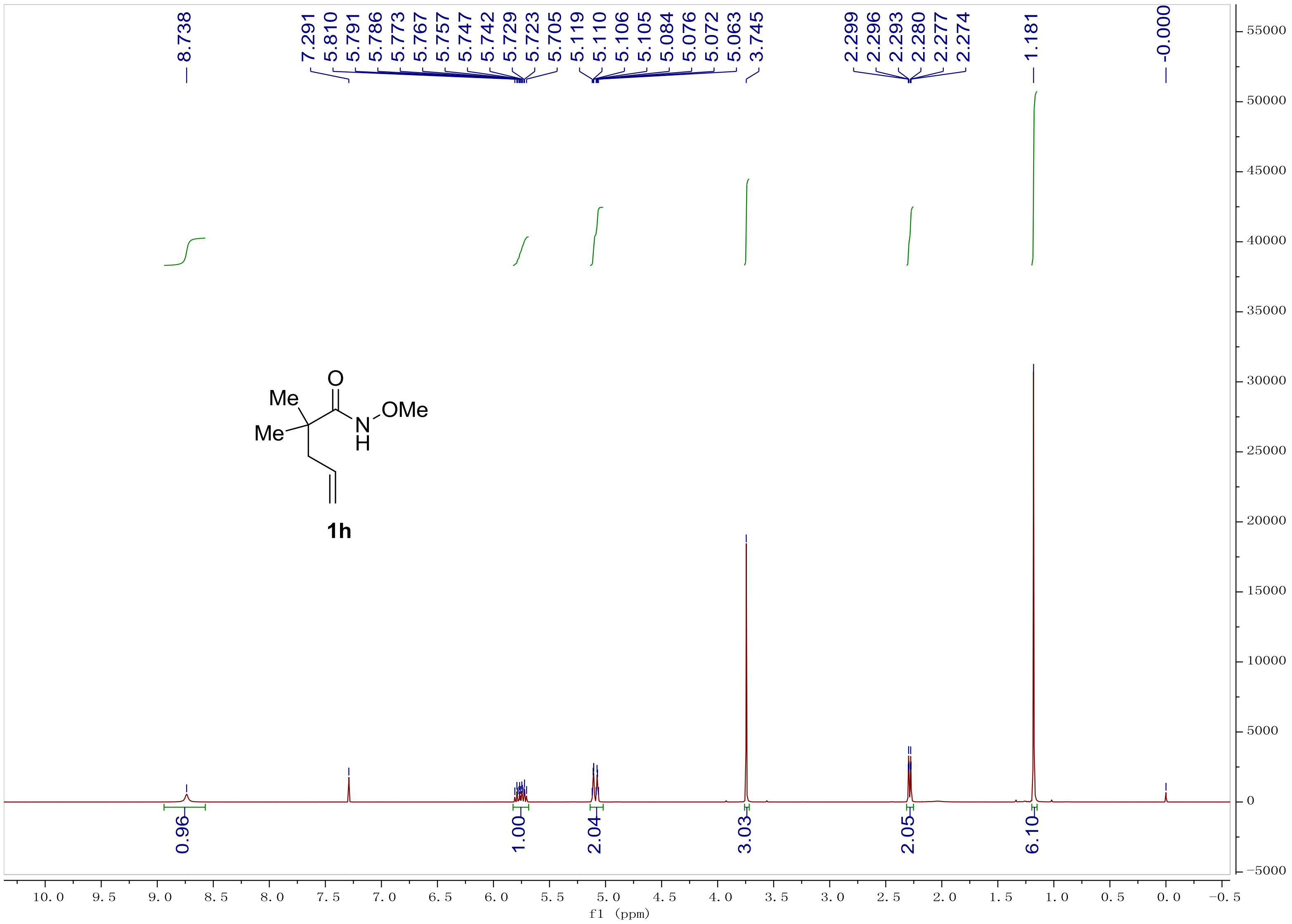
8.738
7.291
5.810
5.791
5.786
5.773
5.767
5.757
5.747
5.742
5.729
5.723
5.705
5.119
5.110
5.106
5.105
5.084
5.076
5.072
5.063
3.745
2.299
2.296
2.293
2.280
2.277
2.274
1.181
-0.000
Me
Me
O
N
H
OMe
1h
0.96
1.00
2.04
3.03
2.05
6.10
f1 (ppm)

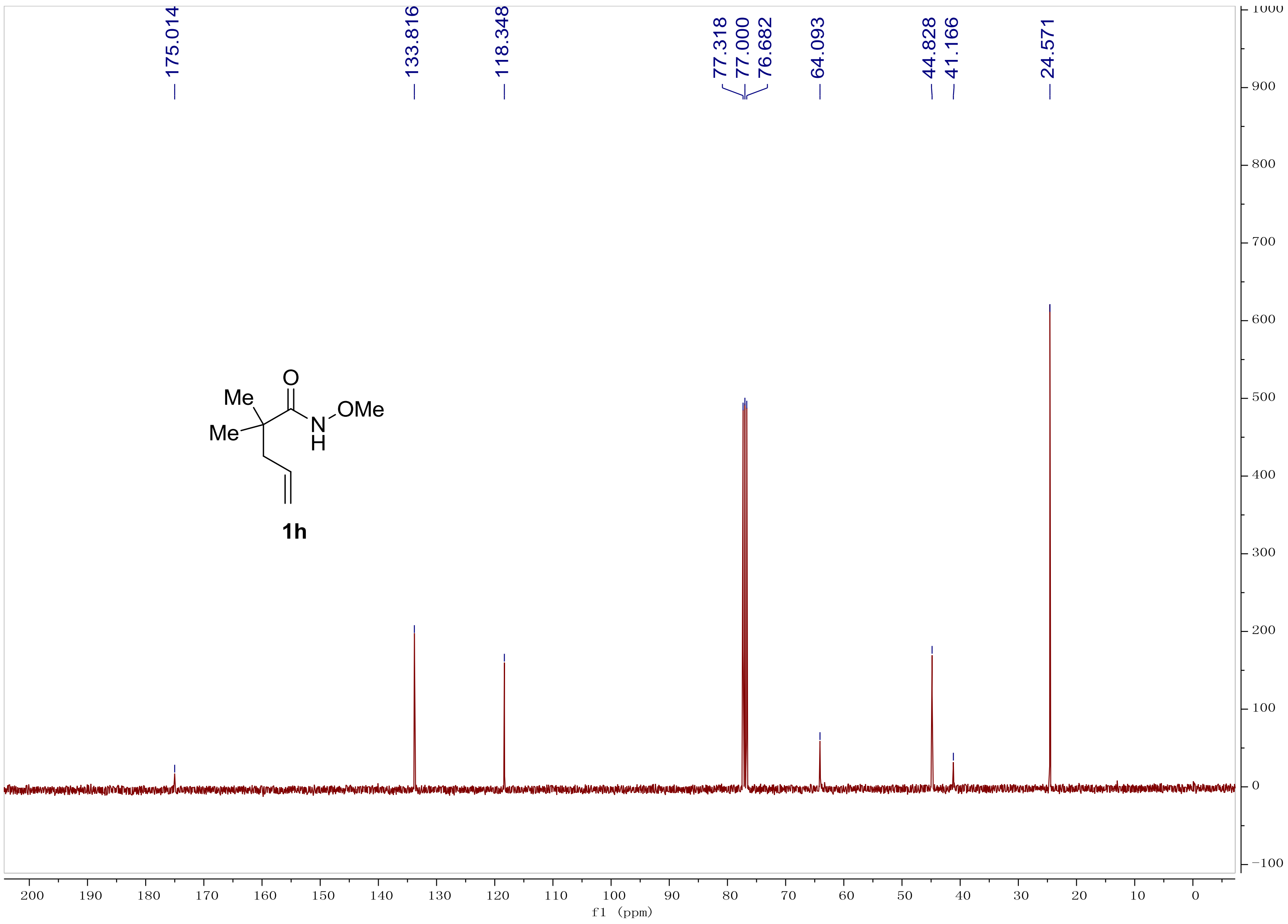
175.014
133.816
118.348
77.318
77.000
76.682
64.093
44.828
41.166
24.571
Me
Me
O
N
H
OMe
1h
f1 (ppm)
200 190 180 170 160 150 140 130 120 110 100 90 80 70 60 50 40 30 20 10 0
1000 900 800 700 600 500 400 300 200 100 0 −100

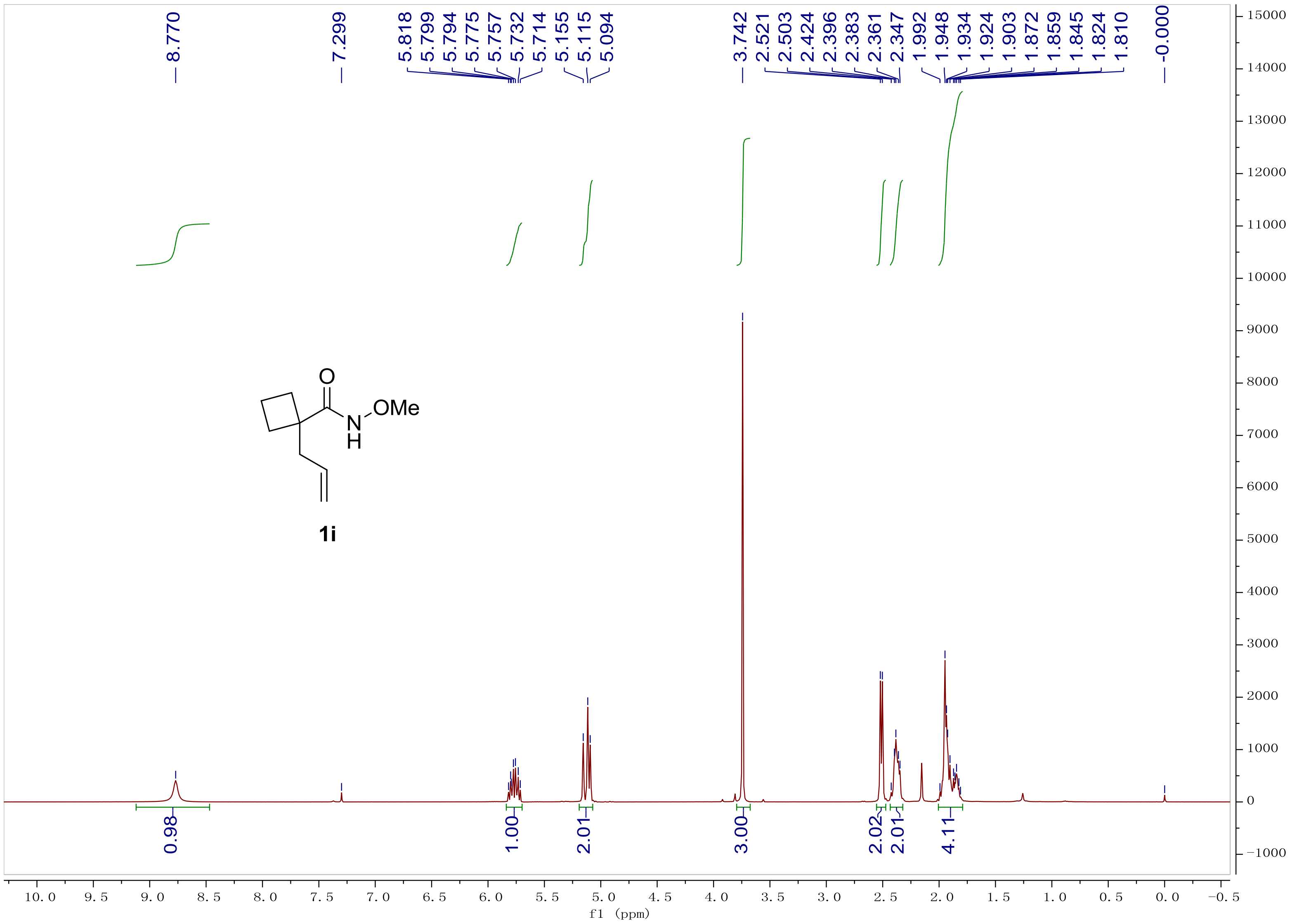
8.770
7.299
5.818
5.799
5.794
5.775
5.757
5.732
5.714
5.155
5.115
5.094
3.742
2.521
2.503
2.424
2.396
2.383
2.361
2.347
1.992
1.948
1.934
1.924
1.903
1.872
1.859
1.845
1.824
1.810
-0.000
O
OMe
N
H
1i
0.98
1.00
2.01
3.00
2.02
2.01
4.11
f1 (ppm)

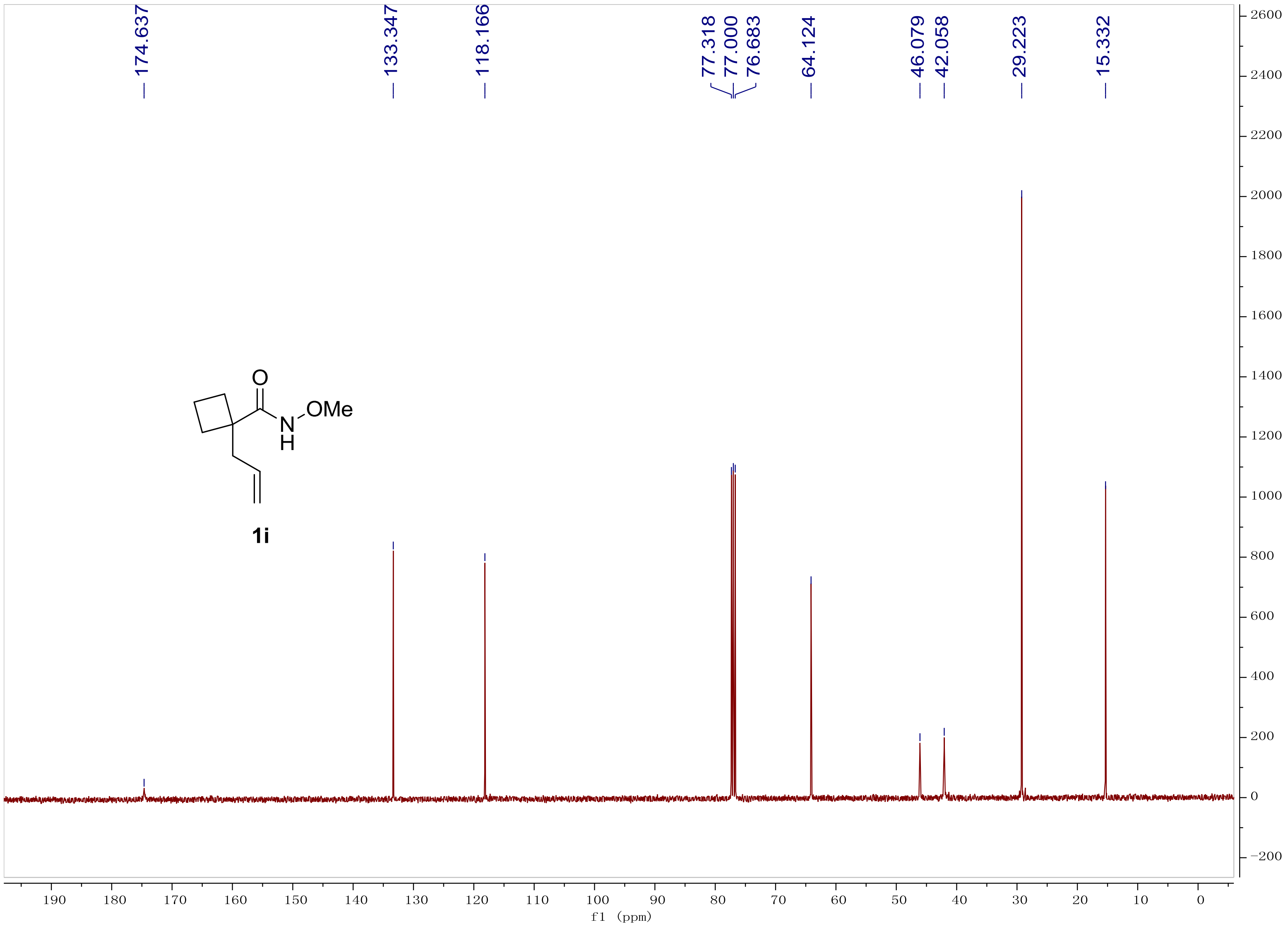

174.637
133.347
118.166
77.318
77.000
76.683
64.124
46.079
42.058
29.223
15.332
O
N
H
OMe
1i
f1 (ppm)

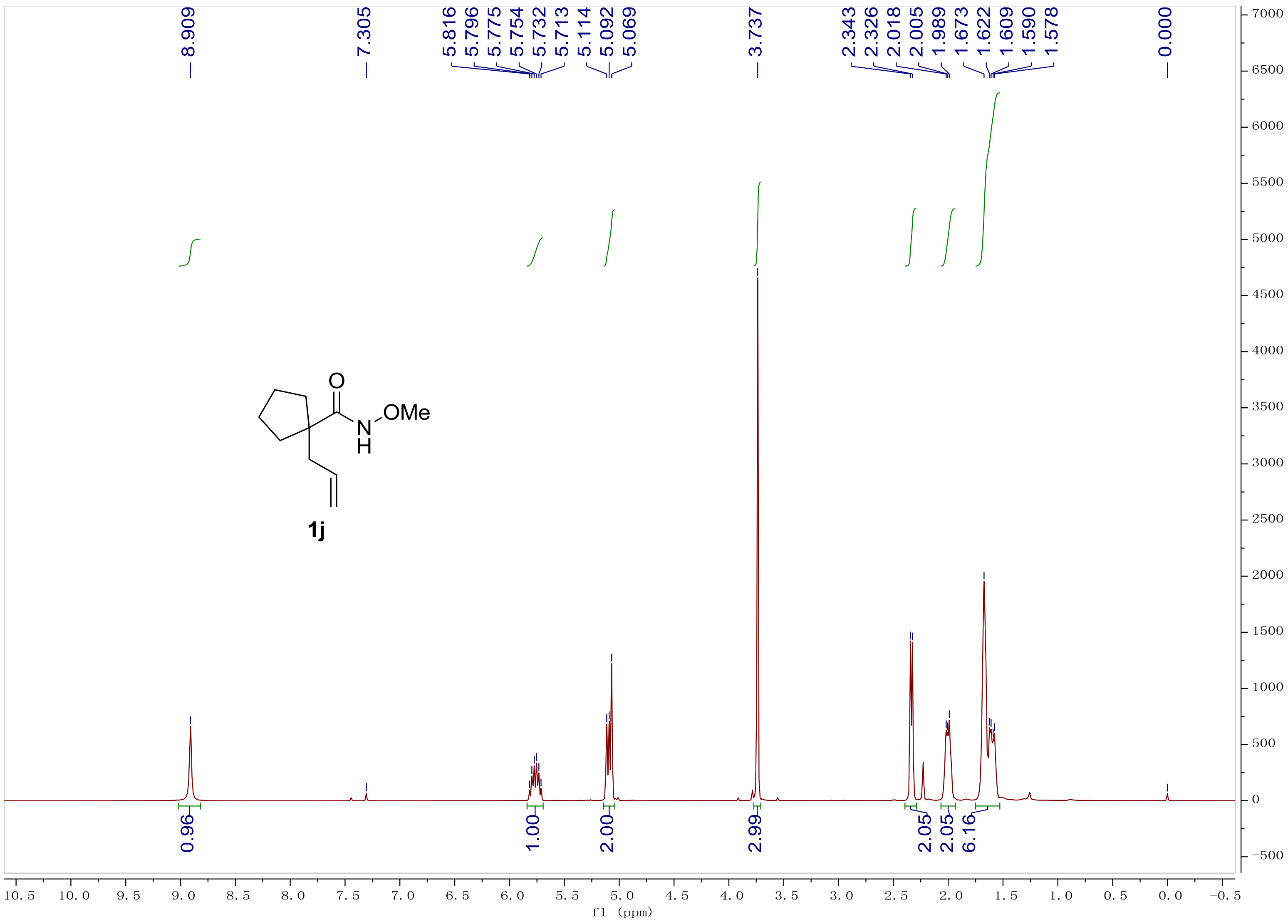
1j

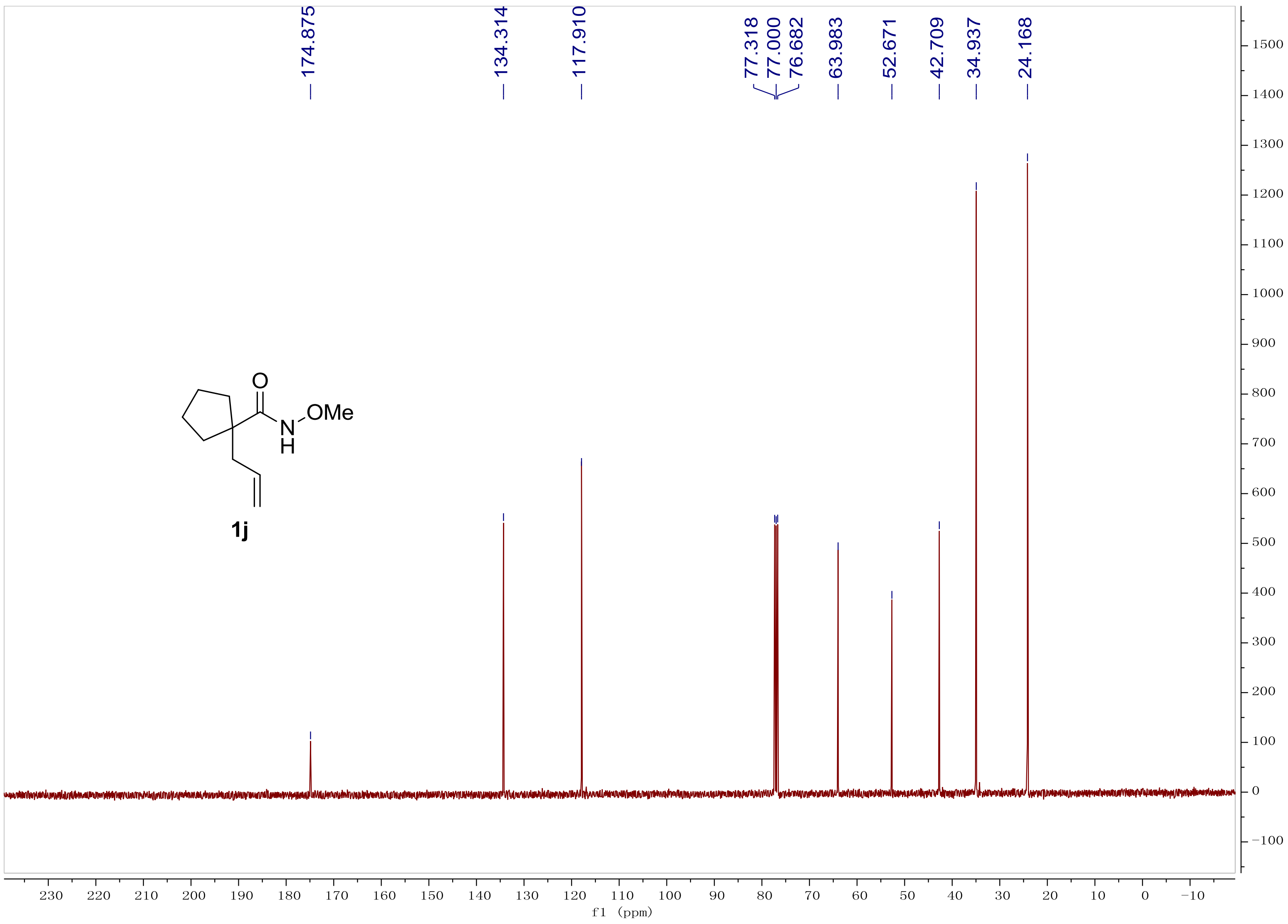
174.875
134.314
117.910
77.318
77.000
76.682
63.983
52.671
42.709
34.937
24.168
O
N
H
OMe
1j
f1 (ppm)

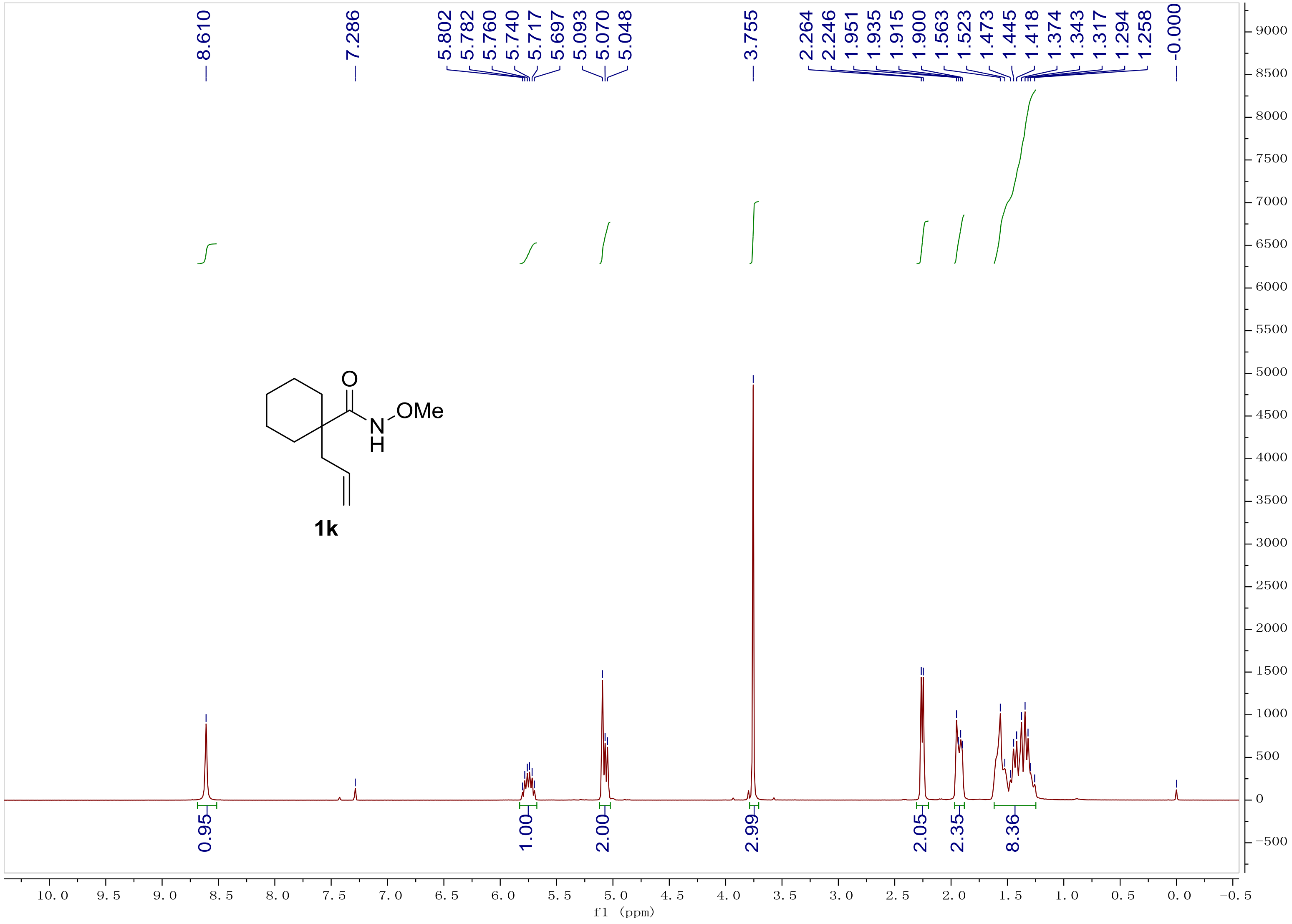

8.610
7.286
5.802 5.782 5.760 5.740 5.717 5.697 5.093 5.070 5.048
3.755
2.264 2.246 1.951 1.935 1.915 1.900 1.563 1.523 1.473 1.445 1.418 1.374 1.343 1.317 1.294 1.258
-0.000
O
N
OMe
H
1k
0.95
1.00
2.00
2.99
2.05
2.35
8.36
f1 (ppm)

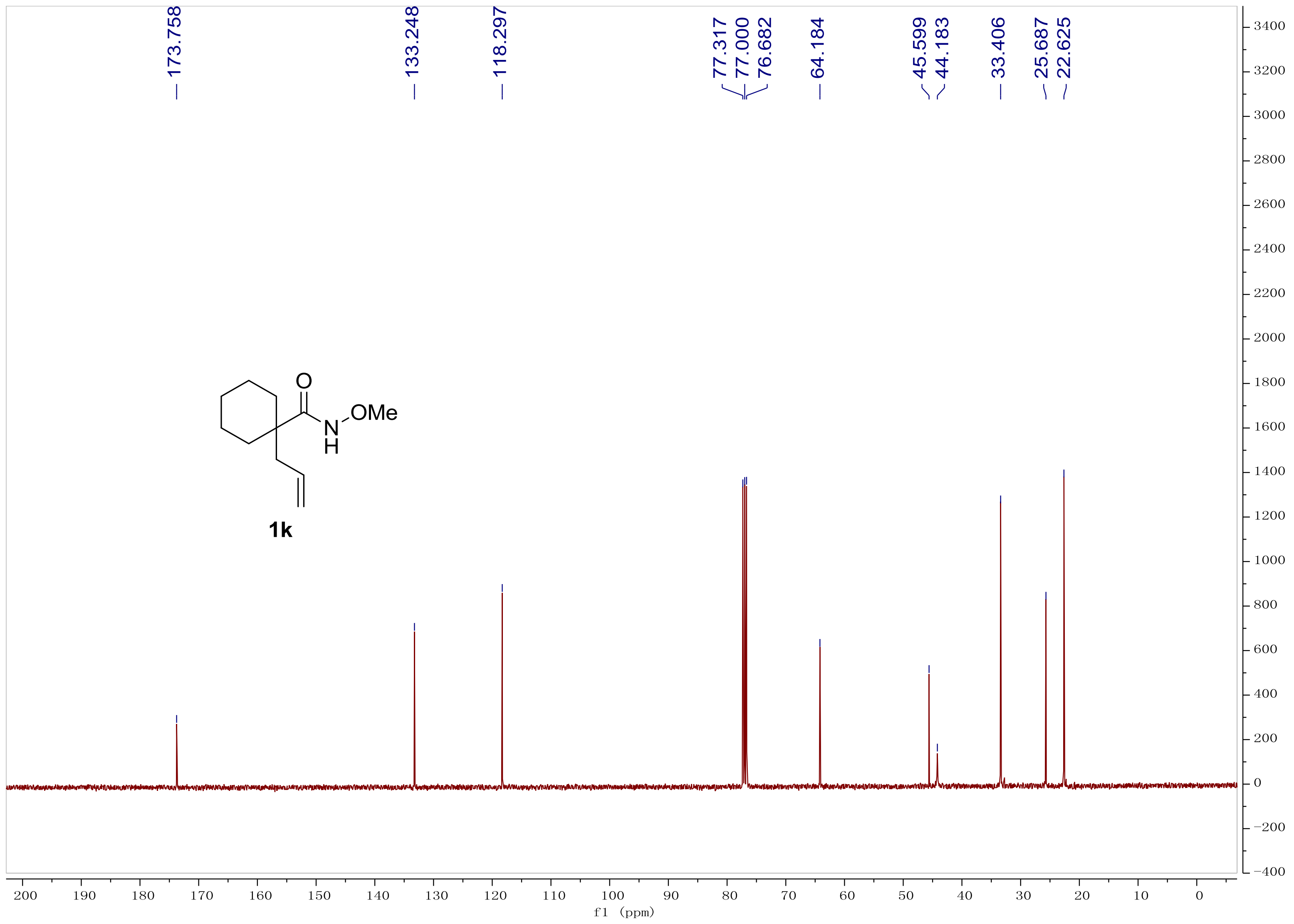
173.758
133.248
118.297
77.317
77.000
76.682
64.184
45.599
44.183
33.406
25.687
22.625
OMe
N
H
O
1k
f1 (ppm)

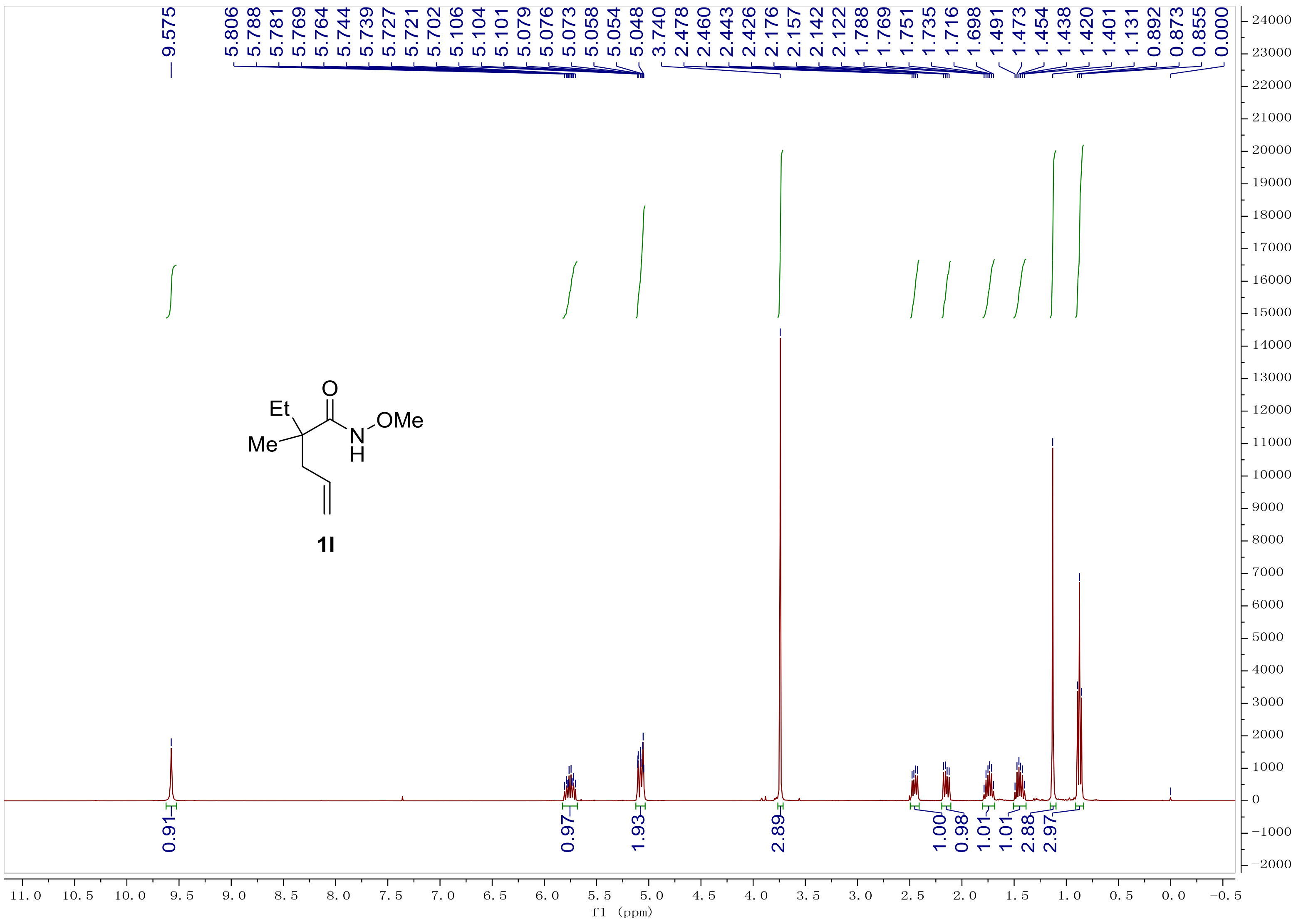

9.575
5.806 5.788 5.781 5.769 5.764 5.744 5.739 5.727 5.721 5.702 5.106 5.104 5.101 5.079 5.076 5.073 5.058 5.054 5.048 3.740 2.478 2.460 2.443 2.426 2.176 2.157 2.142 2.122 1.788 1.769 1.751 1.735 1.716 1.698 1.491 1.473 1.454 1.438 1.420 1.401 1.131 0.892 0.873 0.855 0.000
O
Et
Me
N
H
OMe
1l
0.91
0.97
1.93
2.89
1.00
0.98
1.01
1.01
2.88
2.97
f1 (ppm)

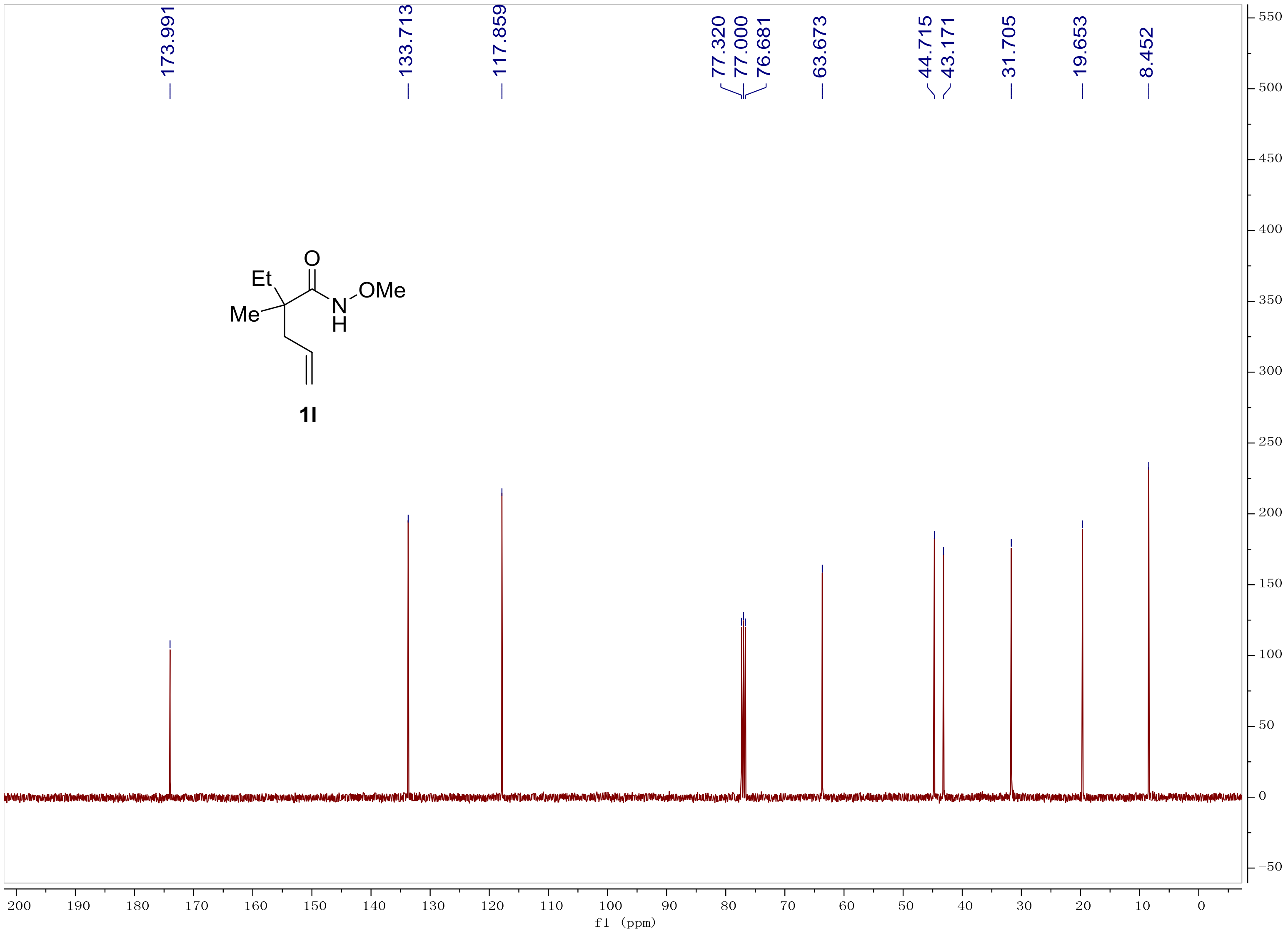

173.991
133.713
117.859
77.320
77.000
76.681
63.673
44.715
43.171
31.705
19.653
8.452
Et
Me
O
N
H
OMe
1l
f1 (ppm)

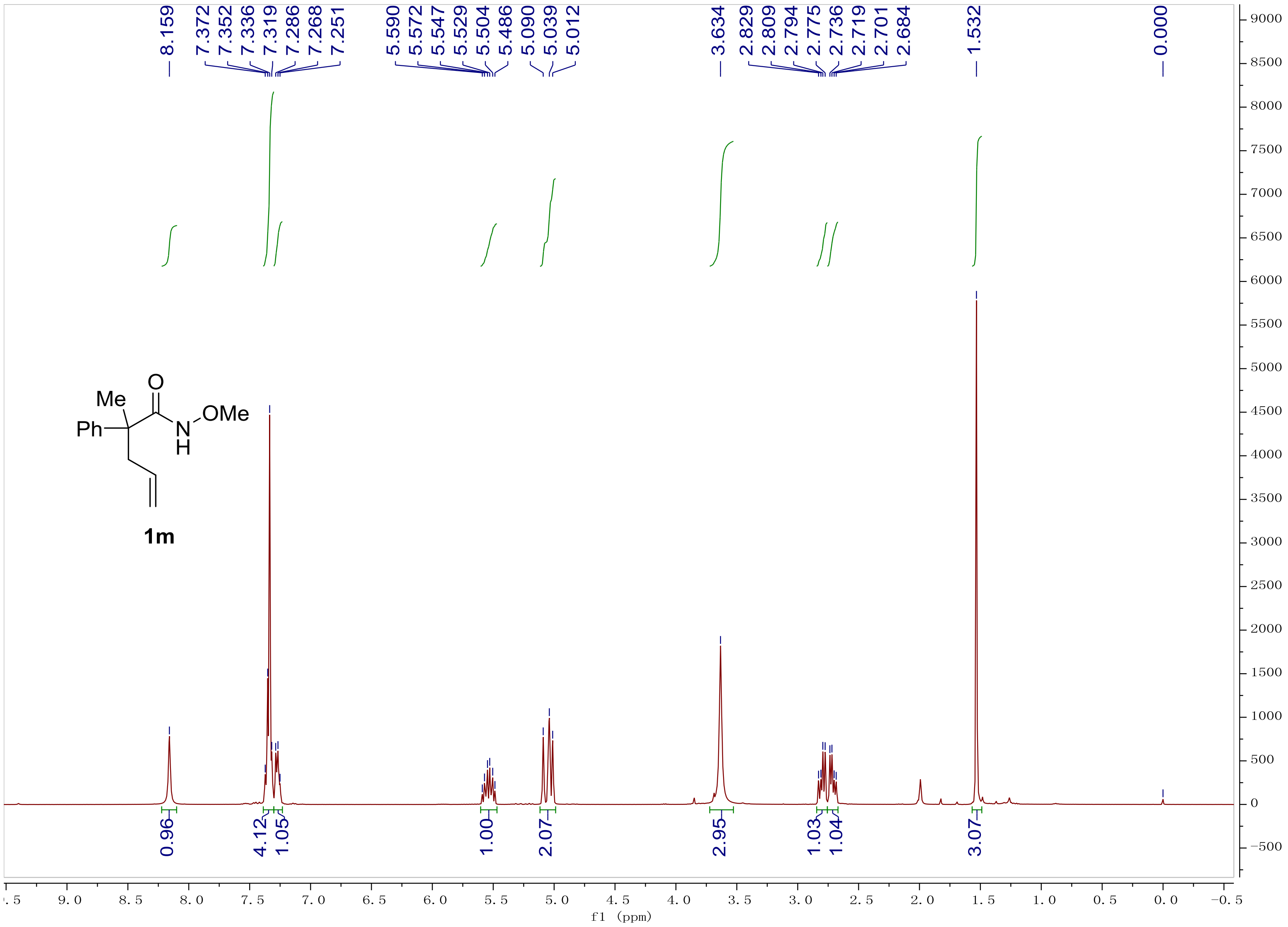

8.159
7.372
7.352
7.336
7.319
7.286
7.268
7.251
5.590
5.572
5.547
5.529
5.504
5.486
5.090
5.039
5.012
3.634
2.829
2.809
2.794
2.775
2.736
2.719
2.701
2.684
1.532
0.000
Me
O
Ph
N
OMe
H
1m
0.96
4.12
1.05
1.00
2.07
2.95
1.03
1.04
3.07
f1 (ppm)

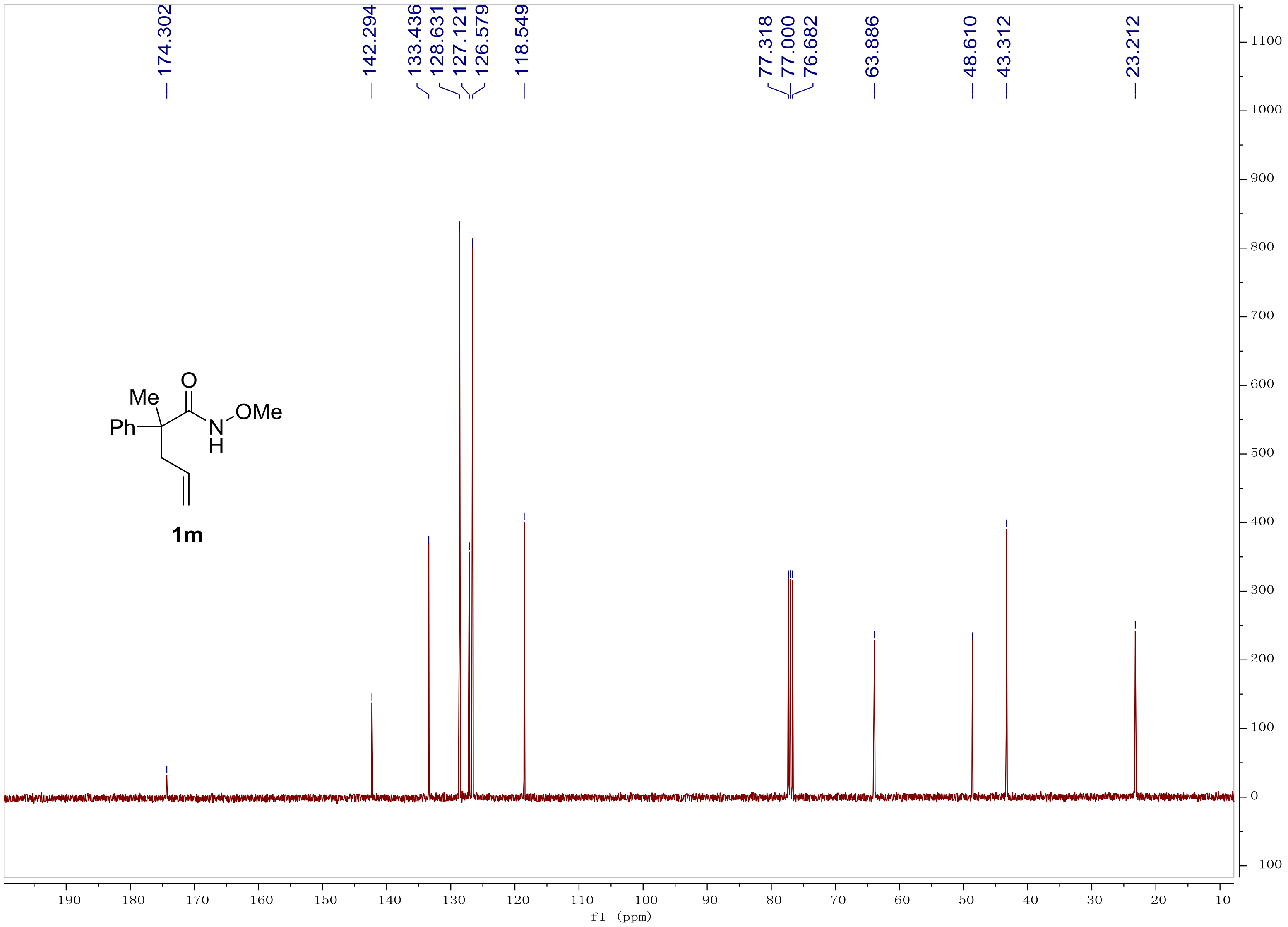

174.302
142.294
133.436
128.631
127.121
126.579
118.549
77.318
77.000
76.682
63.886
48.610
43.312
23.212
Me
O
Ph
N
OMe
H
1m
f1 (ppm)

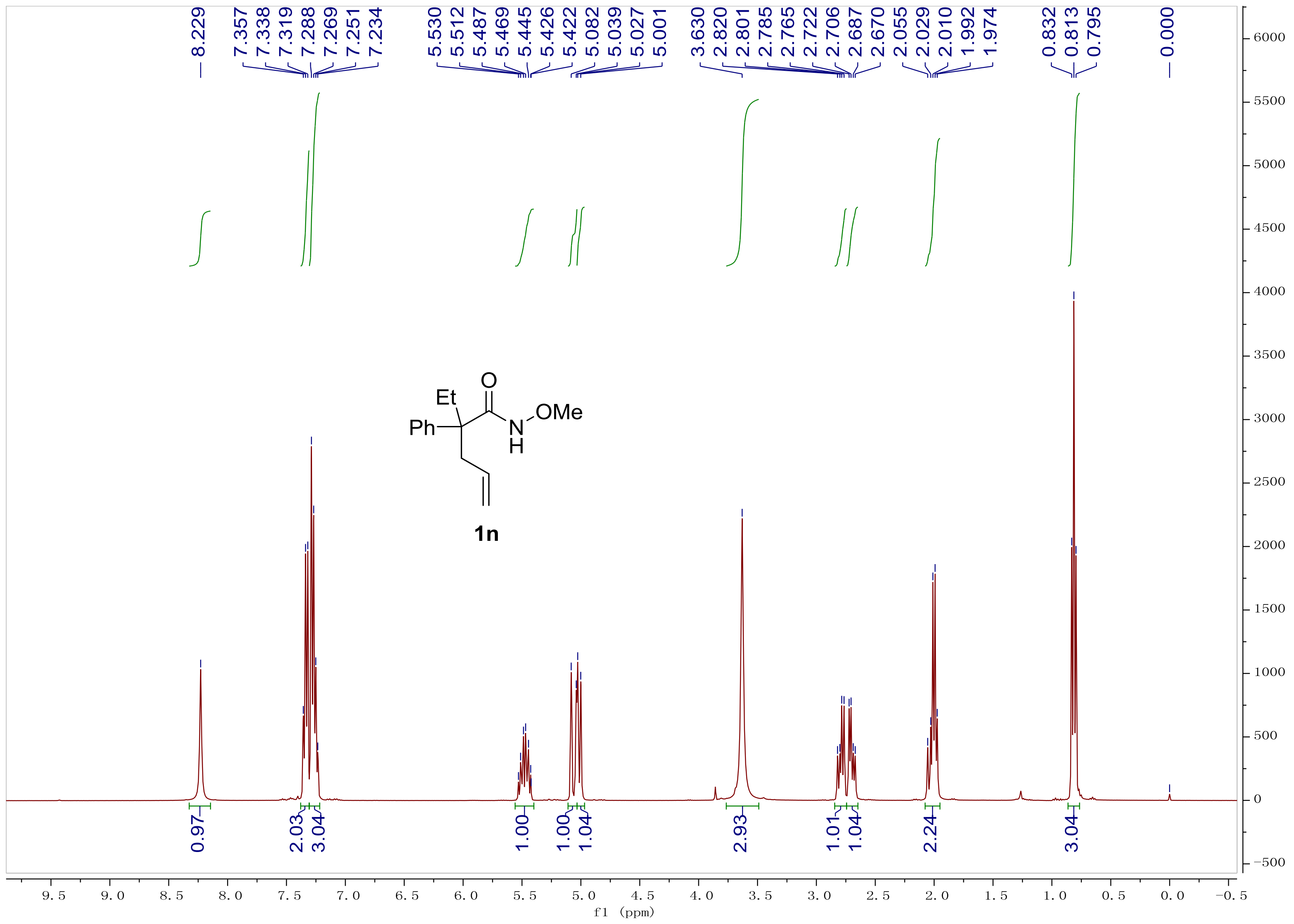
8.229
7.357
7.338
7.319
7.288
7.269
7.251
7.234
5.530
5.512
5.487
5.469
5.445
5.426
5.422
5.082
5.039
5.027
5.001
3.630
2.820
2.801
2.785
2.765
2.722
2.706
2.687
2.670
2.055
2.029
2.010
1.992
1.974
0.832
0.813
0.795
0.000
Et
O
Ph
N
OMe
H
1n
0.97
2.03
3.04
1.00
1.00
1.04
2.93
1.01
1.04
2.24
3.04
f1 (ppm)

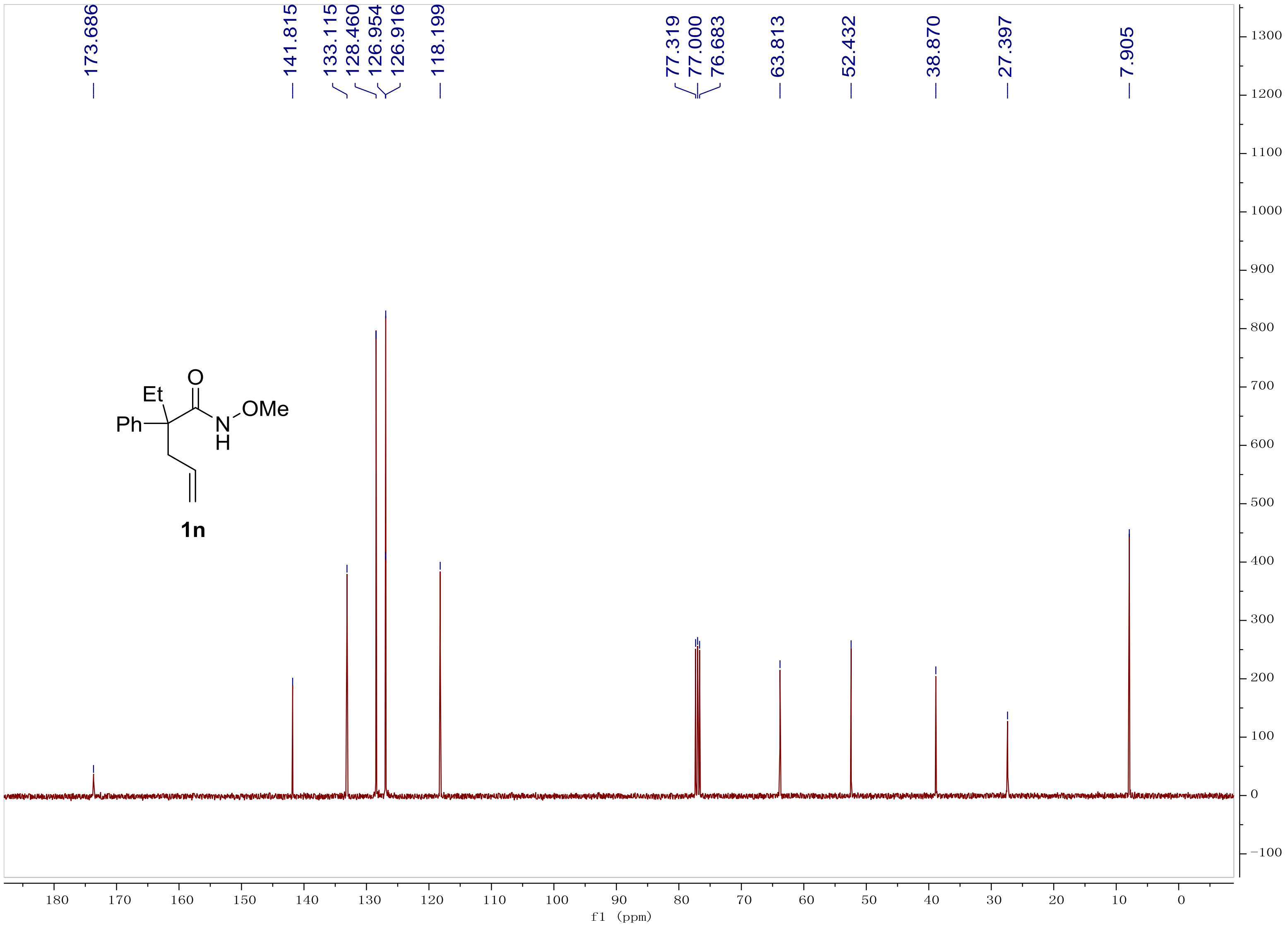
173.686
141.815
133.115
128.460
126.954
126.916
118.199
77.319
77.000
76.683
63.813
52.432
38.870
27.397
7.905
Et
O
Ph
N
OMe
H
1n
f1 (ppm)

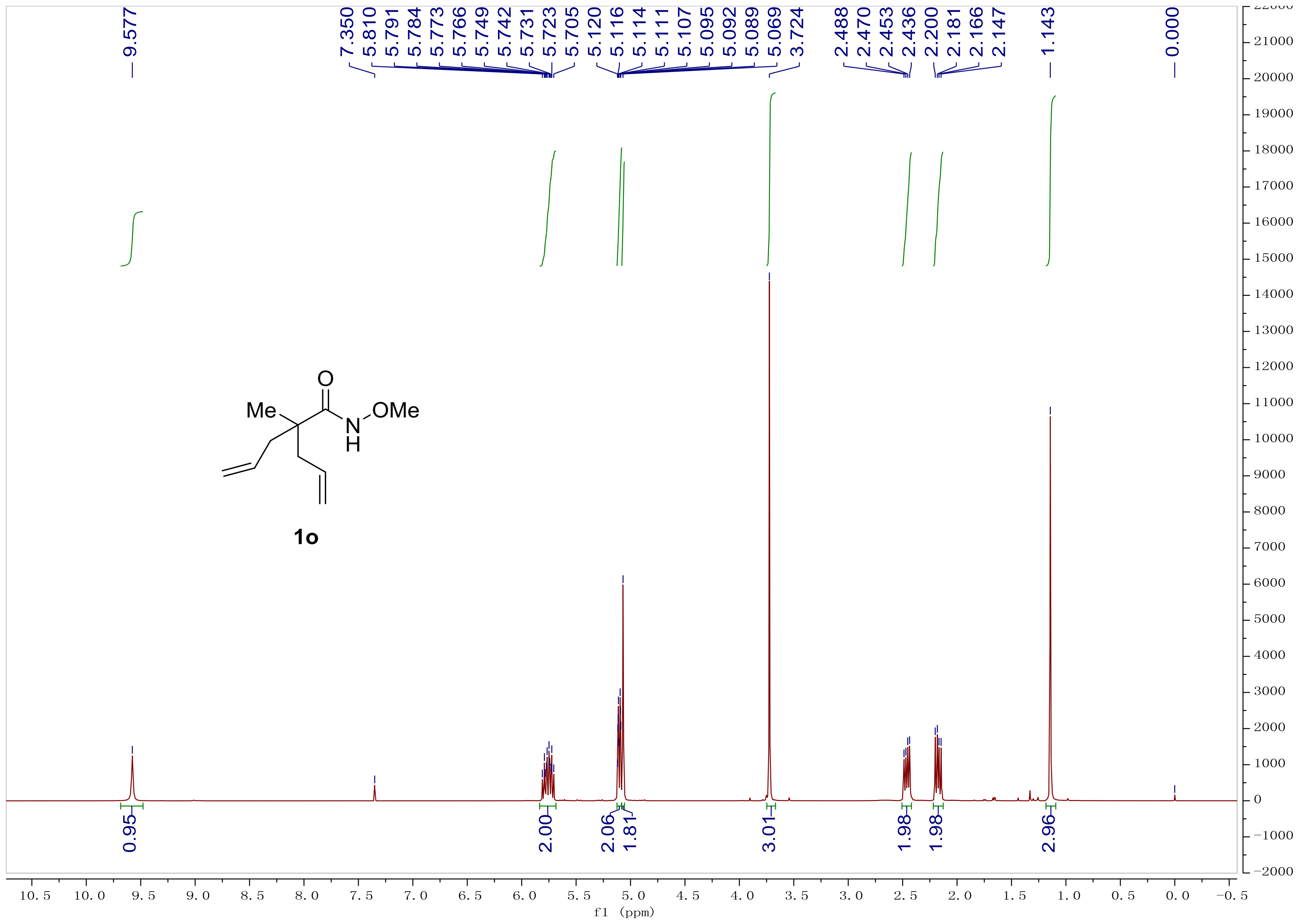

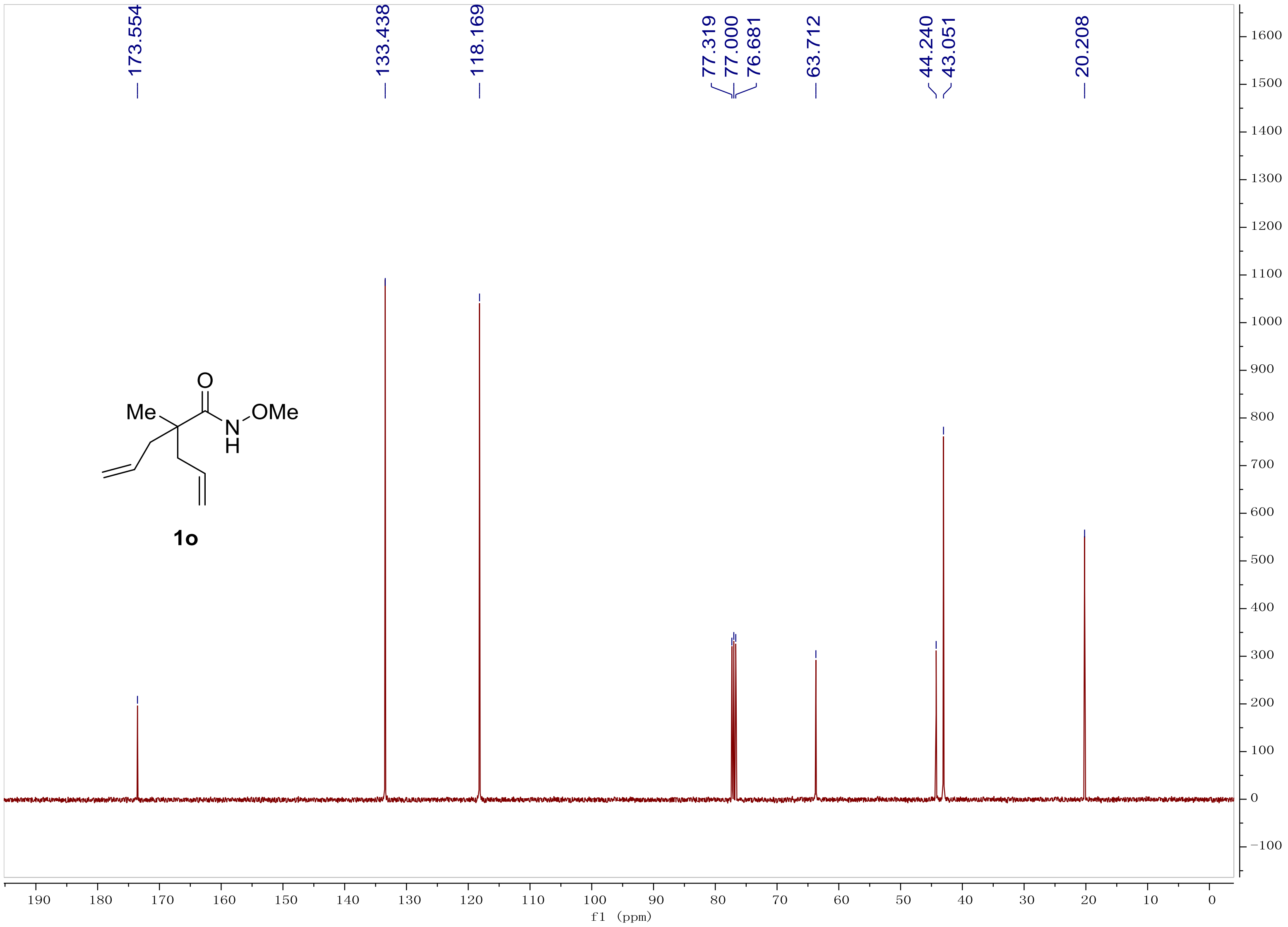

173.554
133.438
118.169
77.319
77.000
76.681
63.712
44.240
43.051
20.208
O
Me
N
OMe
H
1o
1600
1500
1400
1300
1200
1100
1000
900
800
700
600
500
400
300
200
100
0
−100
190
180
170
160
150
140
130
120
110
100
90
80
70
60
50
40
30
20
10
0
f1 (ppm)

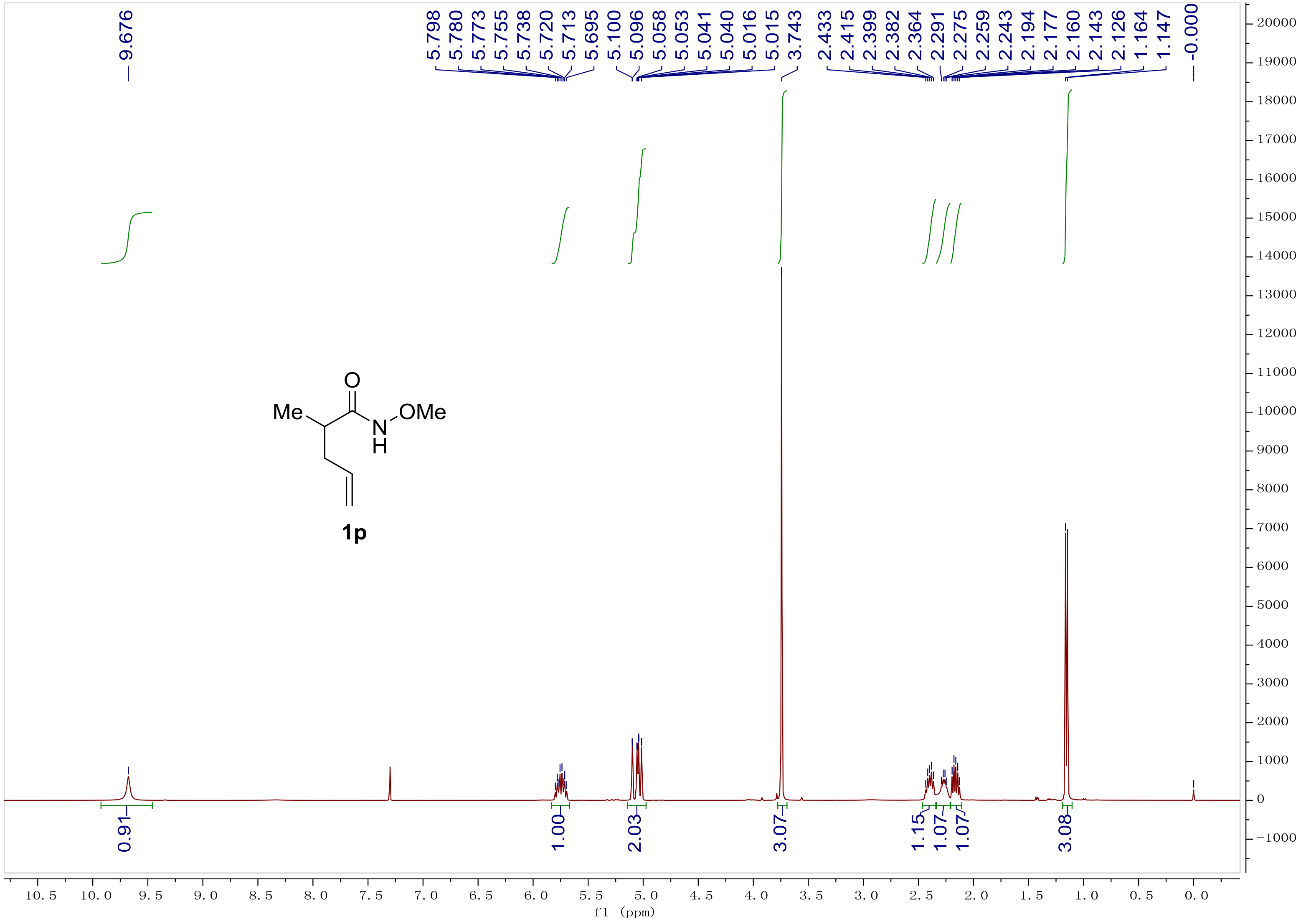
9.676
5.798
5.780
5.773
5.755
5.738
5.720
5.713
5.695
5.100
5.096
5.058
5.053
5.041
5.040
5.016
5.015
3.743
2.433
2.415
2.399
2.382
2.364
2.291
2.275
2.259
2.243
2.194
2.177
2.160
2.143
2.126
1.164
1.147
-0.000
Me
O
N
H
OMe
1p
0.91
1.00
2.03
3.07
1.15
1.07
1.07
3.08
f1 (ppm)

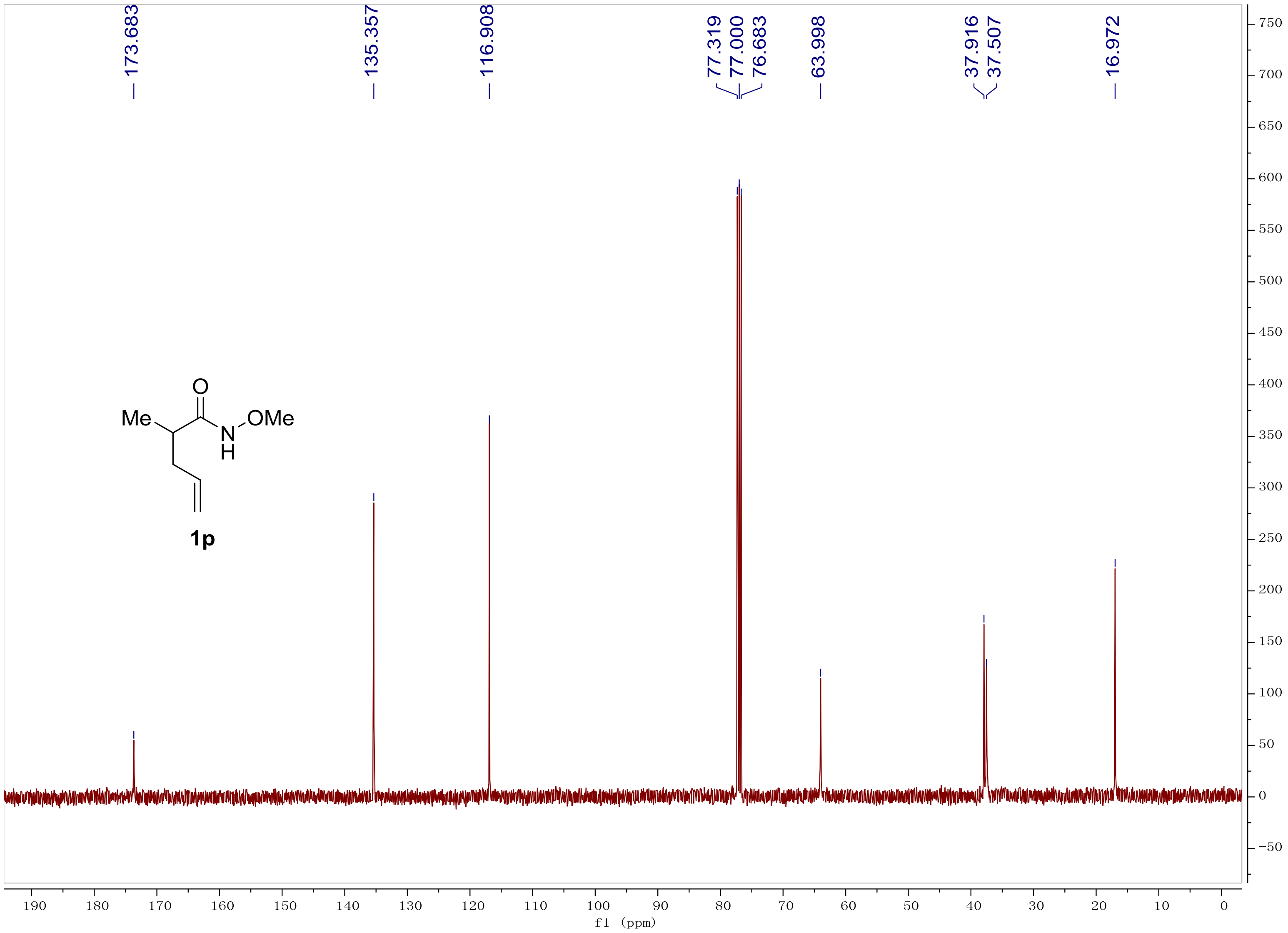
173.683
135.357
116.908
77.319
77.000
76.683
63.998
37.916
37.507
16.972
Me
O
N
H
OMe
1p
f1 (ppm)

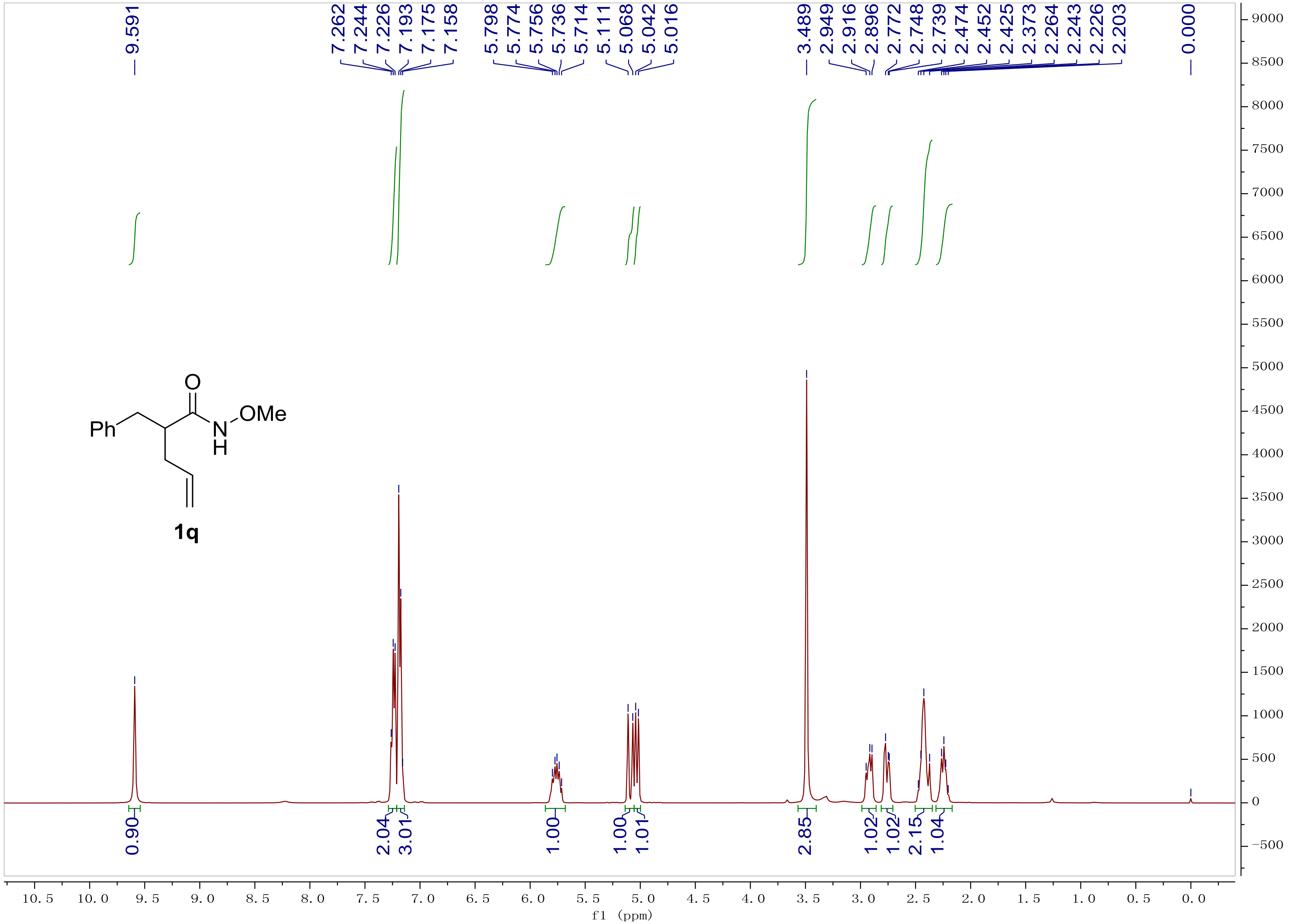

9.591
7.262
7.244
7.226
7.193
7.175
7.158
5.798
5.774
5.756
5.736
5.714
5.111
5.068
5.042
5.016
3.489
2.949
2.916
2.896
2.772
2.748
2.739
2.474
2.452
2.425
2.373
2.264
2.243
2.226
2.203
0.000
Ph
O
N
OMe
H
1q
0.90
2.04
3.01
1.00
1.00
1.01
2.85
1.02
1.02
2.15
1.04
f1 (ppm)

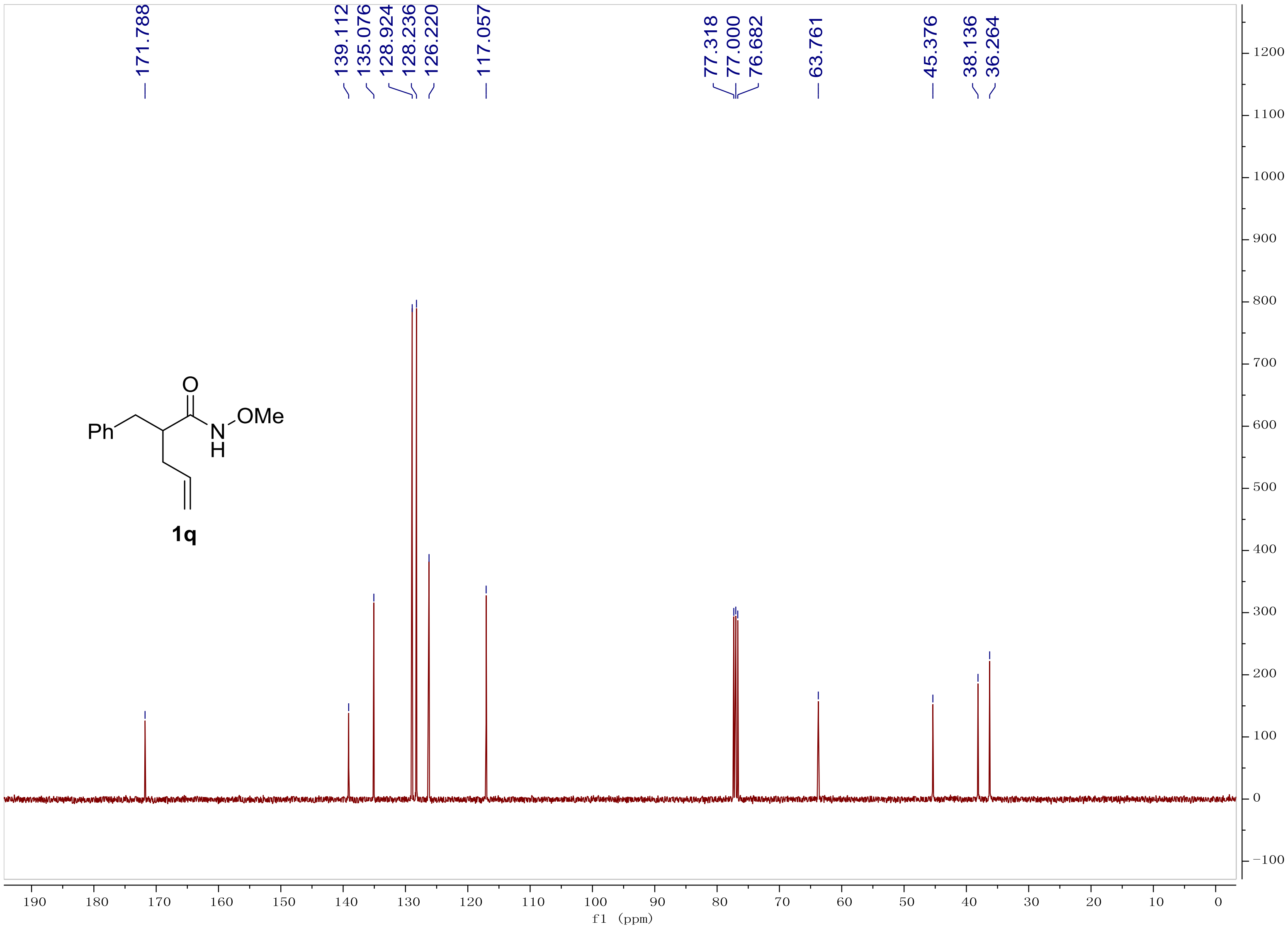
171.788
139.112
135.076
128.924
128.236
126.220
117.057
77.318
77.000
76.682
63.761
45.376
38.136
36.264
Ph
O
N
H
OMe
1q
f1 (ppm)

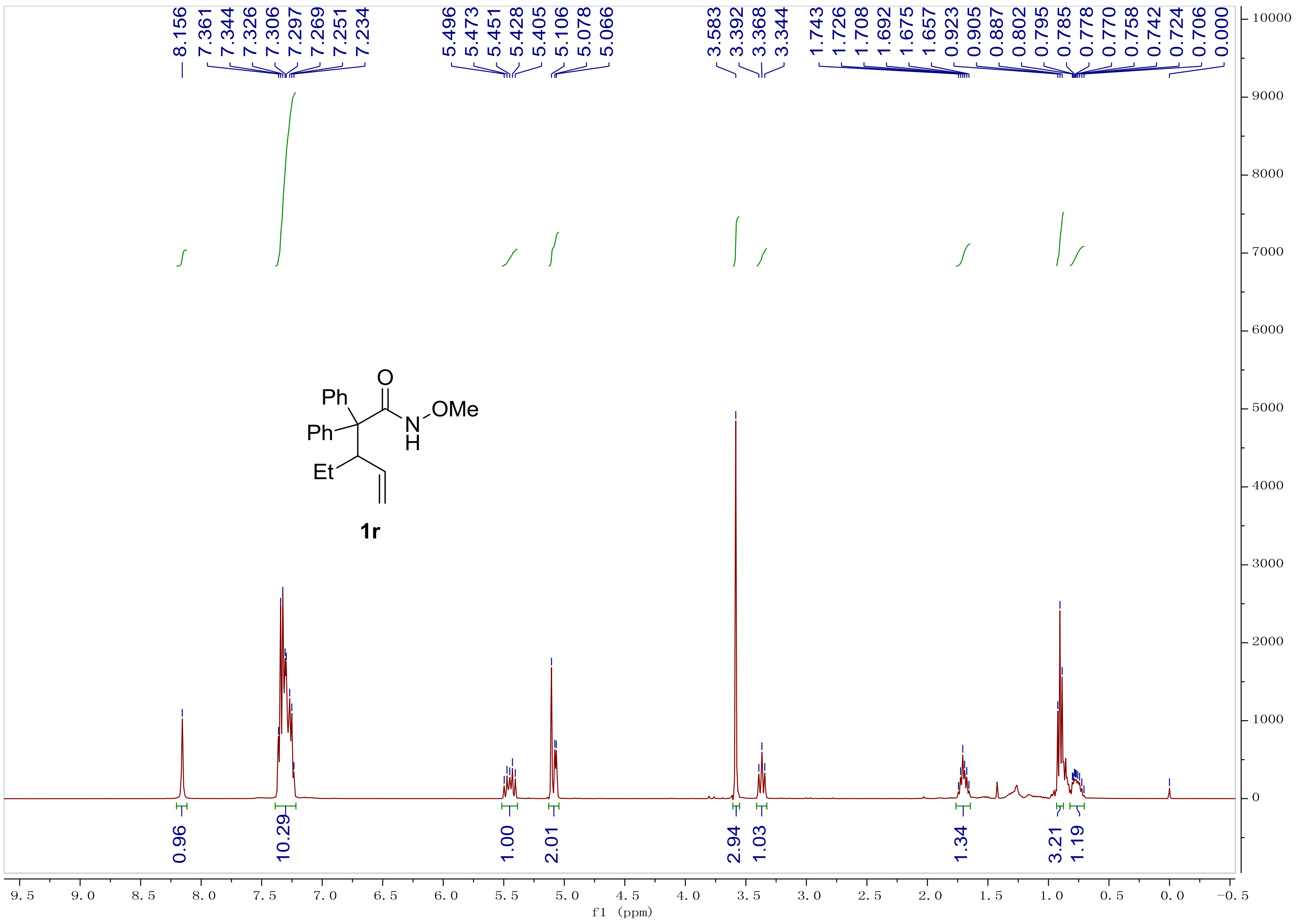

8.156
7.361
7.344
7.326
7.306
7.297
7.269
7.251
7.234
5.496
5.473
5.451
5.428
5.405
5.106
5.078
5.066
3.583
3.392
3.368
3.344
1.743
1.726
1.708
1.692
1.675
1.657
0.923
0.905
0.887
0.802
0.795
0.785
0.778
0.770
0.758
0.742
0.724
0.706
0.000
Ph
Ph
O
OMe
N
H
Et
1r
0.96
10.29
1.00
2.01
2.94
1.03
1.34
3.21
1.19
f1 (ppm)

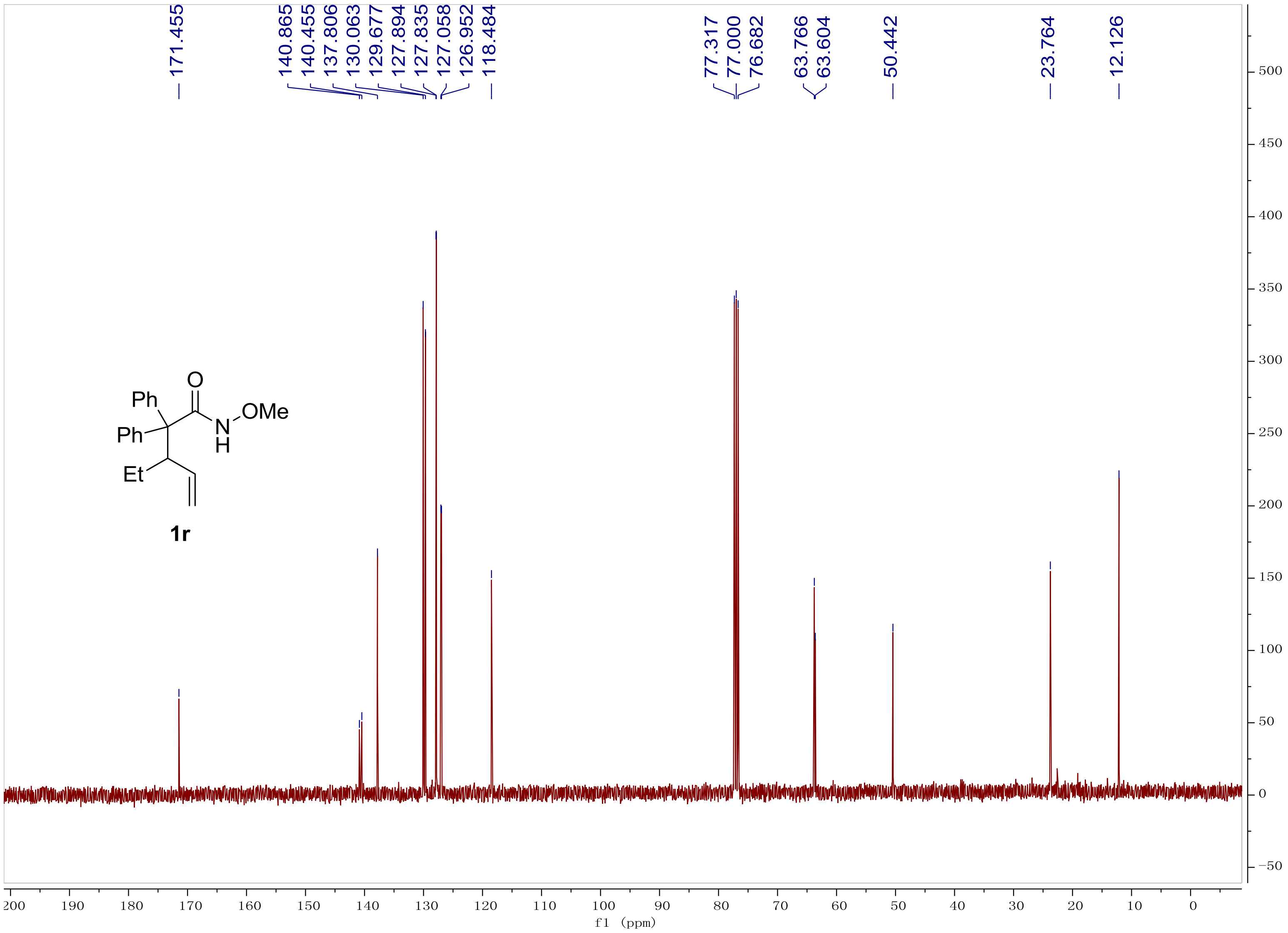
171.455
140.865
140.455
137.806
130.063
129.677
127.894
127.835
127.058
126.952
118.484
77.317
77.000
76.682
63.766
63.604
50.442
23.764
12.126
Ph
Ph
O
N
H
OMe
Et
1r
f1 (ppm)

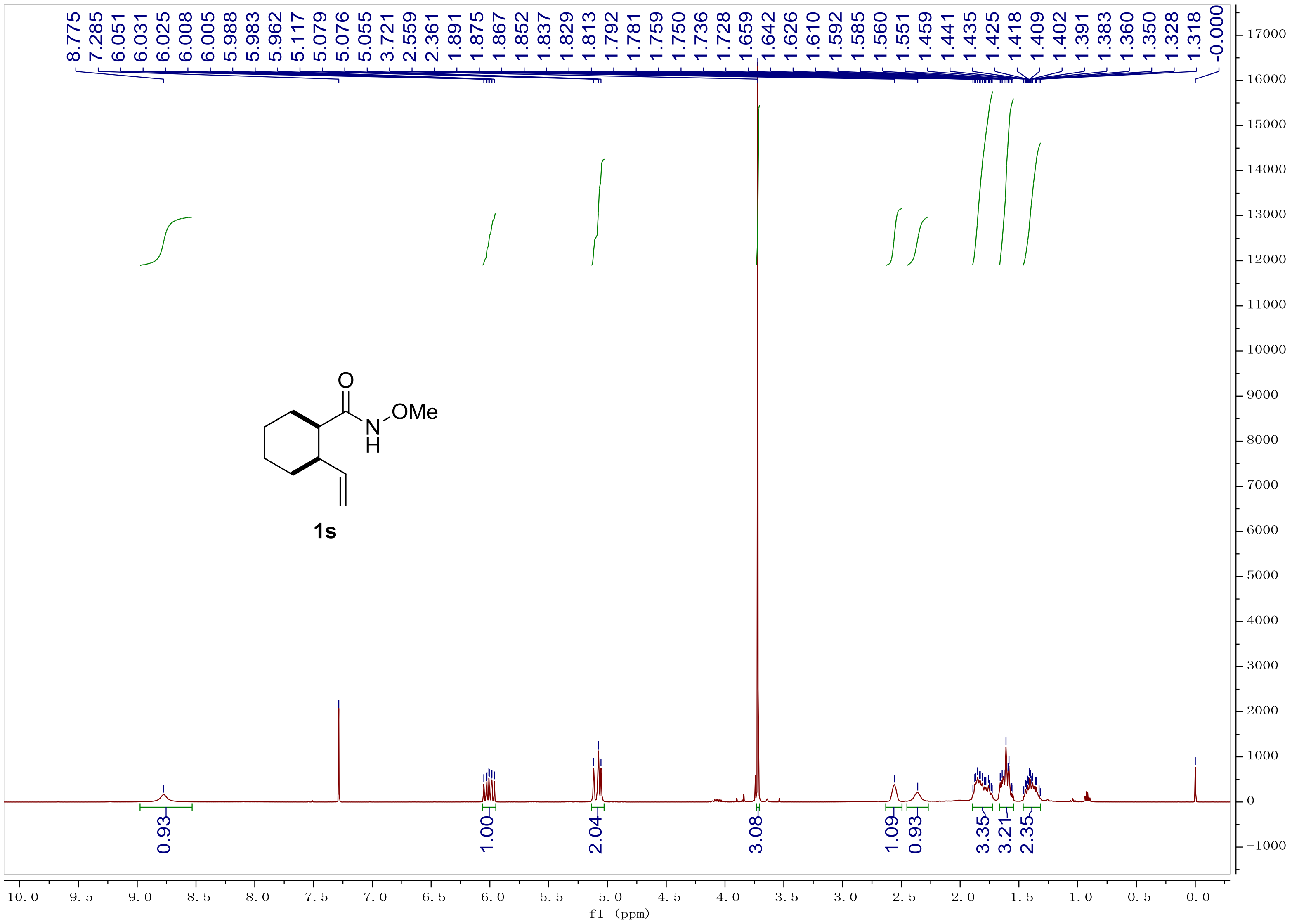

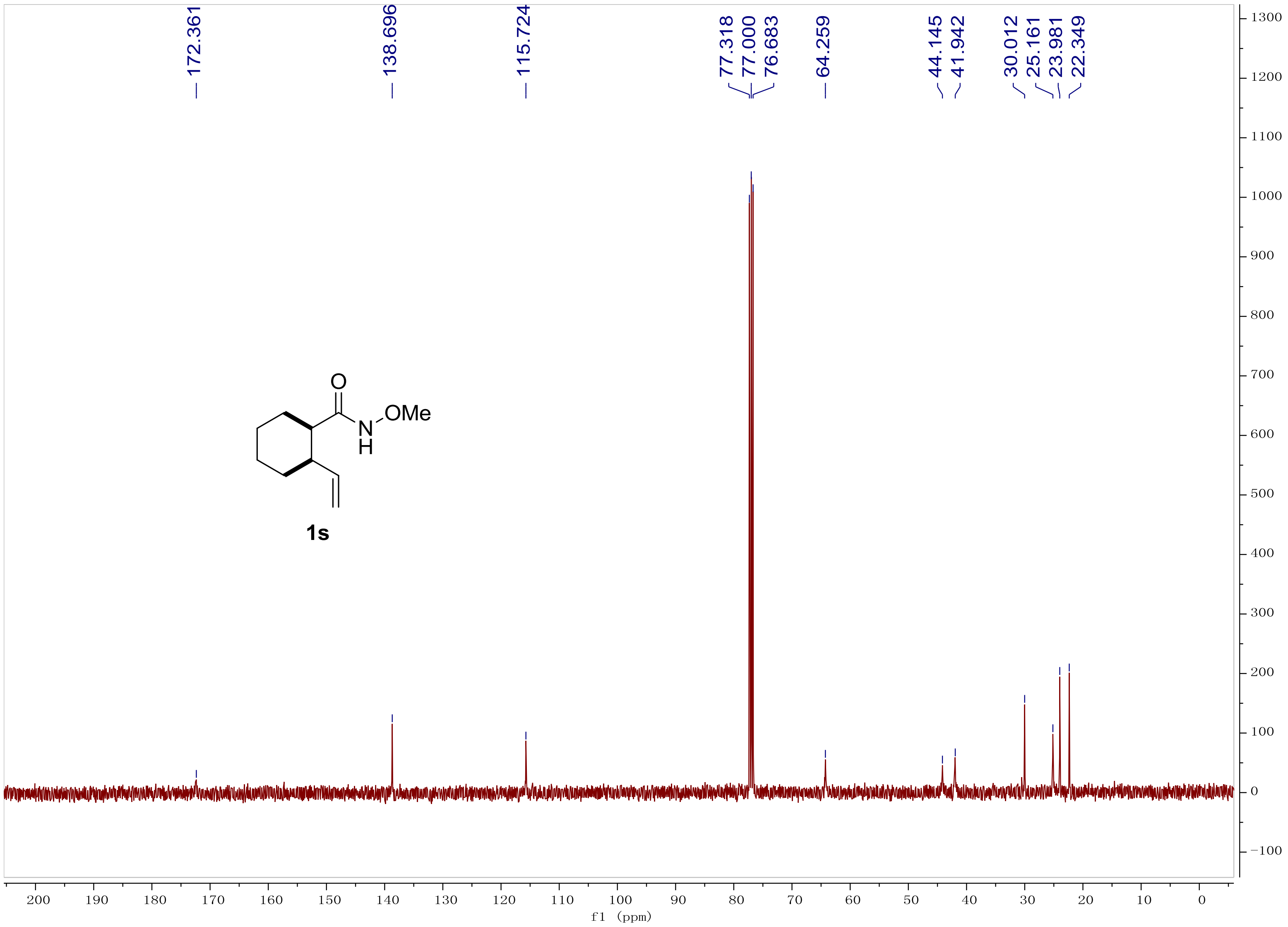
172.361
138.696
115.724
77.318
77.000
76.683
64.259
44.145
41.942
30.012
25.161
23.981
22.349
O
N
H
OMe
1s
f1 (ppm)

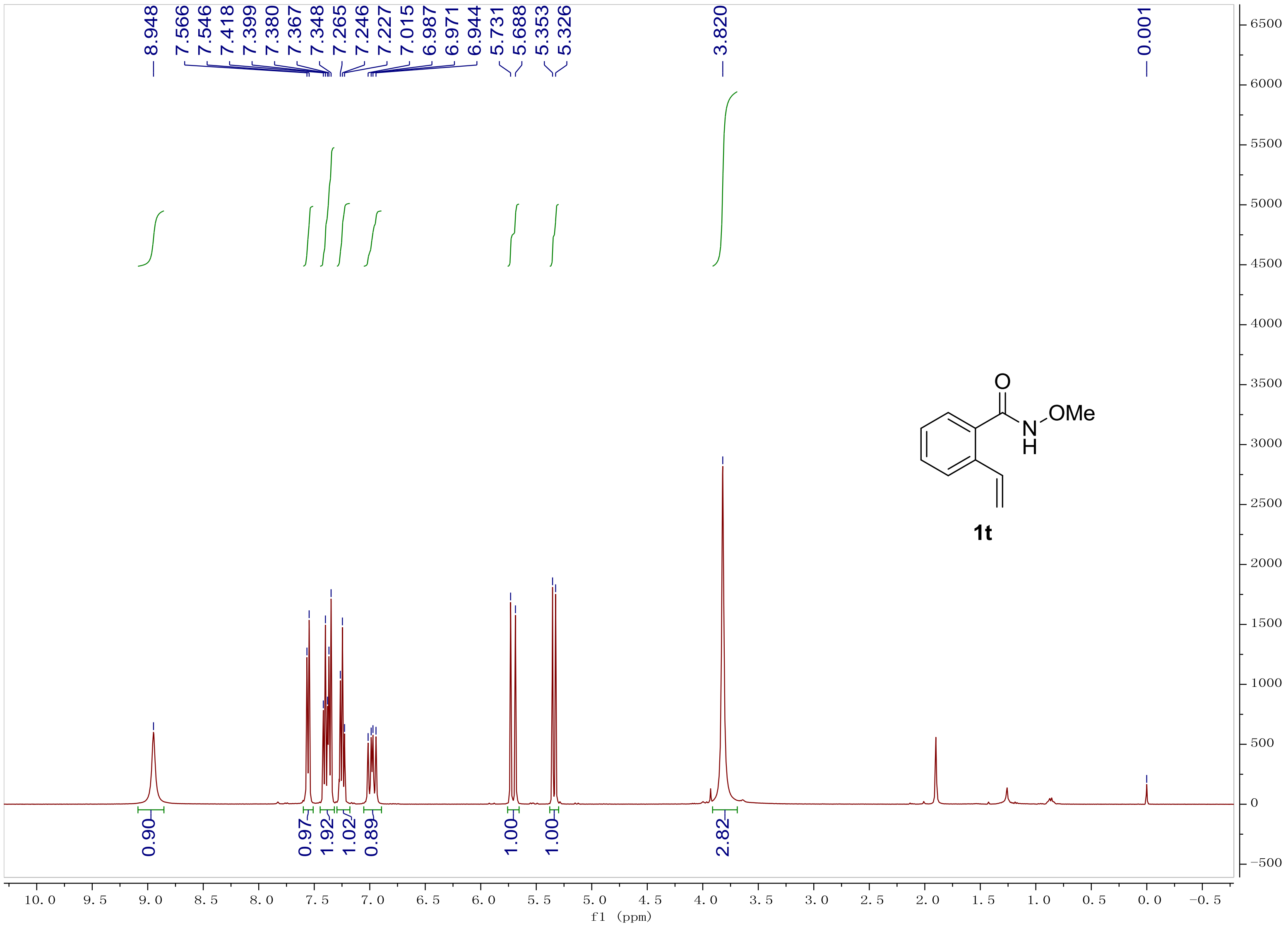

8.948
7.566
7.546
7.418
7.399
7.380
7.367
7.348
7.265
7.246
7.227
7.015
6.987
6.971
6.944
5.731
5.688
5.353
5.326
3.820
0.001
0.90
0.97
1.92
1.02
0.89
1.00
1.00
2.82
O
N
H
OMe
1t
f1 (ppm)

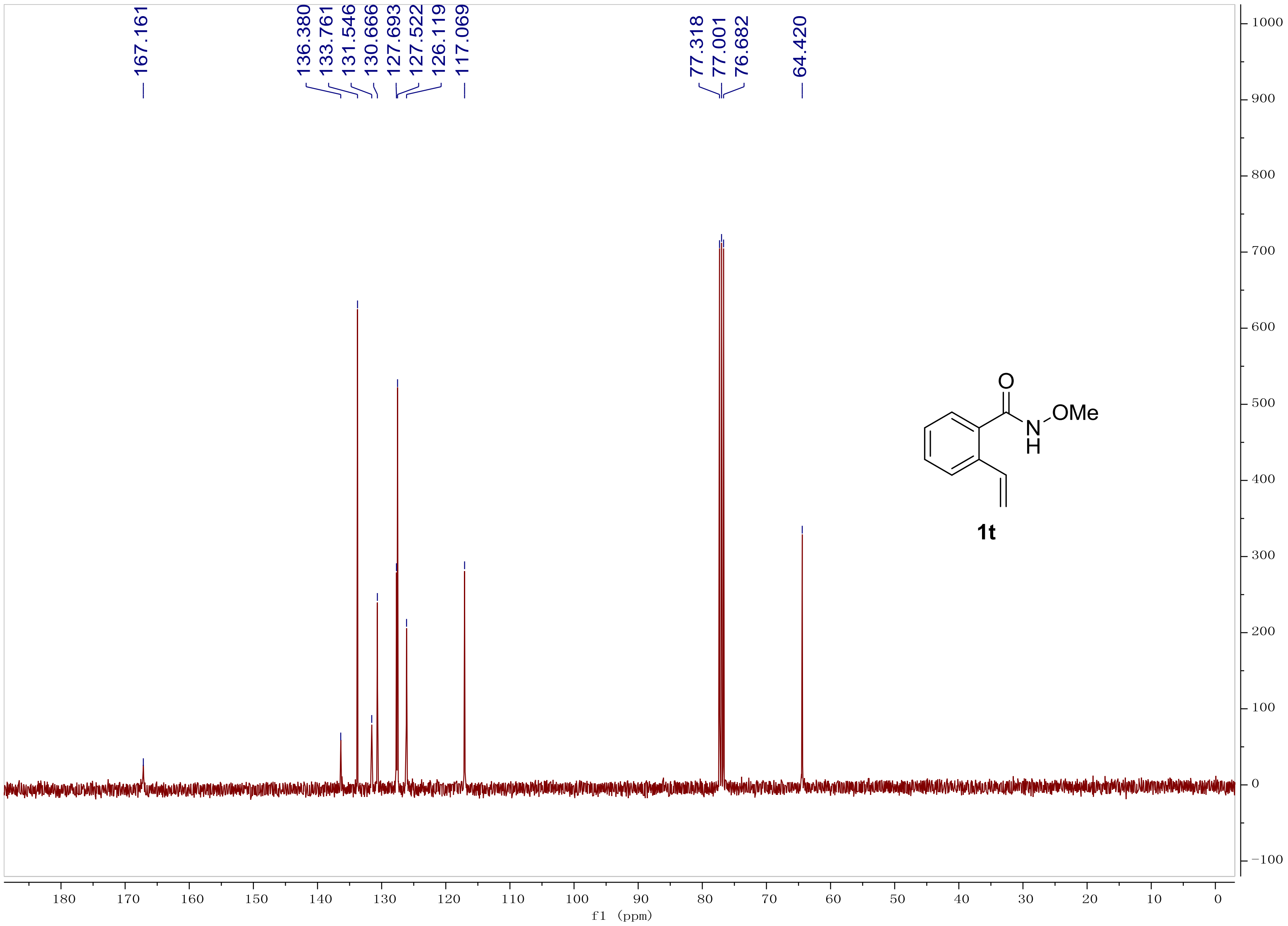
167.161
136.380
133.761
131.546
130.666
127.693
127.522
126.119
117.069
77.318
77.001
76.682
64.420
O
N
H
OMe
1t
f1 (ppm)

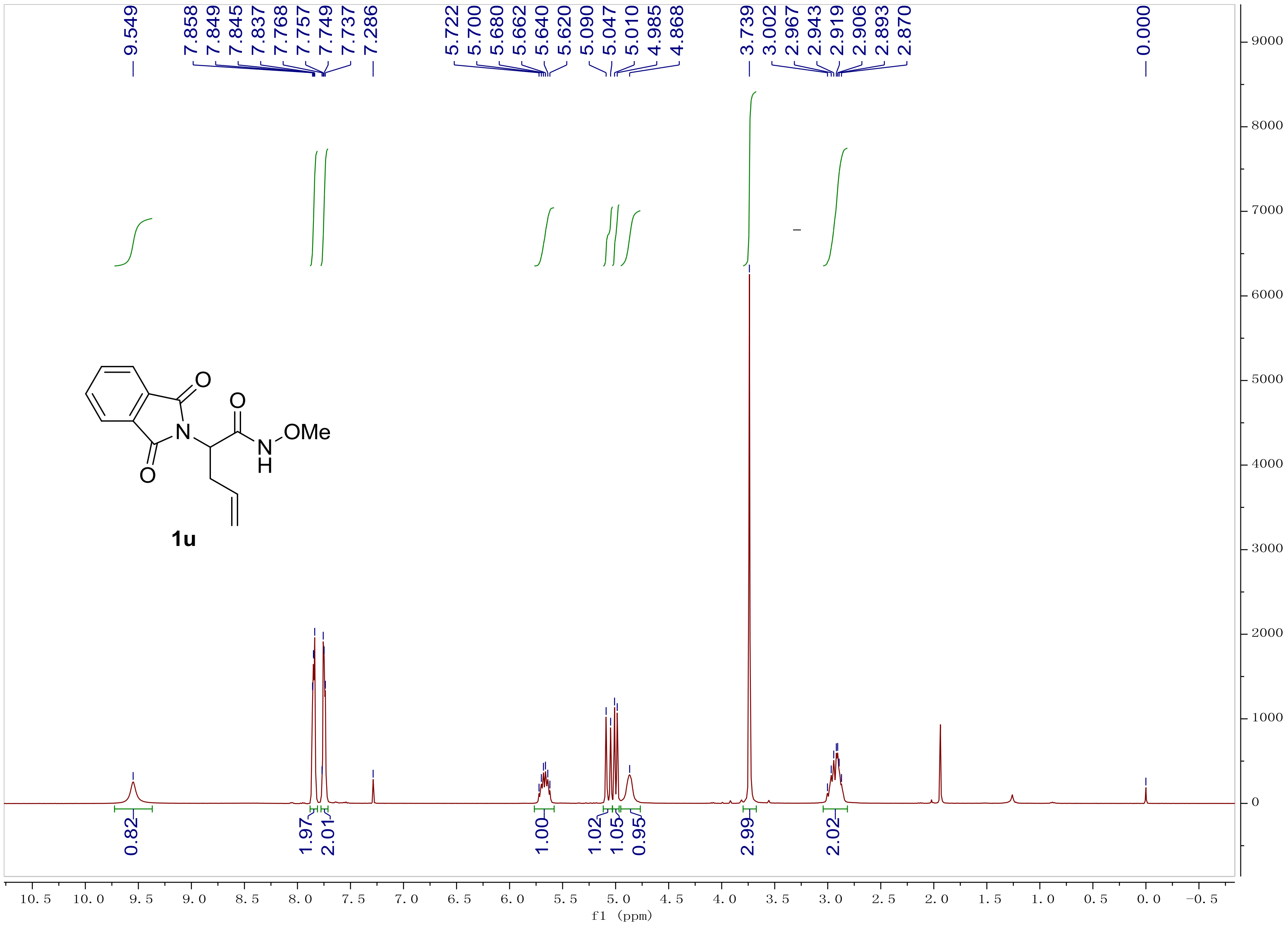
9.549
7.858 7.849 7.845 7.837 7.768 7.757 7.749 7.737 7.286
5.722 5.700 5.680 5.662 5.640 5.620 5.090 5.047 5.010 4.985 4.868
3.739 3.002 2.967 2.943 2.919 2.906 2.893 2.870
0.000
0.82
1.97
2.01
1.00
1.02
1.05
0.95
2.99
2.02
O
N
OMe
H
1u
f1 (ppm)

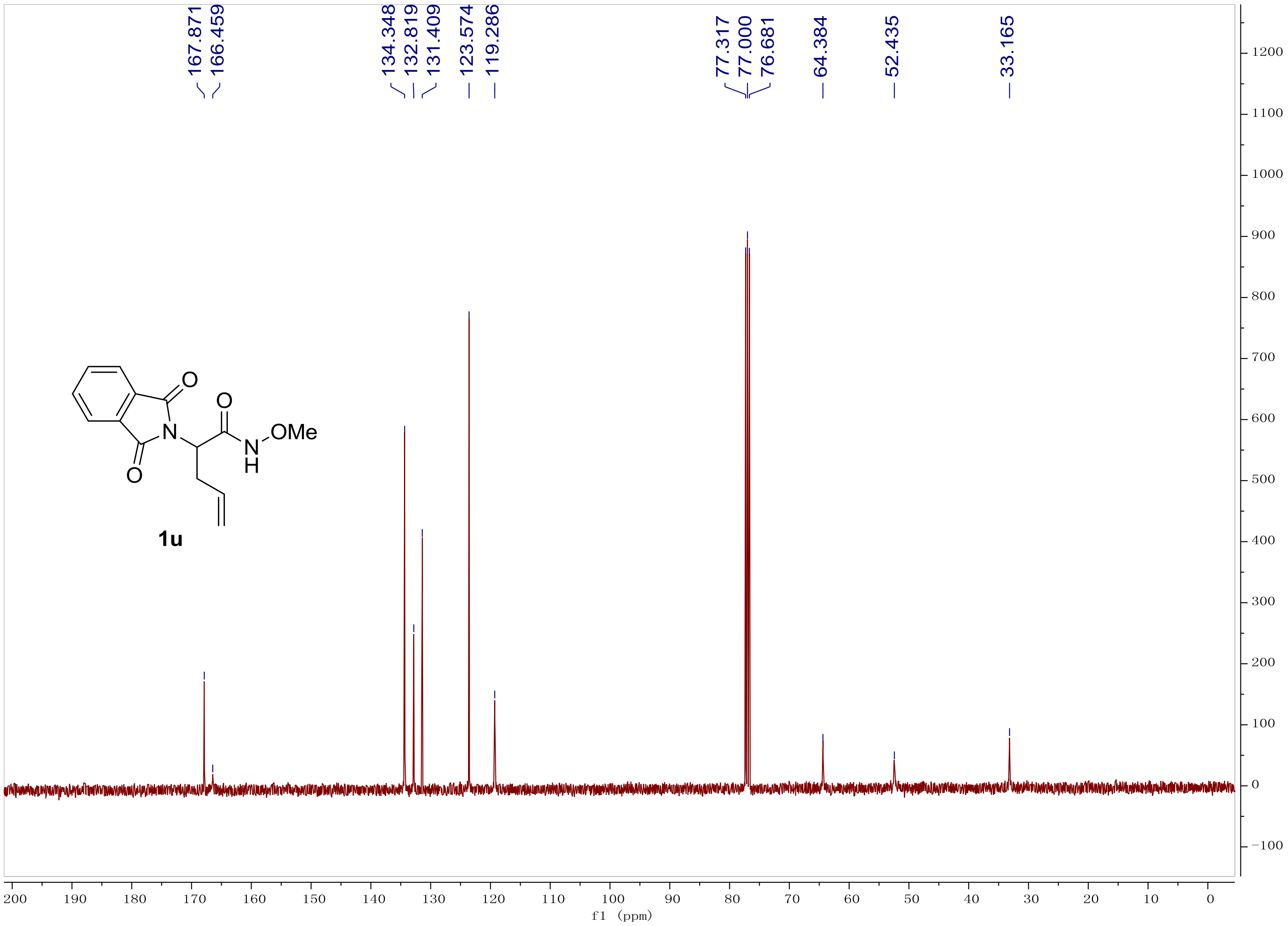
167.871
166.459
134.348
132.819
131.409
123.574
119.286
77.317
77.000
76.681
64.384
52.435
33.165
O
N
OMe
N
H
1u
200 190 180 170 160 150 140 130 120 110 100 90 80 70 60 50 40 30 20 10 0
f1 (ppm)
1200 1100 1000 900 800 700 600 500 400 300 200 100 0 −100

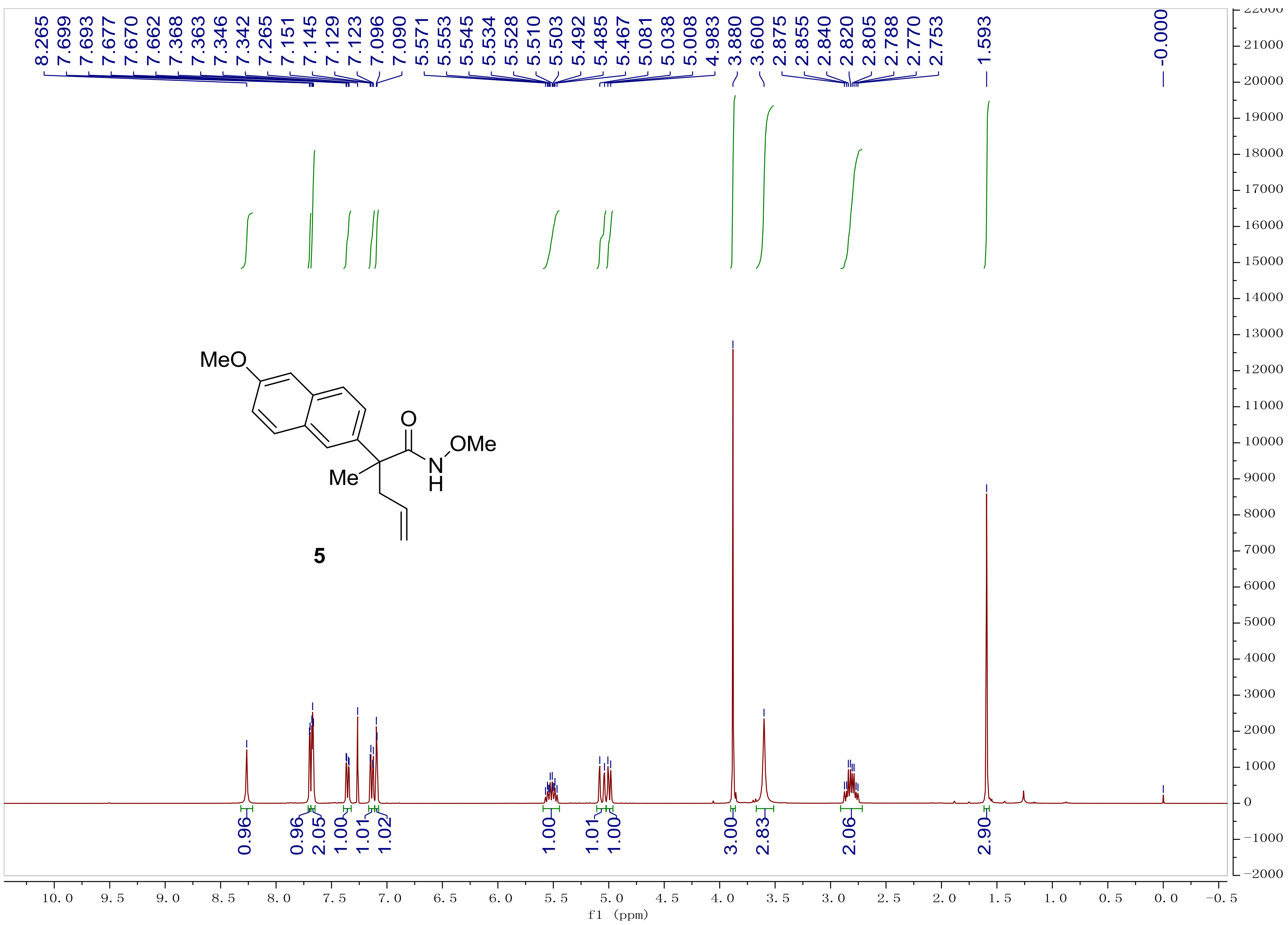

8.265
7.699
7.693
7.677
7.670
7.662
7.368
7.363
7.346
7.342
7.265
7.151
7.145
7.129
7.123
7.096
7.090
5.571
5.553
5.545
5.534
5.528
5.510
5.503
5.492
5.485
5.467
5.081
5.038
5.008
4.983
3.880
3.600
2.875
2.855
2.840
2.820
2.805
2.788
2.770
2.753
1.593
-0.000
MeO
O
OMe
N
H
Me
5
0.96
0.96
2.05
1.00
1.01
1.02
1.00
1.01
1.00
3.00
2.83
2.06
2.90
f1 (ppm)

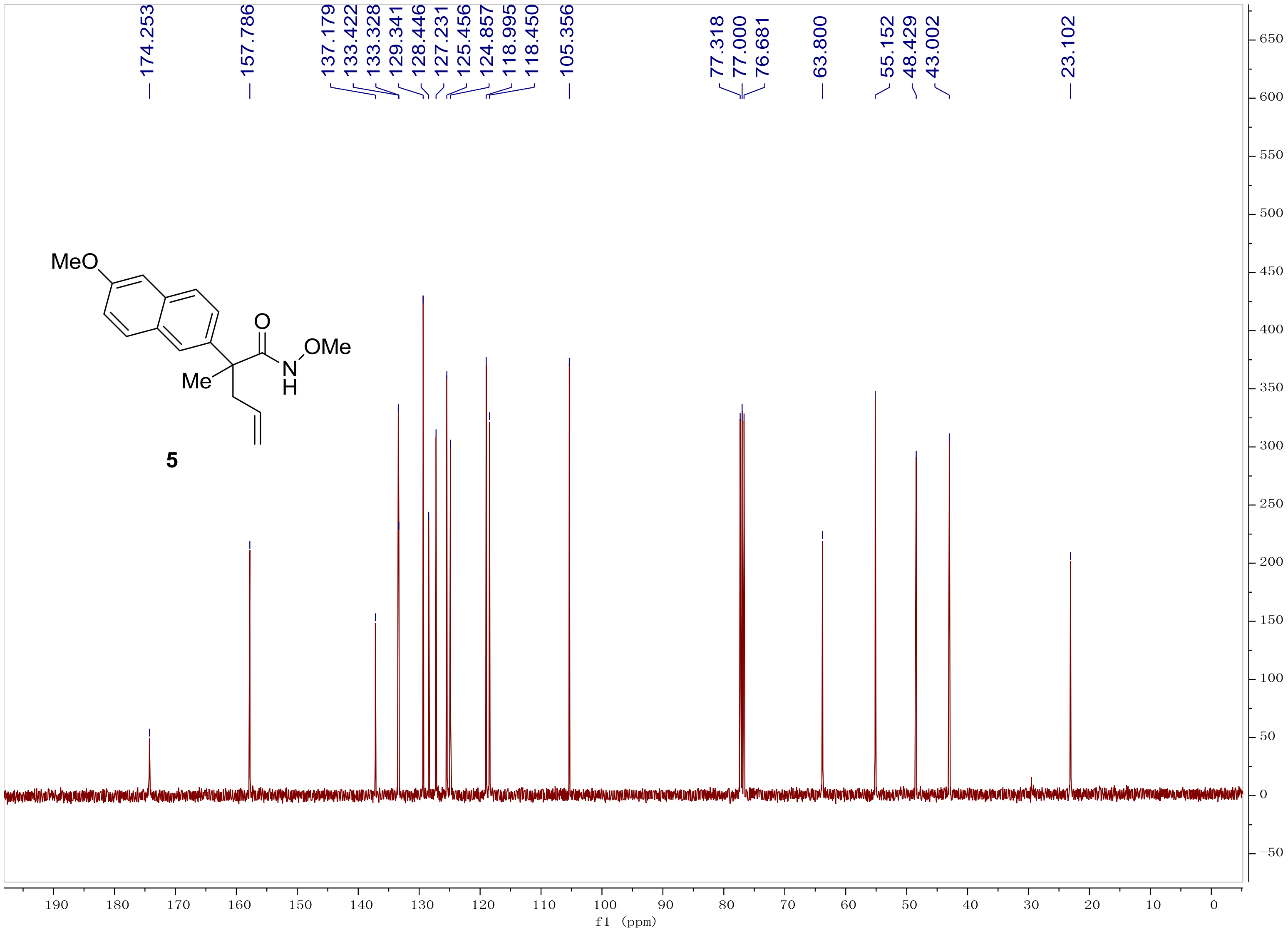
174.253
157.786
137.179
133.422
133.328
129.341
128.446
127.231
125.456
124.857
118.995
118.450
105.356
77.318
77.000
76.681
63.800
55.152
48.429
43.002
23.102
MeO
O
OMe
N
H
Me
5
f1 (ppm)

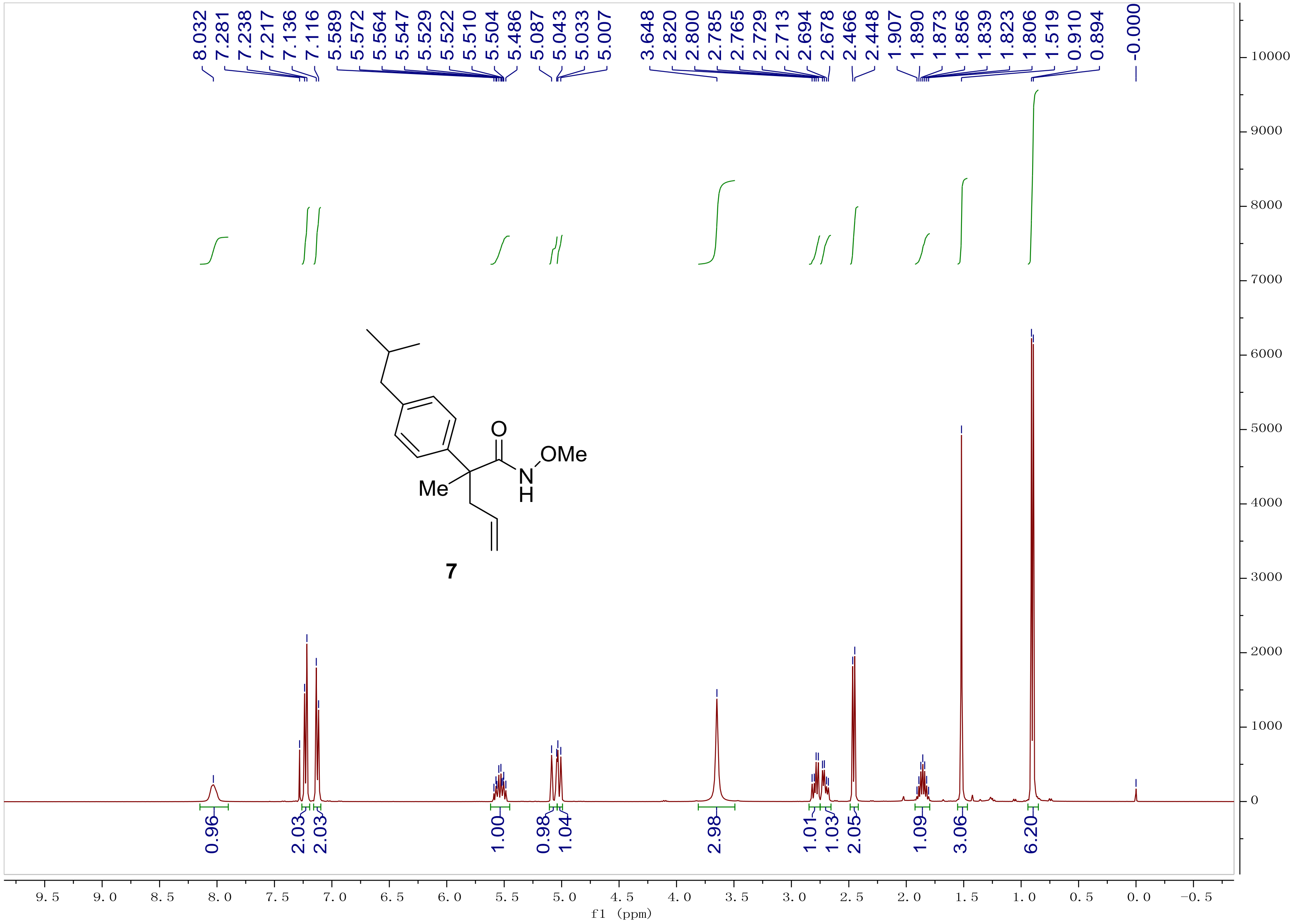
8.032
7.281
7.238
7.217
7.136
7.116
5.589
5.572
5.564
5.547
5.529
5.522
5.510
5.504
5.486
5.087
5.043
5.033
5.007
3.648
2.820
2.800
2.785
2.765
2.729
2.713
2.694
2.678
2.466
2.448
1.907
1.890
1.873
1.856
1.839
1.823
1.806
1.519
0.910
0.894
-0.000
O
OMe
N
H
Me
7
0.96
2.03
2.03
1.00
0.98
1.04
2.98
1.01
1.03
2.05
1.09
3.06
6.20
f1 (ppm)

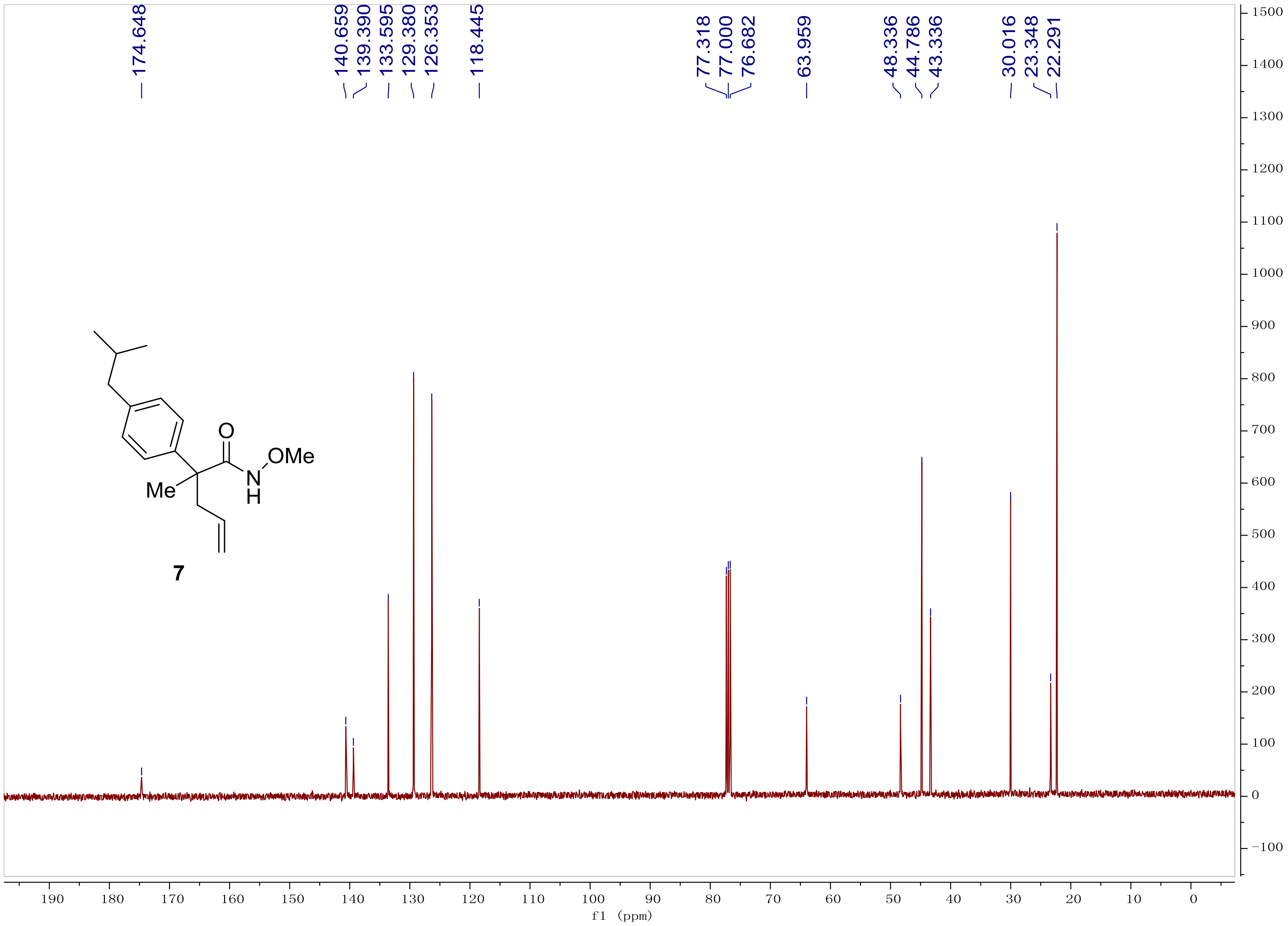
174.648
140.659
139.390
133.595
129.380
126.353
118.445
77.318
77.000
76.682
63.959
48.336
44.786
43.336
30.016
23.348
22.291
O
OMe
N
H
Me
7
f1 (ppm)

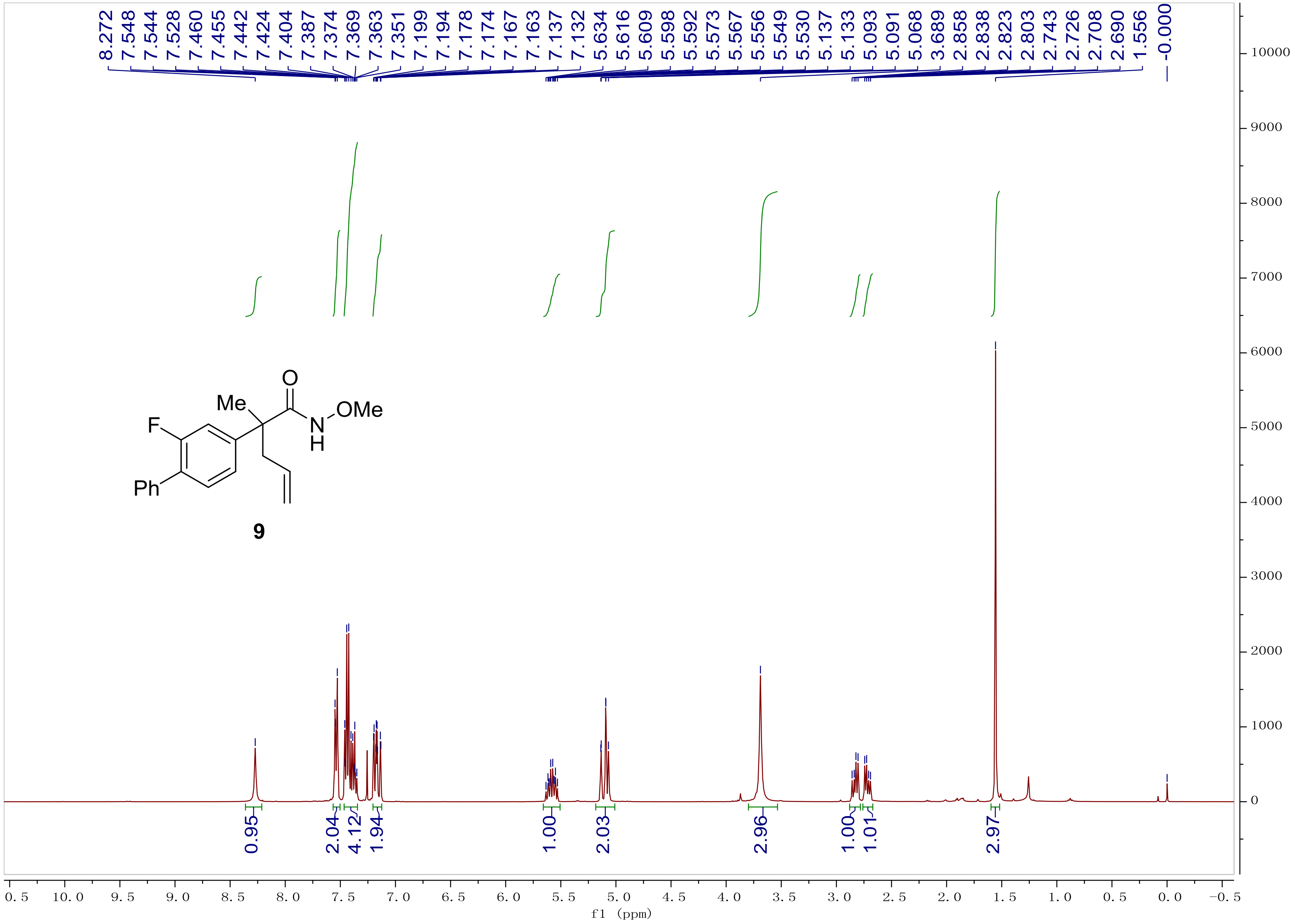
8.272 7.548 7.544 7.528 7.460 7.455 7.442 7.424 7.404 7.387 7.374 7.369 7.363 7.351 7.199 7.194 7.178 7.174 7.167 7.163 7.137 7.132 5.634 5.616 5.609 5.598 5.592 5.573 5.567 5.556 5.549 5.530 5.137 5.133 5.093 5.091 5.068 3.689 2.858 2.838 2.823 2.803 2.743 2.726 2.708 2.690 1.556 -0.000
0.95 2.04 4.12 1.94 1.00 2.03 2.96 1.00 1.01 2.97
O
Me
N
OMe
H
F
Ph
9
f1 (ppm)

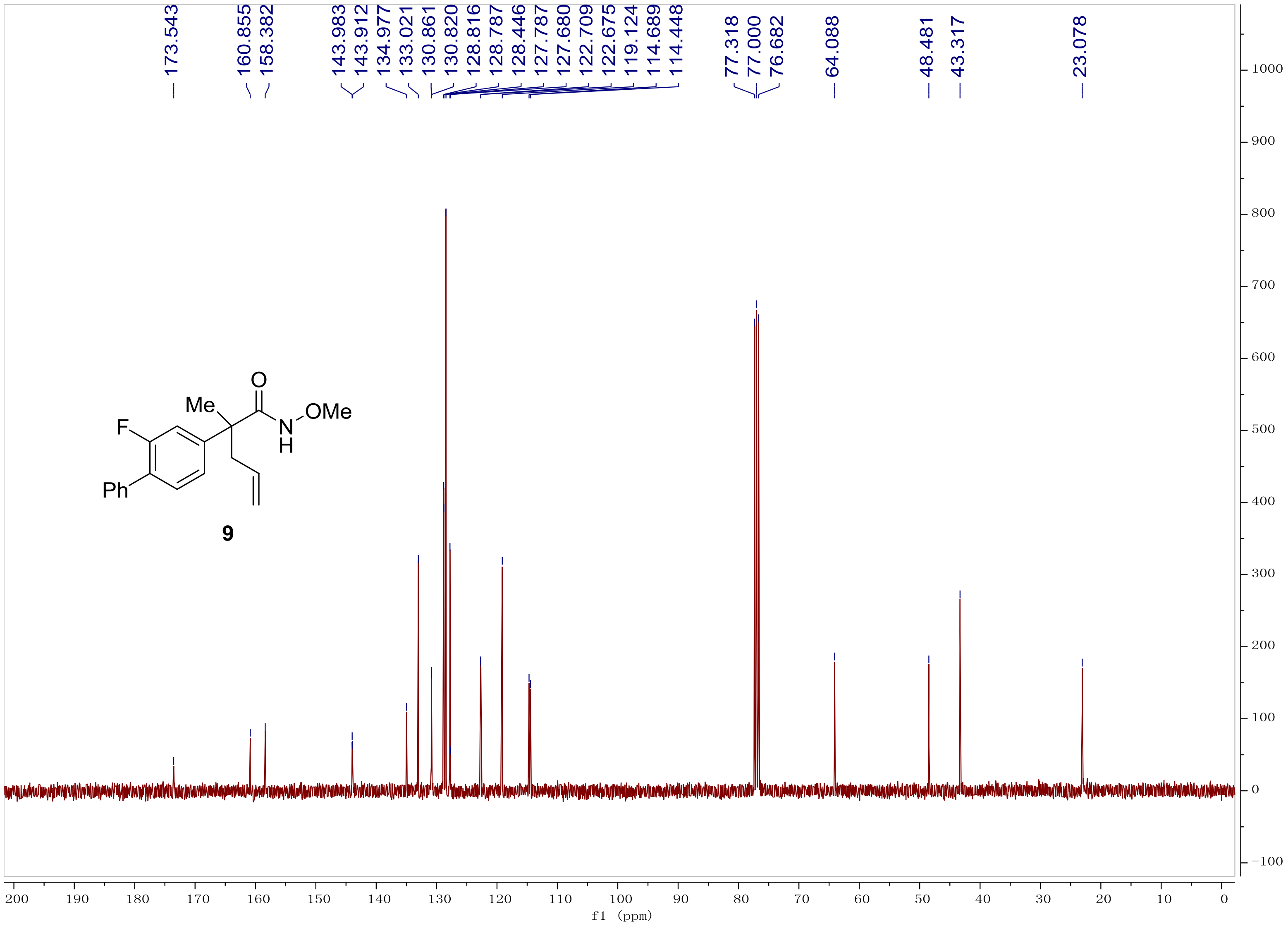

173.543
160.855
158.382
143.983
143.912
134.977
133.021
130.861
130.820
128.816
128.787
128.446
127.787
127.680
122.709
122.675
119.124
114.689
114.448
77.318
77.000
76.682
64.088
48.481
43.317
23.078
F
Ph
Me
O
N
H
OMe
9
f1 (ppm)

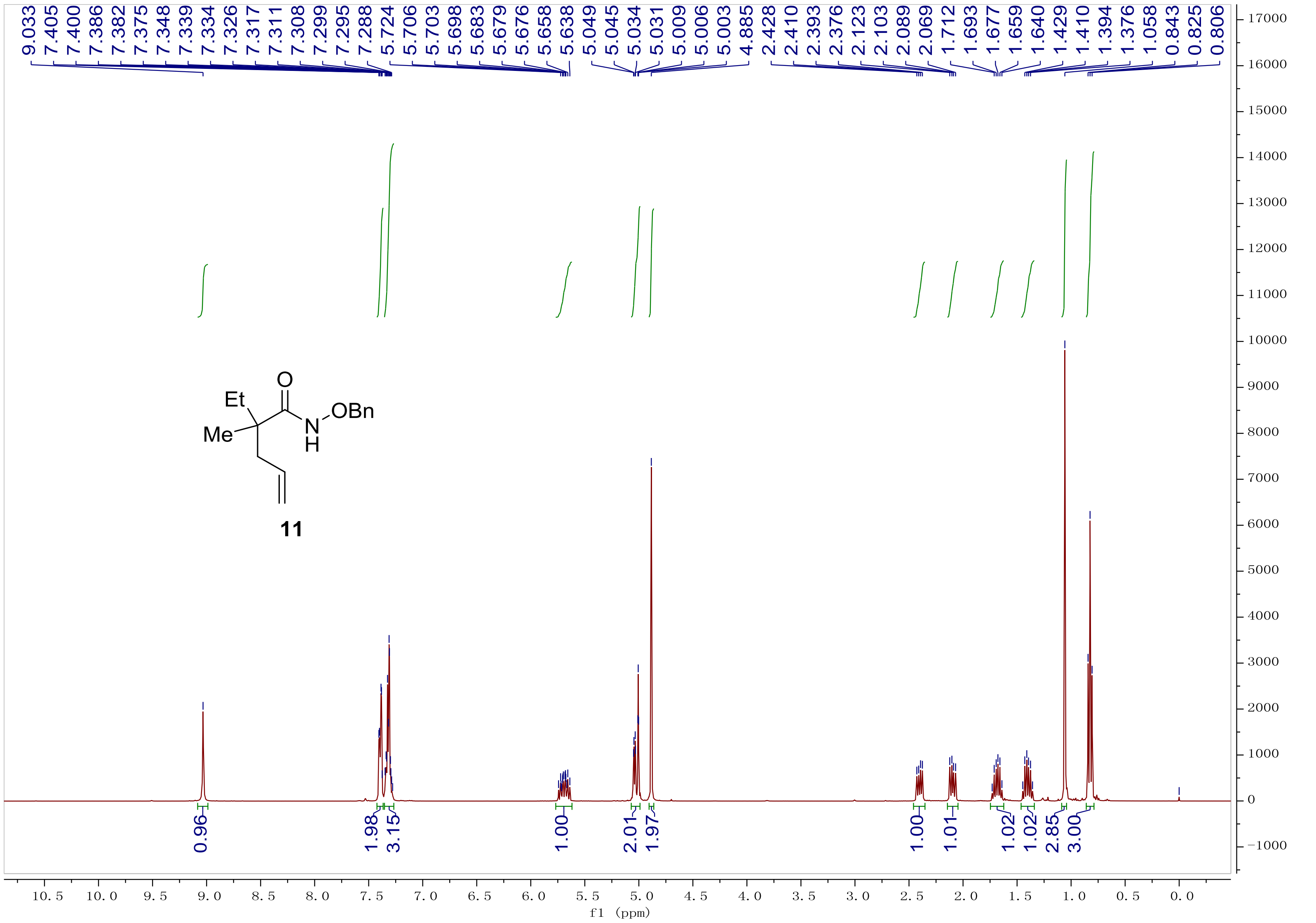
9.033
7.405
7.400
7.386
7.382
7.375
7.348
7.339
7.334
7.326
7.317
7.311
7.308
7.299
7.295
7.288
5.724
5.706
5.703
5.698
5.683
5.679
5.676
5.658
5.638
5.049
5.045
5.034
5.031
5.009
5.006
5.003
4.885
2.428
2.410
2.393
2.376
2.123
2.103
2.089
2.069
1.712
1.693
1.677
1.659
1.640
1.429
1.410
1.394
1.376
1.058
0.843
0.825
0.806
O
Et
N
OBn
Me
H
0.96
1.98
3.15
1.00
2.01
1.97
1.00
1.01
1.02
1.02
2.85
3.00
f1 (ppm)

11

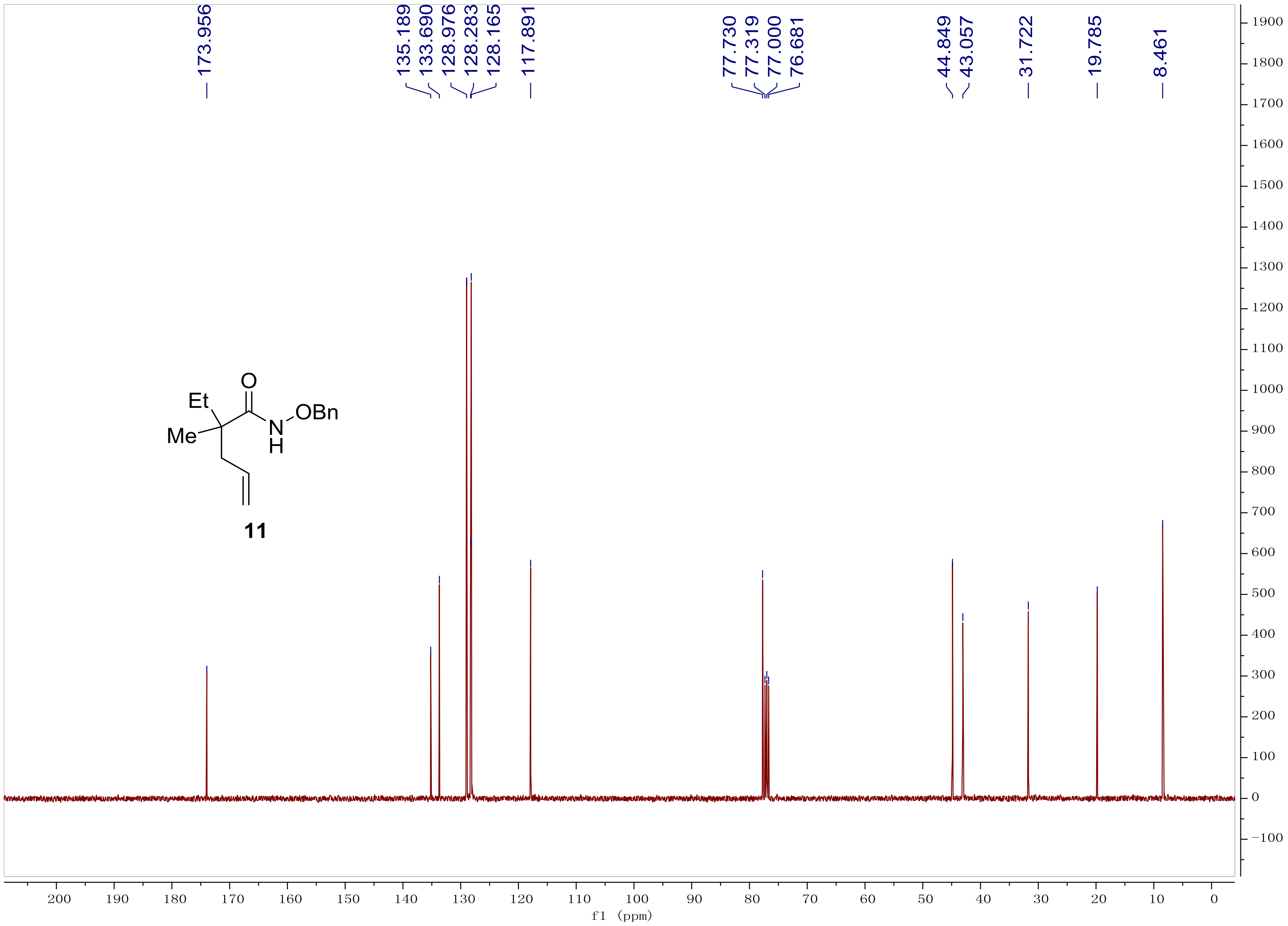

173.956
135.189
133.690
128.976
128.283
128.165
117.891
77.730
77.319
77.000
76.681
44.849
43.057
31.722
19.785
8.461
Et
Me
O
N
H
OBn
f1 (ppm)

11

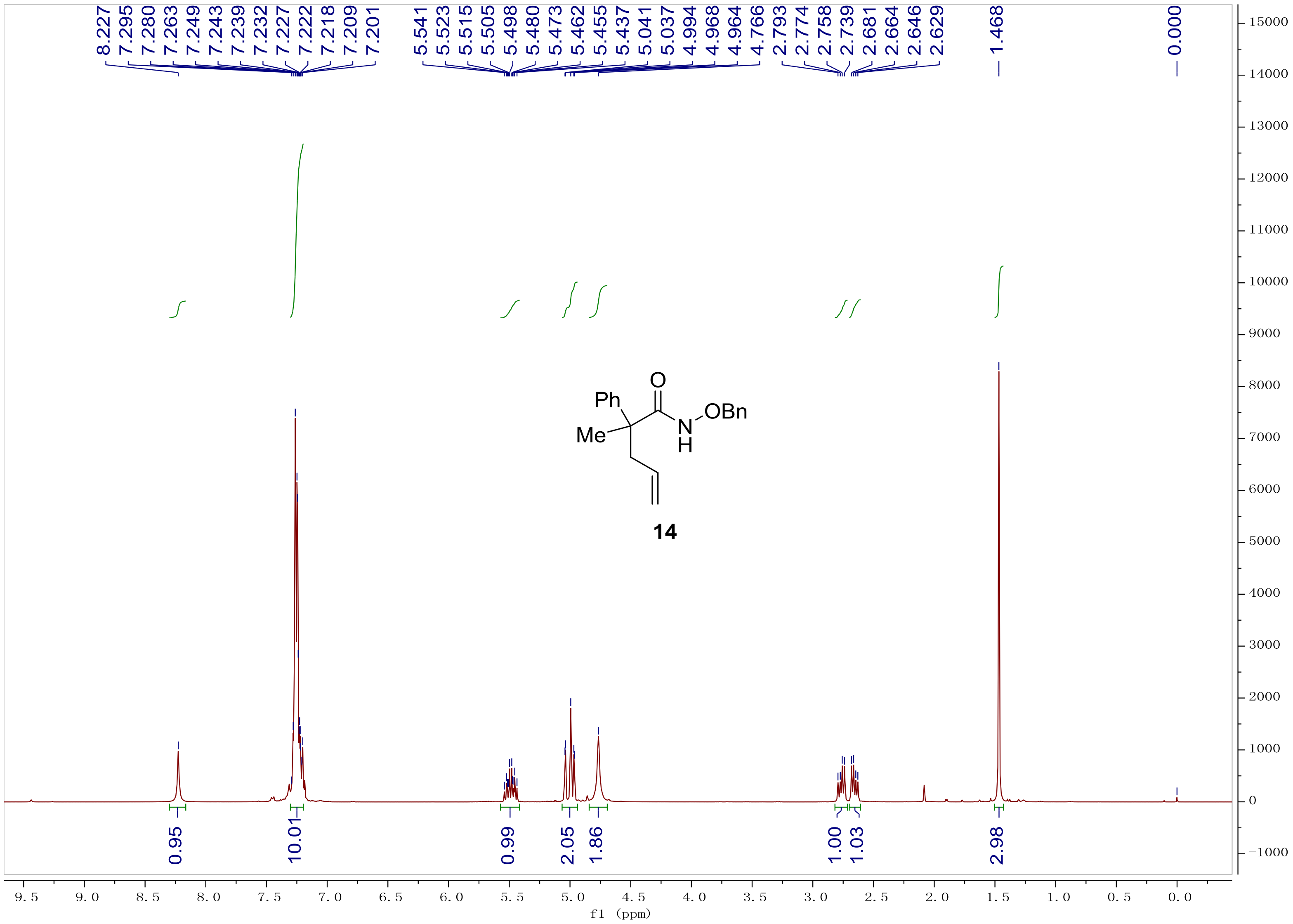
8.227
7.295
7.280
7.263
7.249
7.243
7.239
7.232
7.227
7.222
7.218
7.209
7.201
5.541
5.523
5.515
5.505
5.498
5.480
5.473
5.462
5.455
5.437
5.041
5.037
4.994
4.968
4.964
4.766
2.793
2.774
2.758
2.739
2.681
2.664
2.646
2.629
1.468
0.000
Ph
Me
O
N
H
OBn
14
0.95
10.01
0.99
2.05
1.86
1.00
1.03
2.98
f1 (ppm)

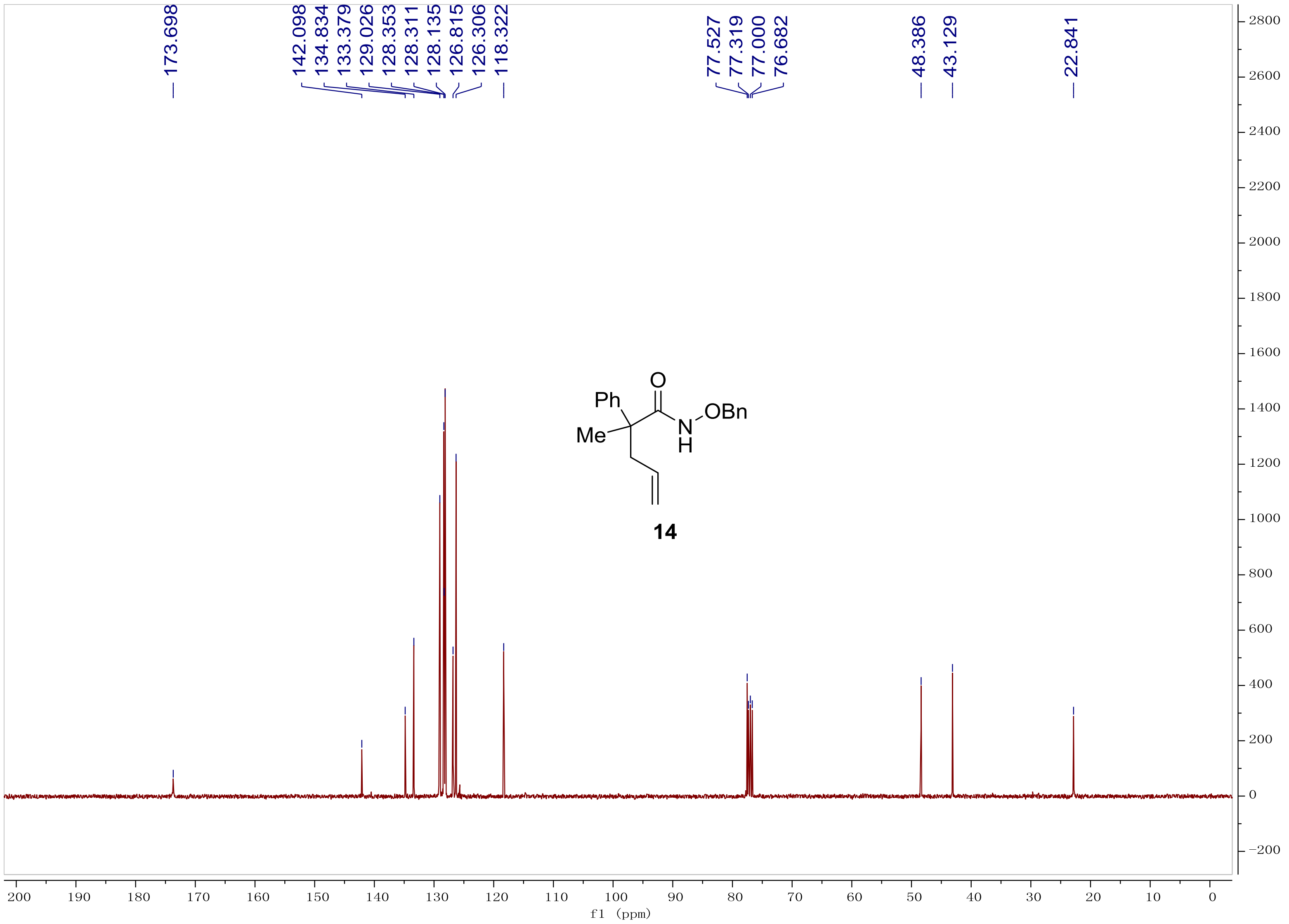
173.698
142.098
134.834
133.379
129.026
128.353
128.311
128.135
126.815
126.306
118.322
77.527
77.319
77.000
76.682
48.386
43.129
22.841
Ph
Me
O
N
H
OBn
14
f1 (ppm)

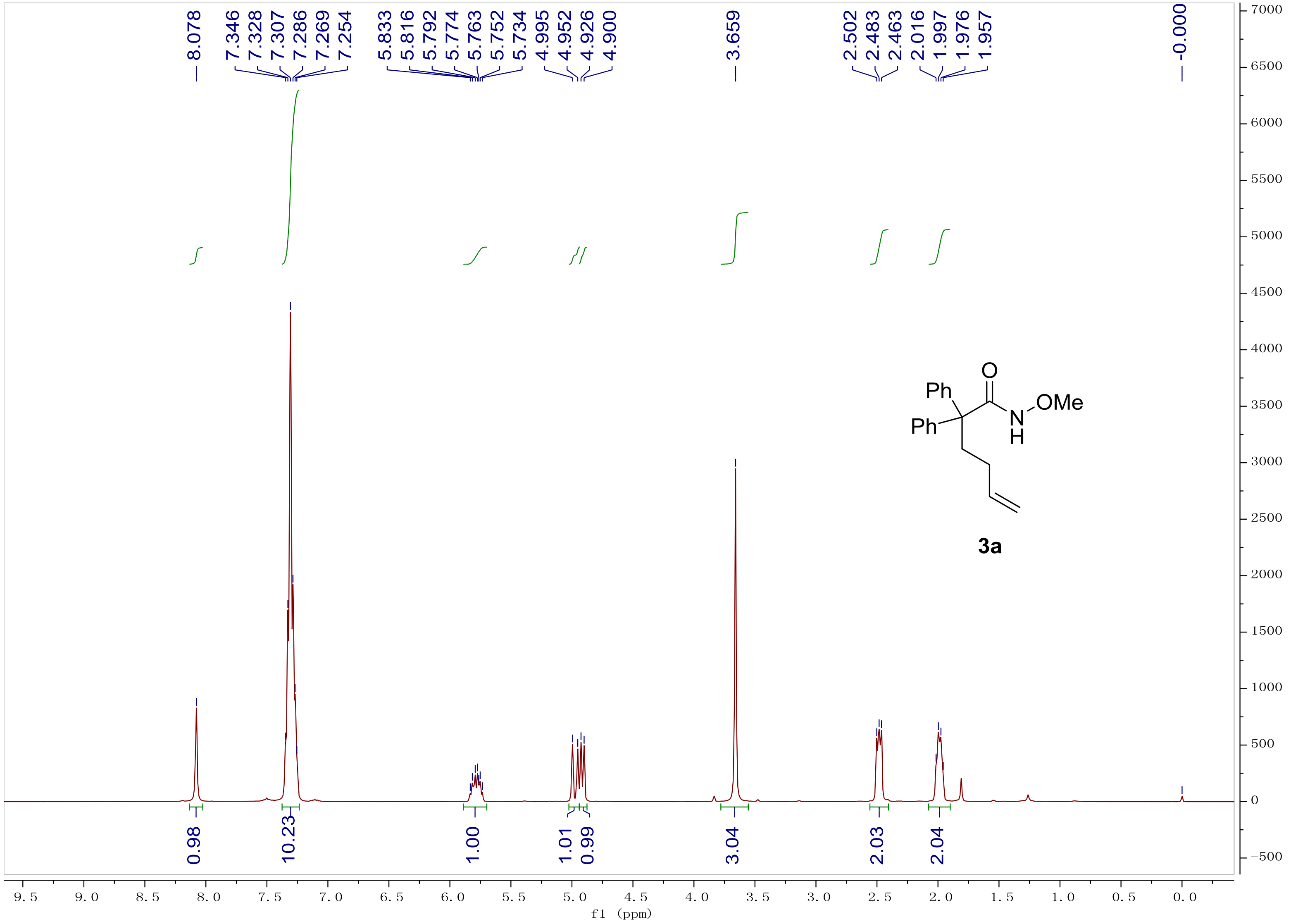
8.078
7.346
7.328
7.307
7.286
7.269
7.254
5.833
5.816
5.792
5.774
5.763
5.752
5.734
4.995
4.952
4.926
4.900
3.659
2.502
2.483
2.463
2.016
1.997
1.976
1.957
-0.000
0.98
10.23
1.00
1.01
0.99
3.04
2.03
2.04
Ph
Ph
O
N
H
OMe
3a
f1 (ppm)

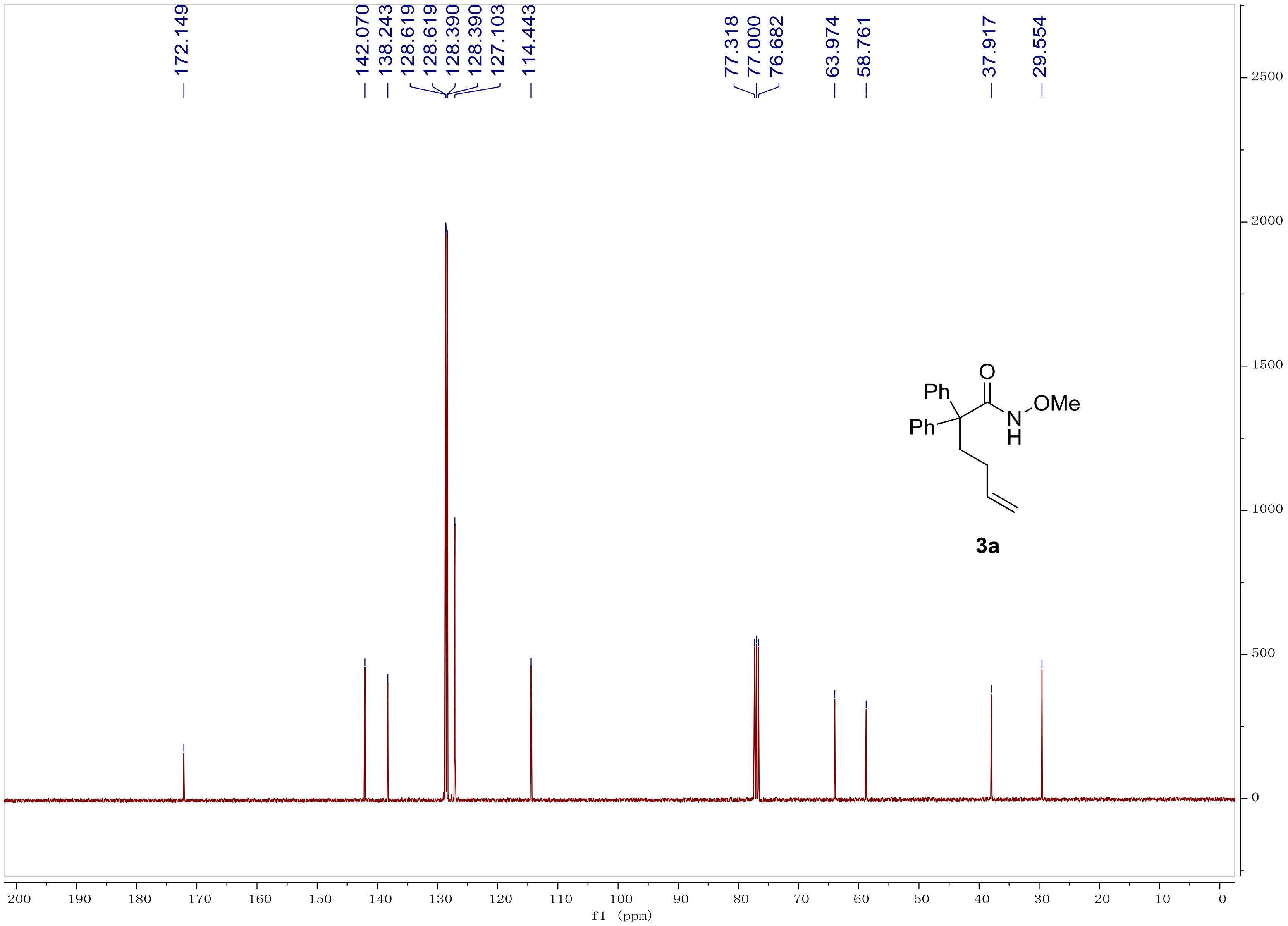

172.149
142.070
138.243
128.619
128.619
128.390
128.390
127.103
114.443
77.318
77.000
76.682
63.974
58.761
37.917
29.554
Ph
Ph
O
N
H
OMe
3a
f1 (ppm)

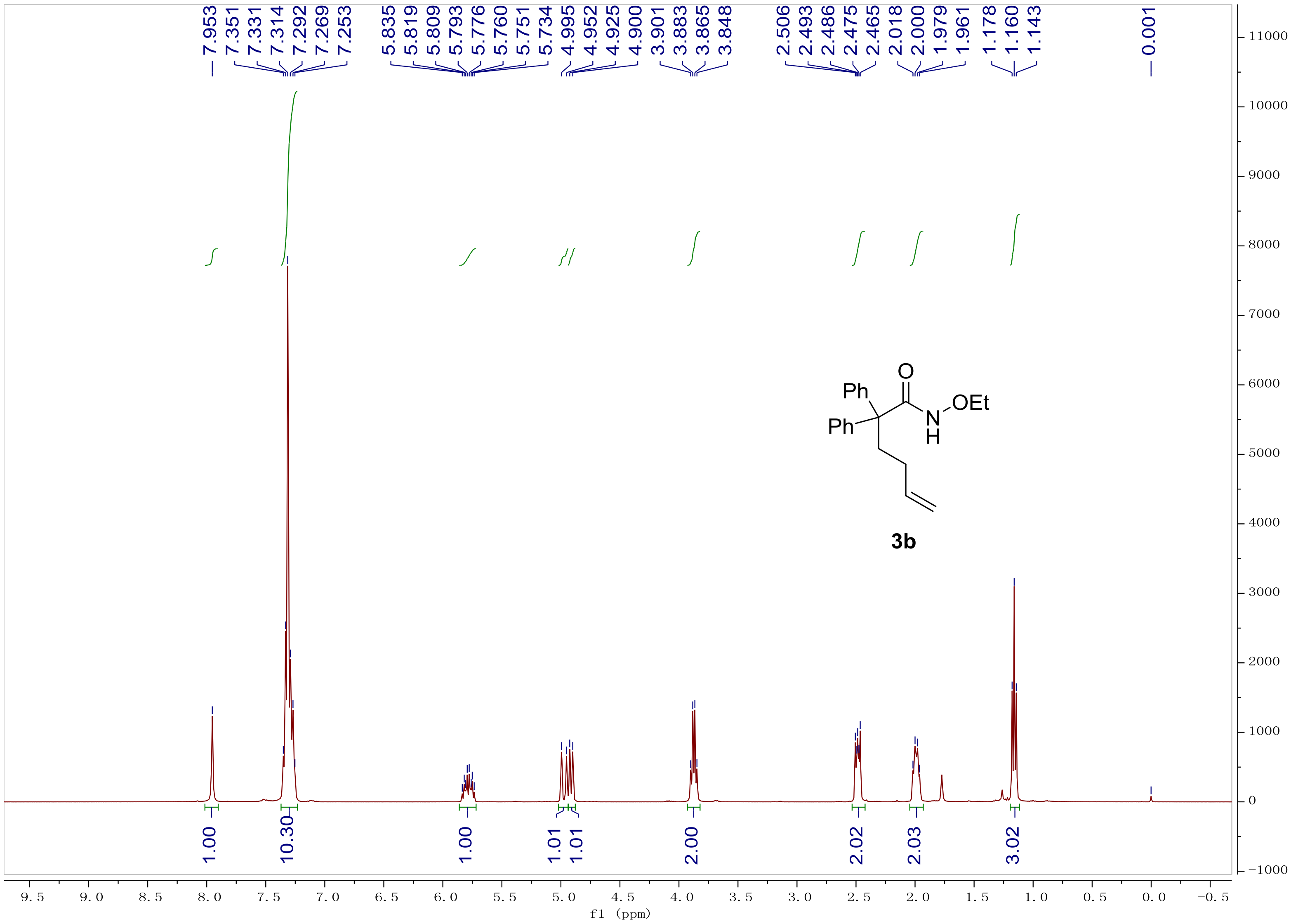
7.953
7.351
7.331
7.314
7.292
7.269
7.253
5.835
5.819
5.809
5.793
5.776
5.760
5.751
5.734
4.995
4.952
4.925
4.900
3.901
3.883
3.865
3.848
2.506
2.493
2.486
2.475
2.465
2.018
2.000
1.979
1.961
1.178
1.160
1.143
0.001
Ph
Ph
O
N
H
OEt
1.00
10.30
1.00
1.01
1.01
2.00
2.02
2.03
3.02
f1 (ppm)

3b

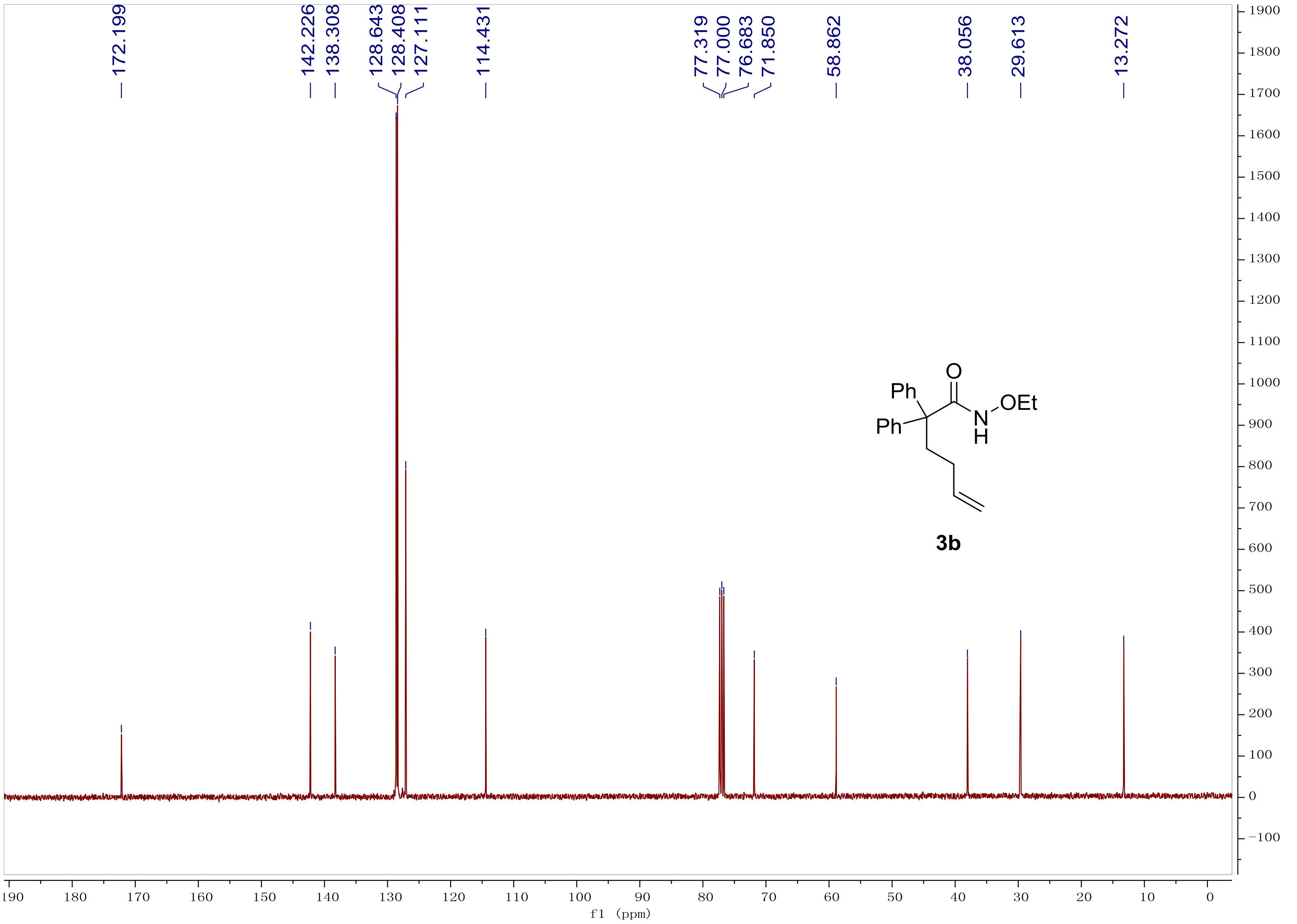
172.199
142.226
138.308
128.643
128.408
127.111
114.431
77.319
77.000
76.683
71.850
58.862
38.056
29.613
13.272
O
Ph
Ph
N
H
OEt
3b
f1 (ppm)

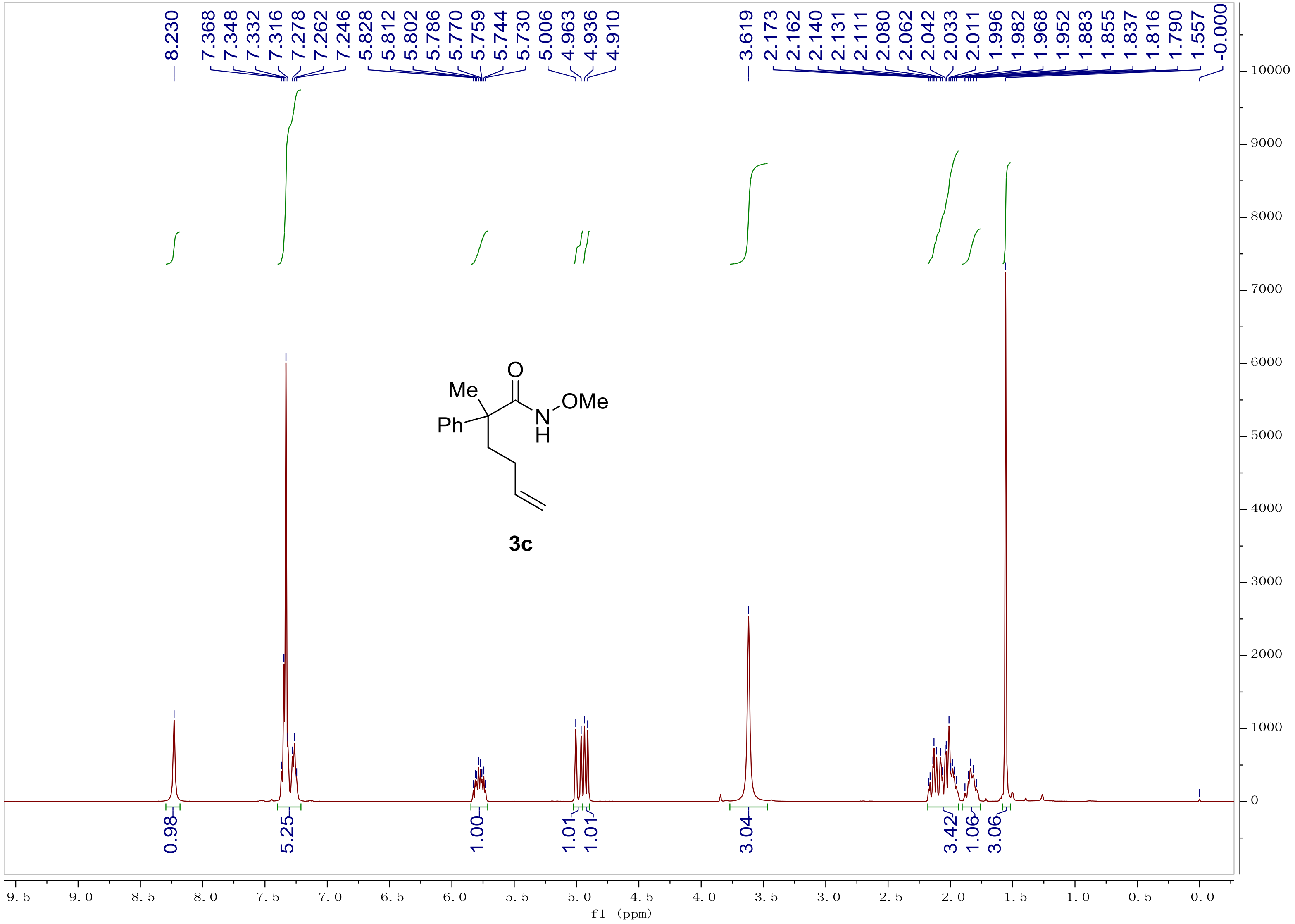
8.230
7.368
7.348
7.332
7.316
7.278
7.262
7.246
5.828
5.812
5.802
5.786
5.770
5.759
5.744
5.730
5.006
4.963
4.936
4.910
3.619
2.173
2.162
2.140
2.131
2.111
2.080
2.062
2.042
2.033
2.011
1.996
1.982
1.968
1.952
1.883
1.855
1.837
1.816
1.790
1.557
-0.000
Me
O
N
OMe
H
Ph
0.98
5.25
1.00
1.01
1.01
3.04
3.42
1.06
3.06
9.5 9.0 8.5 8.0 7.5 7.0 6.5 6.0 5.5 5.0 4.5 4.0 3.5 3.0 2.5 2.0 1.5 1.0 0.5 0.0
f1 (ppm)
10000
9000
8000
7000
6000
5000
4000
3000
2000
1000
0

3c

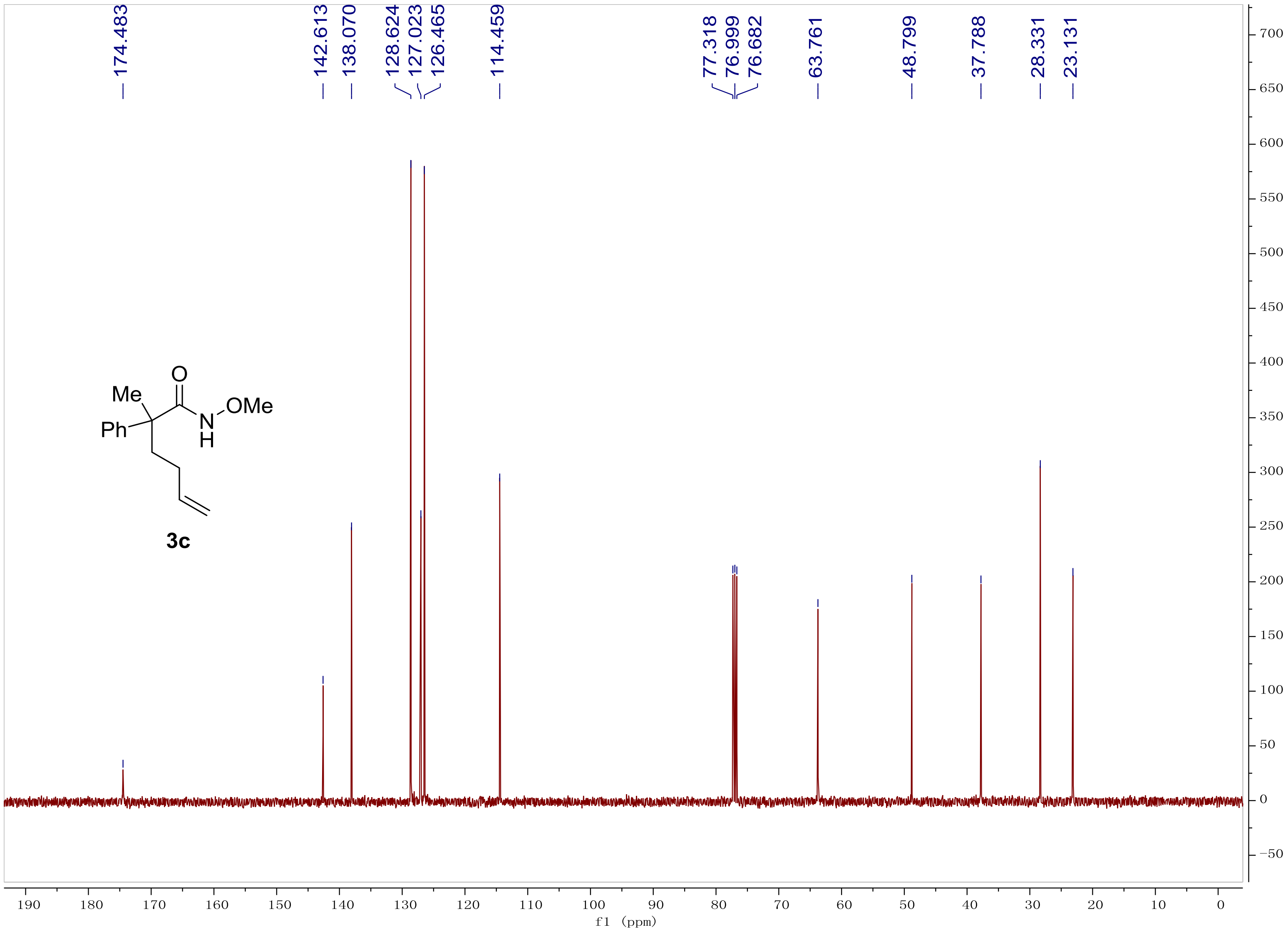

174.483
142.613
138.070
128.624
127.023
126.465
114.459
77.318
76.999
76.682
63.761
48.799
37.788
28.331
23.131
Me
O
Ph
N
H
OMe
3c
f1 (ppm)

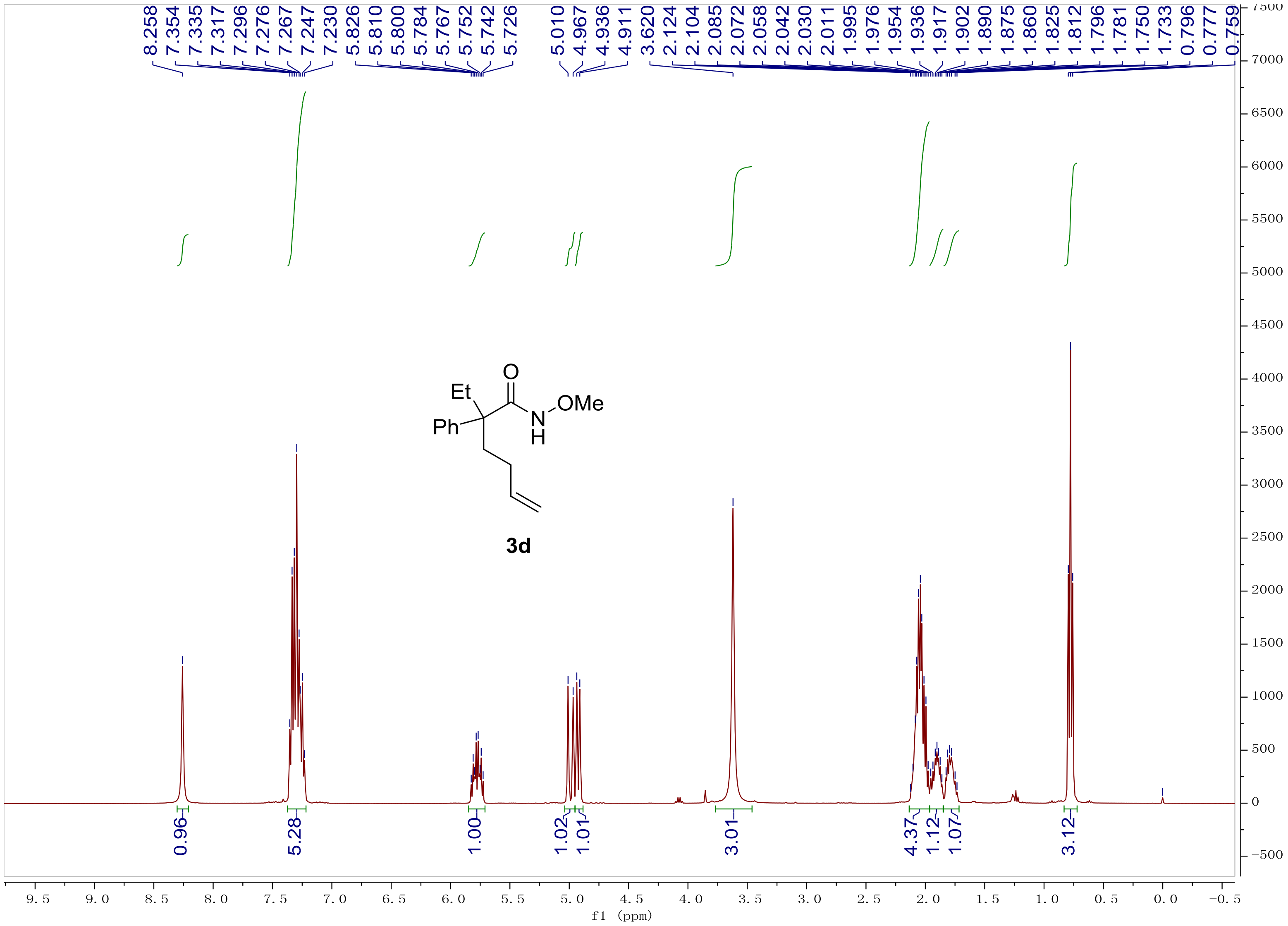
8.258
7.354
7.335
7.317
7.296
7.276
7.267
7.247
7.230
5.826
5.810
5.800
5.784
5.767
5.752
5.742
5.726
5.010
4.967
4.936
4.911
3.620
2.124
2.104
2.085
2.072
2.058
2.042
2.030
2.011
1.995
1.976
1.954
1.936
1.917
1.902
1.890
1.875
1.860
1.825
1.812
1.796
1.781
1.750
1.733
0.796
0.777
0.759
0.96
5.28
1.00
1.02
1.01
3.01
4.37
1.12
1.07
3.12
O
Et
OMe
N
H
Ph
3d
f1 (ppm)

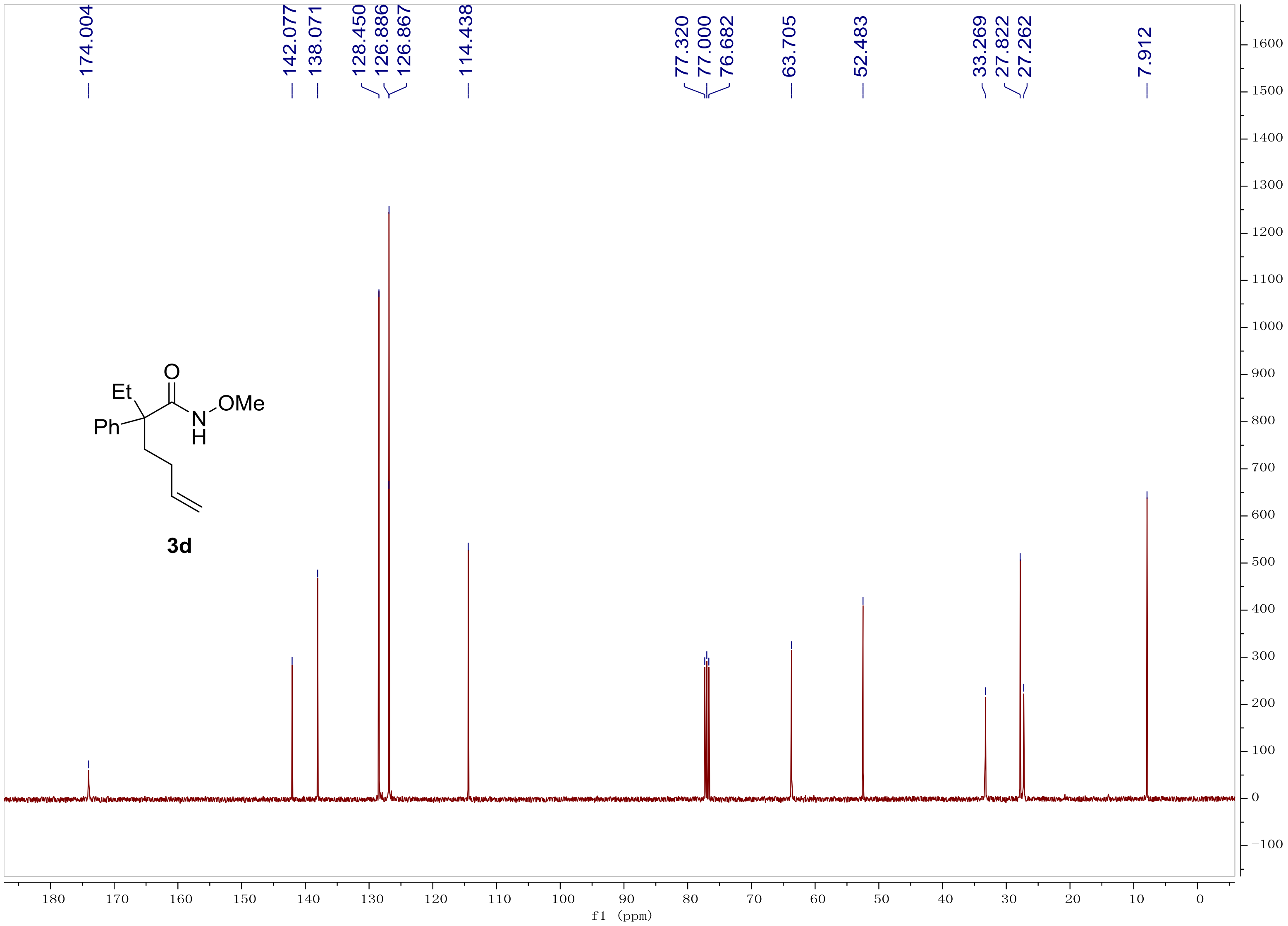
174.004
142.077
138.071
128.450
126.886
126.867
114.438
77.320
77.000
76.682
63.705
52.483
33.269
27.822
27.262
7.912
Et
O
OMe
Ph
N
H
3d
f1 (ppm)

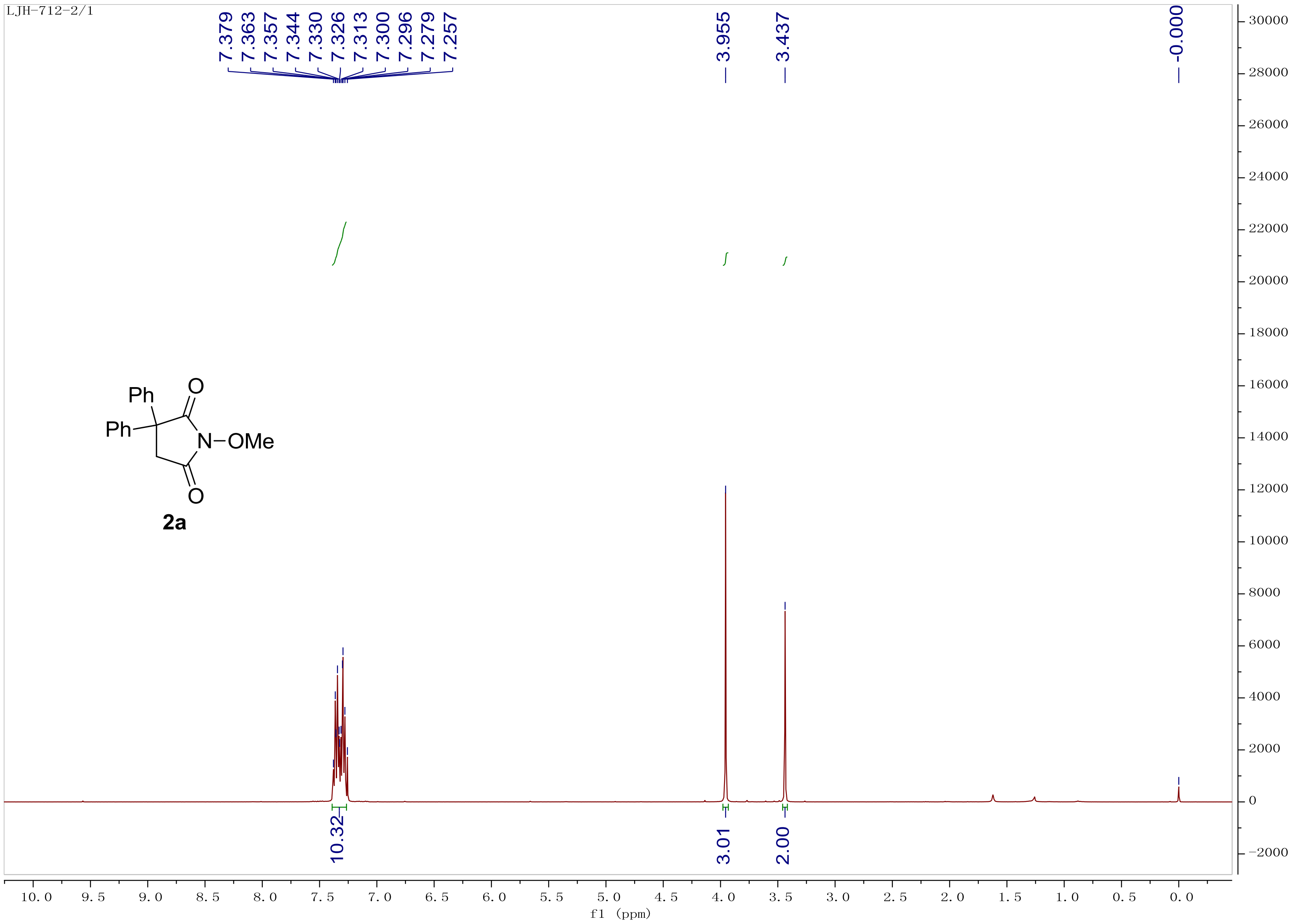
LJH-712-2/1
7.379
7.363
7.357
7.344
7.330
7.326
7.313
7.300
7.296
7.279
7.257
3.955
3.437
-0.000
10.32
3.01
2.00
Ph
Ph
O
N-OMe
O
f1 (ppm)

2a

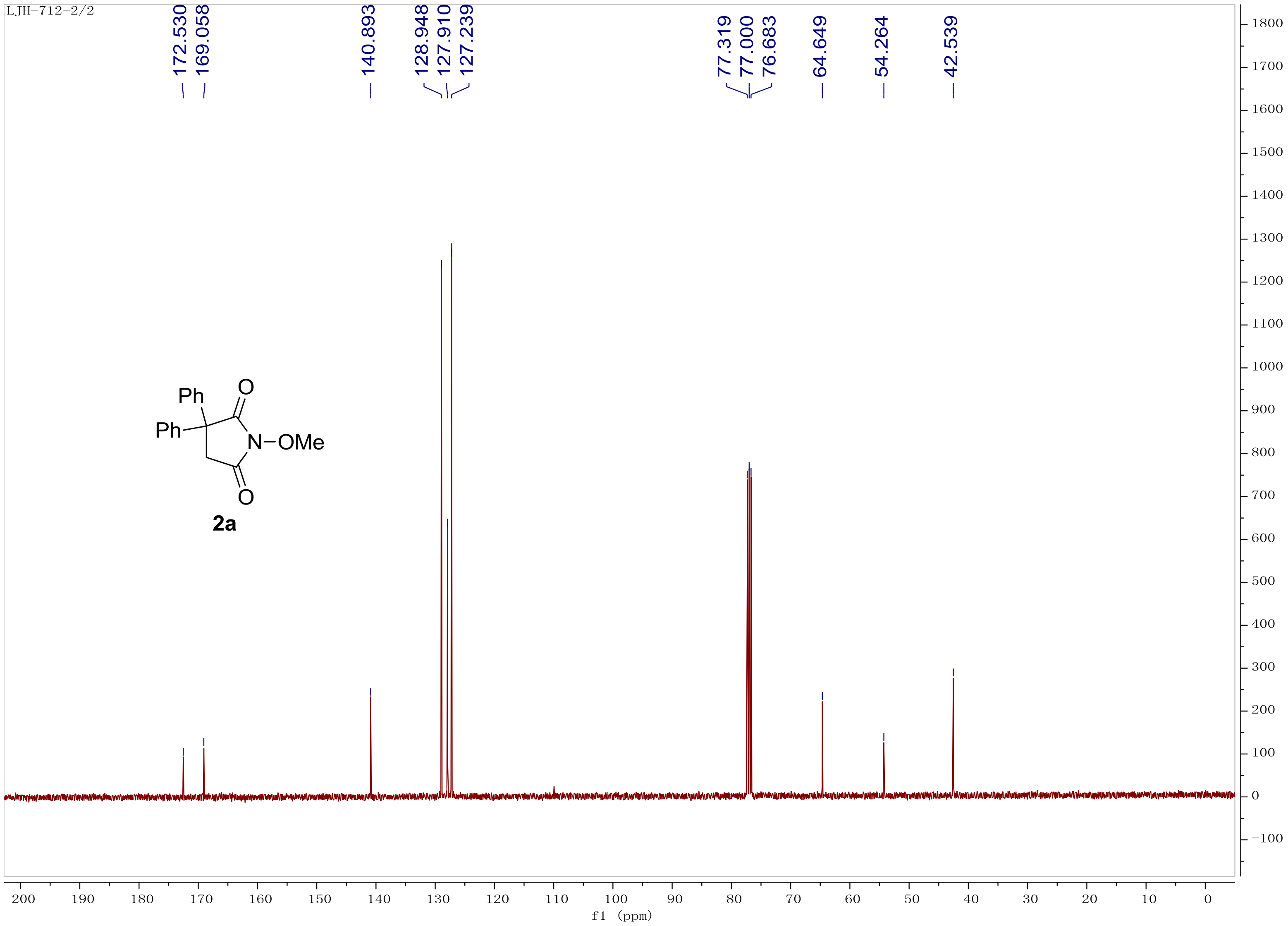

LJH-712-2/2
172.530
169.058
140.893
128.948
127.910
127.239
77.319
77.000
76.683
64.649
54.264
42.539
Ph
Ph
O
O
N–OMe
2a
f1 (ppm)

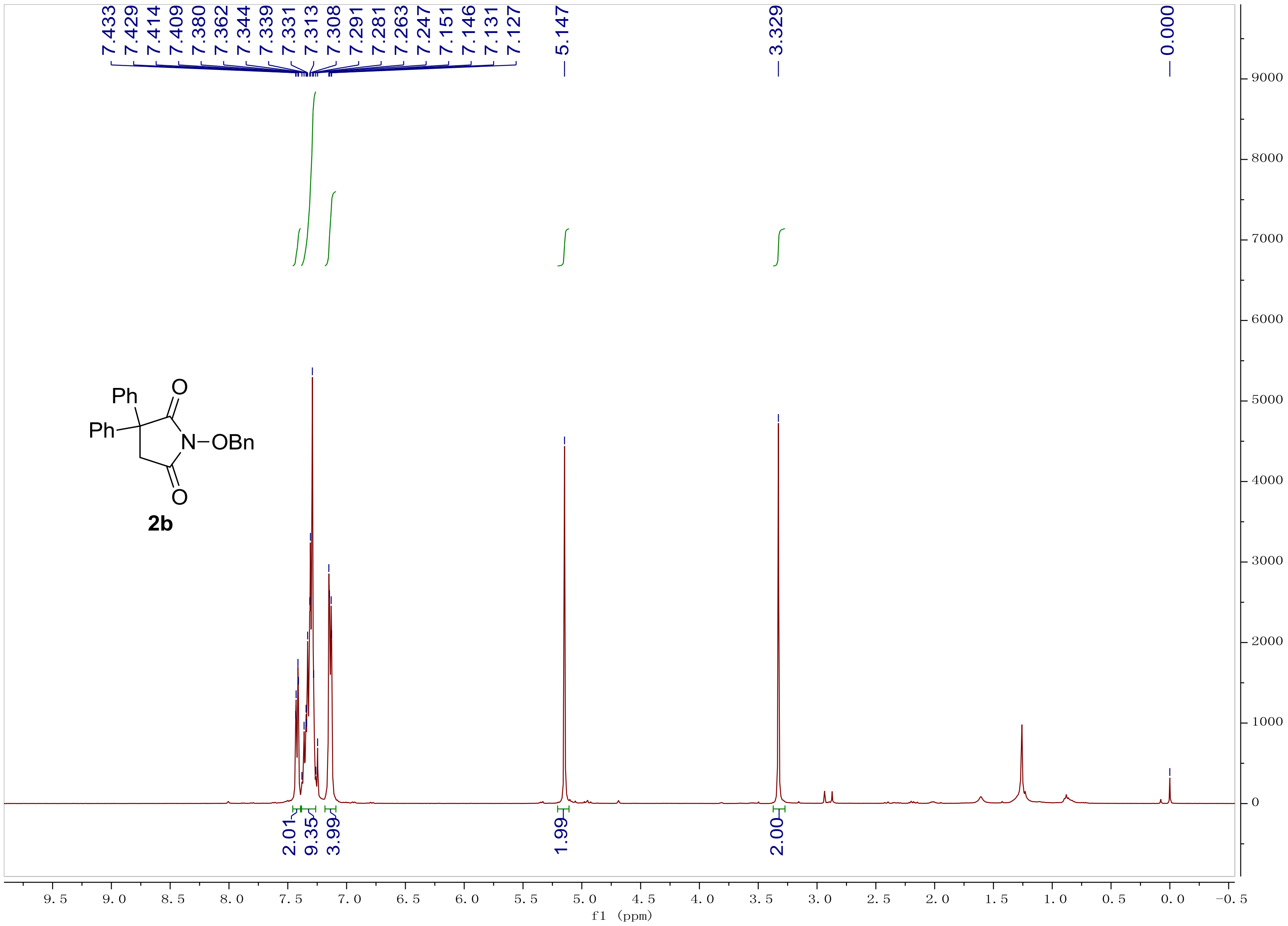
7.433
7.429
7.414
7.409
7.380
7.362
7.344
7.339
7.331
7.313
7.308
7.291
7.281
7.263
7.247
7.151
7.146
7.131
7.127
5.147
3.329
0.000
Ph
Ph
O
N−OBn
O
2b
2.01
9.35
3.99
1.99
2.00
f1 (ppm)

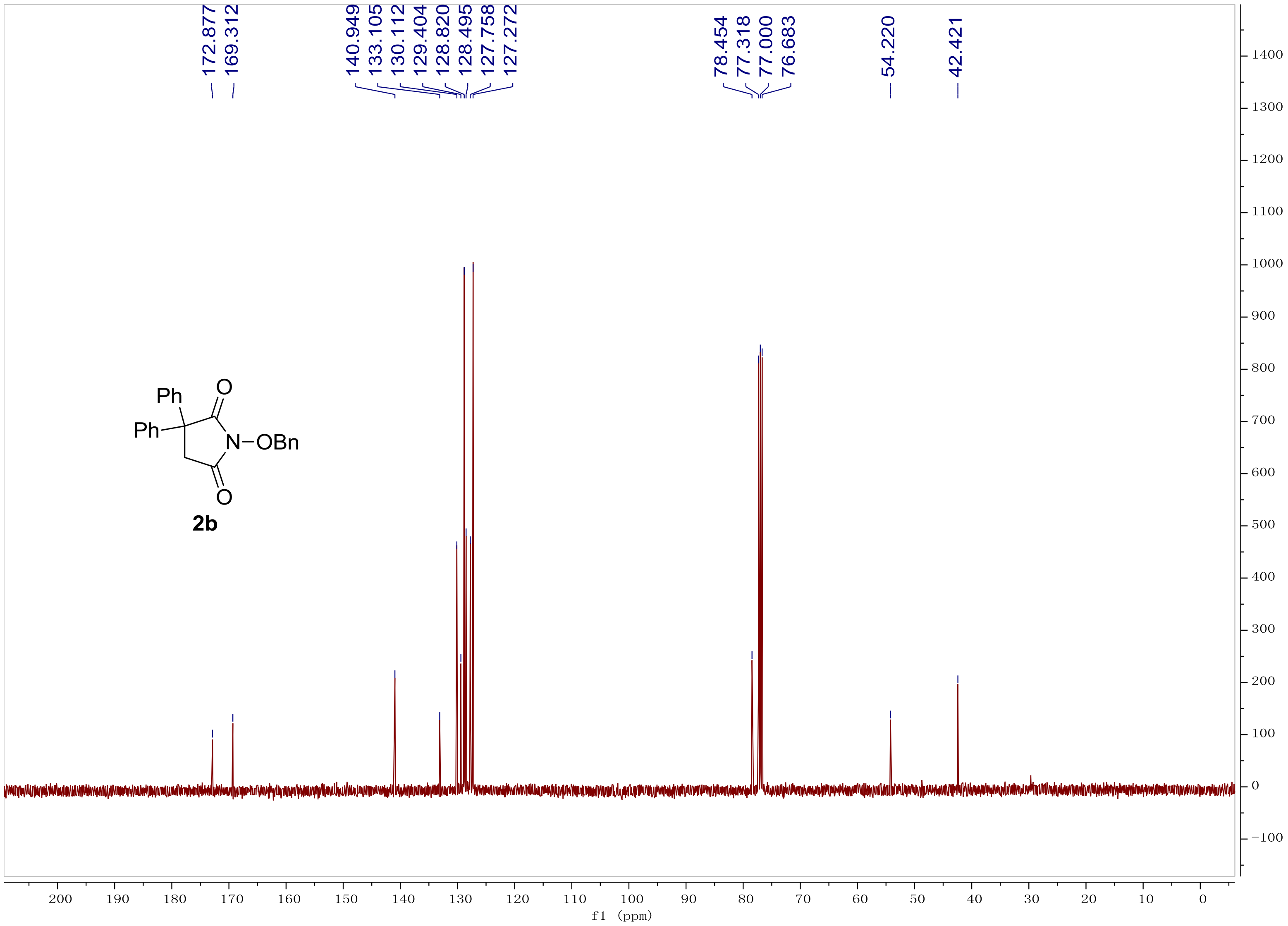

172.877
169.312
140.949
133.105
130.112
129.404
128.820
128.495
127.758
127.272
78.454
77.318
77.000
76.683
54.220
42.421
Ph
Ph
O
N−OBn
O
2b
1400
1300
1200
1100
1000
900
800
700
600
500
400
300
200
100
0
−100
200 190 180 170 160 150 140 130 120 110 100 90 80 70 60 50 40 30 20 10 0
f1 (ppm)

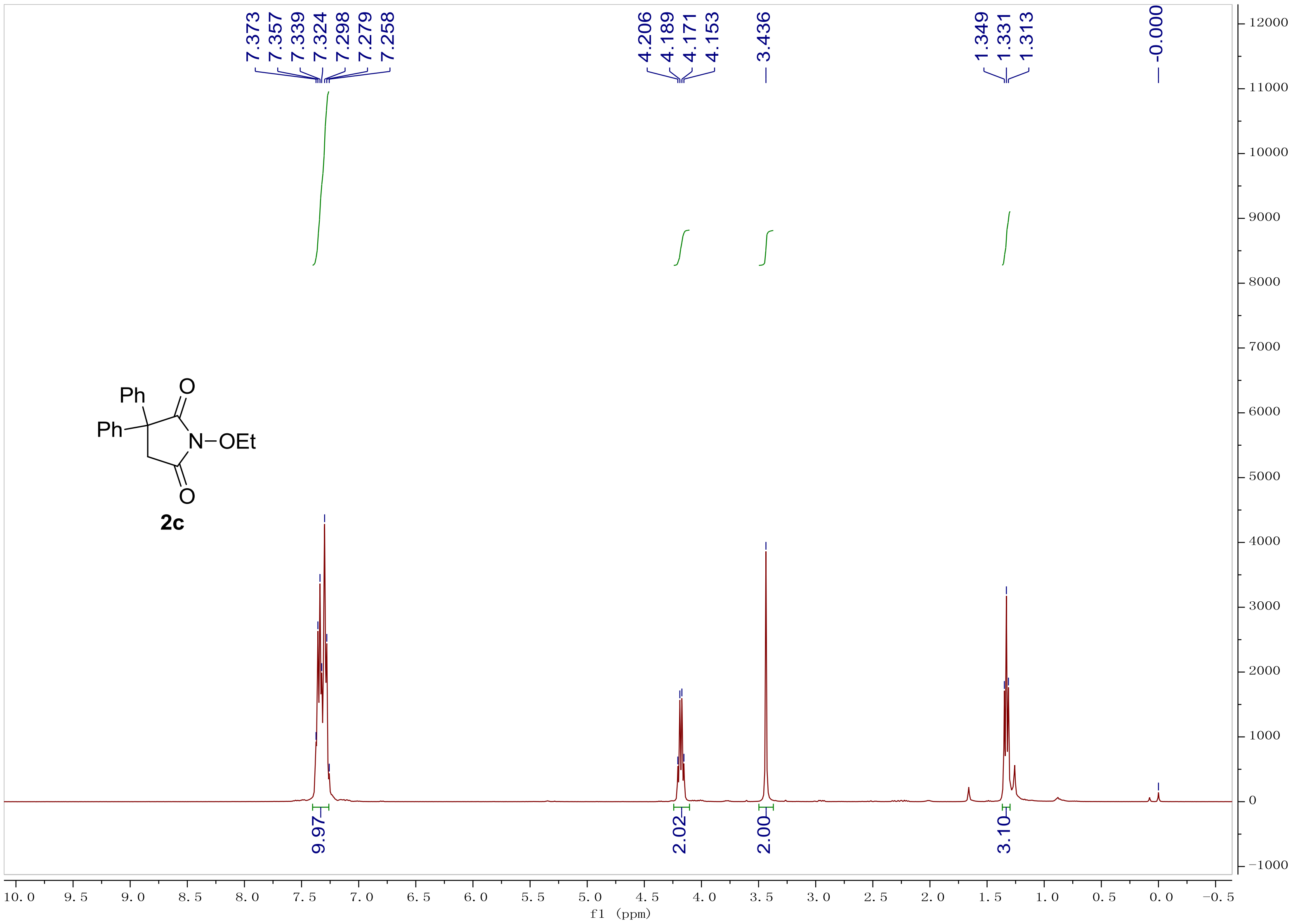
7.373
7.357
7.339
7.324
7.298
7.279
7.258
4.206
4.189
4.171
4.153
3.436
1.349
1.331
1.313
-0.000
Ph
Ph
O
O
N−OEt
2c
9.97
2.02
2.00
3.10
f1 (ppm)

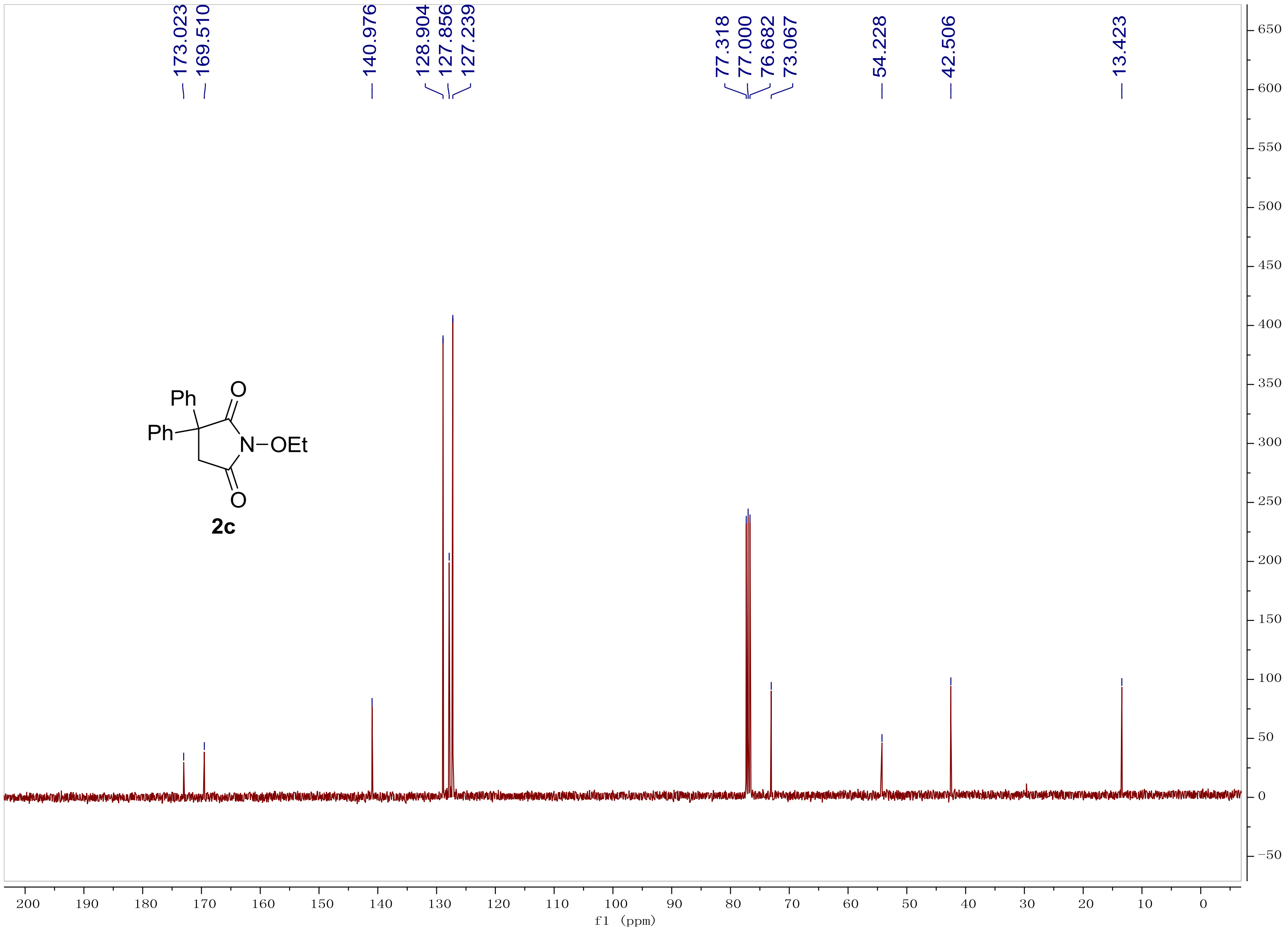
173.023
169.510
140.976
128.904
127.856
127.239
77.318
77.000
76.682
73.067
54.228
42.506
13.423
Ph
Ph
O
N–OEt
O
2c
f1 (ppm)

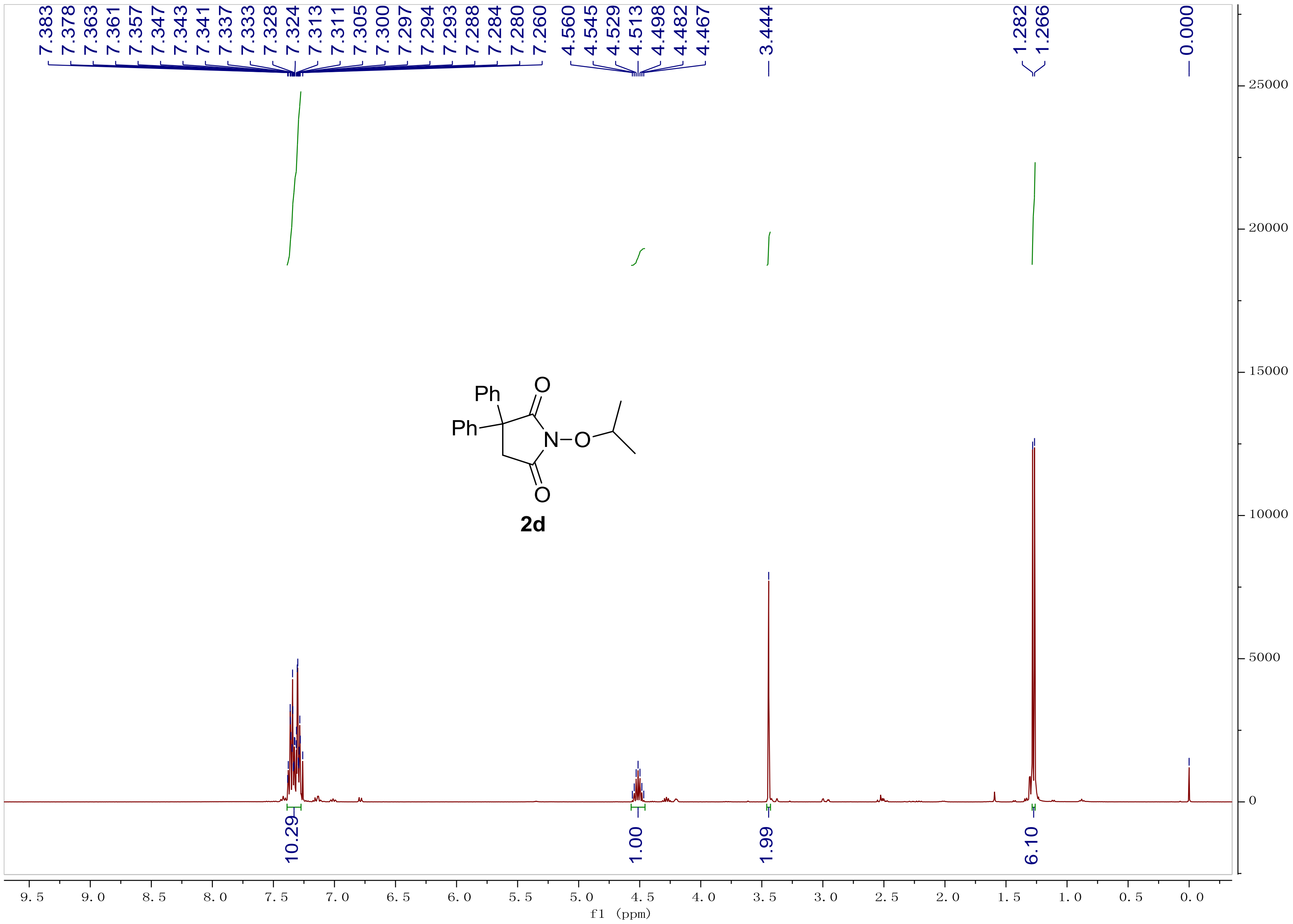
7.383
7.378
7.363
7.361
7.357
7.347
7.343
7.341
7.337
7.333
7.328
7.324
7.313
7.311
7.305
7.300
7.297
7.294
7.293
7.288
7.284
7.280
7.260
4.560
4.545
4.529
4.513
4.498
4.482
4.467
3.444
1.282
1.266
0.000
Ph
Ph
O
O
N
O
2d
10.29
1.00
1.99
6.10
25000
20000
15000
10000
5000
0
9.5
9.0
8.5
8.0
7.5
7.0
6.5
6.0
5.5
5.0
4.5
4.0
3.5
3.0
2.5
2.0
1.5
1.0
0.5
0.0
f1 (ppm)

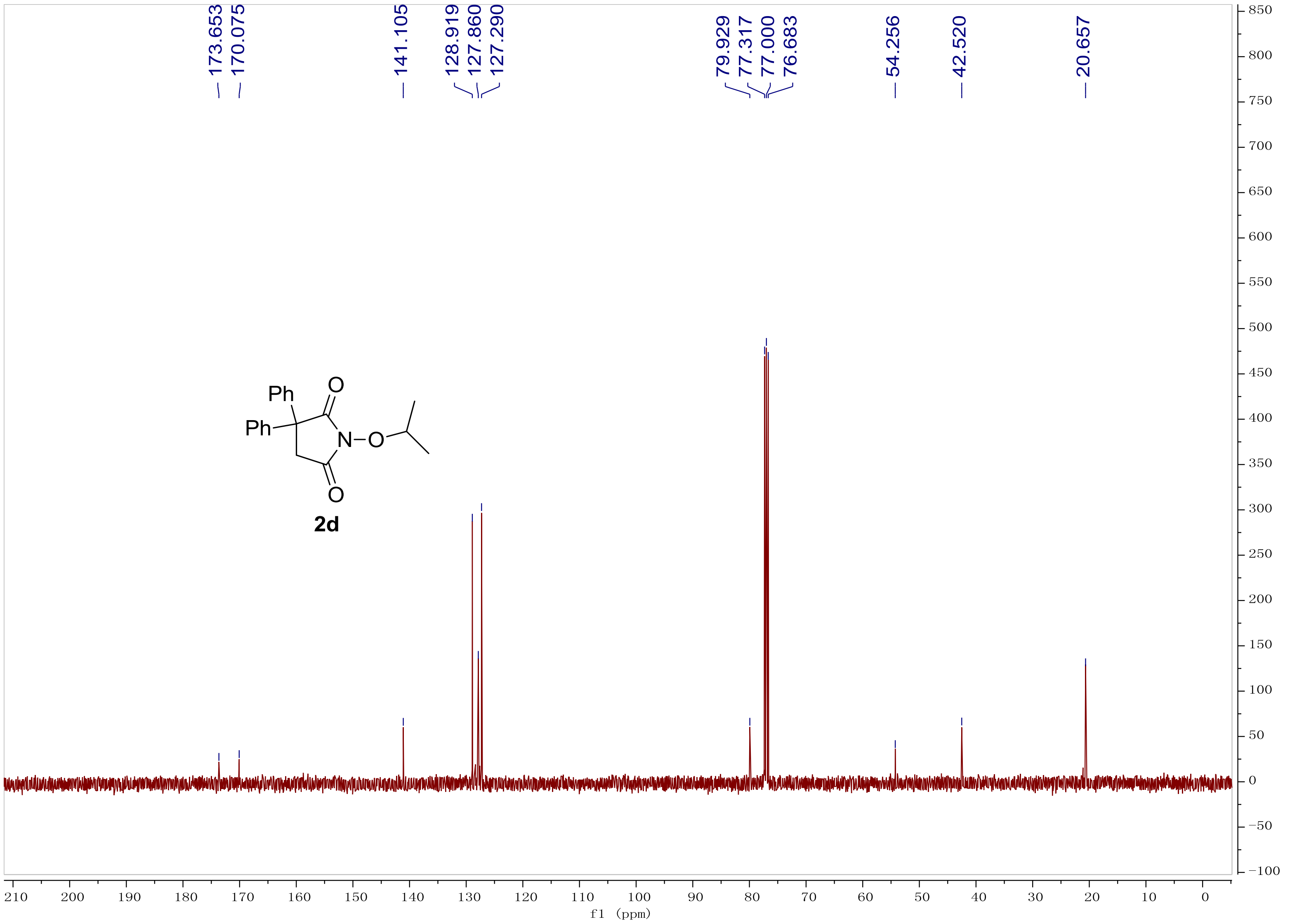
173.653
170.075
141.105
128.919
127.860
127.290
79.929
77.317
77.000
76.683
54.256
42.520
20.657
Ph
Ph
O
N
O
O
2d
f1 (ppm)

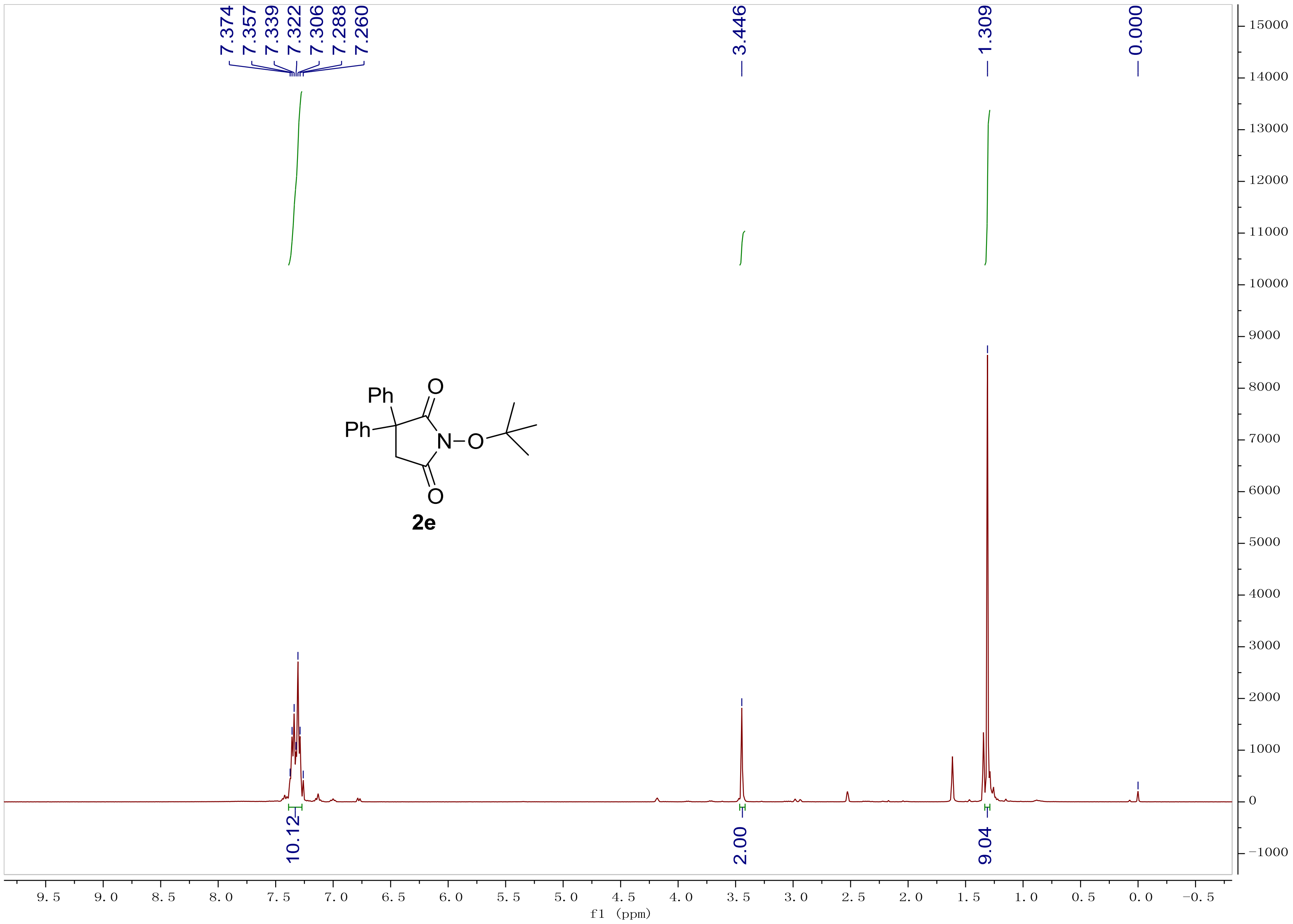

7.374
7.357
7.339
7.322
7.306
7.288
7.260
3.446
1.309
0.000
Ph
Ph
O
N
O
O
2e
10.12
2.00
9.04
f1 (ppm)

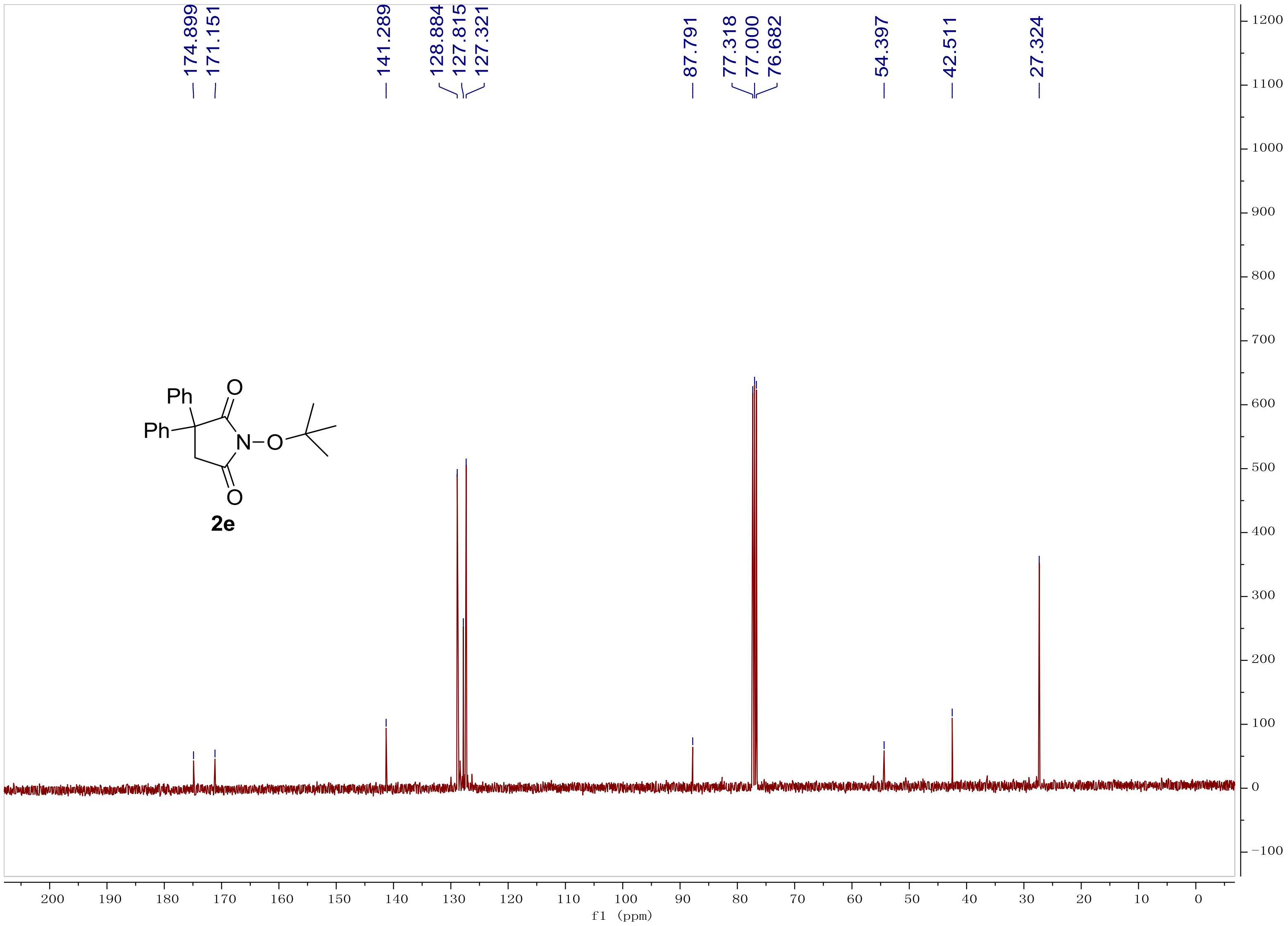

174.899
171.151
141.289
128.884
127.815
127.321
87.791
77.318
77.000
76.682
54.397
42.511
27.324
Ph
Ph
O
N
O
O
2e
1200
1100
1000
900
800
700
600
500
400
300
200
100
0
−100
200 190 180 170 160 150 140 130 120 110 100 90 80 70 60 50 40 30 20 10 0
f1 (ppm)

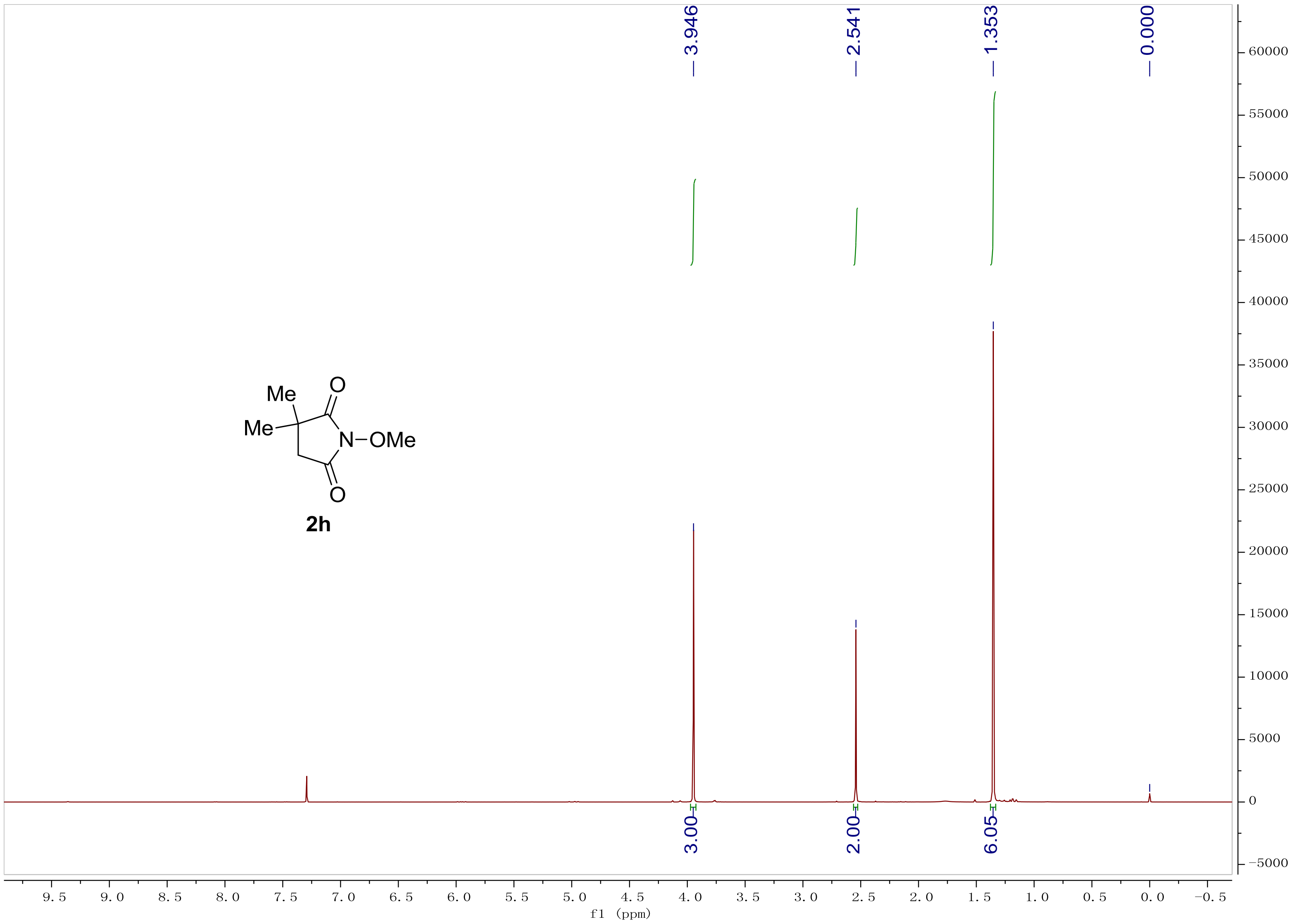

3.946
2.541
1.353
0.000
Me
Me
O
N–OMe
O
2h
3.00
2.00
6.05
f1 (ppm)

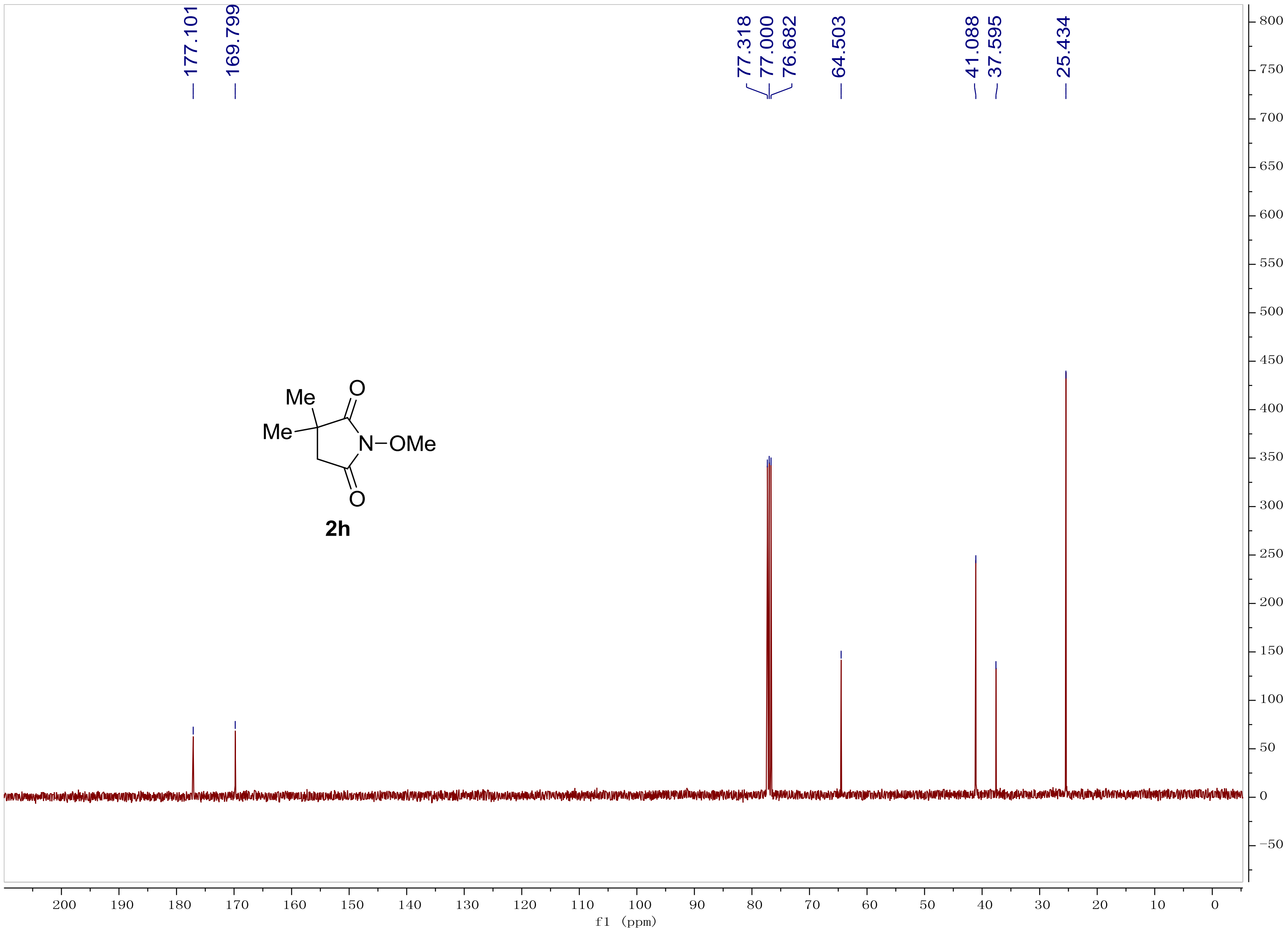

177.101
169.799
77.318
77.000
76.682
64.503
41.088
37.595
25.434
Me
Me
O
N−OMe
O
800
750
700
650
600
550
500
450
400
350
300
250
200
150
100
50
0
−50
200
190
180
170
160
150
140
130
120
110
100
90
80
70
60
50
40
30
20
10
0
f1 (ppm)

2h

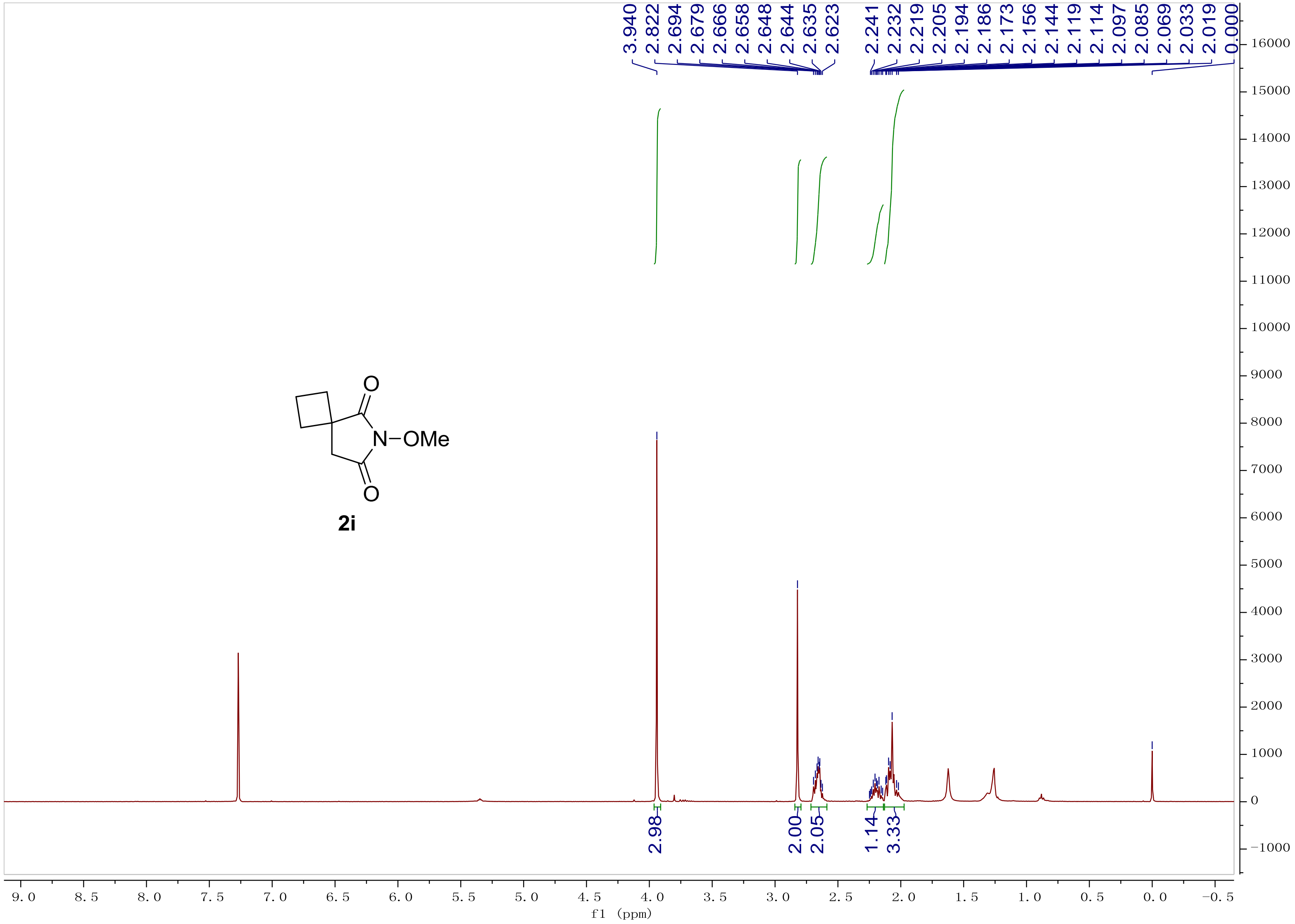
3.940
2.822
2.694
2.679
2.666
2.658
2.648
2.644
2.635
2.623
2.241
2.232
2.219
2.205
2.194
2.186
2.173
2.156
2.144
2.119
2.114
2.097
2.085
2.069
2.033
2.019
0.000
N–OMe
O
O
2i
2.98
2.00
2.05
1.14
3.33
f1 (ppm)

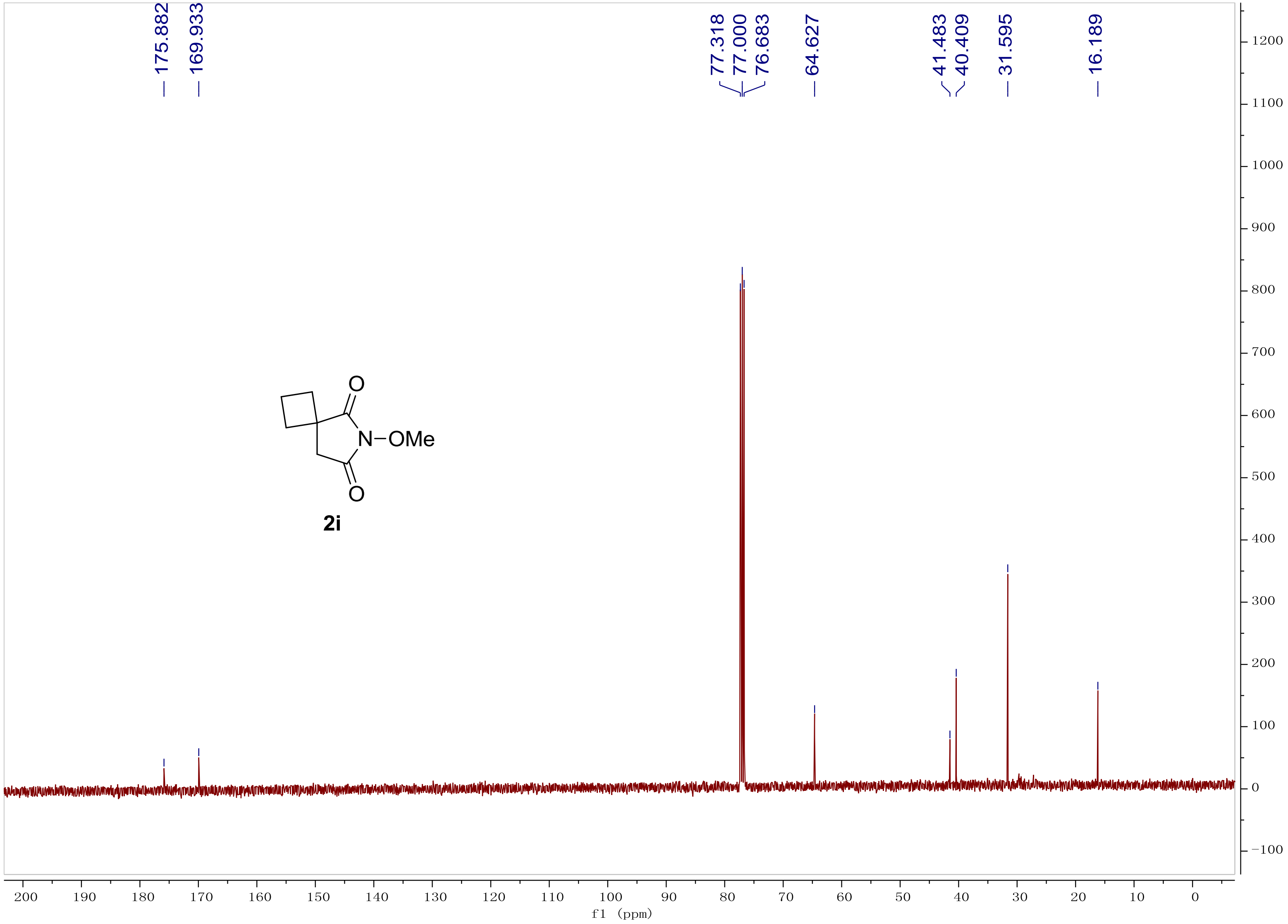
175.882
169.933
77.318
77.000
76.683
64.627
41.483
40.409
31.595
16.189
O
N–OMe
O
2i
1200
1100
1000
900
800
700
600
500
400
300
200
100
0
−100
200 190 180 170 160 150 140 130 120 110 100 90 80 70 60 50 40 30 20 10 0
f1 (ppm)

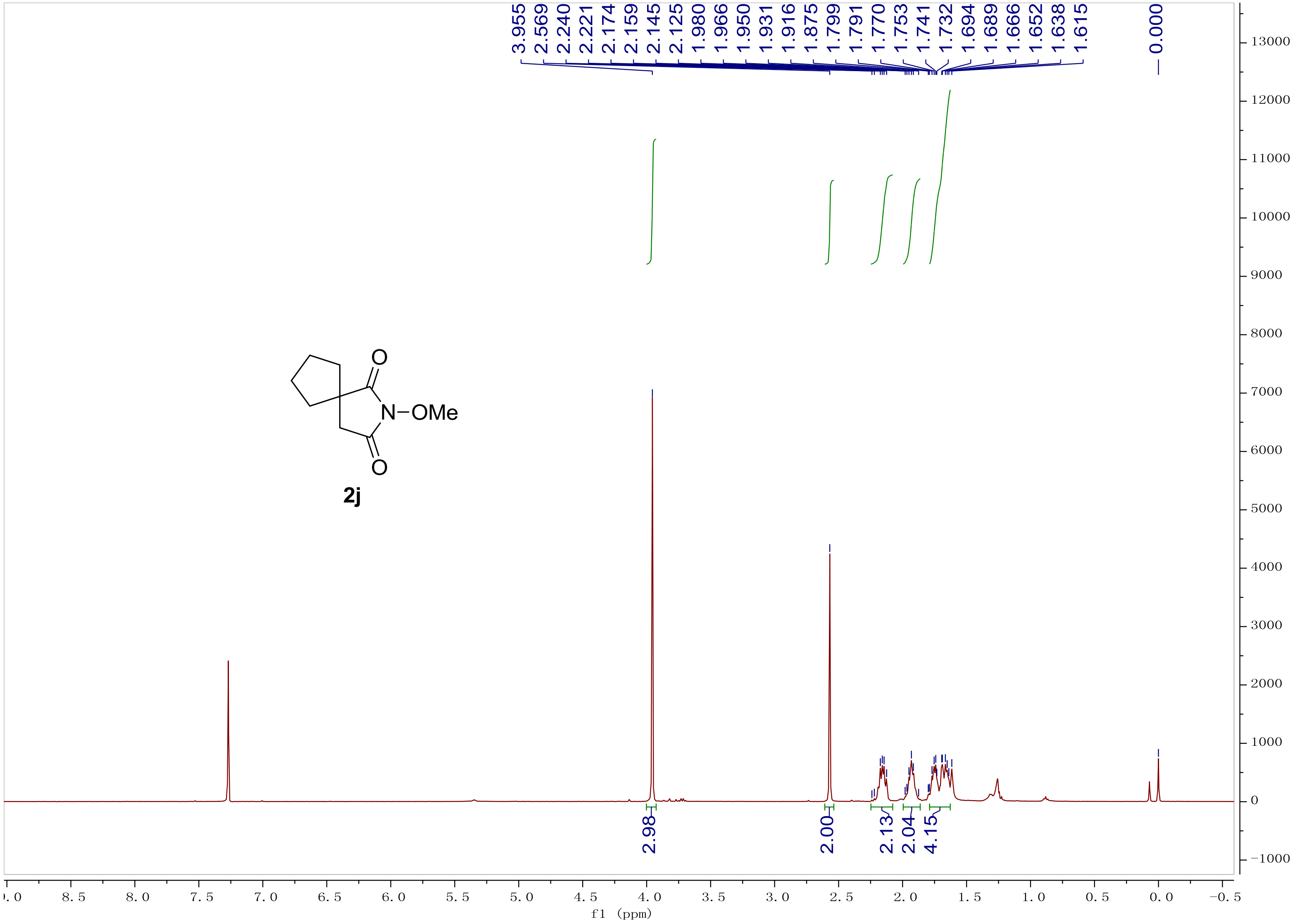
3.955
2.569
2.240
2.221
2.174
2.159
2.145
2.125
1.980
1.966
1.950
1.931
1.916
1.875
1.799
1.791
1.770
1.753
1.741
1.732
1.694
1.689
1.666
1.652
1.638
1.615
0.000
N−OMe
O
O
2j
2.98
2.00
2.13
2.04
4.15
f1 (ppm)

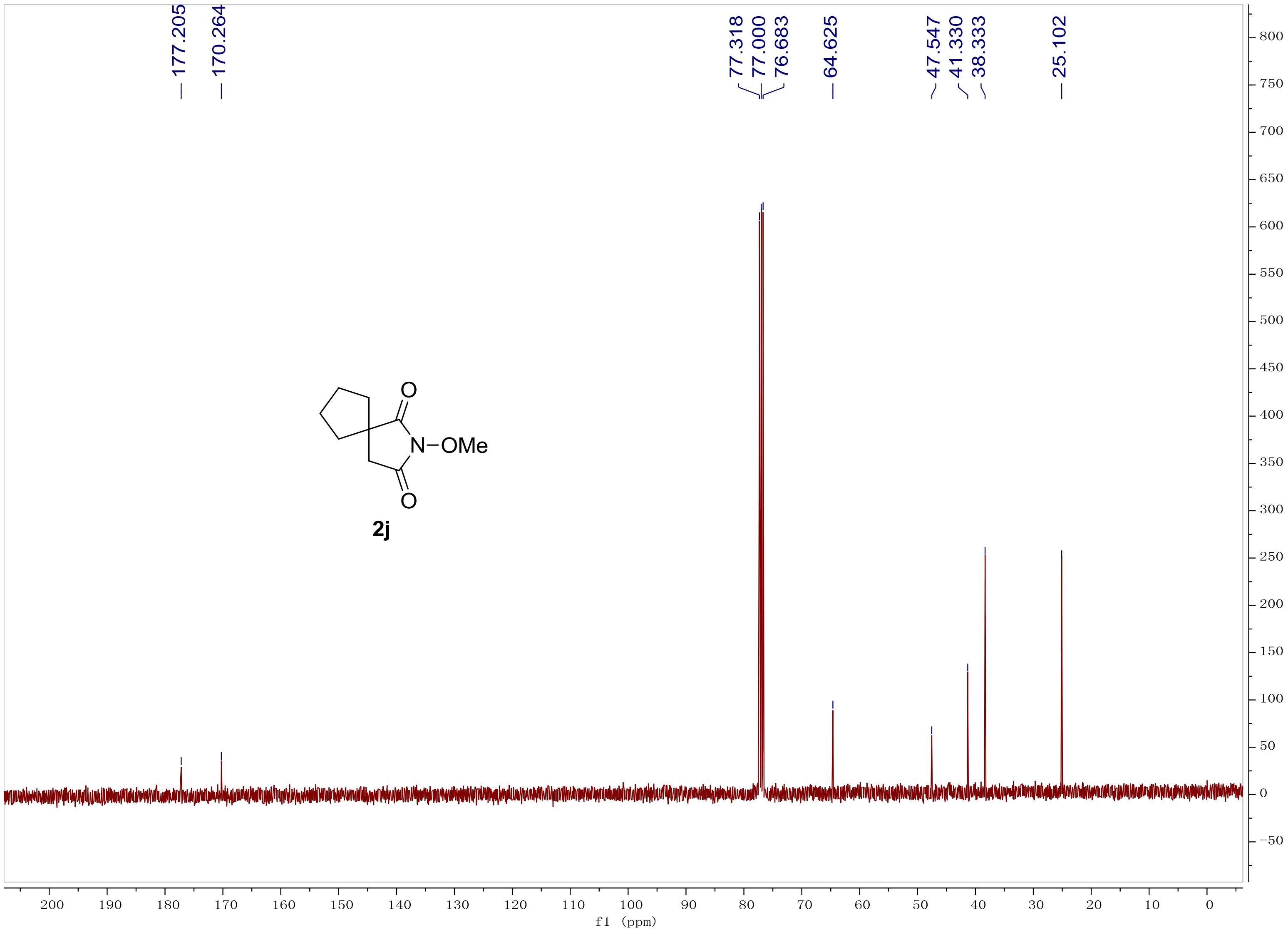
177.205
170.264
77.318
77.000
76.683
64.625
47.547
41.330
38.333
25.102
O
N−OMe
O
2j
f1 (ppm)

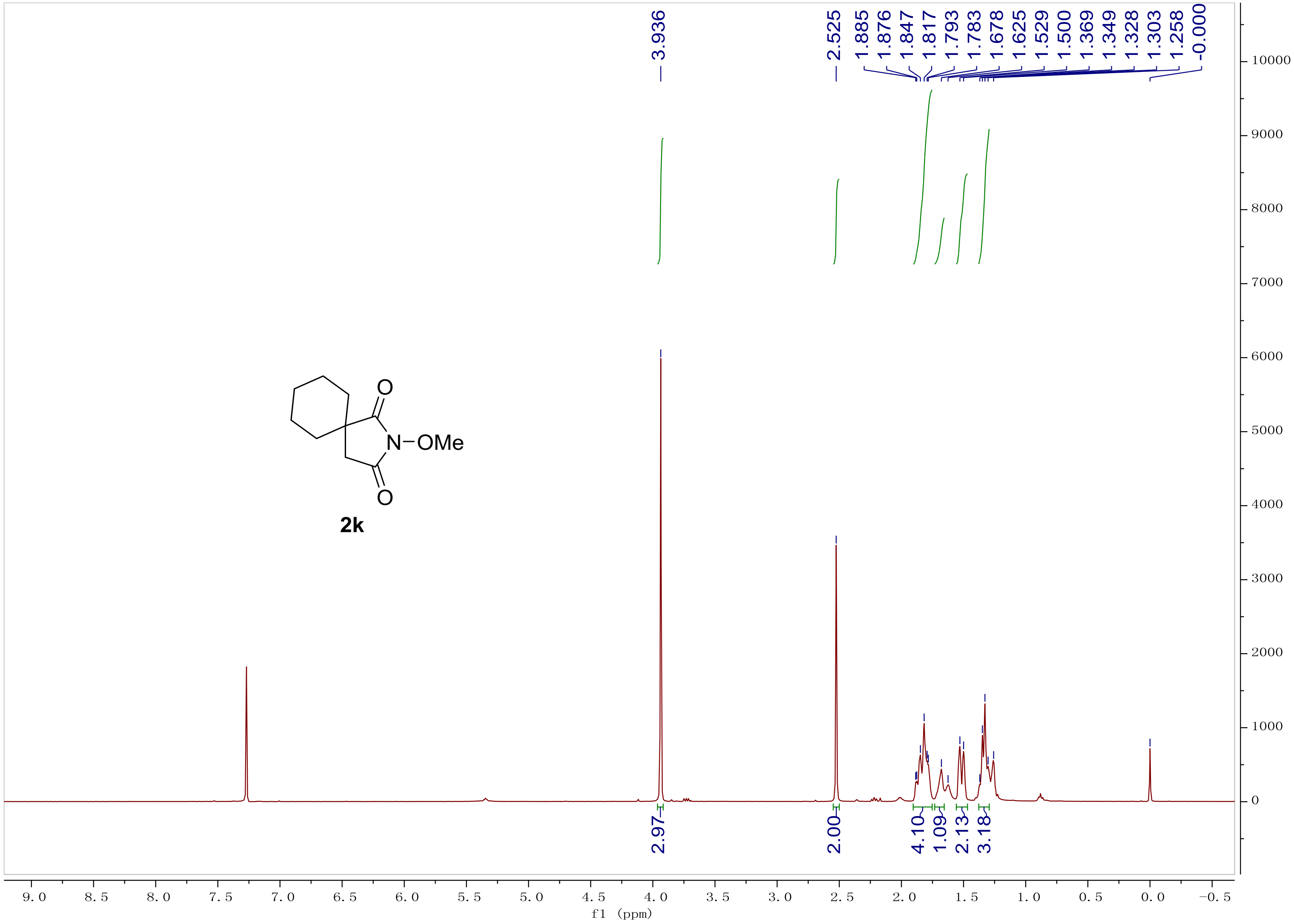

3.936
2.525
1.885
1.876
1.847
1.817
1.793
1.783
1.678
1.625
1.529
1.500
1.369
1.349
1.328
1.303
1.258
-0.000
O
N–OMe
O
2k
2.97
2.00
4.10
1.09
2.13
3.18
10000
9000
8000
7000
6000
5000
4000
3000
2000
1000
0
9.0
8.5
8.0
7.5
7.0
6.5
6.0
5.5
5.0
4.5
4.0
3.5
3.0
2.5
2.0
1.5
1.0
0.5
0.0
-0.5
f1 (ppm)

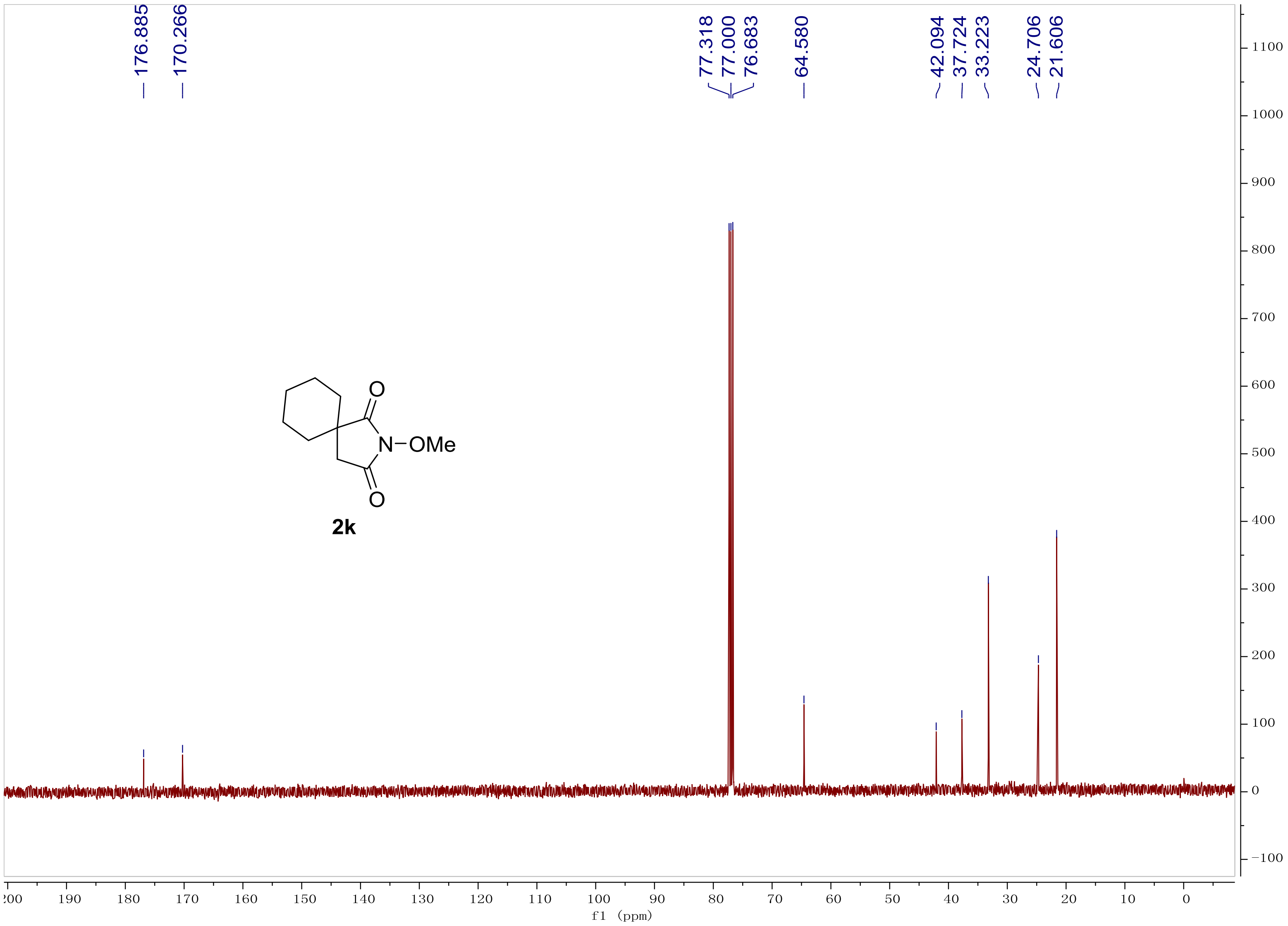
176.885
170.266
77.318
77.000
76.683
64.580
42.094
37.724
33.223
24.706
21.606
O
N−OMe
O
2k
1100
1000
900
800
700
600
500
400
300
200
100
0
−100
200 190 180 170 160 150 140 130 120 110 100 90 80 70 60 50 40 30 20 10 0
f1 (ppm)

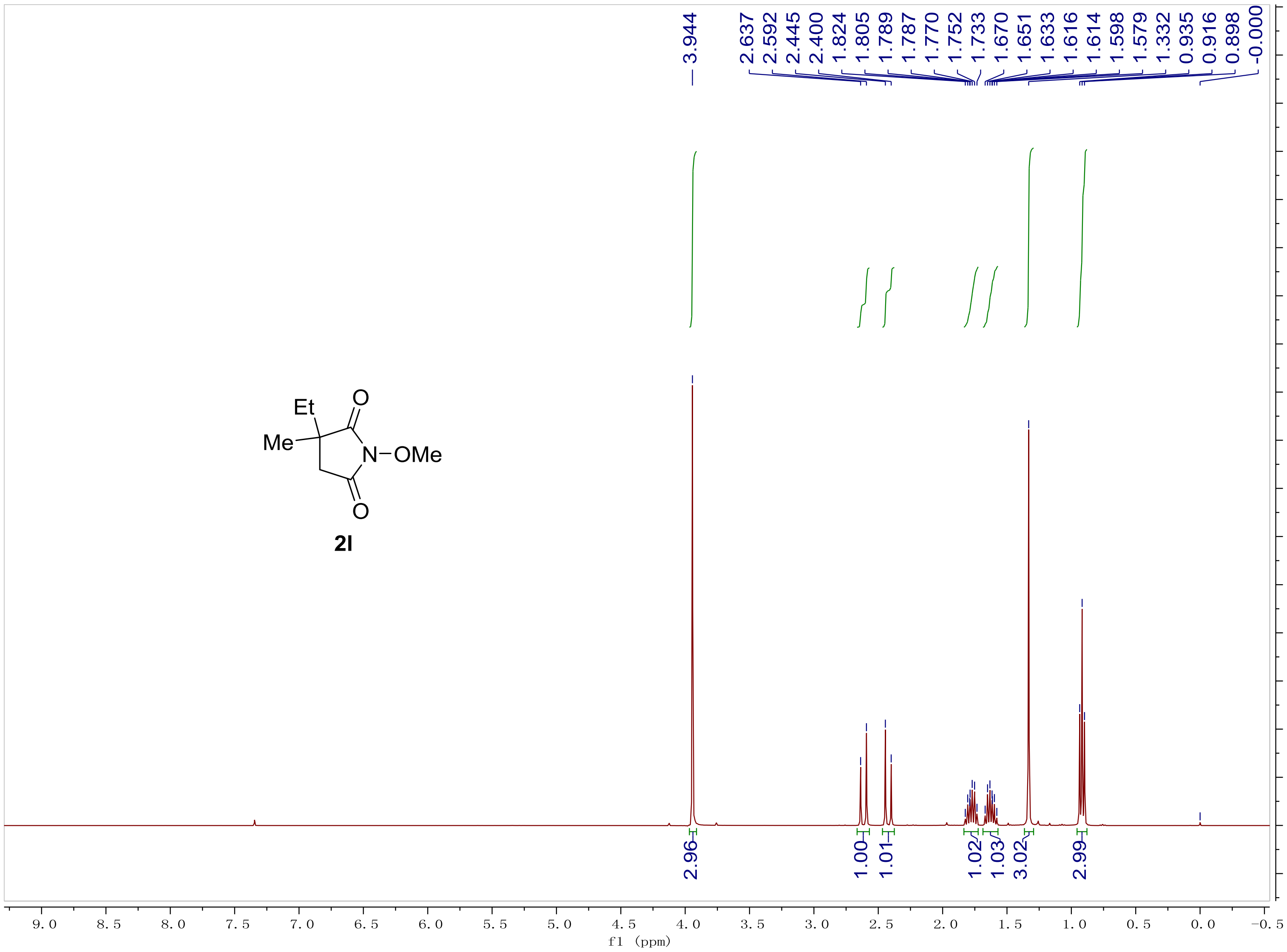
Et
Me
O
N-OMe
O
2l
3.944
2.637
2.592
2.445
2.400
1.824
1.805
1.789
1.787
1.770
1.752
1.733
1.670
1.651
1.633
1.616
1.614
1.598
1.579
1.332
0.935
0.916
0.898
-0.000
2.96
1.00
1.01
1.02
1.03
3.02
2.99
f1 (ppm)

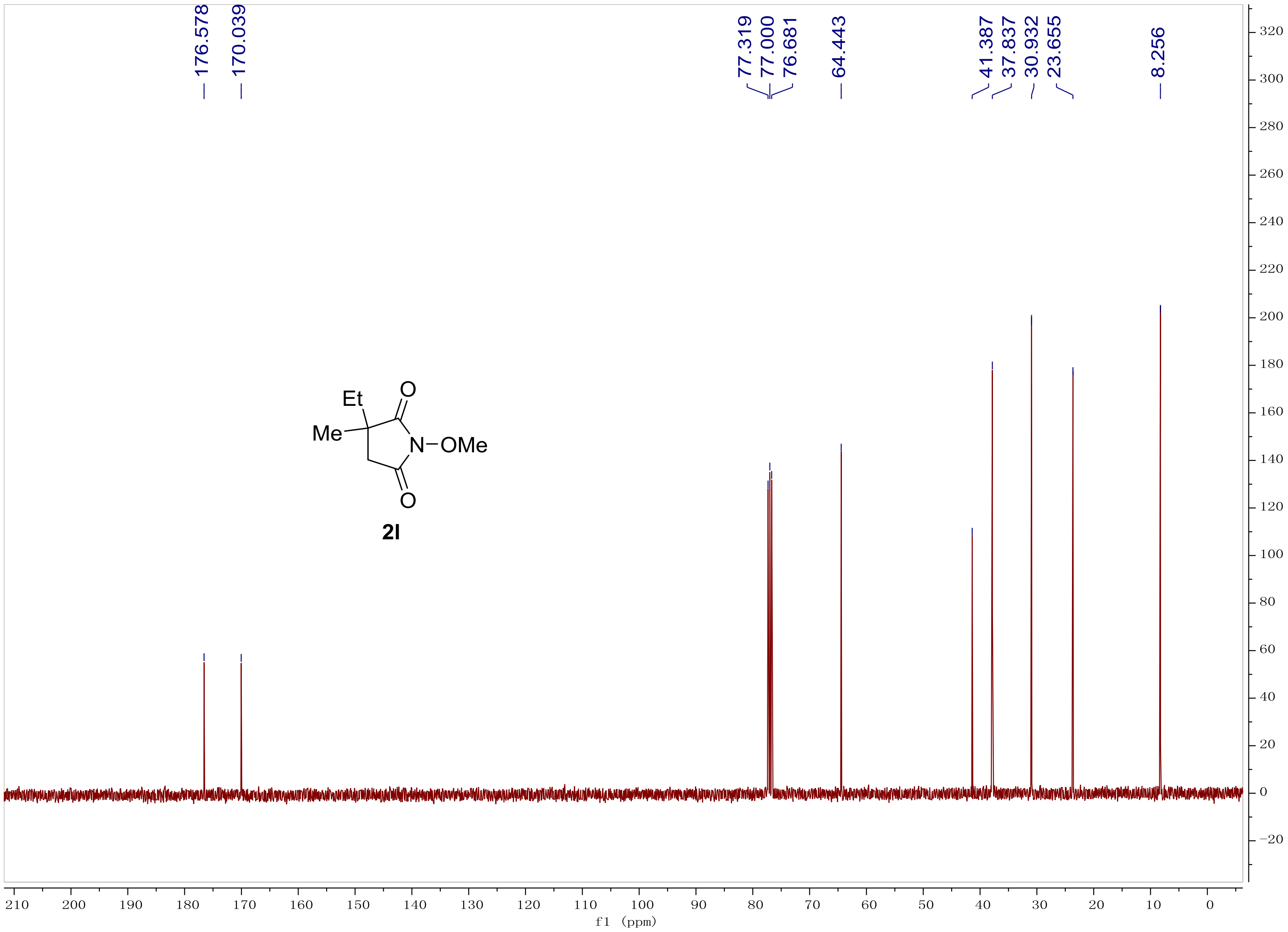

176.578
170.039
77.319
77.000
76.681
64.443
41.387
37.837
30.932
23.655
8.256
Et
O
Me
N−OMe
O
2l
f1 (ppm)

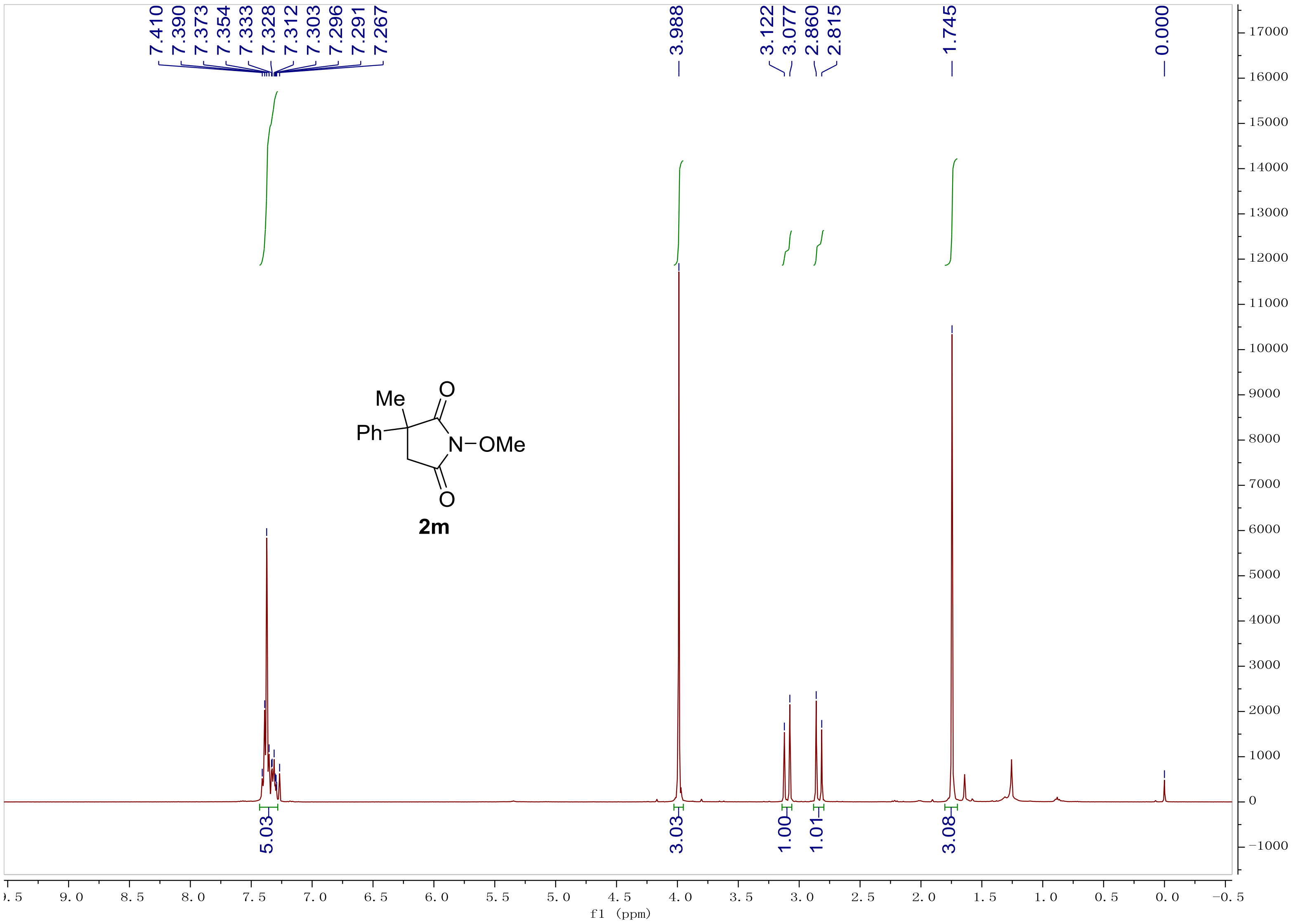
7.410
7.390
7.373
7.354
7.333
7.328
7.312
7.303
7.296
7.291
7.267
3.988
3.122
3.077
2.860
2.815
1.745
0.000
5.03
3.03
1.00
1.01
3.08
Me
Ph
N
OMe
O
2m
f1 (ppm)

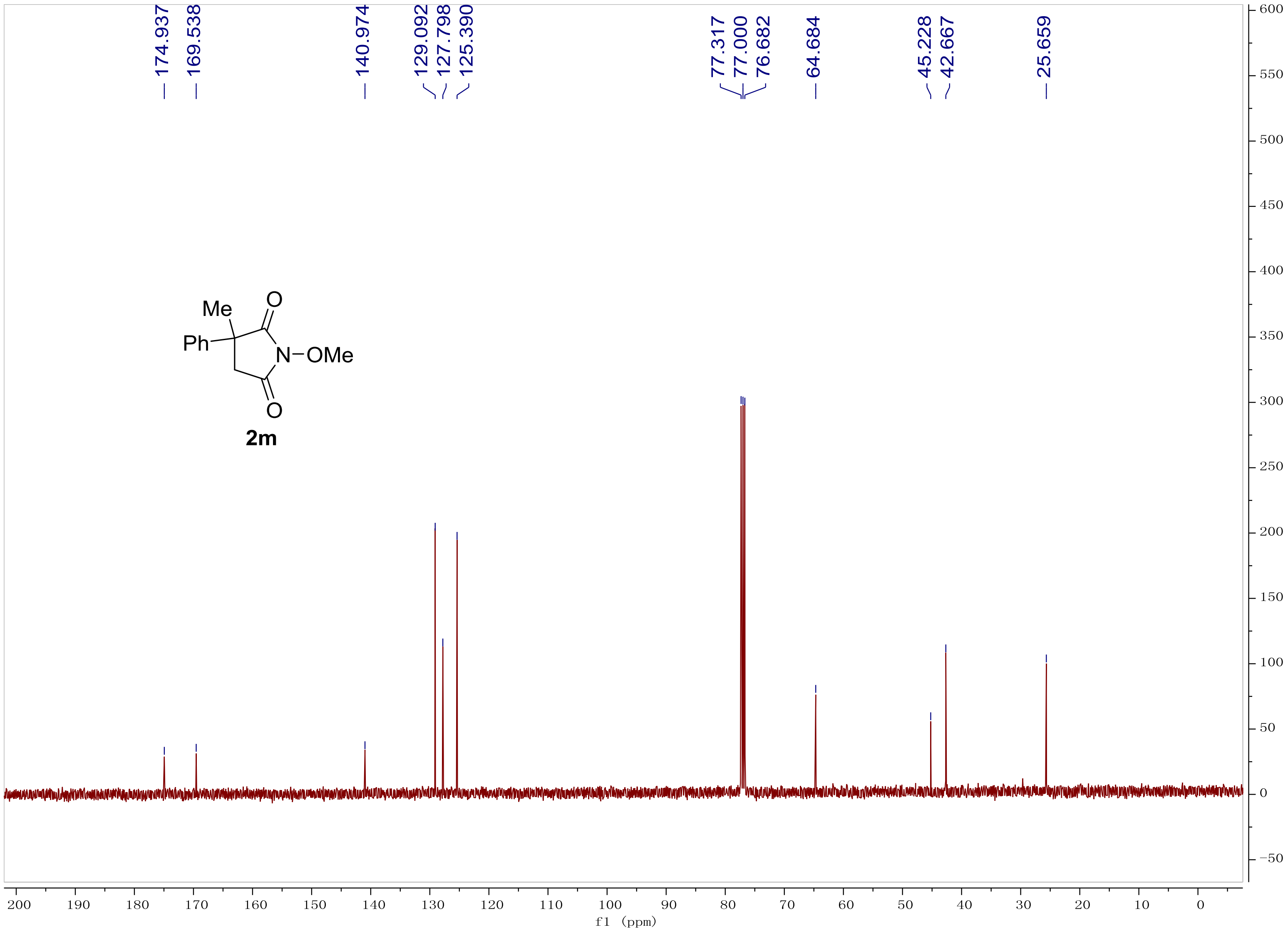
174.937
169.538
140.974
129.092
127.798
125.390
77.317
77.000
76.682
64.684
45.228
42.667
25.659
Me
Ph
N−OMe
O
O
f1 (ppm)

2m

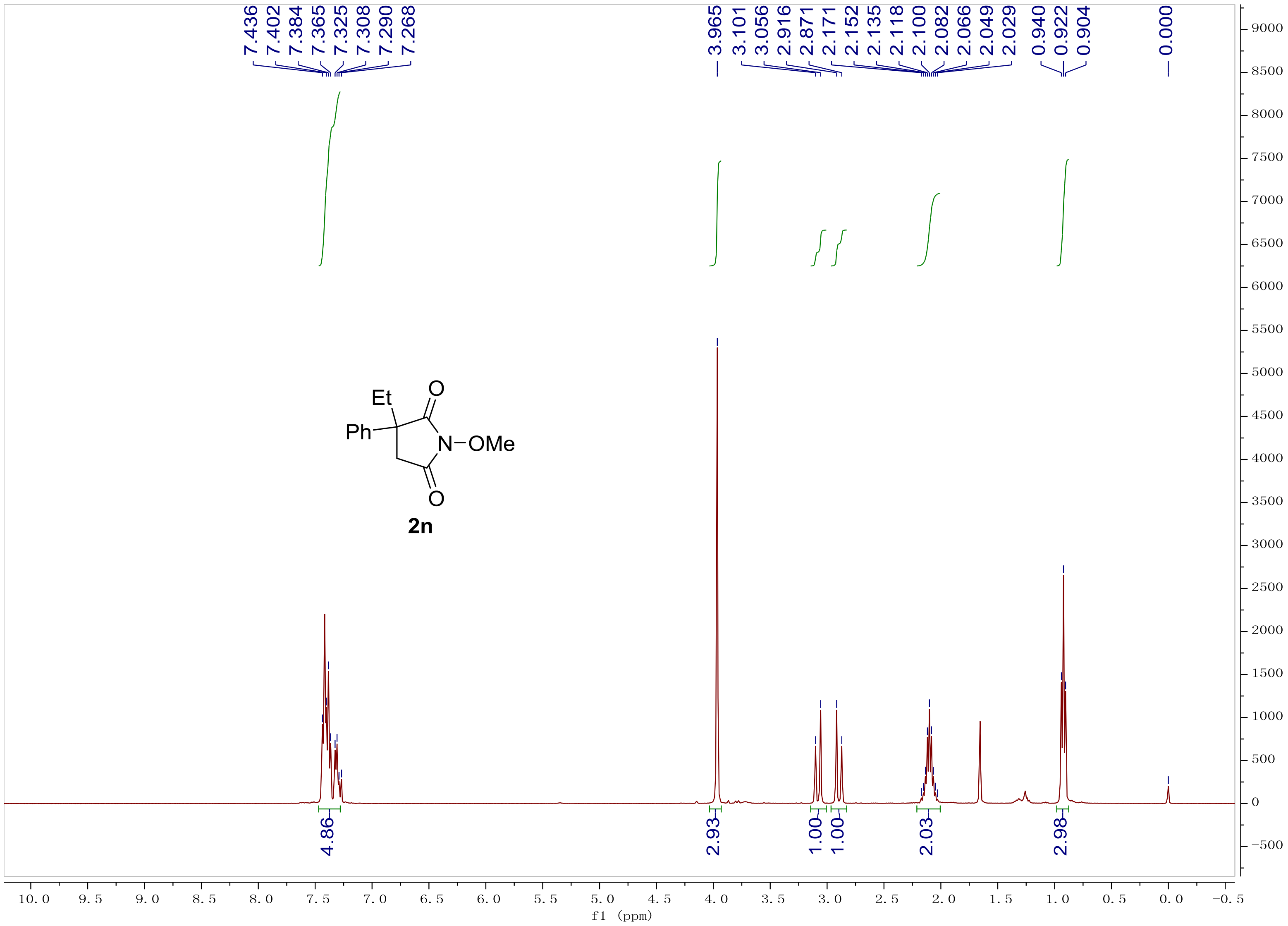

7.436
7.402
7.384
7.365
7.325
7.308
7.290
7.268
3.965
3.101
3.056
2.916
2.871
2.171
2.152
2.135
2.118
2.100
2.082
2.066
2.049
2.029
0.940
0.922
0.904
0.000
Et
O
Ph
N−OMe
O
2n
4.86
2.93
1.00
1.00
2.03
2.98
f1 (ppm)

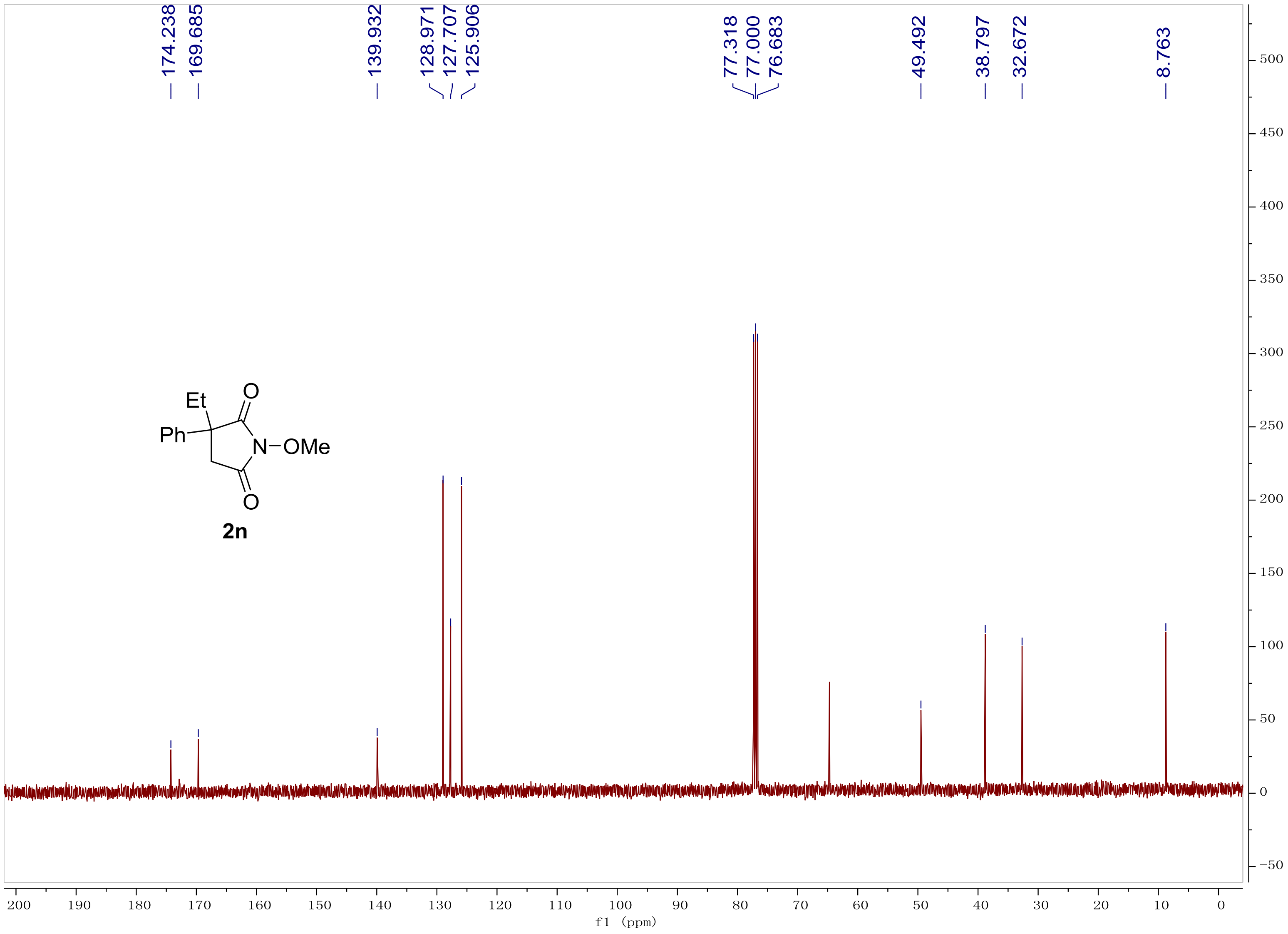
174.238
169.685
139.932
128.971
127.707
125.906
77.318
77.000
76.683
49.492
38.797
32.672
8.763
Et
O
Ph
N−OMe
O
200
190
180
170
160
150
140
130
120
110
100
90
80
70
60
50
40
30
20
10
0
f1 (ppm)
500
450
400
350
300
250
200
150
100
50
0
−50

2n

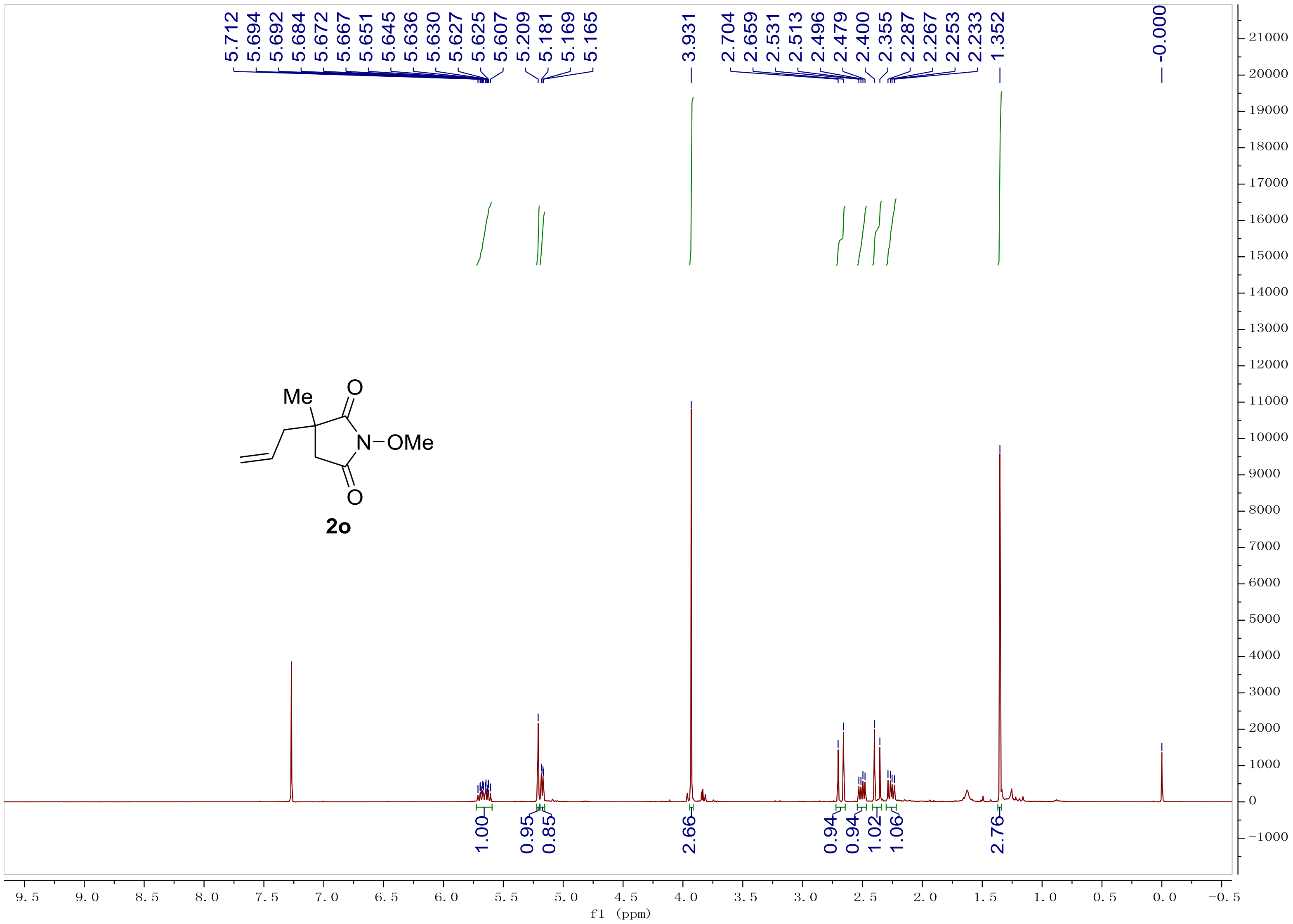
5.712
5.694
5.692
5.684
5.672
5.667
5.651
5.645
5.636
5.630
5.627
5.625
5.607
5.209
5.181
5.169
5.165
3.931
2.704
2.659
2.531
2.513
2.496
2.479
2.400
2.355
2.287
2.267
2.253
2.233
1.352
-0.000
Me
O
N−OMe
O
2o
1.00
0.95
0.85
2.66
0.94
0.94
1.02
1.06
2.76
f1 (ppm)

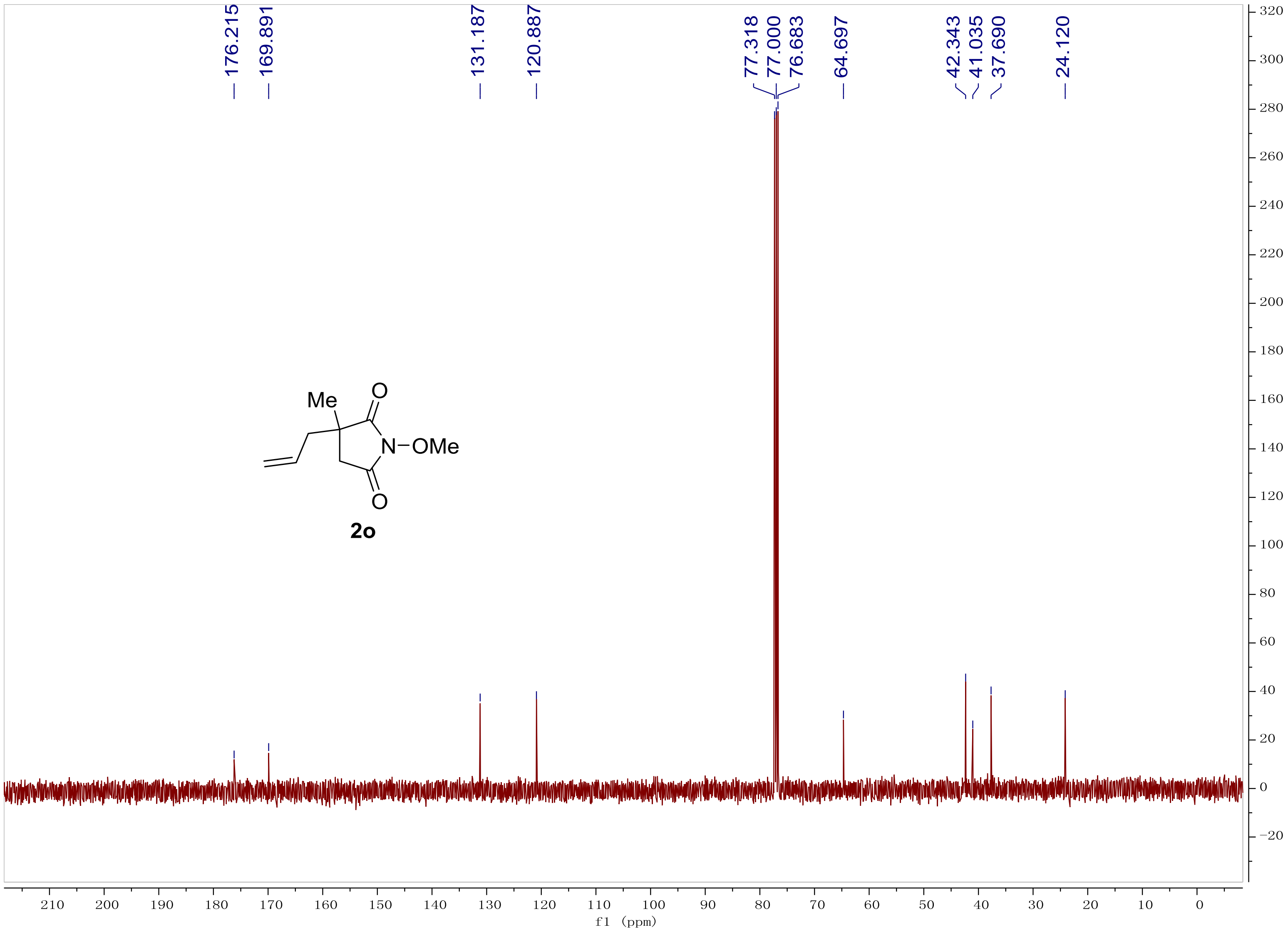
176.215
169.891
131.187
120.887
77.318
77.000
76.683
64.697
42.343
41.035
37.690
24.120
Me
O
N−OMe
O
2o
f1 (ppm)

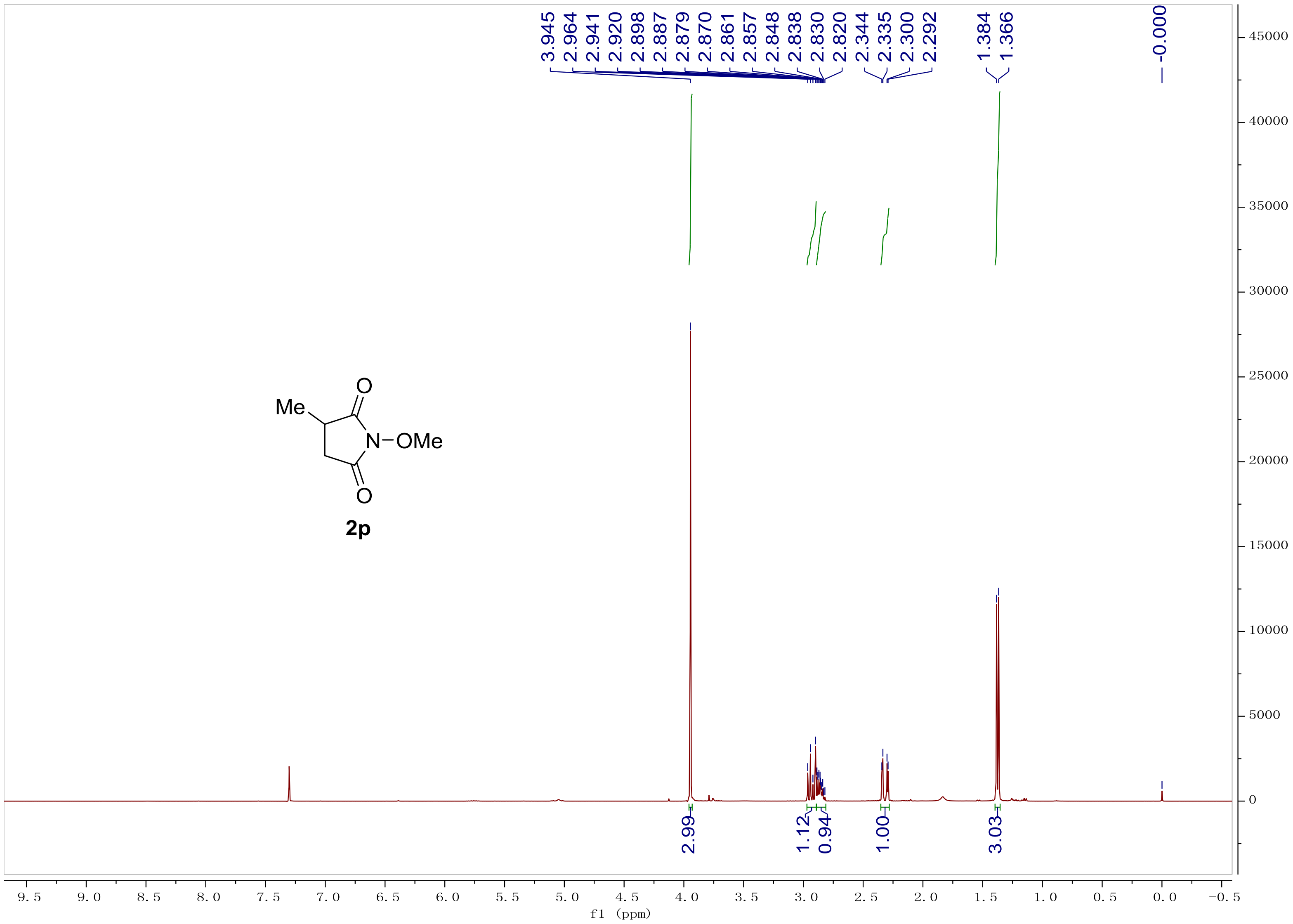
3.945 2.964 2.941 2.920 2.898 2.887 2.879 2.870 2.861 2.857 2.848 2.838 2.830 2.820 2.344 2.335 2.300 2.292 1.384 1.366 -0.000
Me
O
N−OMe
O
2p
2.99
1.12
0.94
1.00
3.03
45000
40000
35000
30000
25000
20000
15000
10000
5000
0
9.5 9.0 8.5 8.0 7.5 7.0 6.5 6.0 5.5 5.0 4.5 4.0 3.5 3.0 2.5 2.0 1.5 1.0 0.5 0.0 −0.5
f1 (ppm)

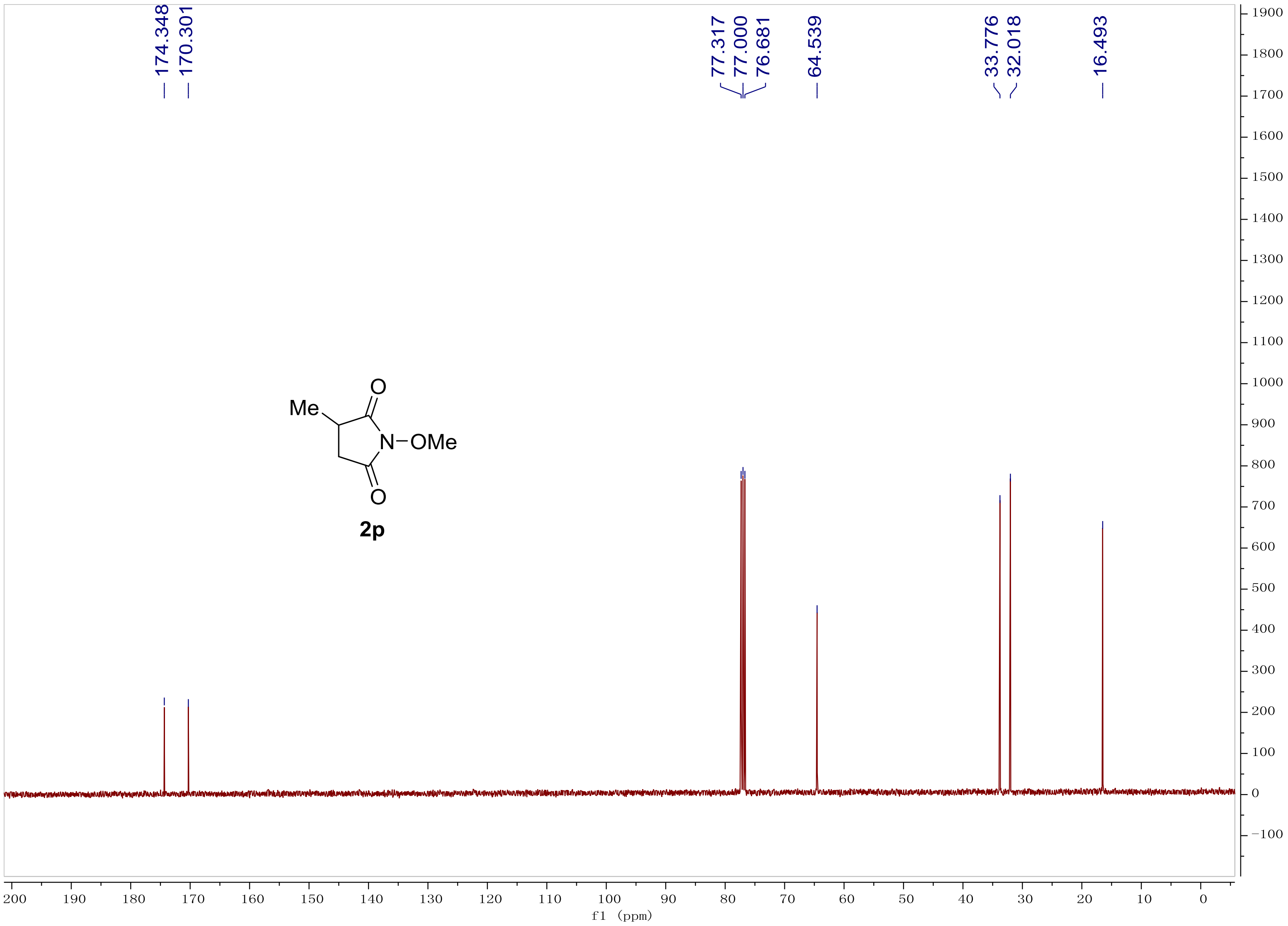
174.348
170.301
77.317
77.000
76.681
64.539
33.776
32.018
16.493
Me
O
N−OMe
O
2p
f1 (ppm)

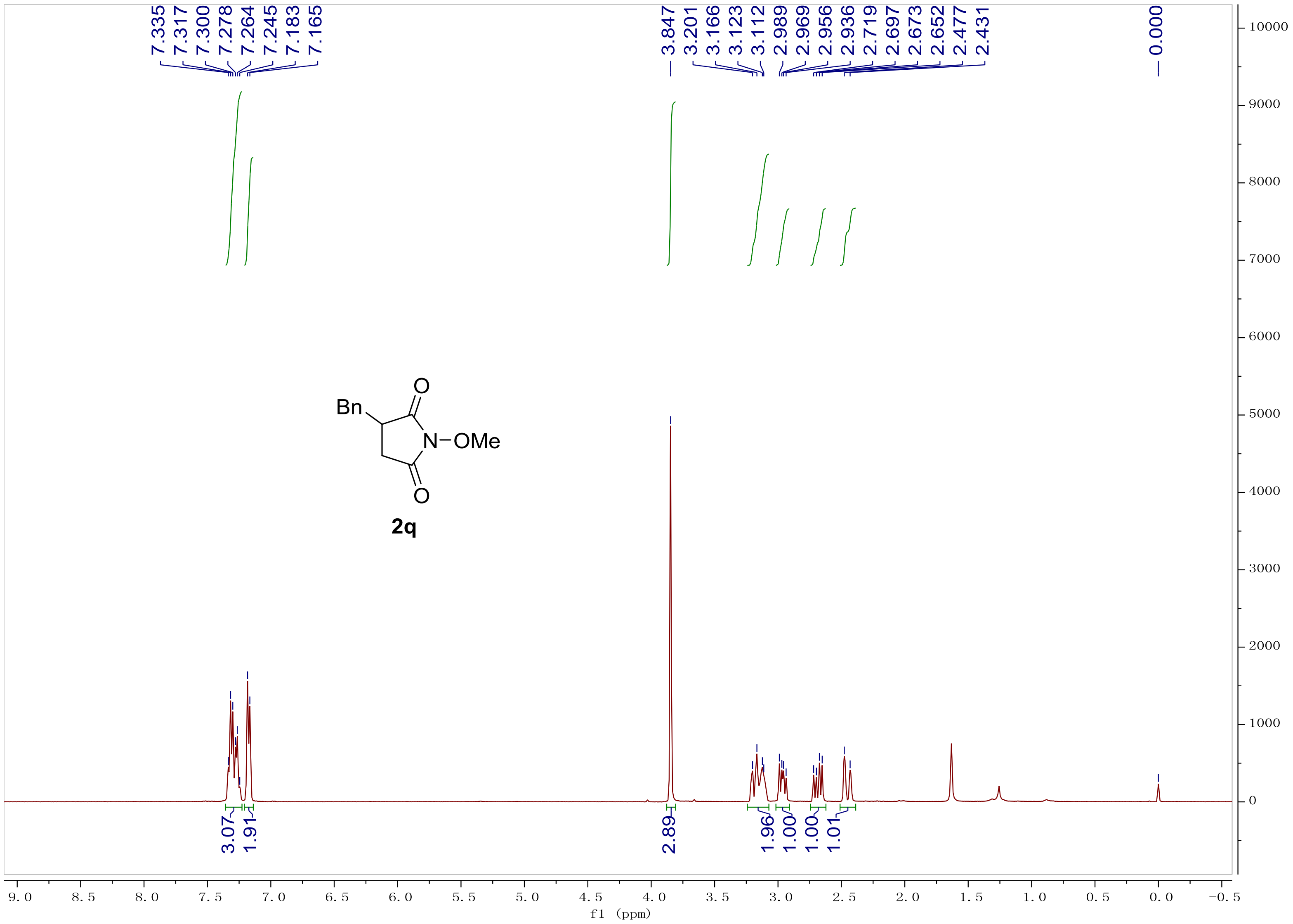
7.335
7.317
7.300
7.278
7.264
7.245
7.183
7.165
3.847
3.201
3.166
3.123
3.112
2.989
2.969
2.956
2.936
2.719
2.697
2.673
2.652
2.477
2.431
0.000
Bn
O
N−OMe
O
2q
3.07
1.91
2.89
1.96
1.00
1.00
1.01
f1 (ppm)

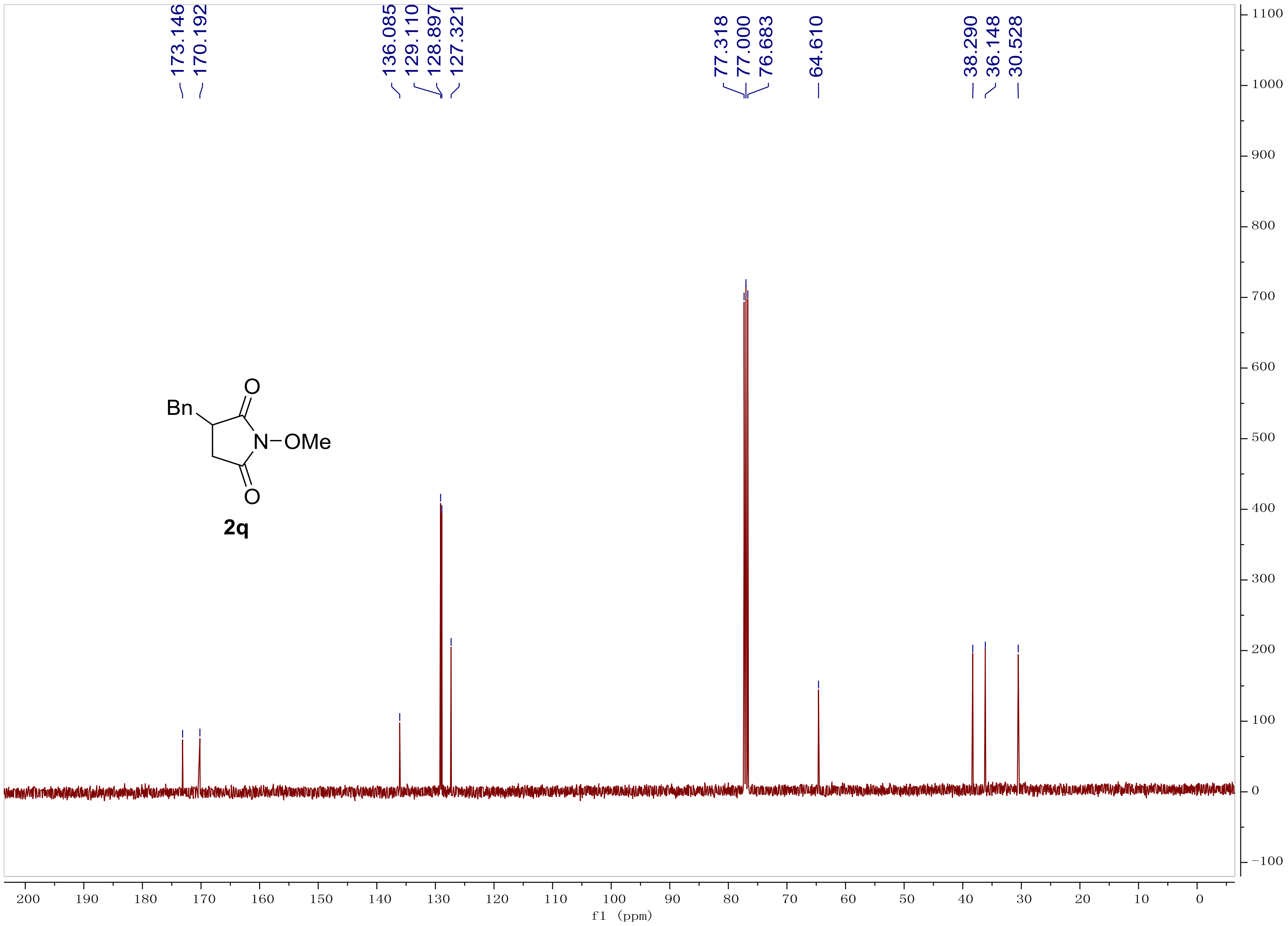
173.146
170.192
136.085
129.110
128.897
127.321
77.318
77.000
76.683
64.610
38.290
36.148
30.528
Bn
N−OMe
O
O
2q
f1 (ppm)

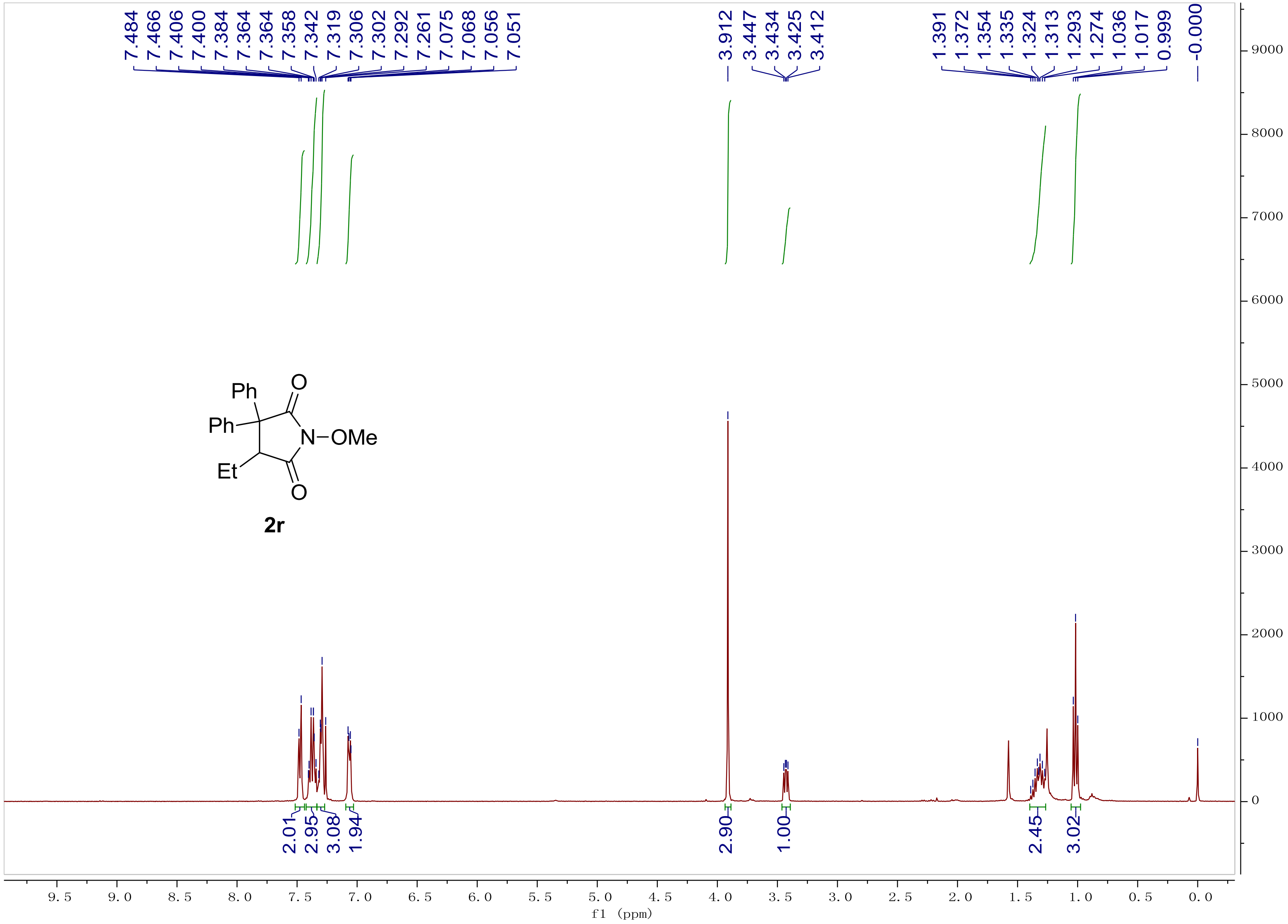

7.484
7.466
7.406
7.400
7.384
7.364
7.364
7.358
7.342
7.319
7.306
7.302
7.292
7.261
7.075
7.068
7.056
7.051
3.912
3.447
3.434
3.425
3.412
1.391
1.372
1.354
1.335
1.324
1.313
1.293
1.274
1.036
1.017
0.999
-0.000
Ph
Ph
O
N-OMe
Et
O
2r
2.01
2.95
3.08
1.94
2.90
1.00
2.45
3.02
f1 (ppm)

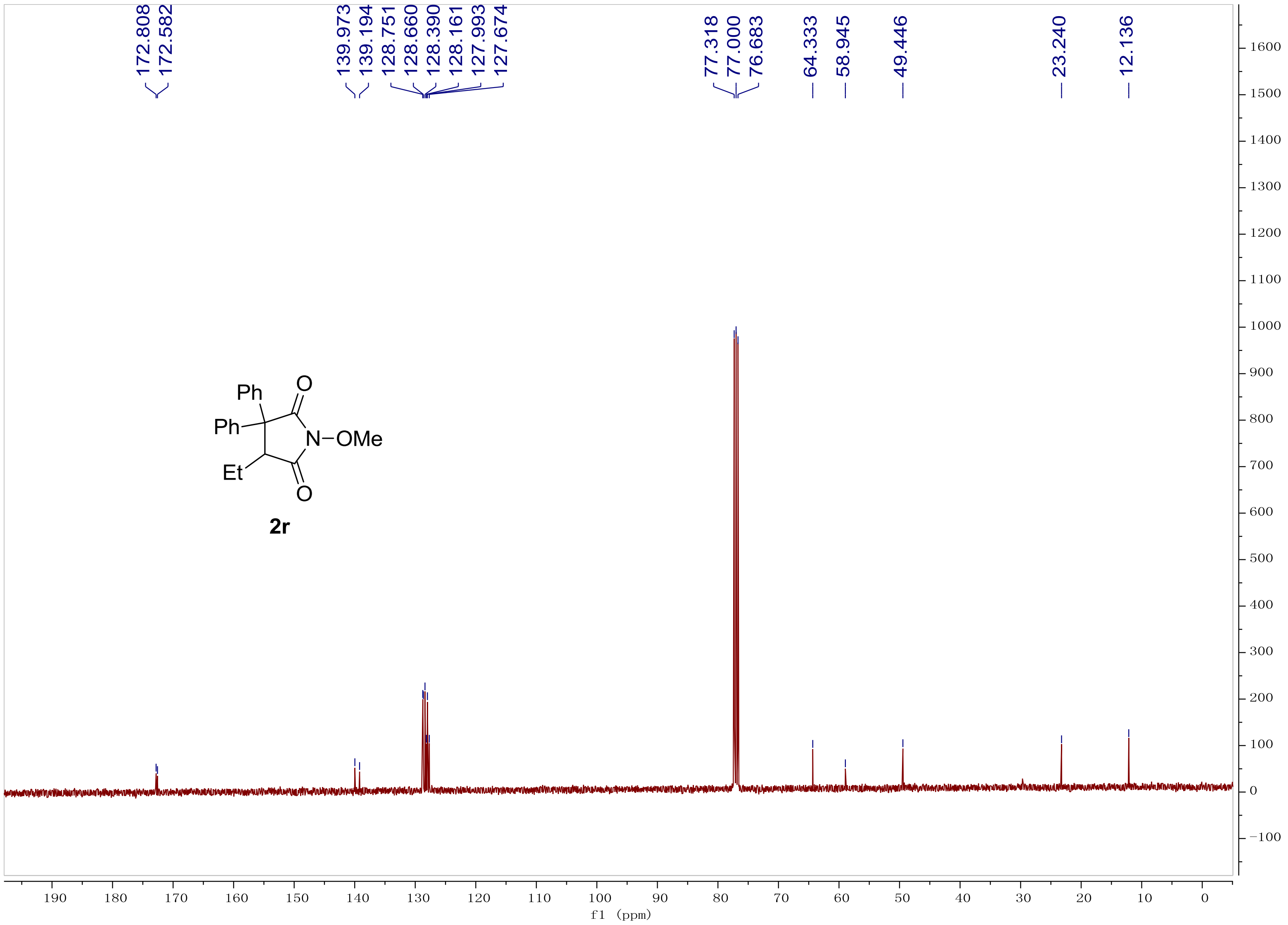
172.808
172.582
139.973
139.194
128.751
128.660
128.390
128.161
127.993
127.674
77.318
77.000
76.683
64.333
58.945
49.446
23.240
12.136
Ph
Ph
O
N−OMe
Et
O
2r
f1 (ppm)

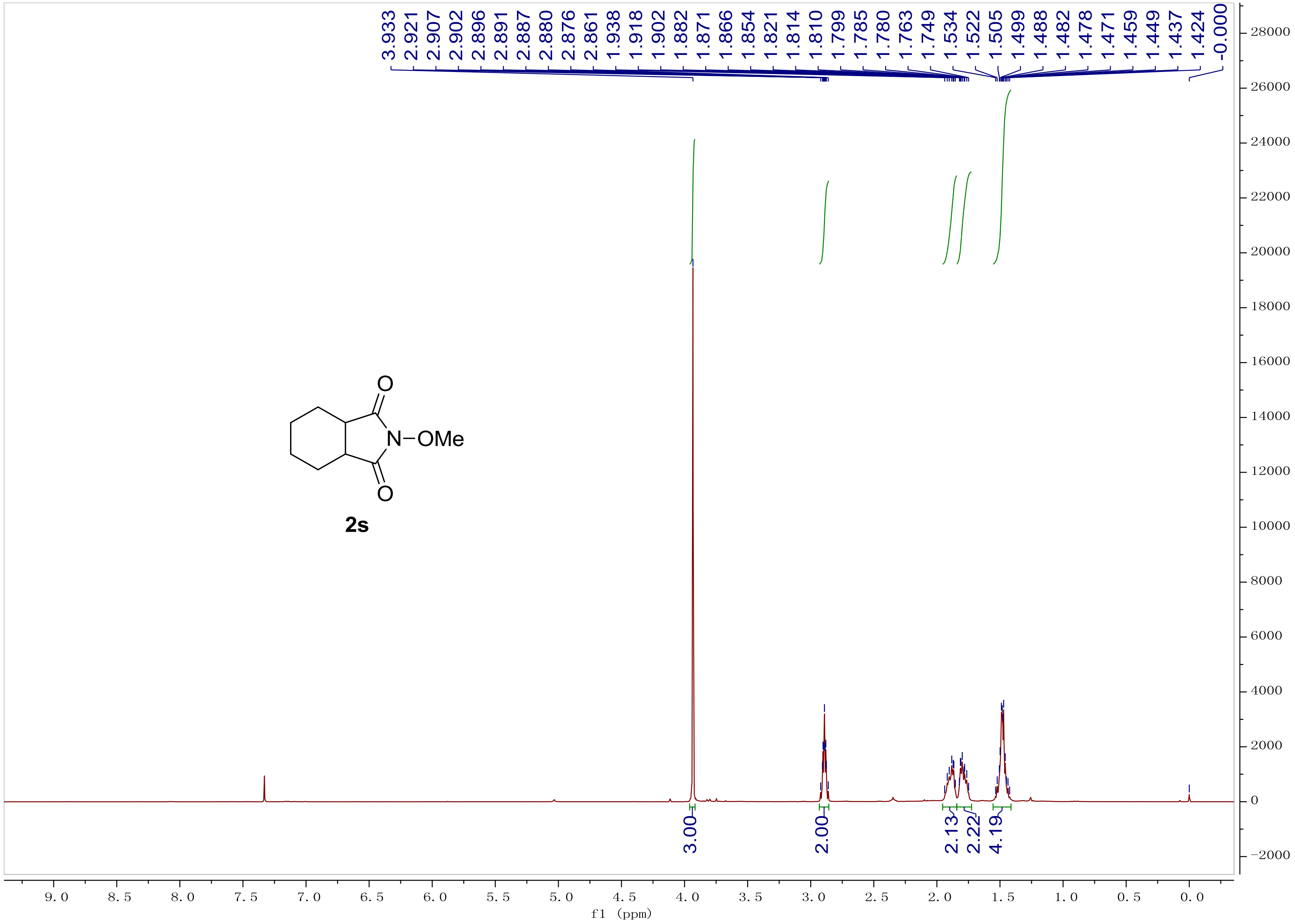
3.933
2.921
2.907
2.902
2.896
2.891
2.887
2.880
2.876
2.861
1.938
1.918
1.902
1.882
1.871
1.866
1.854
1.821
1.814
1.810
1.799
1.785
1.780
1.763
1.749
1.534
1.522
1.505
1.499
1.488
1.482
1.478
1.471
1.459
1.449
1.437
1.424
-0.000
N−OMe
O
O
2s
3.00
2.00
2.13
2.22
4.19
f1 (ppm)

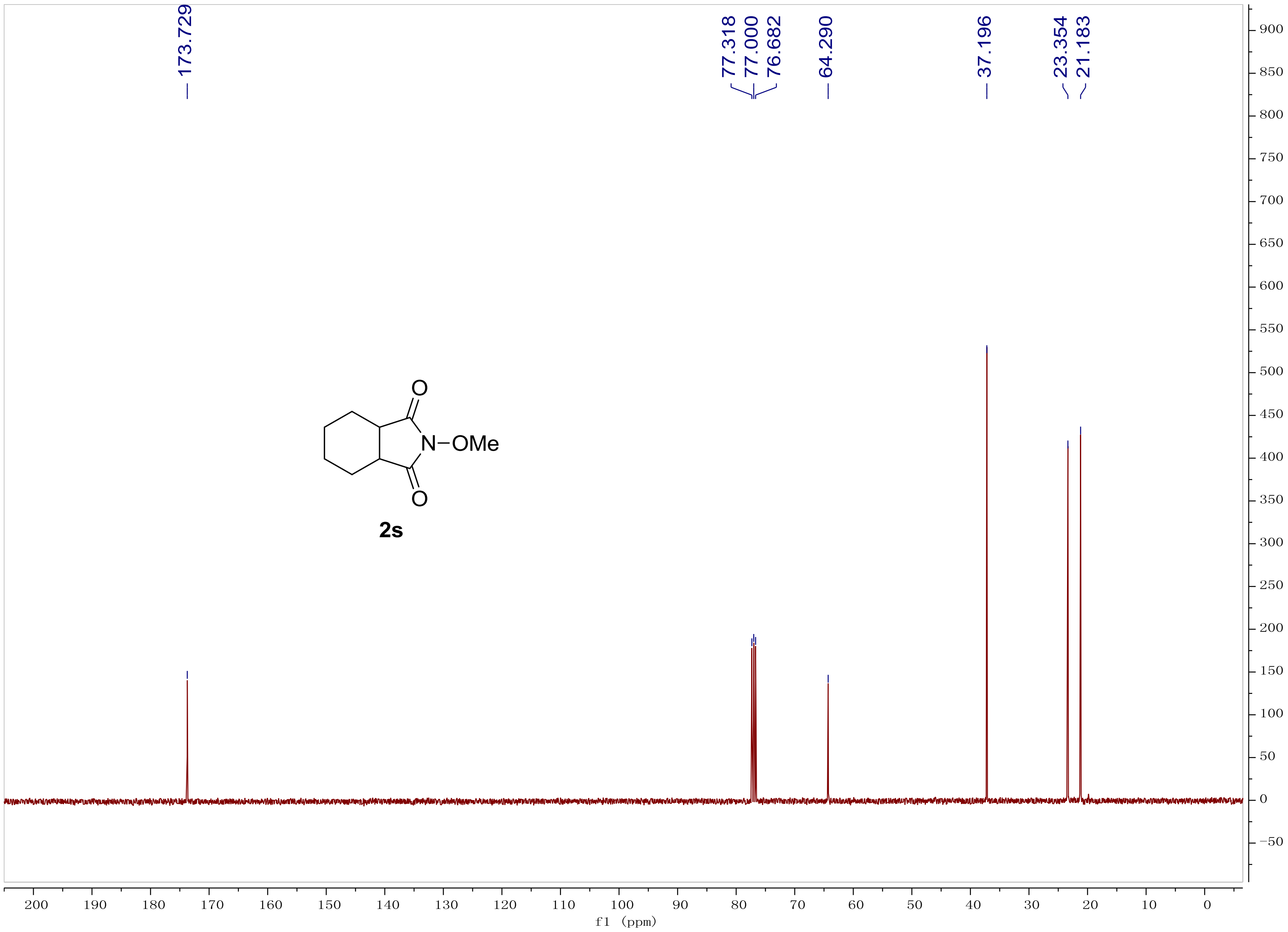
173.729
77.318
77.000
76.682
64.290
37.196
23.354
21.183
N–OMe
O
2s
f1 (ppm)

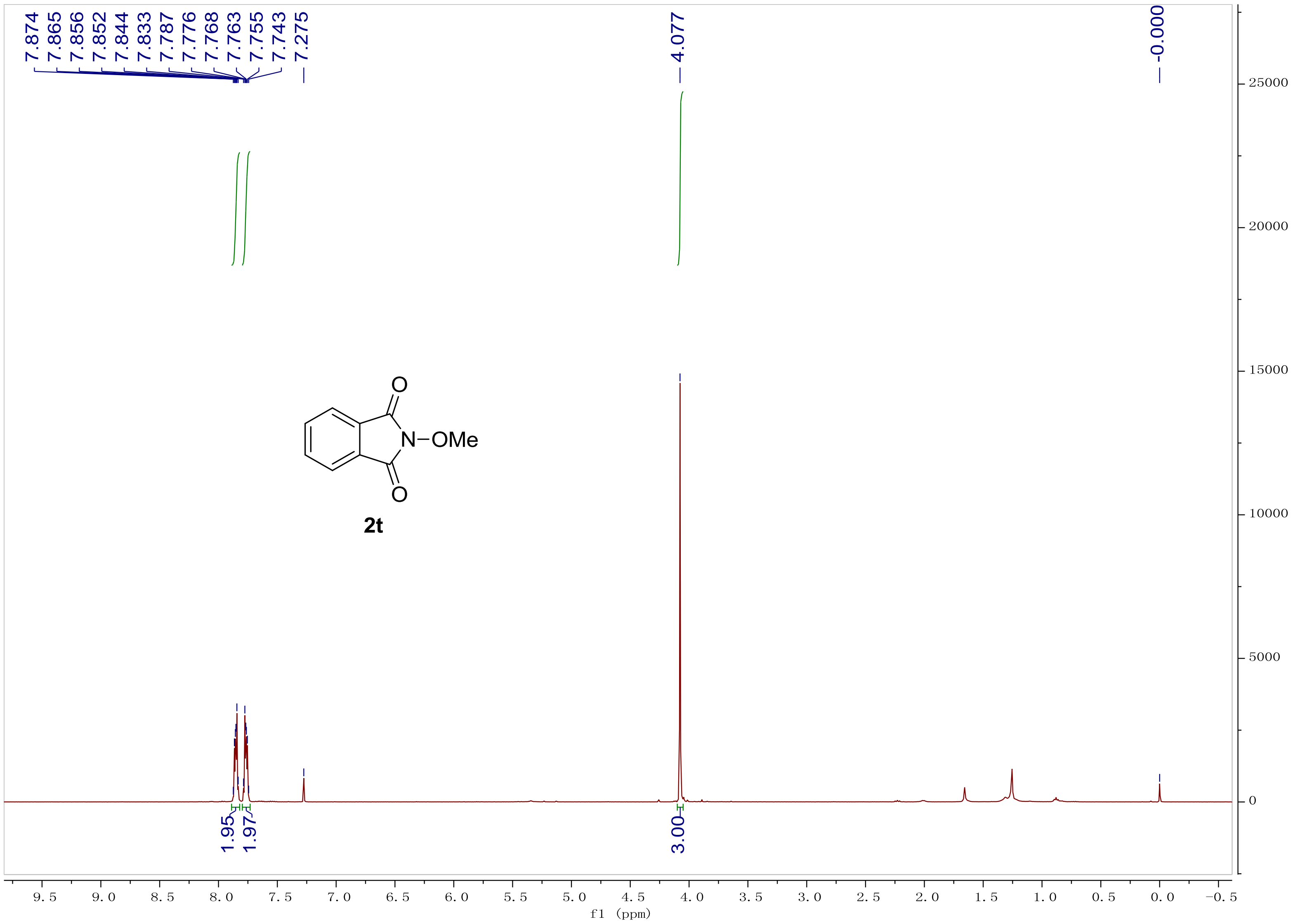
7.874
7.865
7.856
7.852
7.844
7.833
7.787
7.776
7.768
7.763
7.755
7.743
7.275
4.077
-0.000
25000
20000
15000
10000
5000
0
O
N−OMe
O
2t
1.95
1.97
3.00
9.5
9.0
8.5
8.0
7.5
7.0
6.5
6.0
5.5
5.0
4.5
4.0
3.5
3.0
2.5
2.0
1.5
1.0
0.5
0.0
-0.5
f1 (ppm)

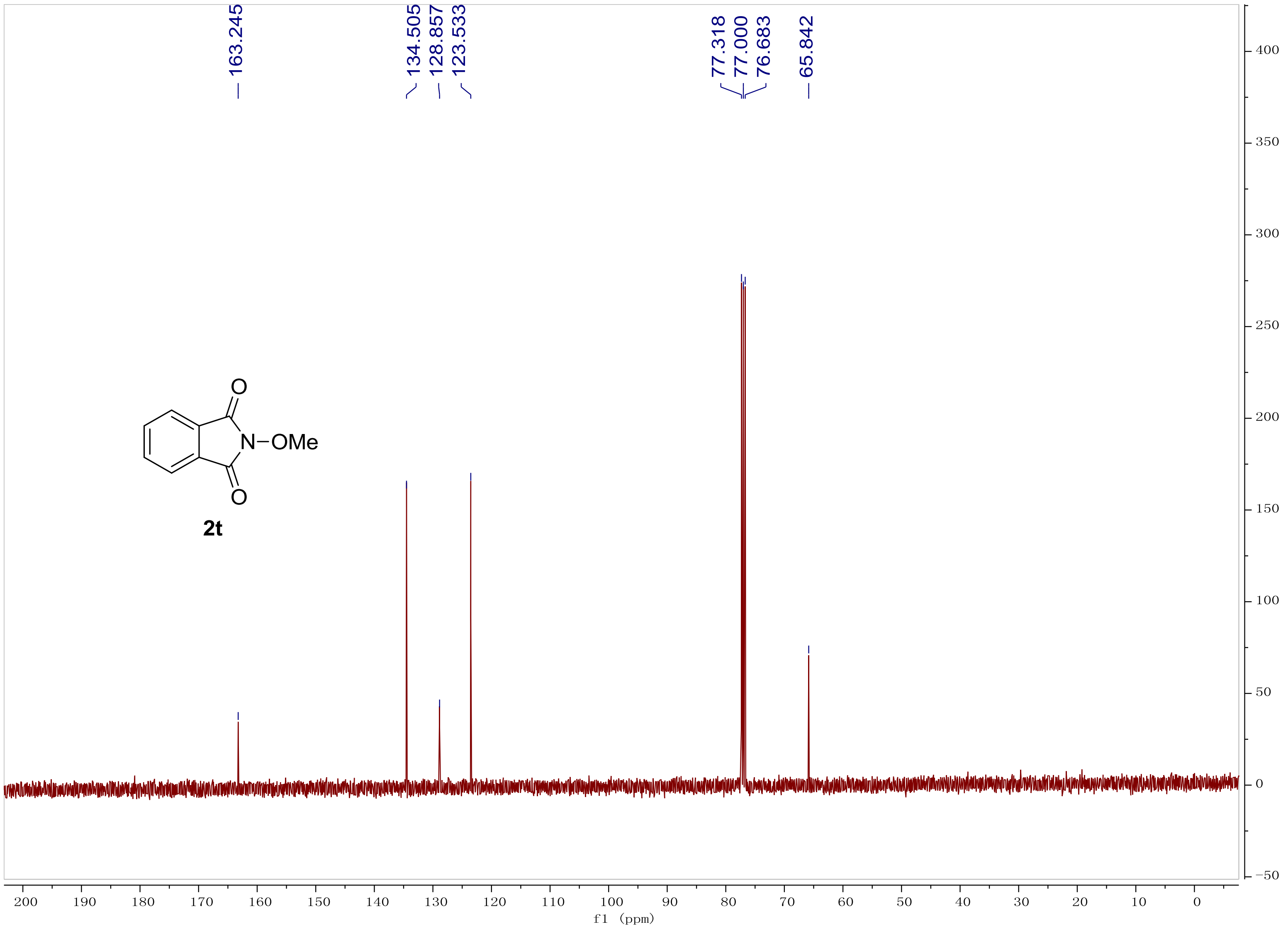
163.245
134.505
128.857
123.533
77.318
77.000
76.683
65.842
N−OMe
O
O
2t
400
350
300
250
200
150
100
50
0
−50
200
190
180
170
160
150
140
130
120
110
100
90
80
70
60
50
40
30
20
10
0
f1 (ppm)

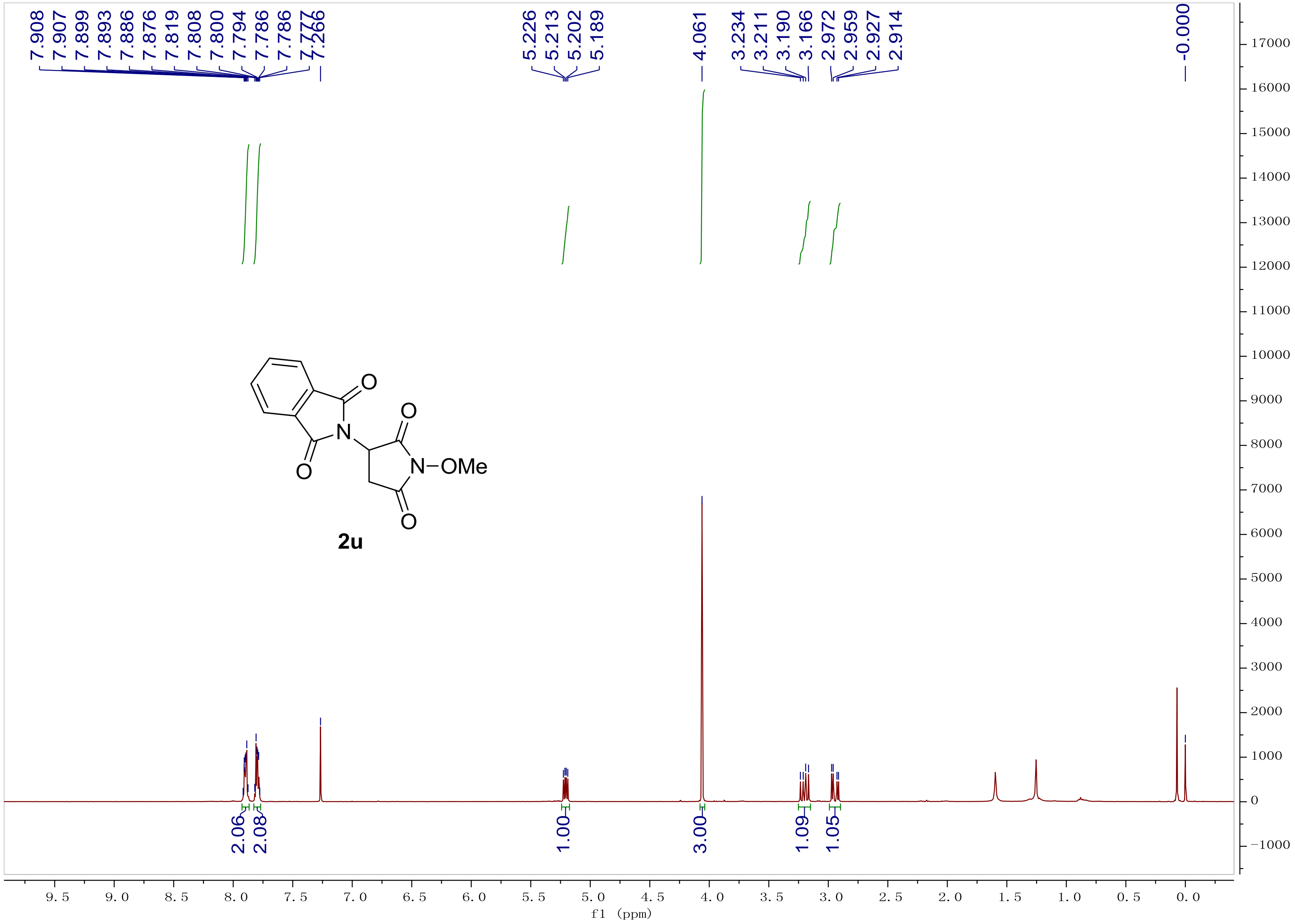
7.908
7.907
7.899
7.893
7.886
7.876
7.819
7.808
7.800
7.794
7.786
7.786
7.777
7.266
5.226
5.213
5.202
5.189
4.061
3.234
3.211
3.190
3.166
2.972
2.959
2.927
2.914
-0.000
O
O
N
O
O
N–OMe
2u
2.06
2.08
1.00
3.00
1.09
1.05
f1 (ppm)

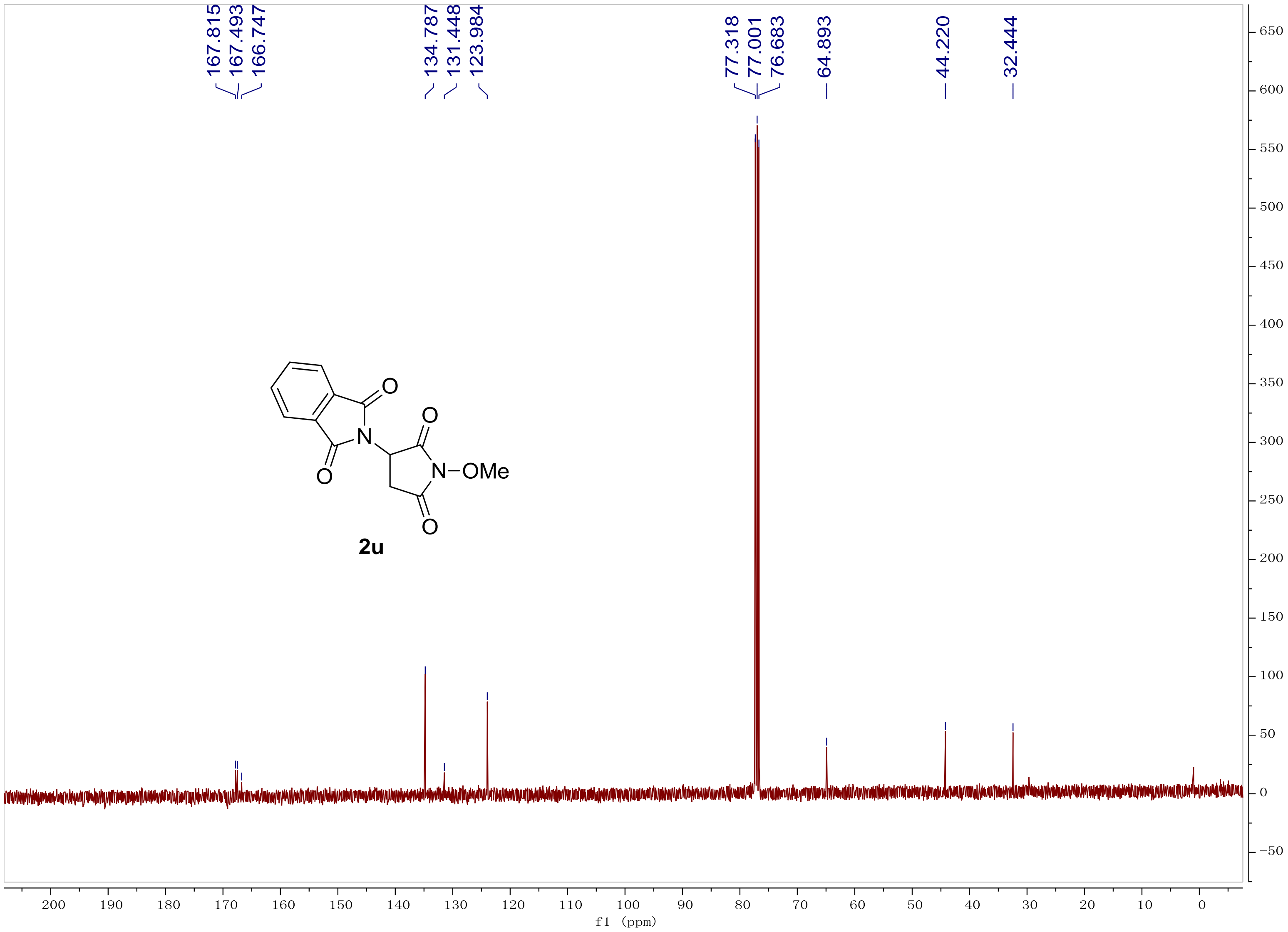
167.815
167.493
166.747
134.787
131.448
123.984
77.318
77.001
76.683
64.893
44.220
32.444
N
O
O
O
O
N–OMe
2u
650
600
550
500
450
400
350
300
250
200
150
100
50
0
−50
200
190
180
170
160
150
140
130
120
110
100
90
80
70
60
50
40
30
20
10
0
f1 (ppm)

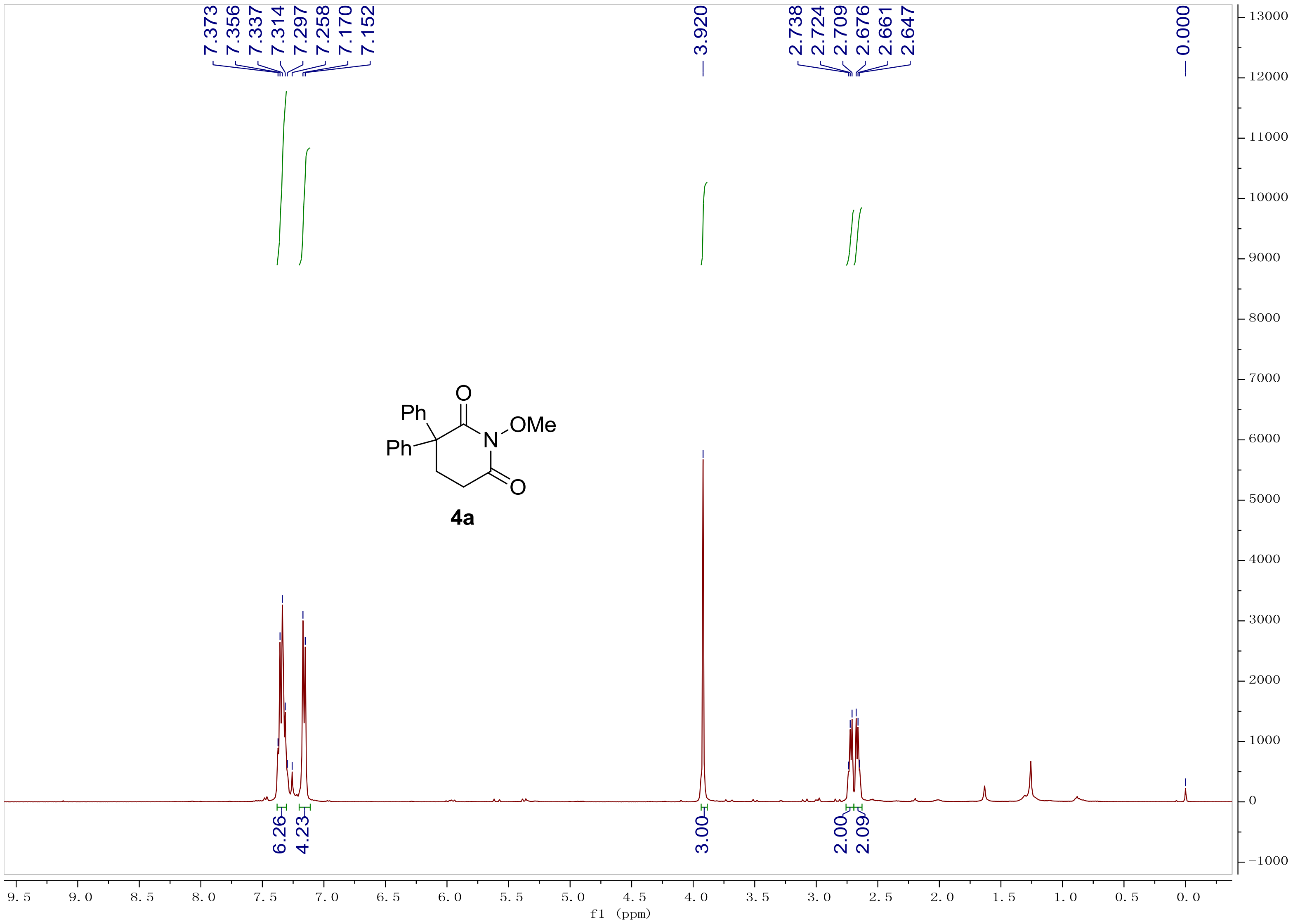
7.373
7.356
7.337
7.314
7.297
7.258
7.170
7.152
3.920
2.738
2.724
2.709
2.676
2.661
2.647
0.000
Ph
Ph
O
N
OMe
O
4a
6.26
4.23
3.00
2.00
2.09
f1 (ppm)

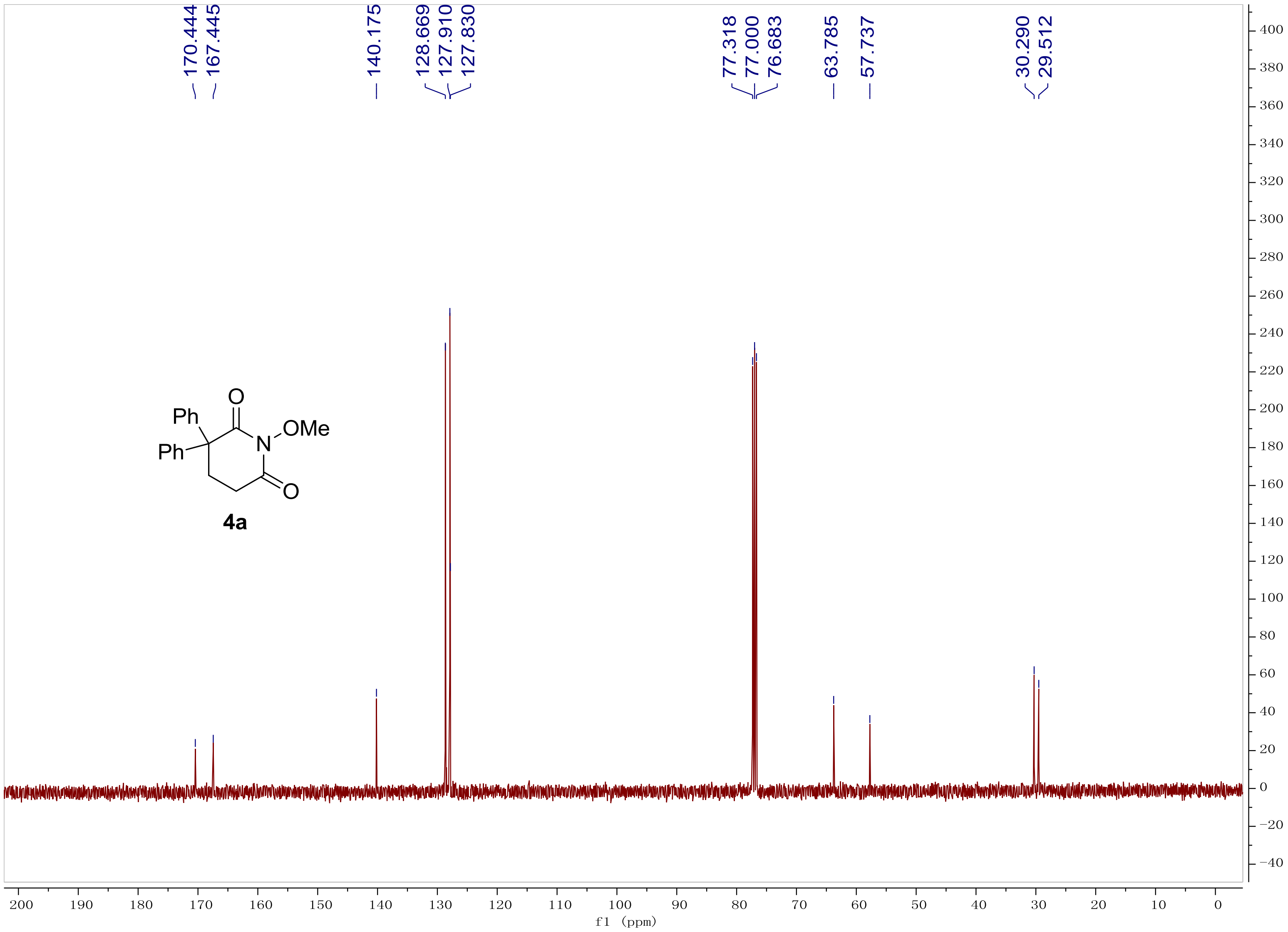

170.444
167.445
140.175
128.669
127.910
127.830
77.318
77.000
76.683
63.785
57.737
30.290
29.512
Ph
Ph
O
N
OMe
O
4a
f1 (ppm)

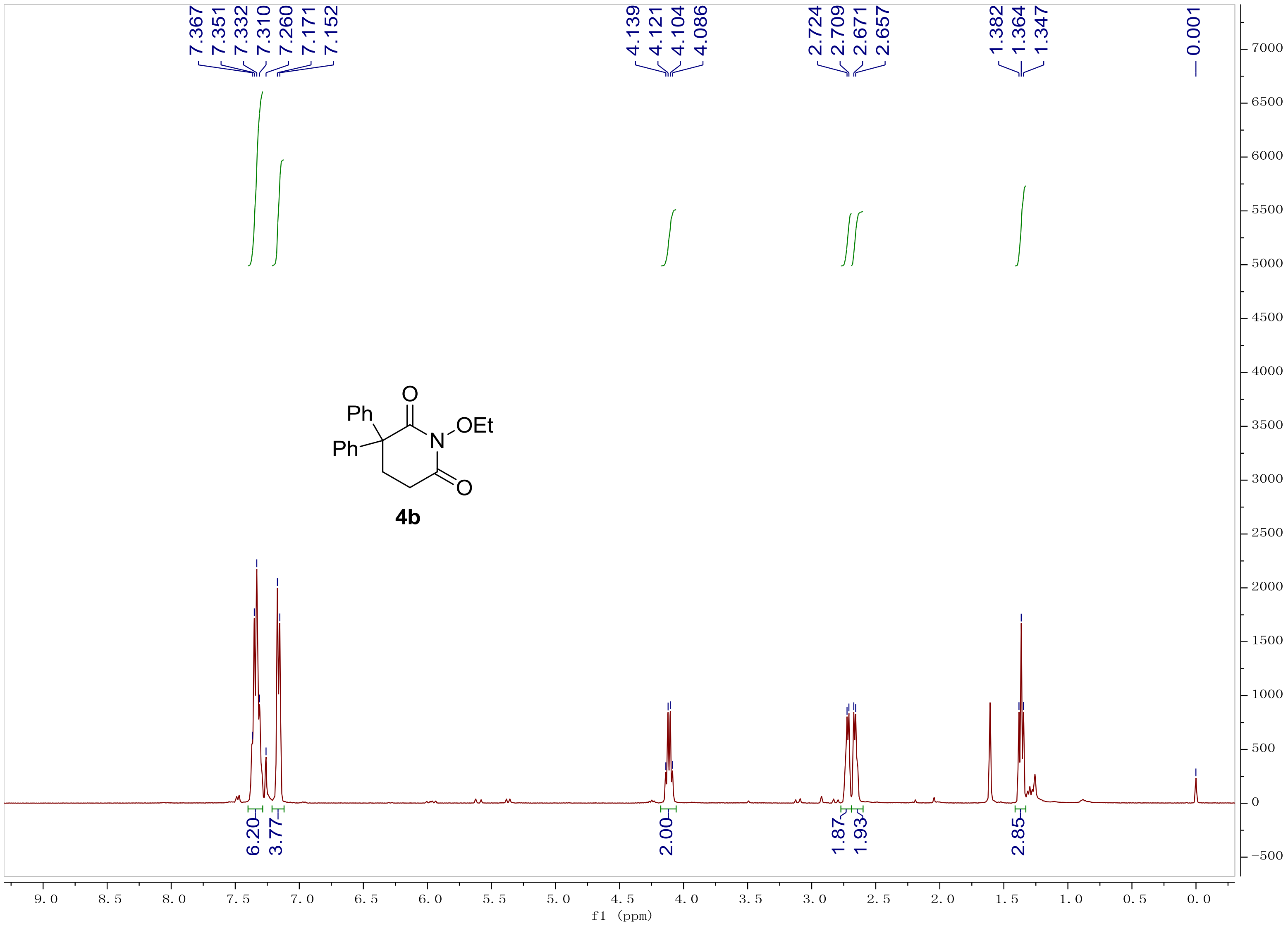
7.367
7.351
7.332
7.310
7.260
7.171
7.152
4.139
4.121
4.104
4.086
2.724
2.709
2.671
2.657
1.382
1.364
1.347
0.001
Ph
Ph
O
N
OEt
O
6.20
3.77
2.00
1.87
1.93
2.85
f1 (ppm)

4b

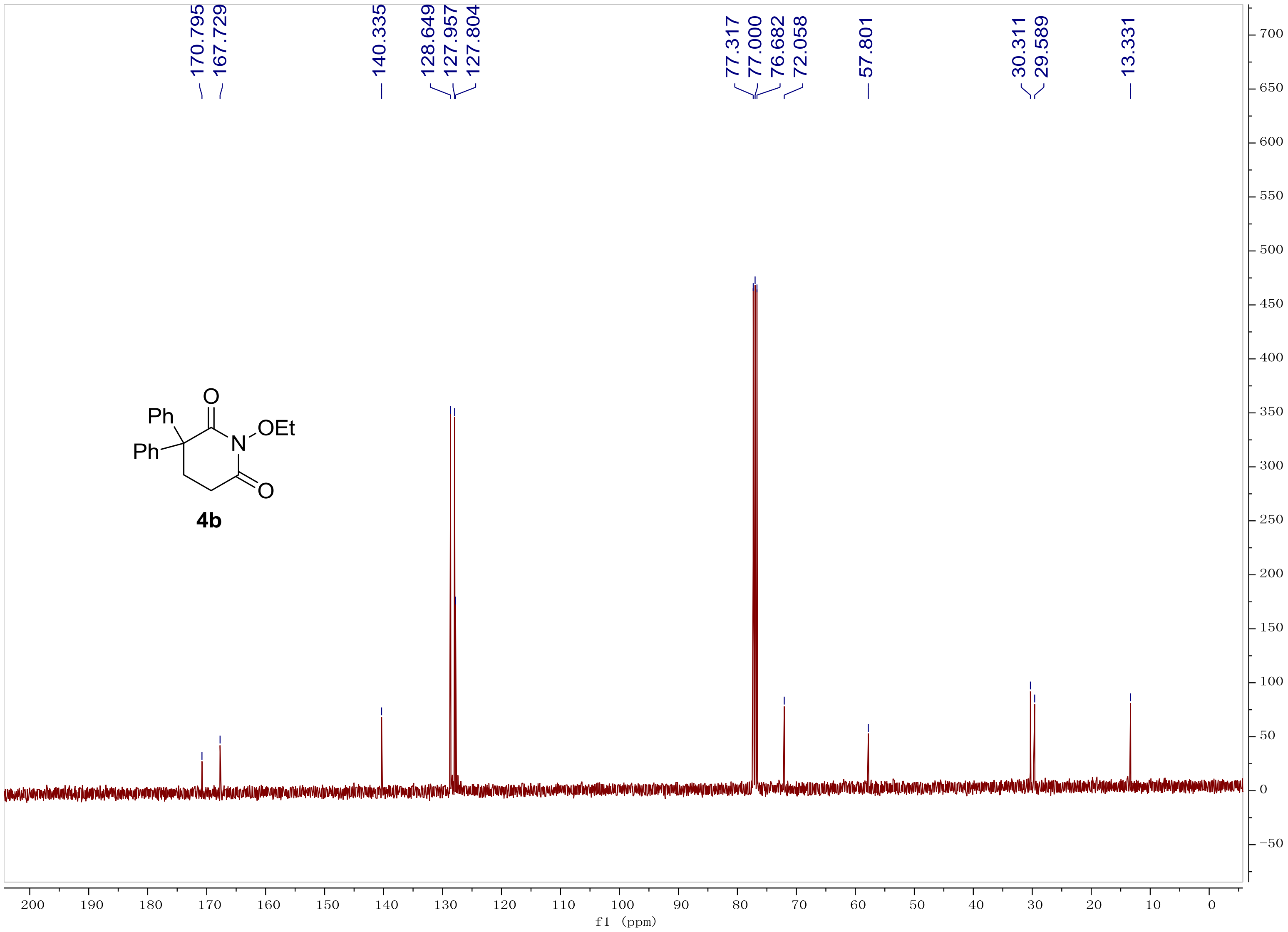

170.795
167.729
140.335
128.649
127.957
127.804
77.317
77.000
76.682
72.058
57.801
30.311
29.589
13.331
Ph
Ph
O
O
N
OEt
f1 (ppm)

4b

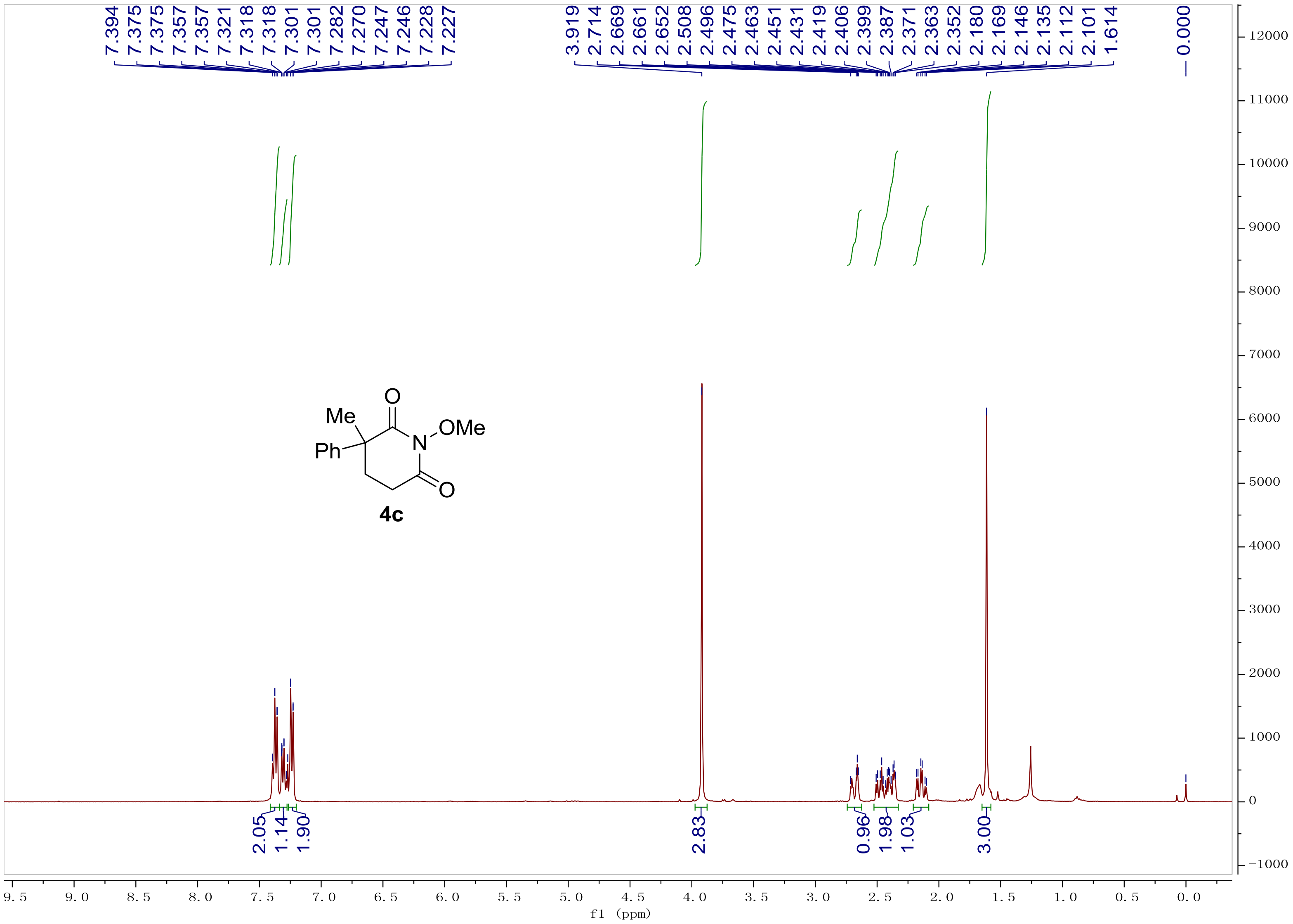
7.394
7.375
7.375
7.357
7.357
7.321
7.318
7.318
7.301
7.301
7.282
7.270
7.247
7.246
7.228
7.227
3.919
2.714
2.669
2.661
2.652
2.508
2.496
2.475
2.463
2.451
2.431
2.419
2.406
2.399
2.387
2.371
2.363
2.352
2.180
2.169
2.146
2.135
2.112
2.101
1.614
0.000
Me
O
N
OMe
Ph
O
2.05
1.14
1.90
2.83
0.96
1.98
1.03
3.00
f1 (ppm)
4c

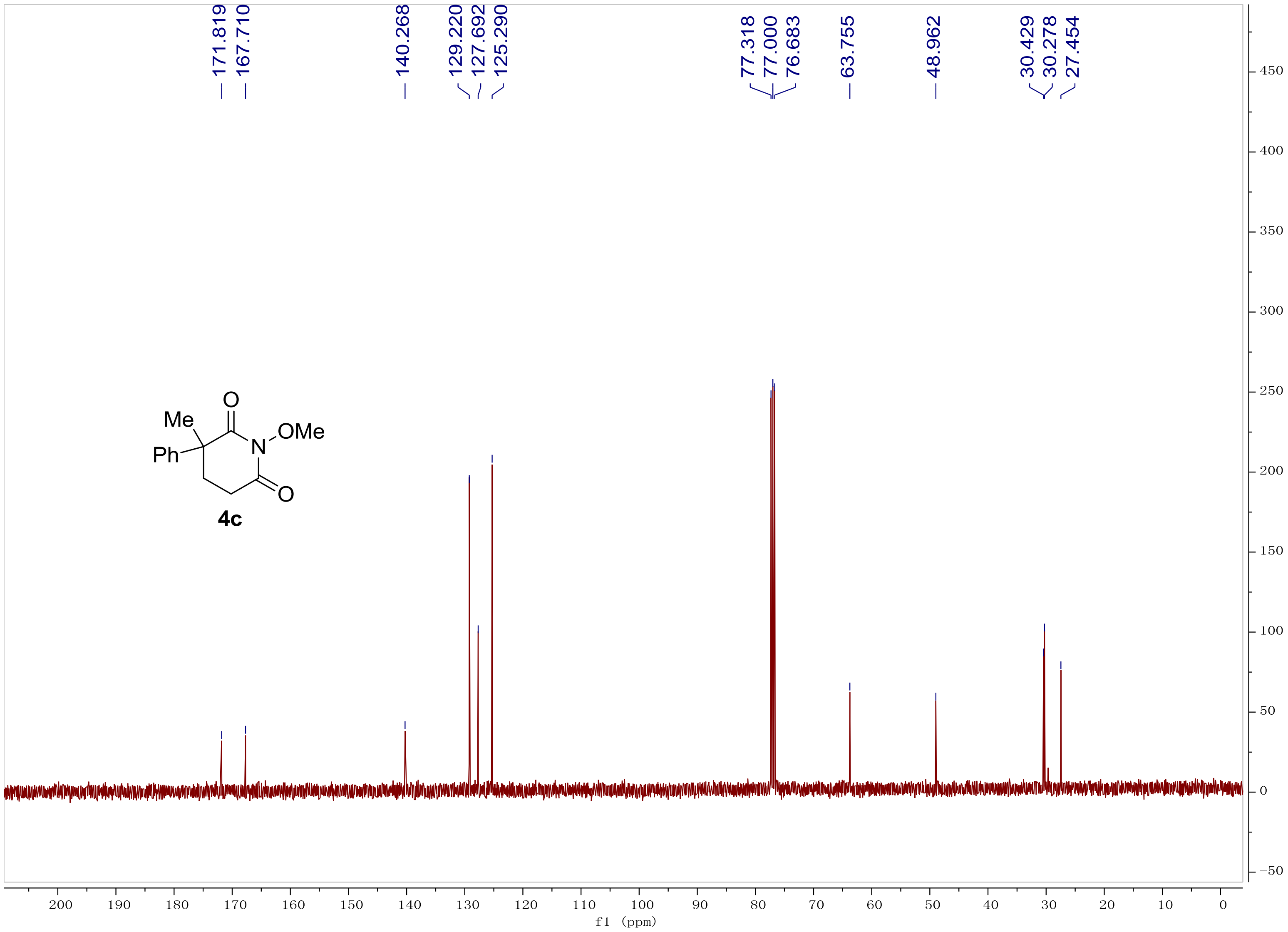
171.819
167.710
140.268
129.220
127.692
125.290
77.318
77.000
76.683
63.755
48.962
30.429
30.278
27.454
Me
O
OMe
N
Ph
O
4c
f1 (ppm)

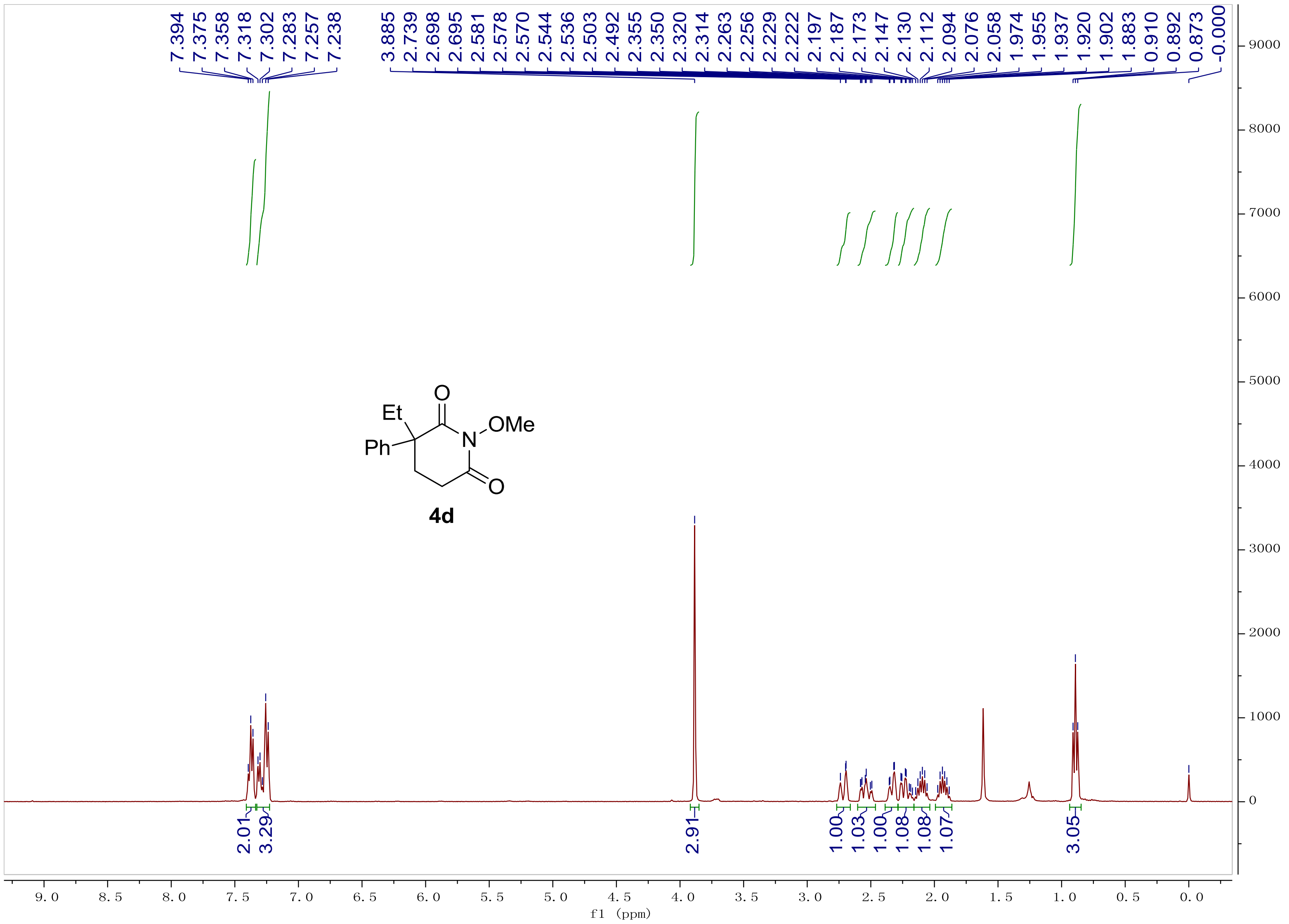

7.394
7.375
7.358
7.318
7.302
7.283
7.257
7.238
3.885
2.739
2.698
2.695
2.581
2.578
2.570
2.544
2.536
2.503
2.492
2.355
2.350
2.320
2.314
2.263
2.256
2.229
2.222
2.197
2.187
2.173
2.147
2.130
2.112
2.094
2.076
2.058
1.974
1.955
1.937
1.920
1.902
1.883
0.910
0.892
0.873
-0.000
Et
Ph
O
N
OMe
O
4d
2.01
3.29
2.91
1.00
1.03
1.00
1.08
1.08
1.07
3.05
f1 (ppm)

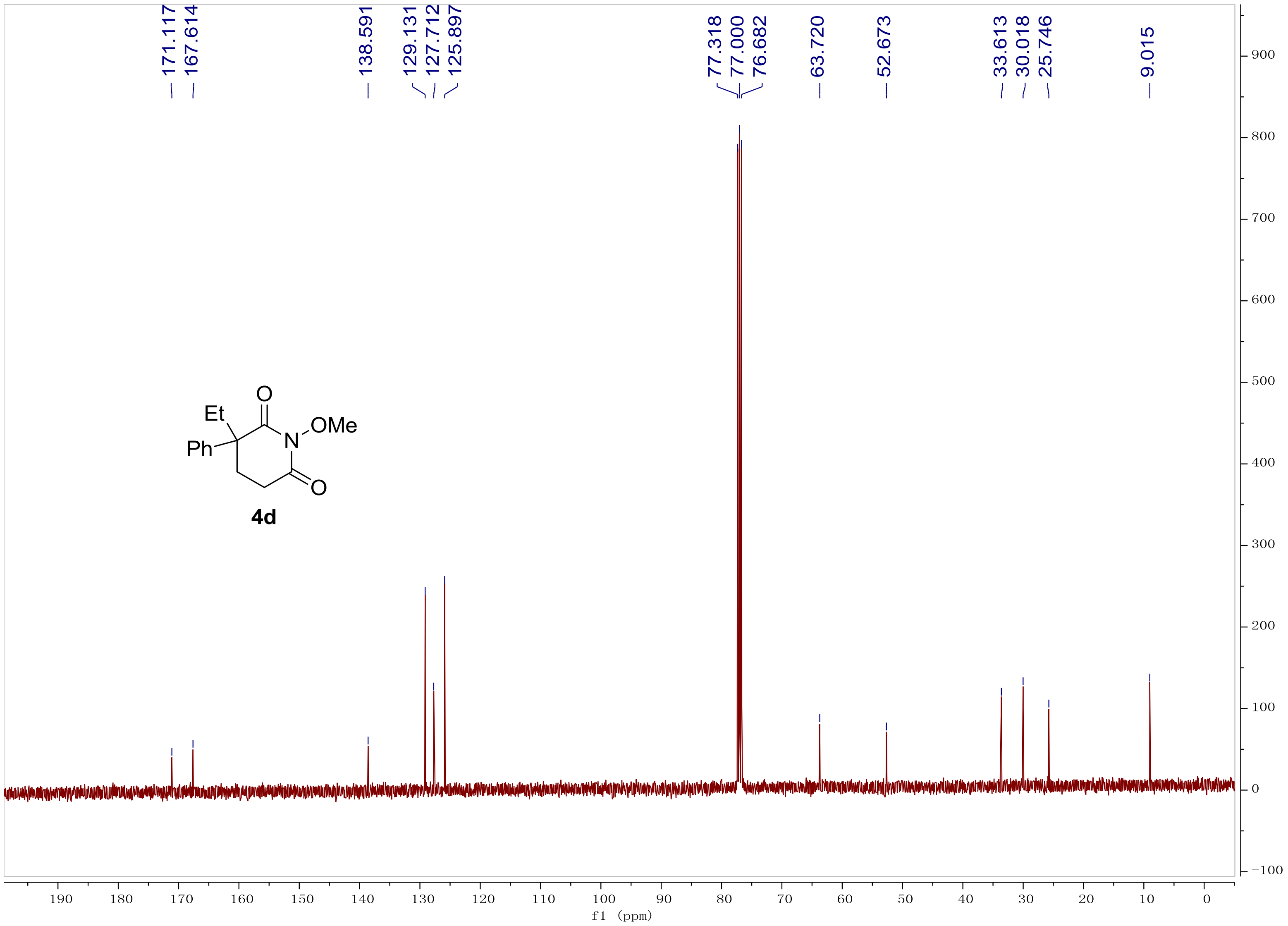

171.117
167.614
138.591
129.131
127.712
125.897
77.318
77.000
76.682
63.720
52.673
33.613
30.018
25.746
9.015
Et
Ph
O
N
OMe
O
4d
f1 (ppm)

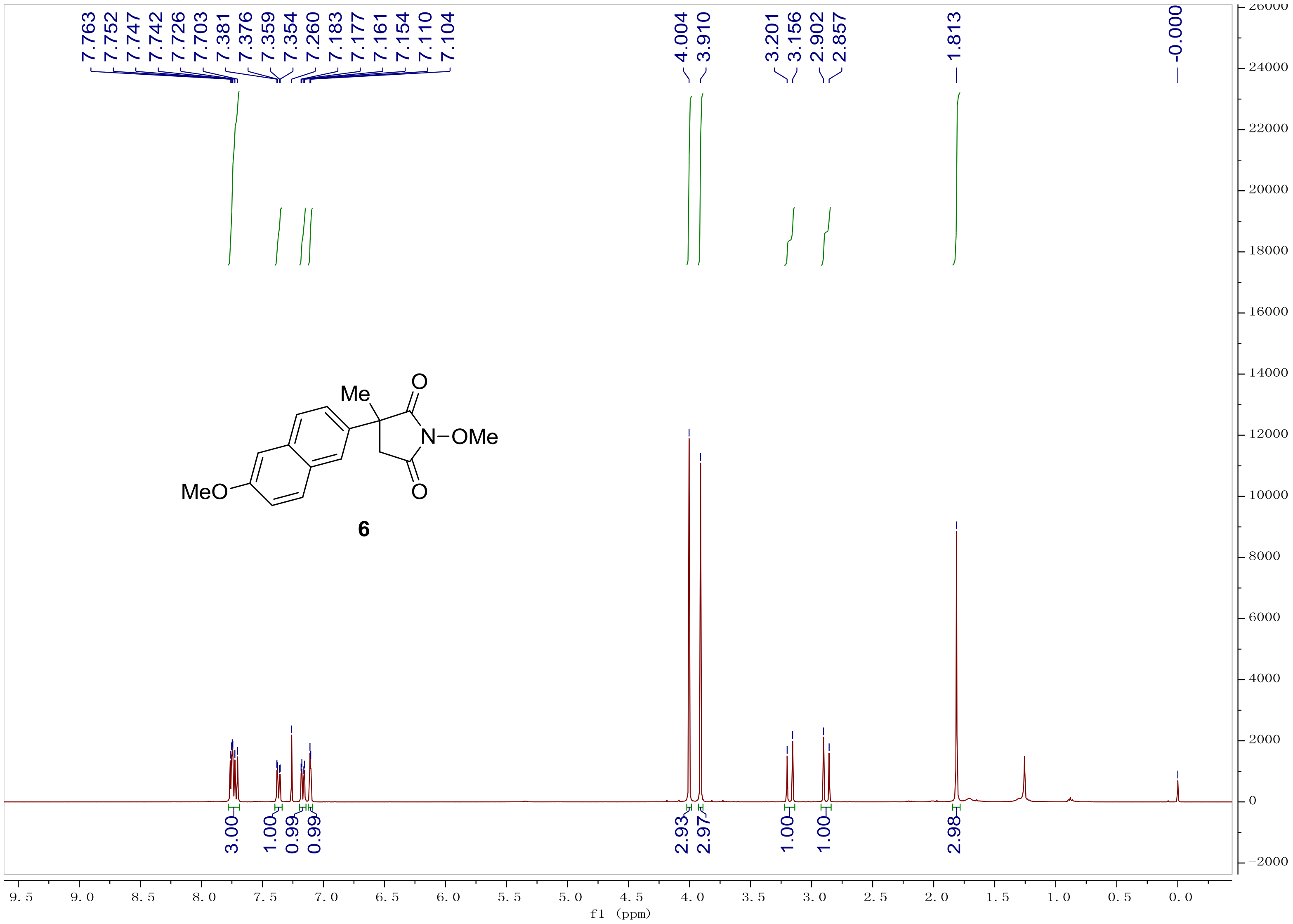

7.763
7.752
7.747
7.742
7.726
7.703
7.381
7.376
7.359
7.354
7.260
7.183
7.177
7.161
7.154
7.110
7.104
4.004
3.910
3.201
3.156
2.902
2.857
1.813
-0.000
Me
O
N-OMe
O
MeO
6
3.00
1.00
0.99
0.99
2.93
2.97
1.00
1.00
2.98
f1 (ppm)

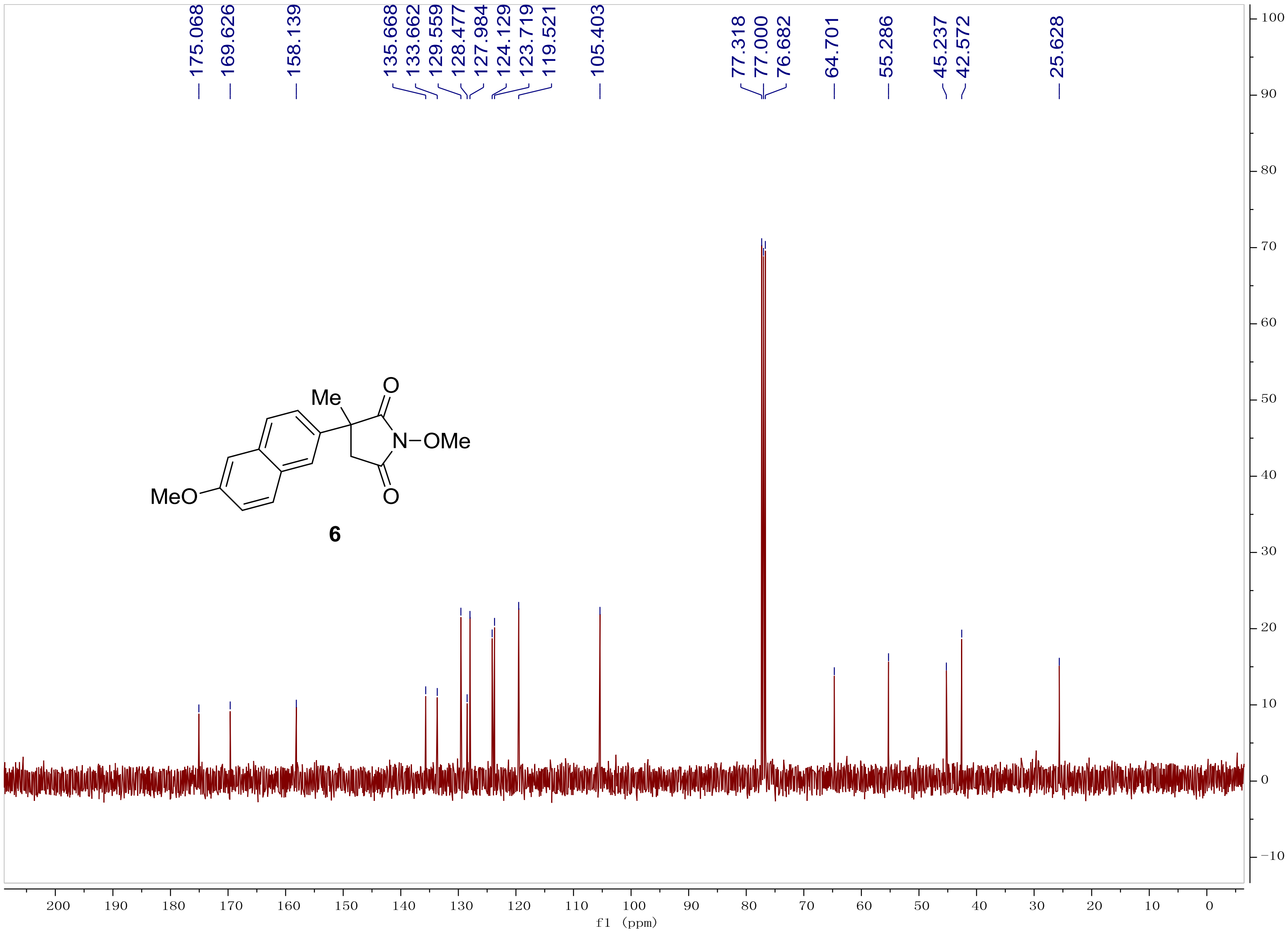
175.068
169.626
158.139
135.668
133.662
129.559
128.477
127.984
124.129
123.719
119.521
105.403
77.318
77.000
76.682
64.701
55.286
45.237
42.572
25.628
Me
O
N–OMe
O
MeO
6
f1 (ppm)

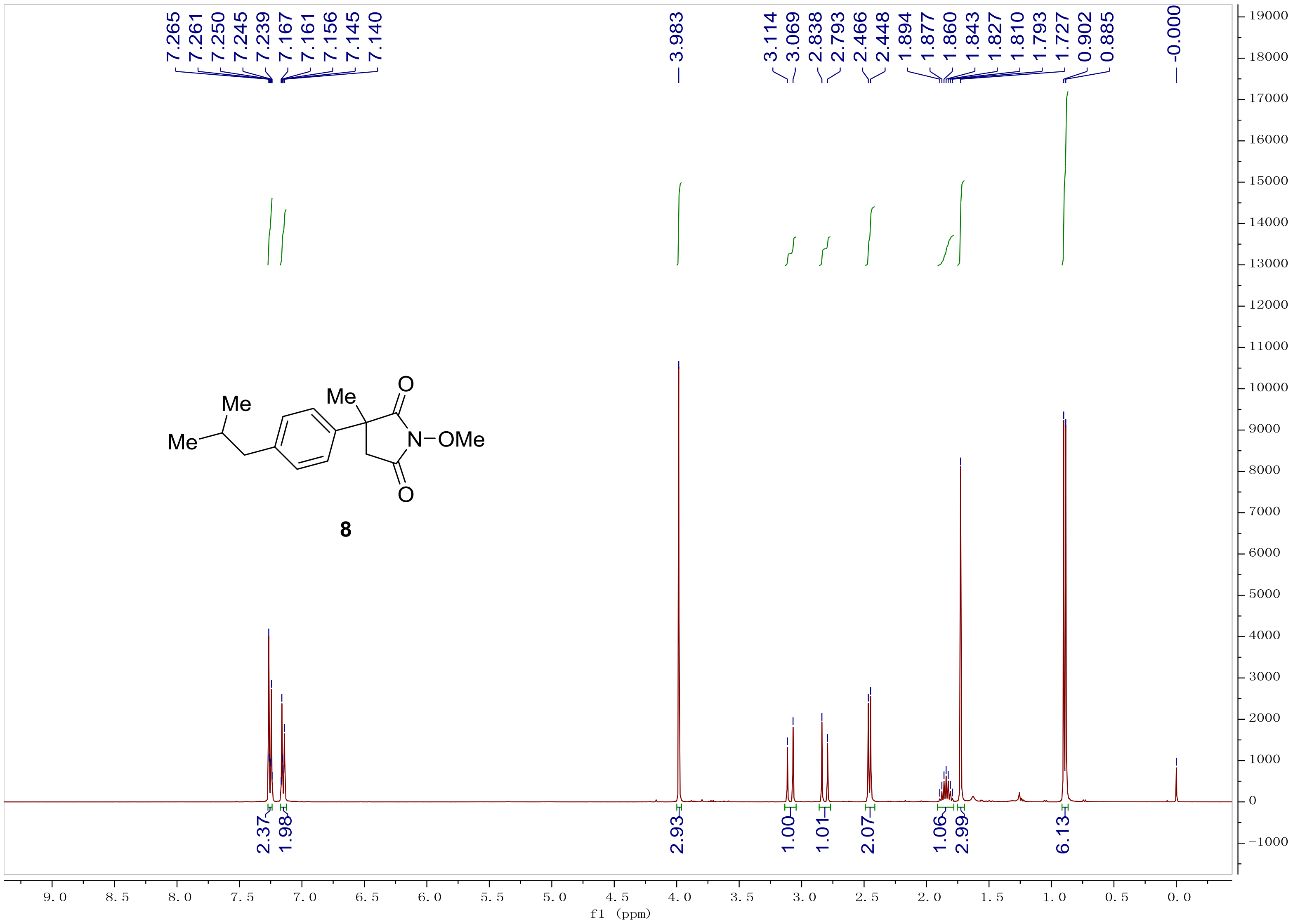
7.265
7.261
7.250
7.245
7.239
7.167
7.161
7.156
7.145
7.140
3.983
3.114
3.069
2.838
2.793
2.466
2.448
1.894
1.877
1.860
1.843
1.827
1.810
1.793
1.727
0.902
0.885
-0.000
Me
Me
Me
O
O
N−OMe
2.37
1.98
2.93
1.00
1.01
2.07
1.06
2.99
6.13
f1 (ppm)

8

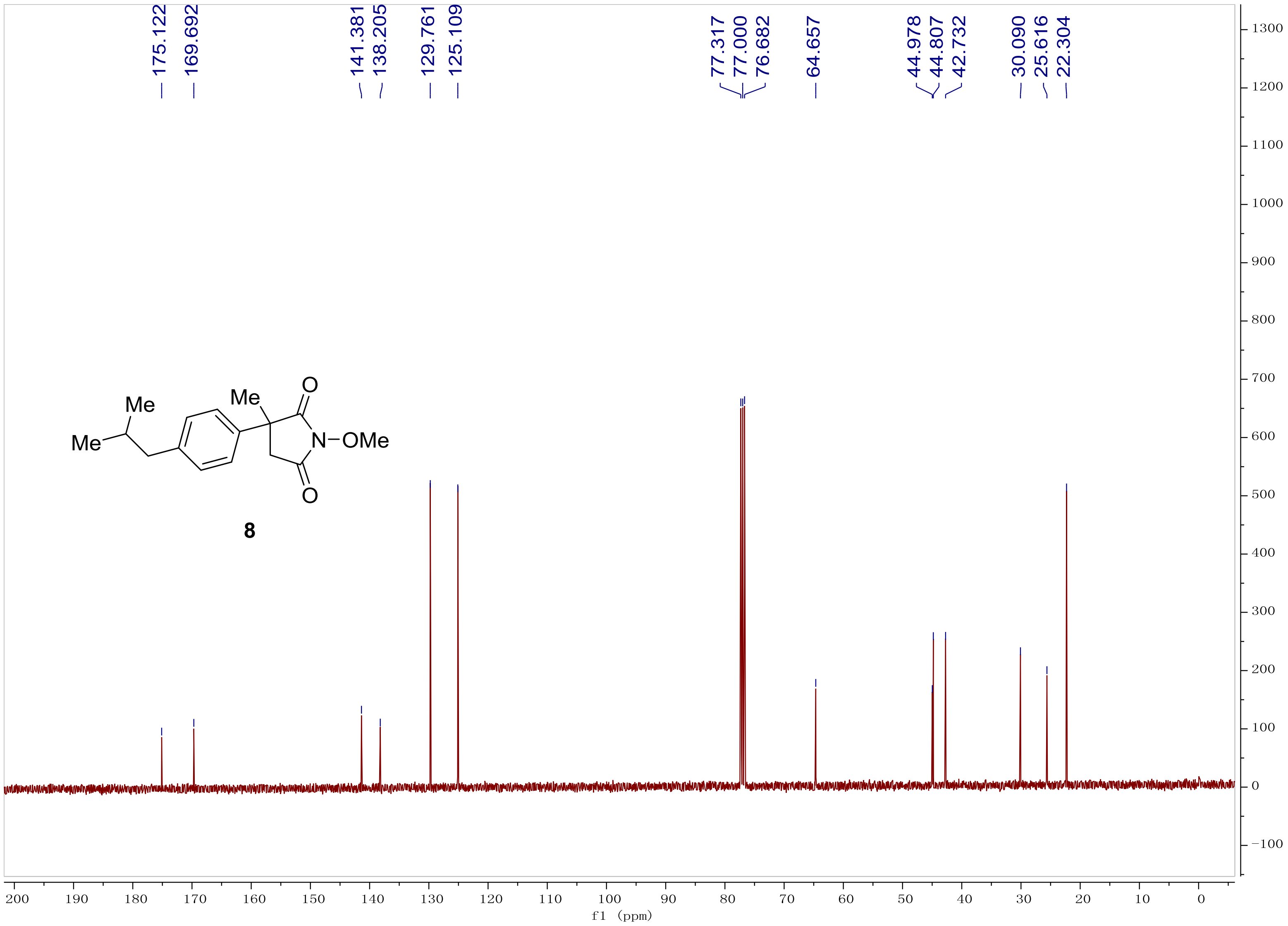
175.122
169.692
141.381
138.205
129.761
125.109
77.317
77.000
76.682
64.657
44.978
44.807
42.732
30.090
25.616
22.304
Me
Me
Me
O
N−OMe
O
8
f1 (ppm)

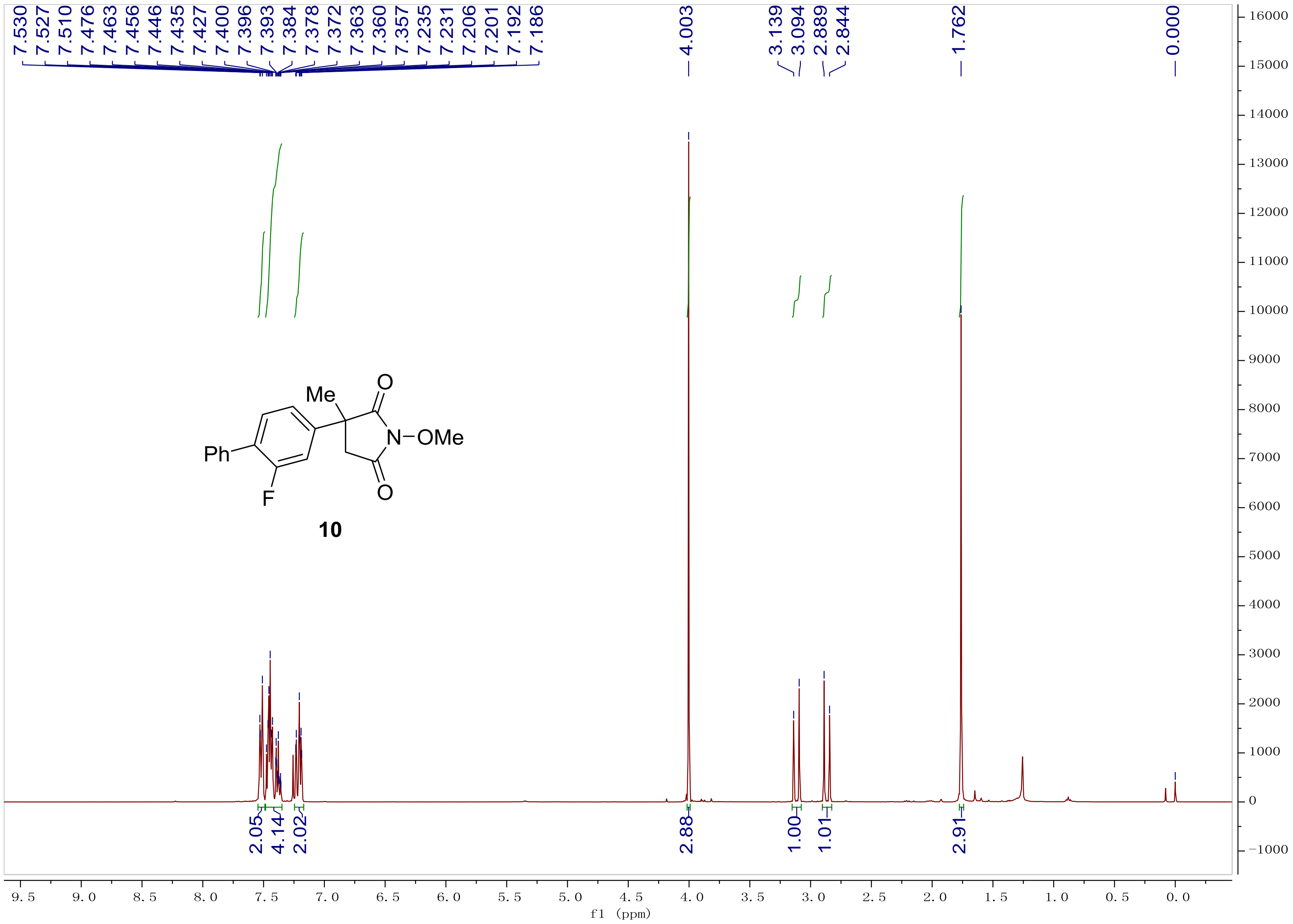
7.530
7.527
7.510
7.476
7.463
7.456
7.446
7.435
7.427
7.400
7.396
7.393
7.384
7.378
7.372
7.363
7.360
7.357
7.235
7.231
7.206
7.201
7.192
7.186
4.003
3.139
3.094
2.889
2.844
1.762
0.000
Me
O
N–OMe
Ph
F
O
10
2.05
4.14
2.02
2.88
1.00
1.01
2.91
f1 (ppm)

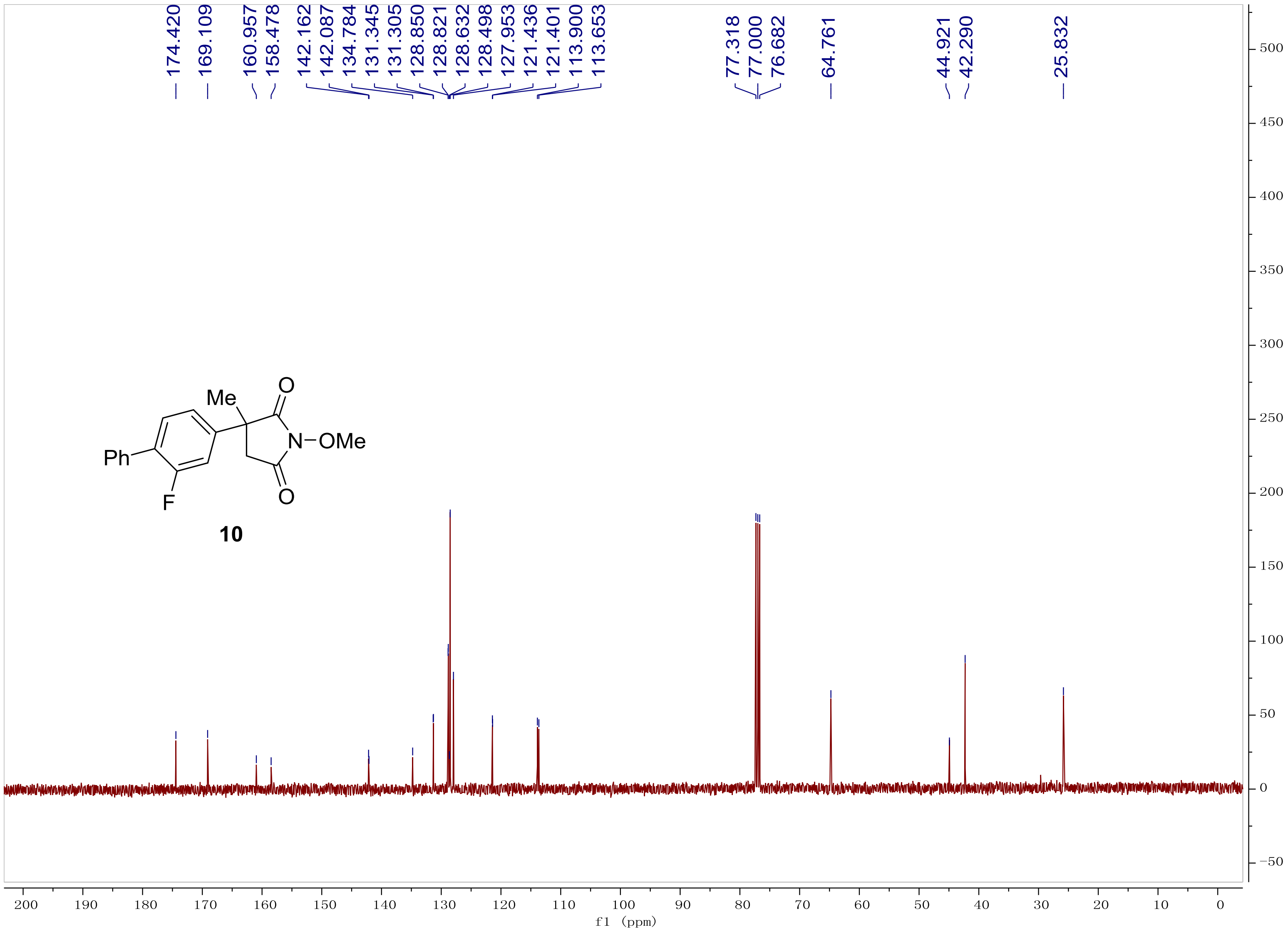

174.420
169.109
160.957
158.478
142.162
142.087
134.784
131.345
131.305
128.850
128.821
128.632
128.498
127.953
121.436
121.401
113.900
113.653
77.318
77.000
76.682
64.761
44.921
42.290
25.832
Me
O
N−OMe
Ph
F
O
10
f1 (ppm)

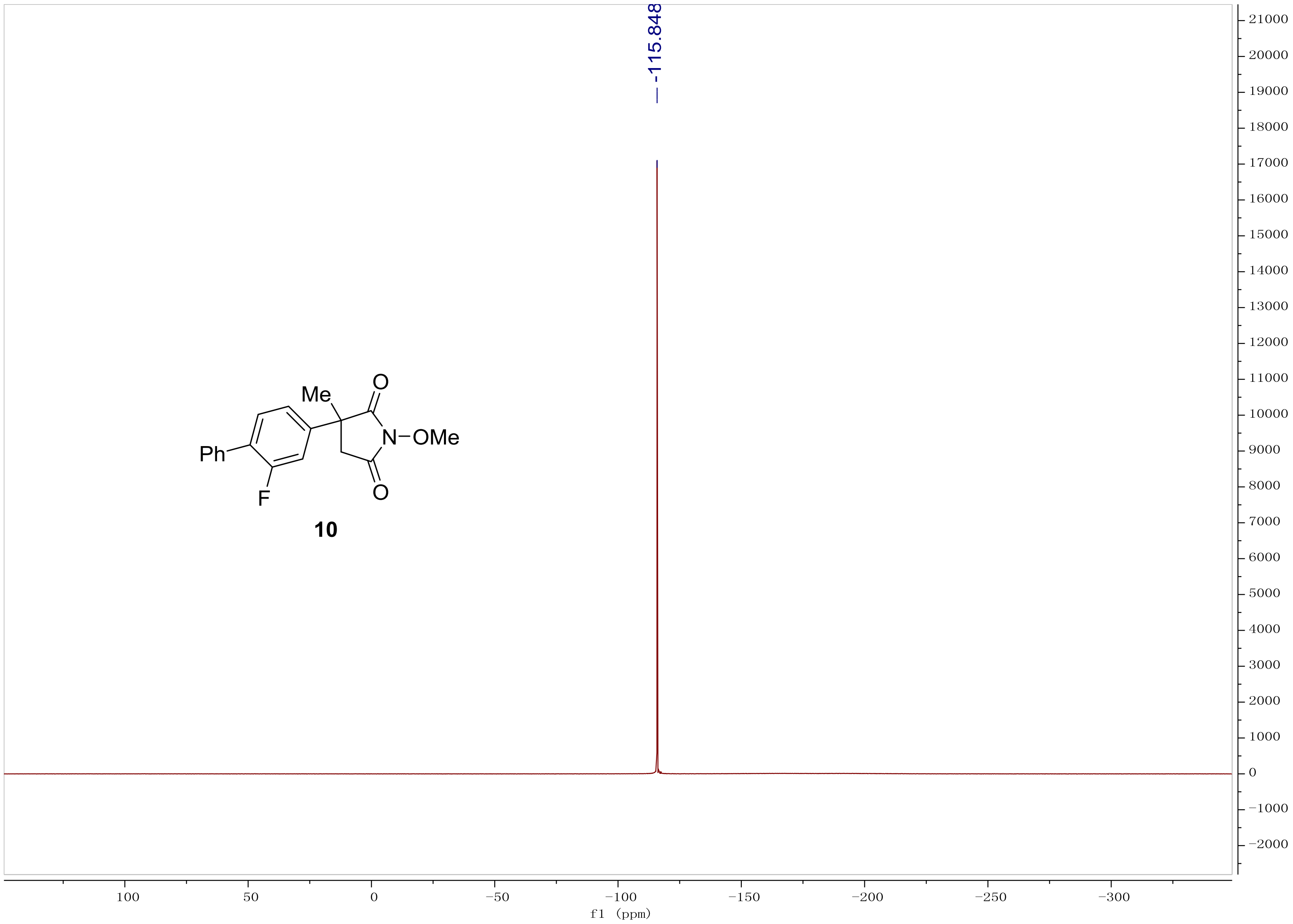

-115.848
Me
O
N
OMe
Ph
F
O
21000
20000
19000
18000
17000
16000
15000
14000
13000
12000
11000
10000
9000
8000
7000
6000
5000
4000
3000
2000
1000
0
−1000
−2000
100
50
0
−50
−100
−150
−200
−250
−300
f1 (ppm)

10

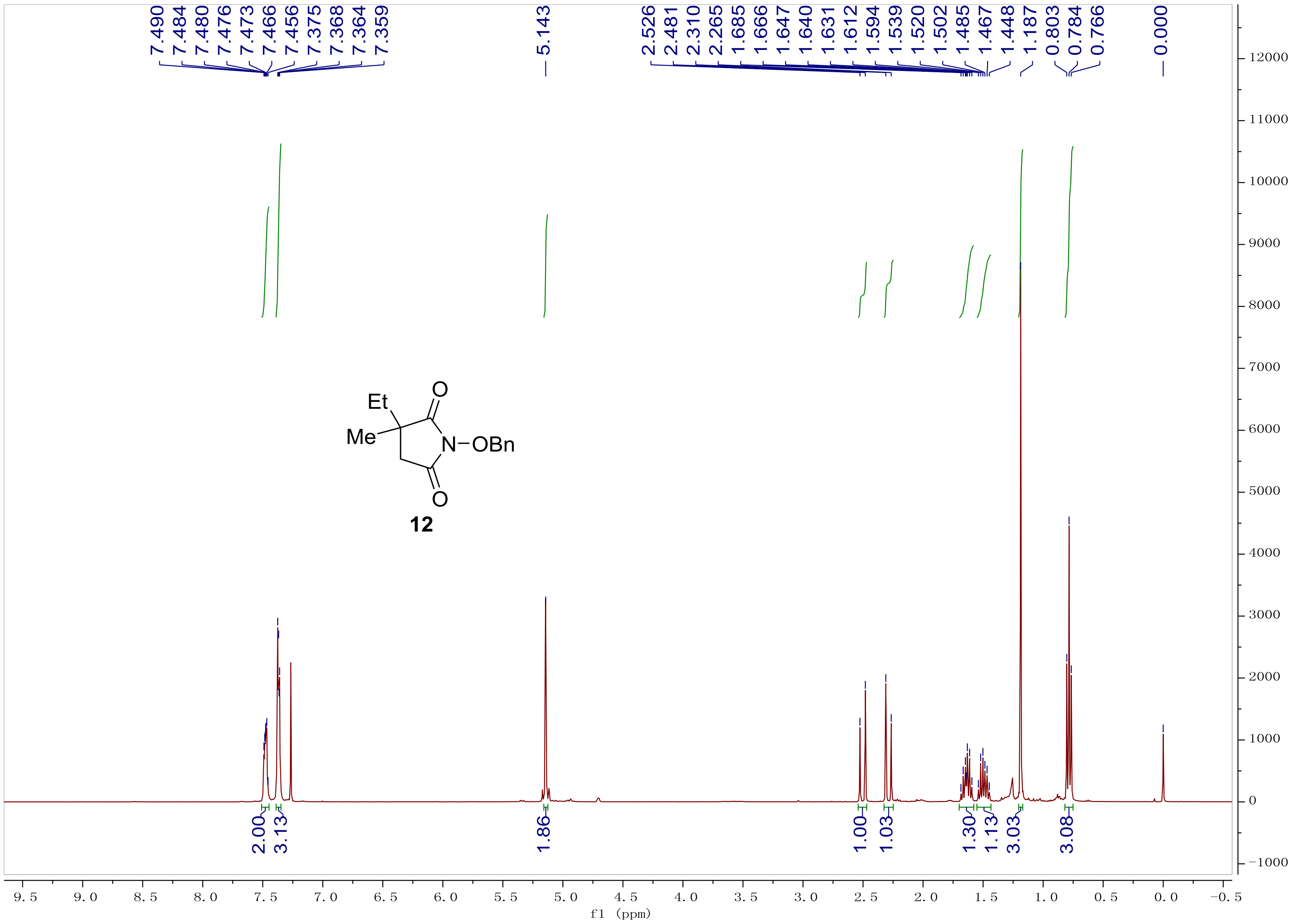

7.490
7.484
7.480
7.476
7.473
7.466
7.456
7.375
7.368
7.364
7.359
5.143
2.526
2.481
2.310
2.265
1.685
1.666
1.647
1.640
1.631
1.612
1.594
1.539
1.520
1.502
1.485
1.467
1.448
1.187
0.803
0.784
0.766
0.000
Et
Me
N−OBn
O
O
2.00
3.13
1.86
1.00
1.03
1.30
1.13
3.03
3.08
12000
11000
10000
9000
8000
7000
6000
5000
4000
3000
2000
1000
0
−1000
9.5 9.0 8.5 8.0 7.5 7.0 6.5 6.0 5.5 5.0 4.5 4.0 3.5 3.0 2.5 2.0 1.5 1.0 0.5 0.0 −0.5
f1 (ppm)

12

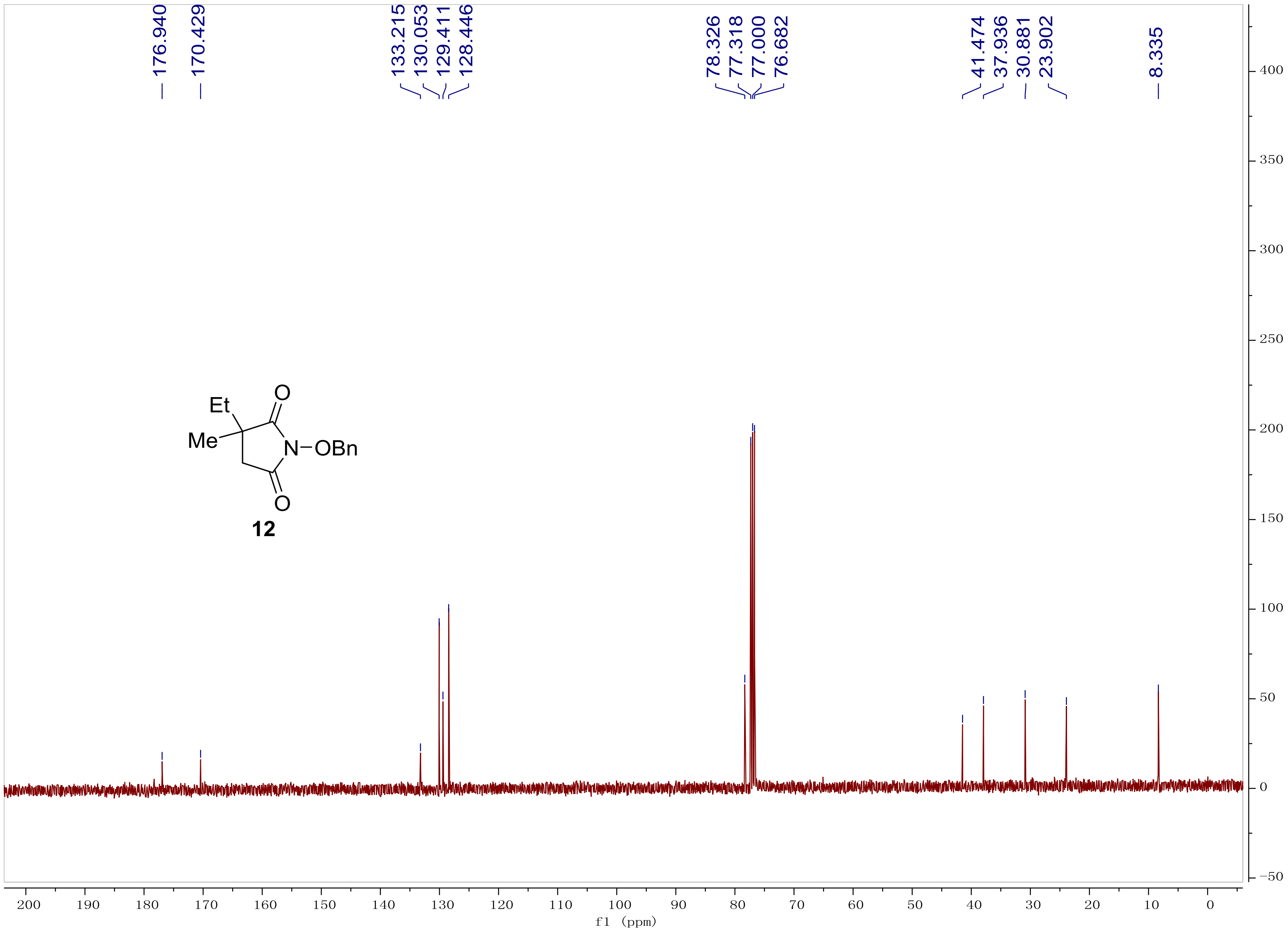

176.940
170.429
133.215
130.053
129.411
128.446
78.326
77.318
77.000
76.682
41.474
37.936
30.881
23.902
8.335
Et
Me
N−OBn
O
O
12
400
350
300
250
200
150
100
50
0
−50
200 190 180 170 160 150 140 130 120 110 100 90 80 70 60 50 40 30 20 10 0
f1 (ppm)

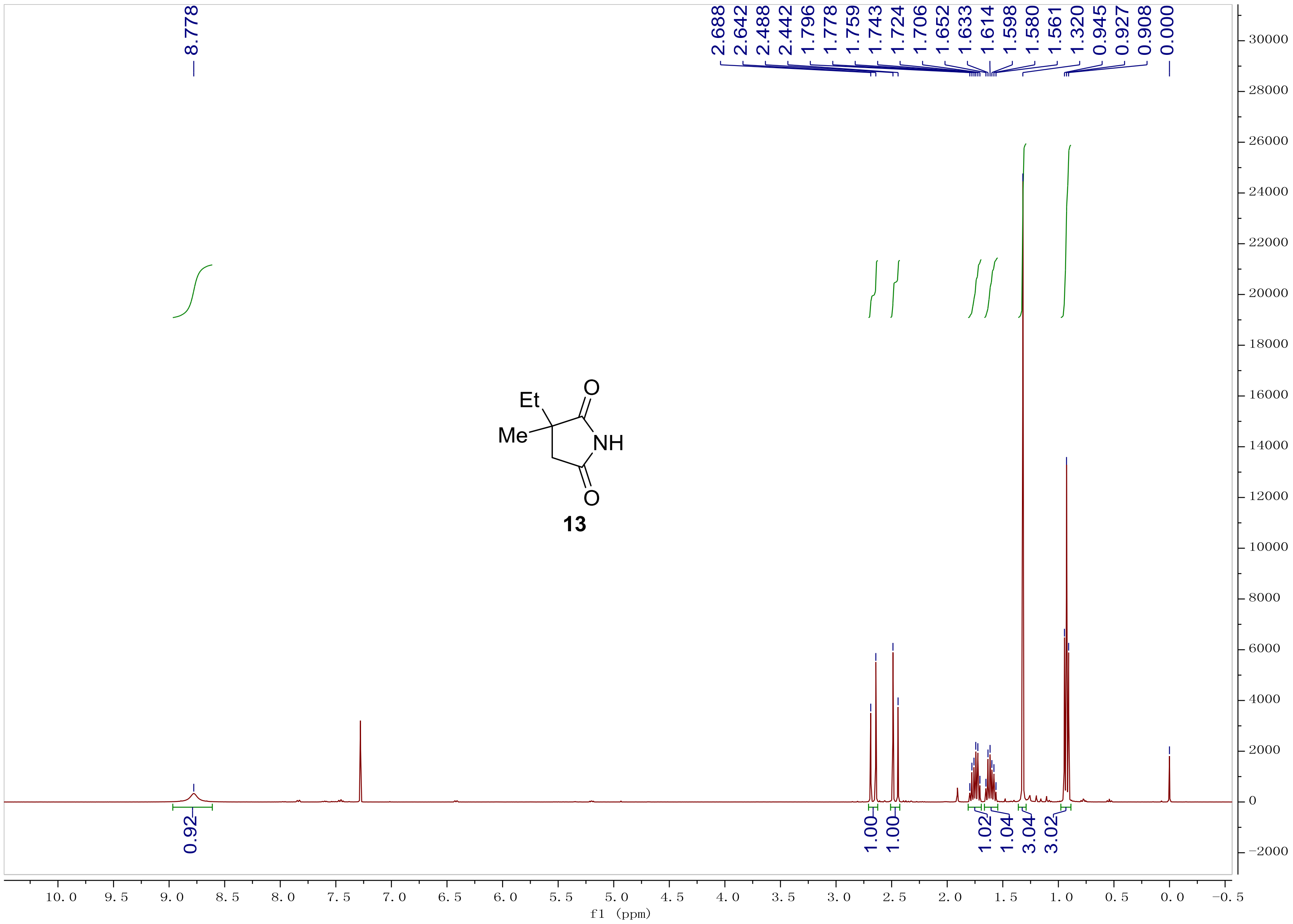
8.778
2.688
2.642
2.488
2.442
1.796
1.778
1.759
1.743
1.724
1.706
1.652
1.633
1.614
1.598
1.580
1.561
1.320
0.945
0.927
0.908
0.000
Et
Me
O
NH
O
13
0.92
1.00
1.00
1.02
1.04
3.04
3.02
f1 (ppm)

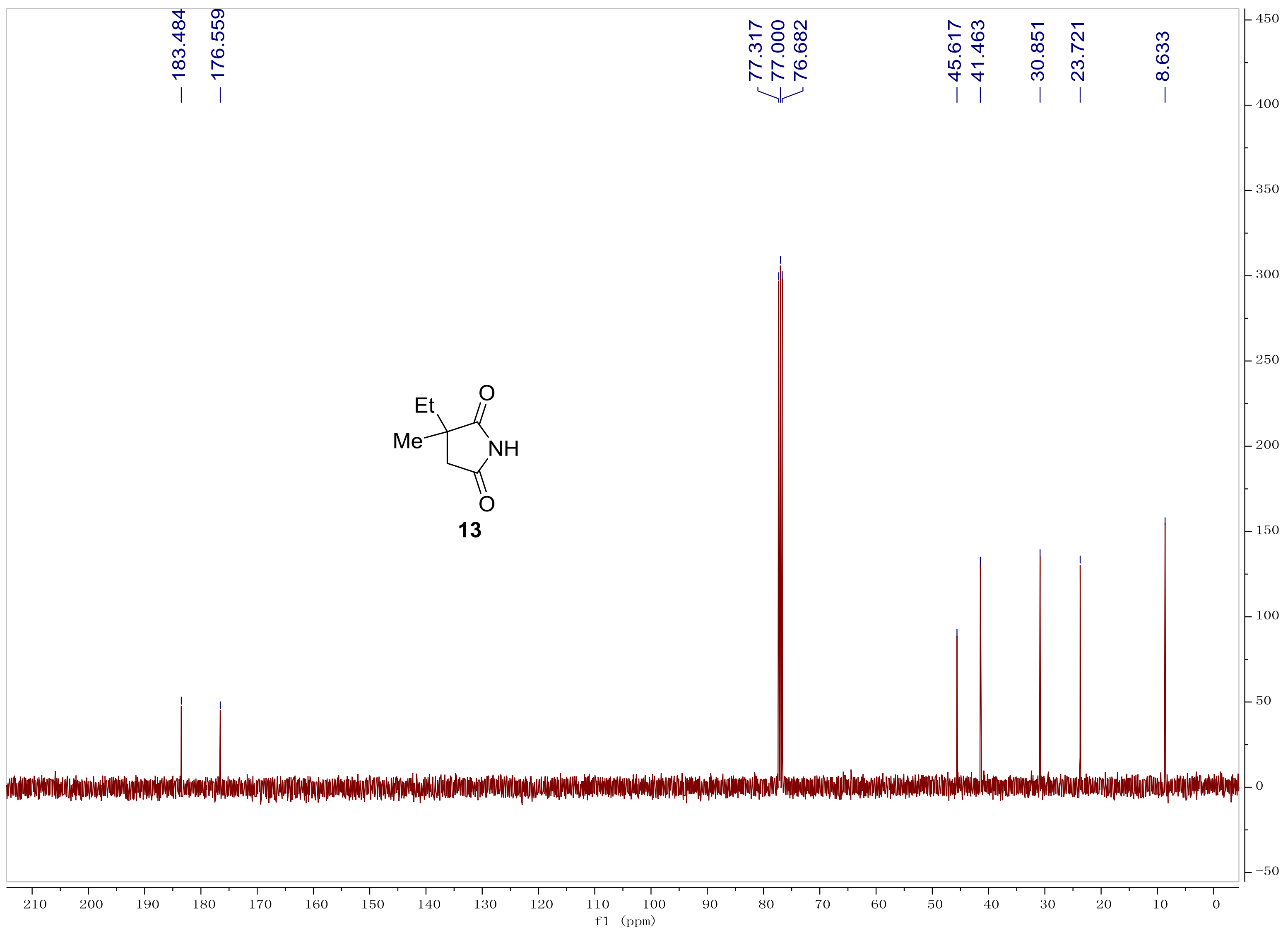
183.484
176.559
77.317
77.000
76.682
45.617
41.463
30.851
23.721
8.633
Et
Me
O
NH
O
13
f1 (ppm)

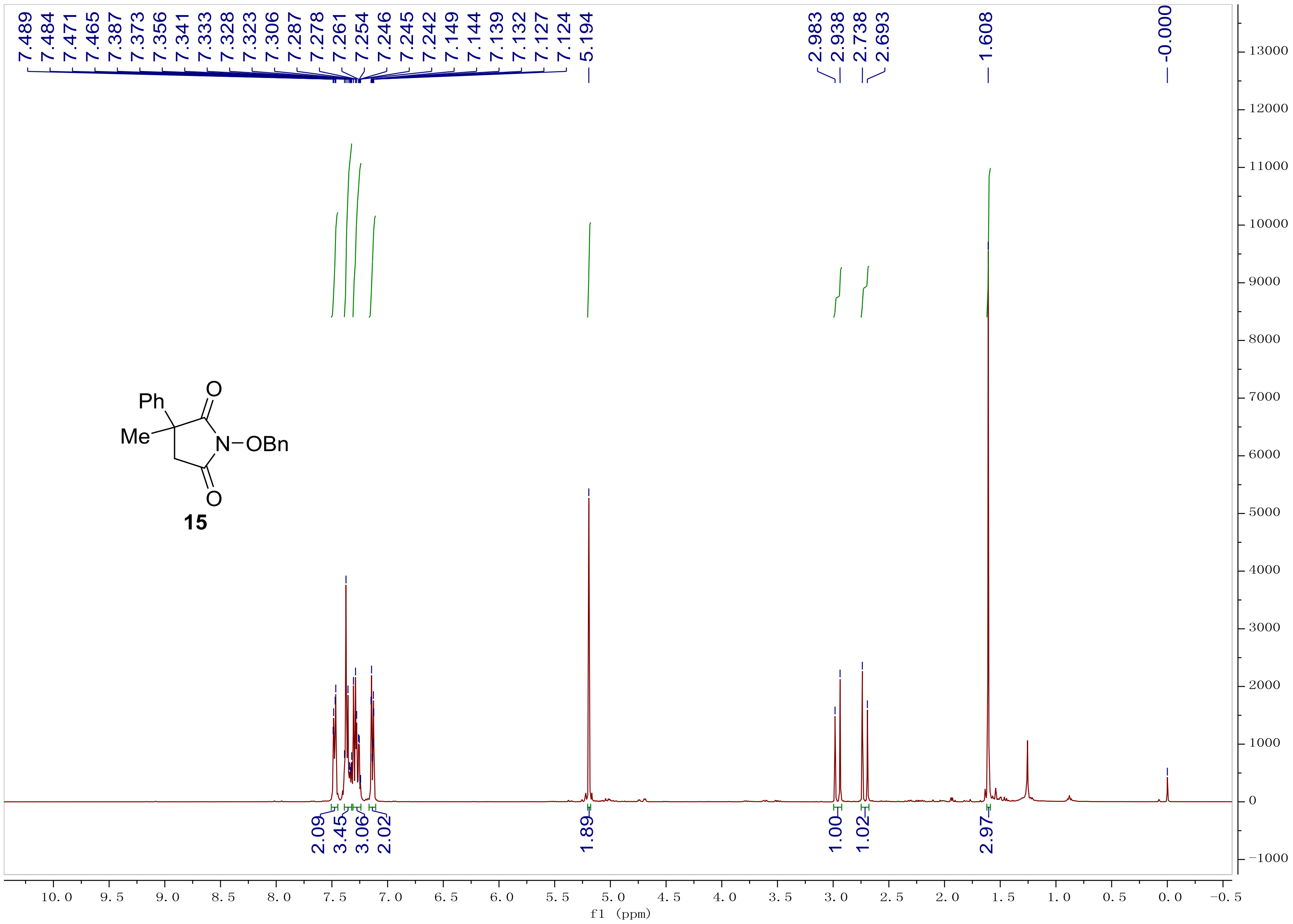
7.489
7.484
7.471
7.465
7.387
7.373
7.356
7.341
7.333
7.328
7.323
7.306
7.287
7.278
7.261
7.254
7.246
7.245
7.242
7.149
7.144
7.139
7.132
7.127
7.124
5.194
2.983
2.938
2.738
2.693
1.608
-0.000
Ph
O
Me
N–OBn
O
15
2.09
3.45
3.06
2.02
1.89
1.00
1.02
2.97
f1 (ppm)

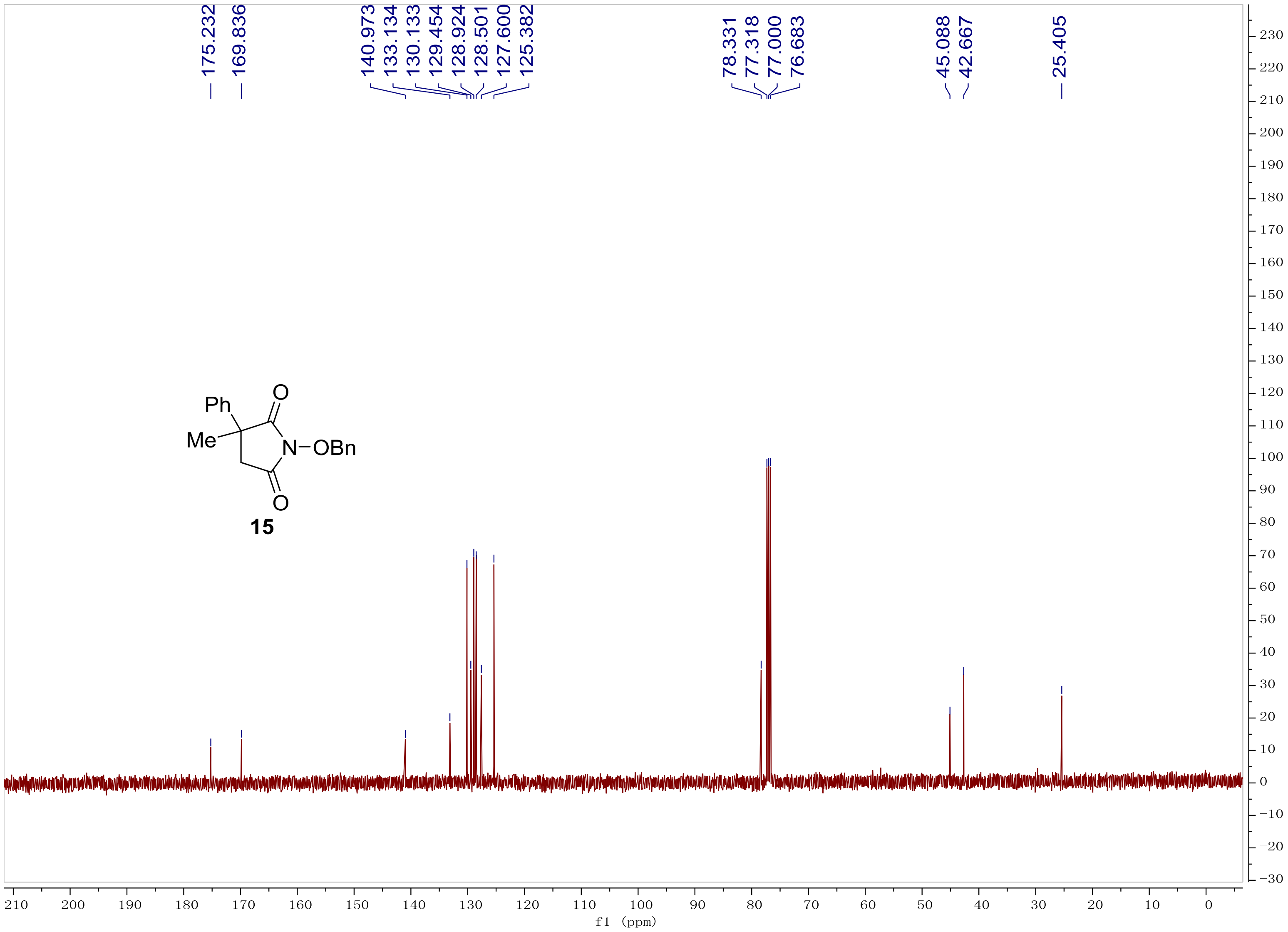

175.232
169.836
140.973
133.134
130.133
129.454
128.924
128.501
127.600
125.382
78.331
77.318
77.000
76.683
45.088
42.667
25.405
Ph
Me
N–OBn
O
O
15
f1 (ppm)

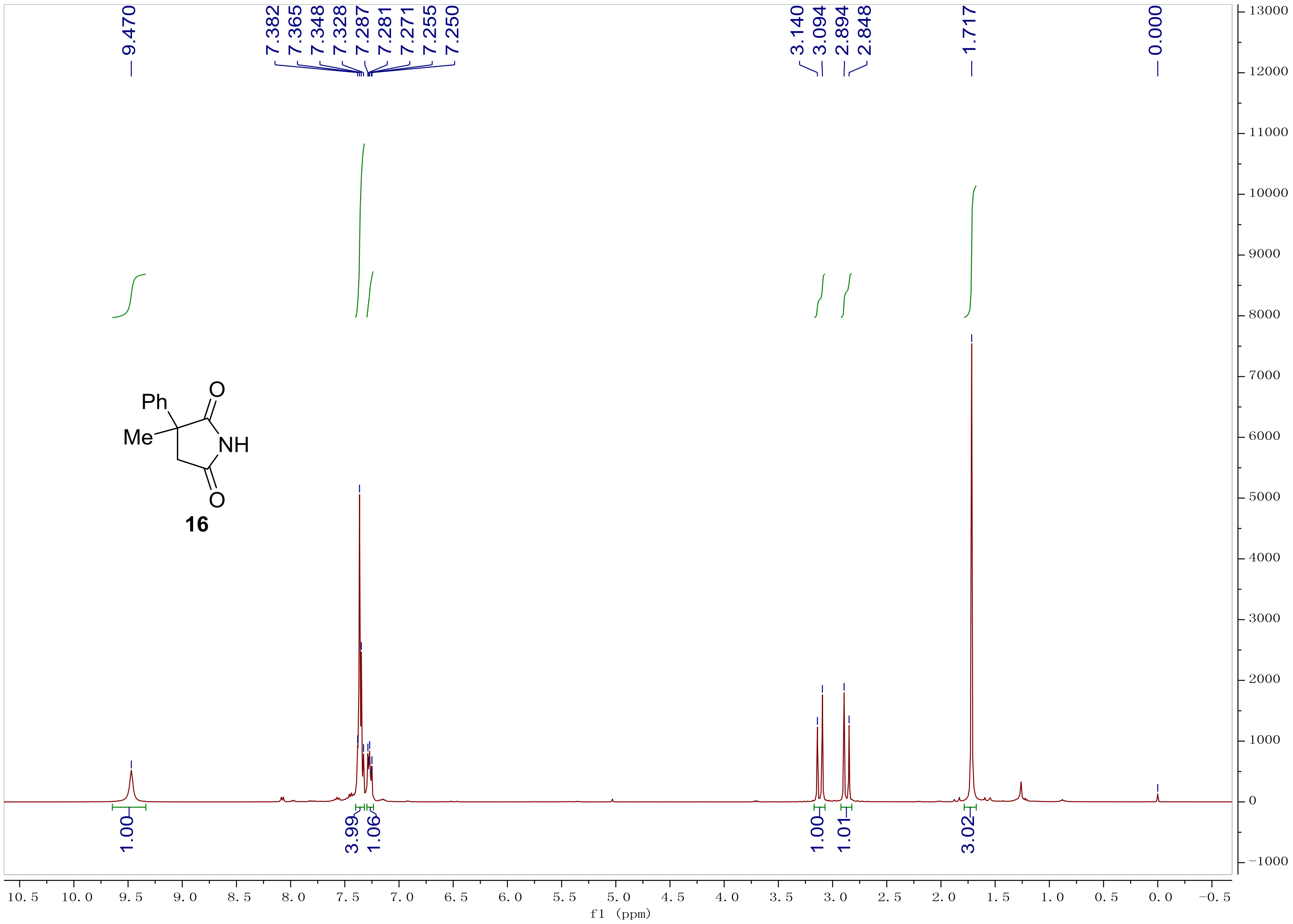
9.470
7.382
7.365
7.348
7.328
7.287
7.281
7.271
7.255
7.250
3.140
3.094
2.894
2.848
1.717
0.000
Ph
Me
NH
O
O
16
1.00
3.99
1.06
1.00
1.01
3.02
f1 (ppm)

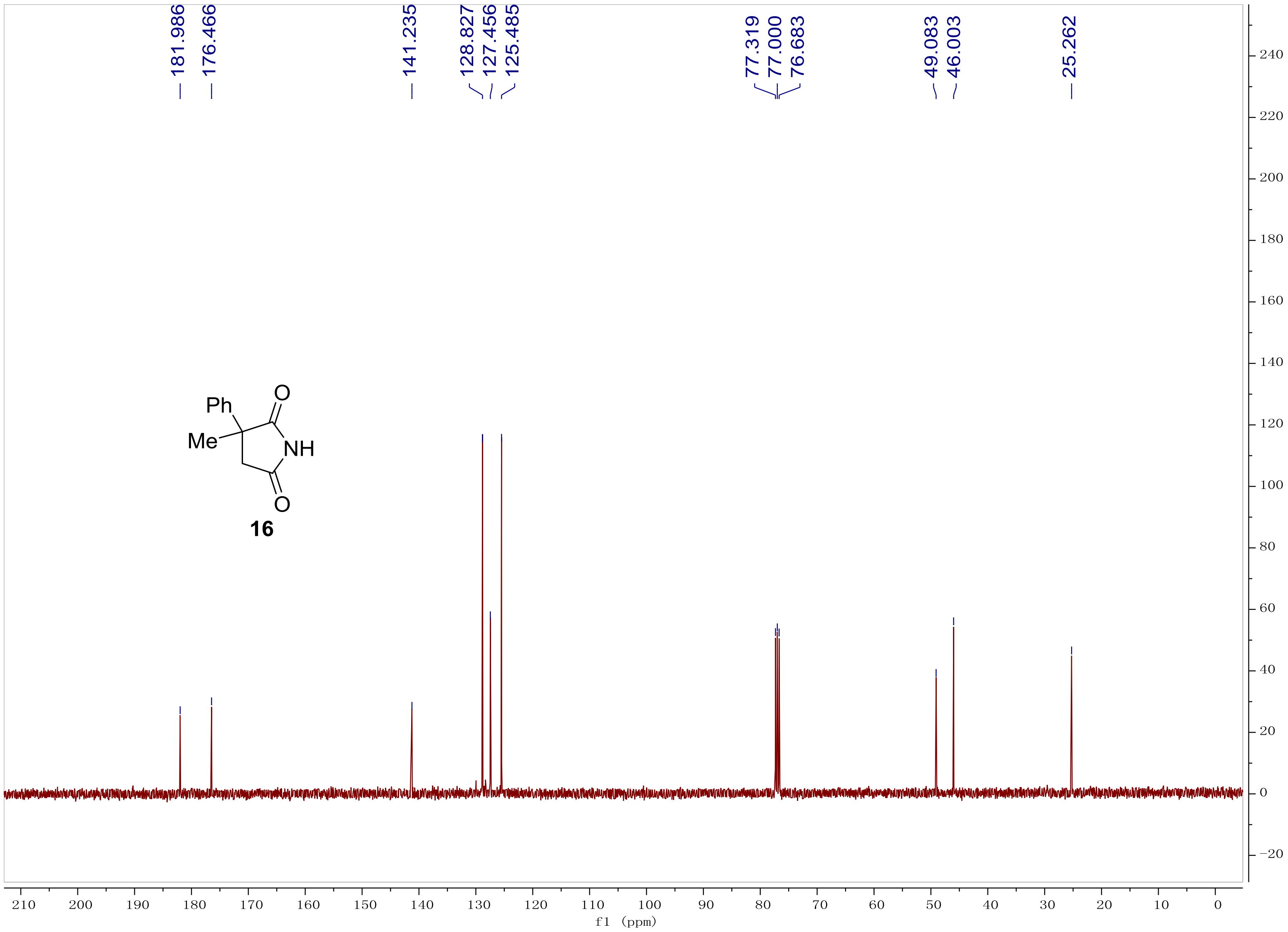
181.986
176.466
141.235
128.827
127.456
125.485
77.319
77.000
76.683
49.083
46.003
25.262
Ph
Me
O
NH
O
16
f1 (ppm)

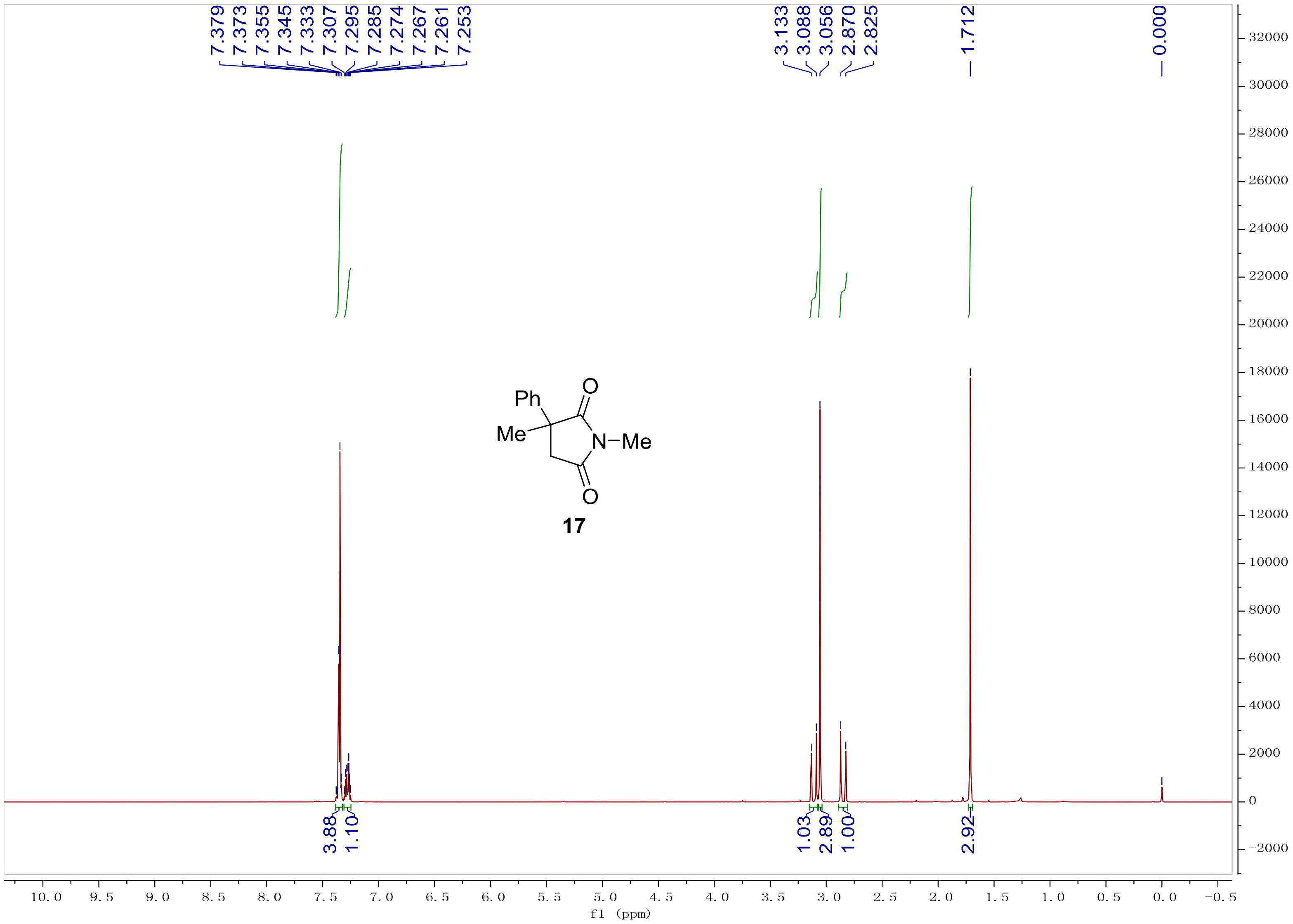

7.379
7.373
7.355
7.345
7.333
7.307
7.295
7.285
7.274
7.267
7.261
7.253
3.133
3.088
3.056
2.870
2.825
1.712
0.000
Ph
Me
O
N−Me
O
3.88
1.10
1.03
2.89
1.00
2.92
f1 (ppm)

17

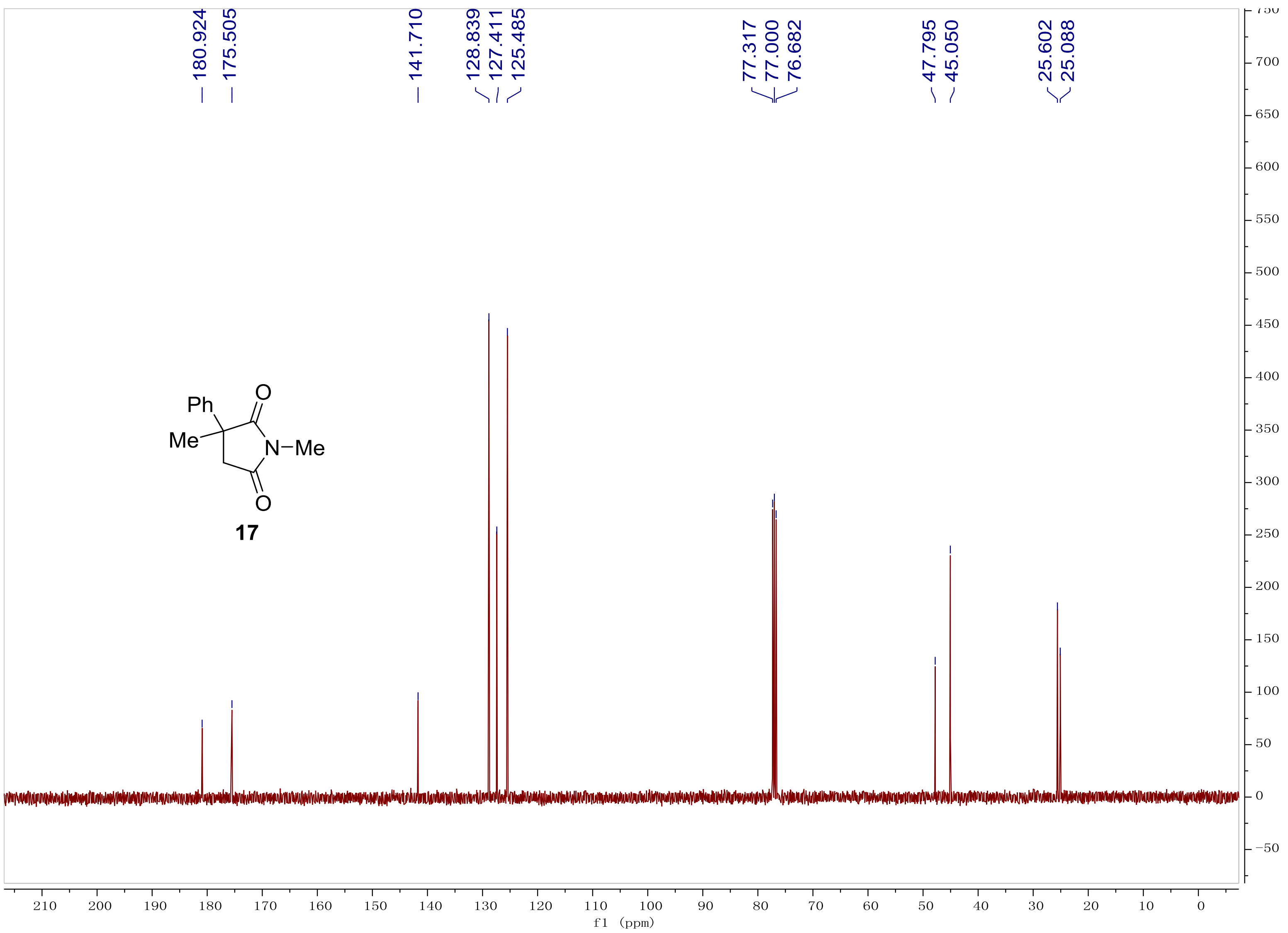

180.924
175.505
141.710
128.839
127.411
125.485
77.317
77.000
76.682
47.795
45.050
25.602
25.088
Ph
Me
N−Me
O
O
17
f1 (ppm)

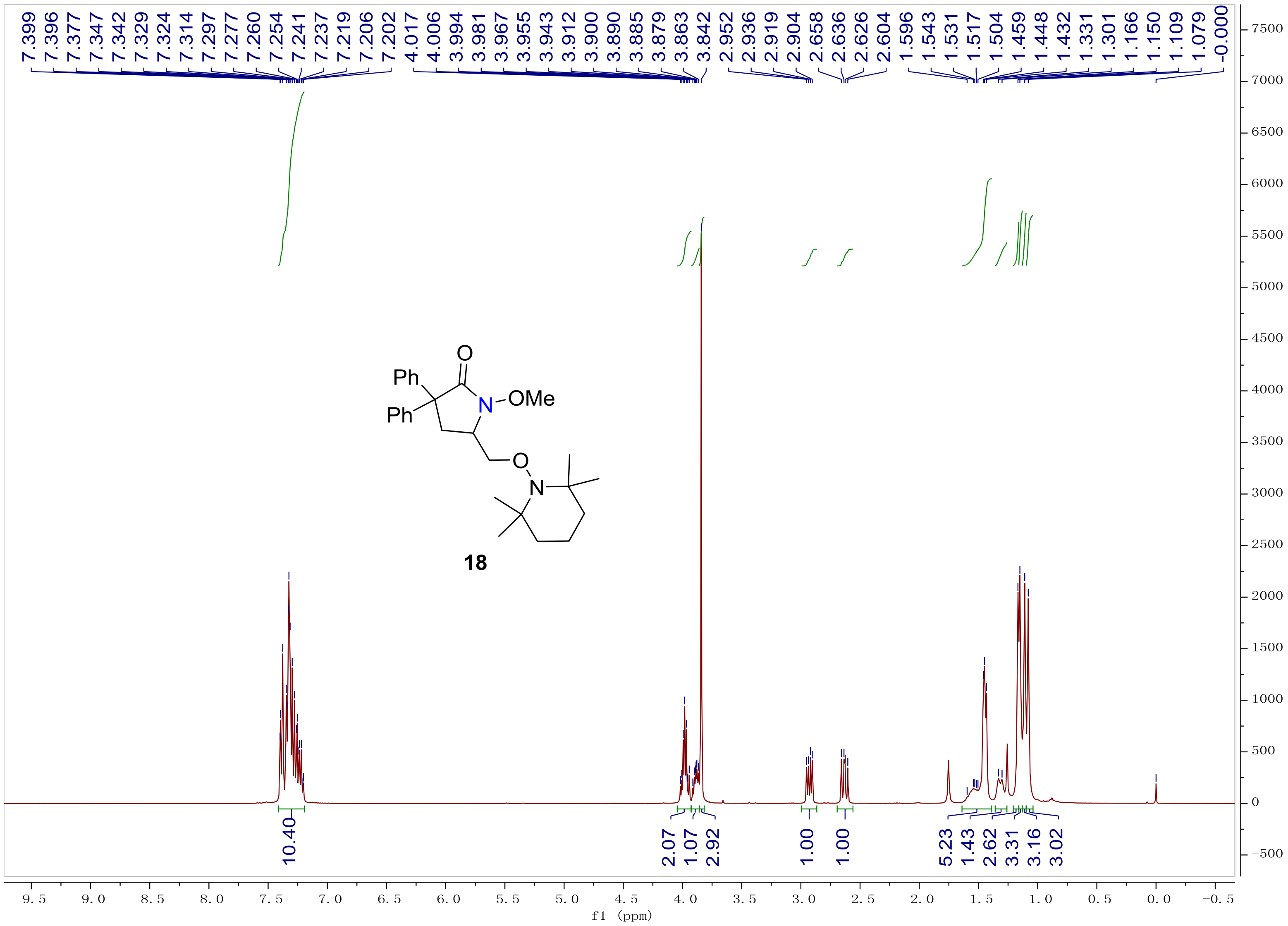
7.399
7.396
7.377
7.347
7.342
7.329
7.324
7.314
7.297
7.277
7.260
7.254
7.241
7.237
7.219
7.206
7.202
4.017
4.006
3.994
3.981
3.967
3.955
3.943
3.912
3.900
3.890
3.885
3.879
3.863
3.842
2.952
2.936
2.919
2.904
2.658
2.636
2.626
2.604
1.596
1.543
1.531
1.517
1.504
1.459
1.448
1.432
1.331
1.301
1.166
1.150
1.109
1.079
-0.000
O
Ph
Ph
N
OMe
O
N
18
10.40
2.07
1.07
2.92
1.00
1.00
5.23
1.43
2.62
3.31
3.16
3.02
f1 (ppm)

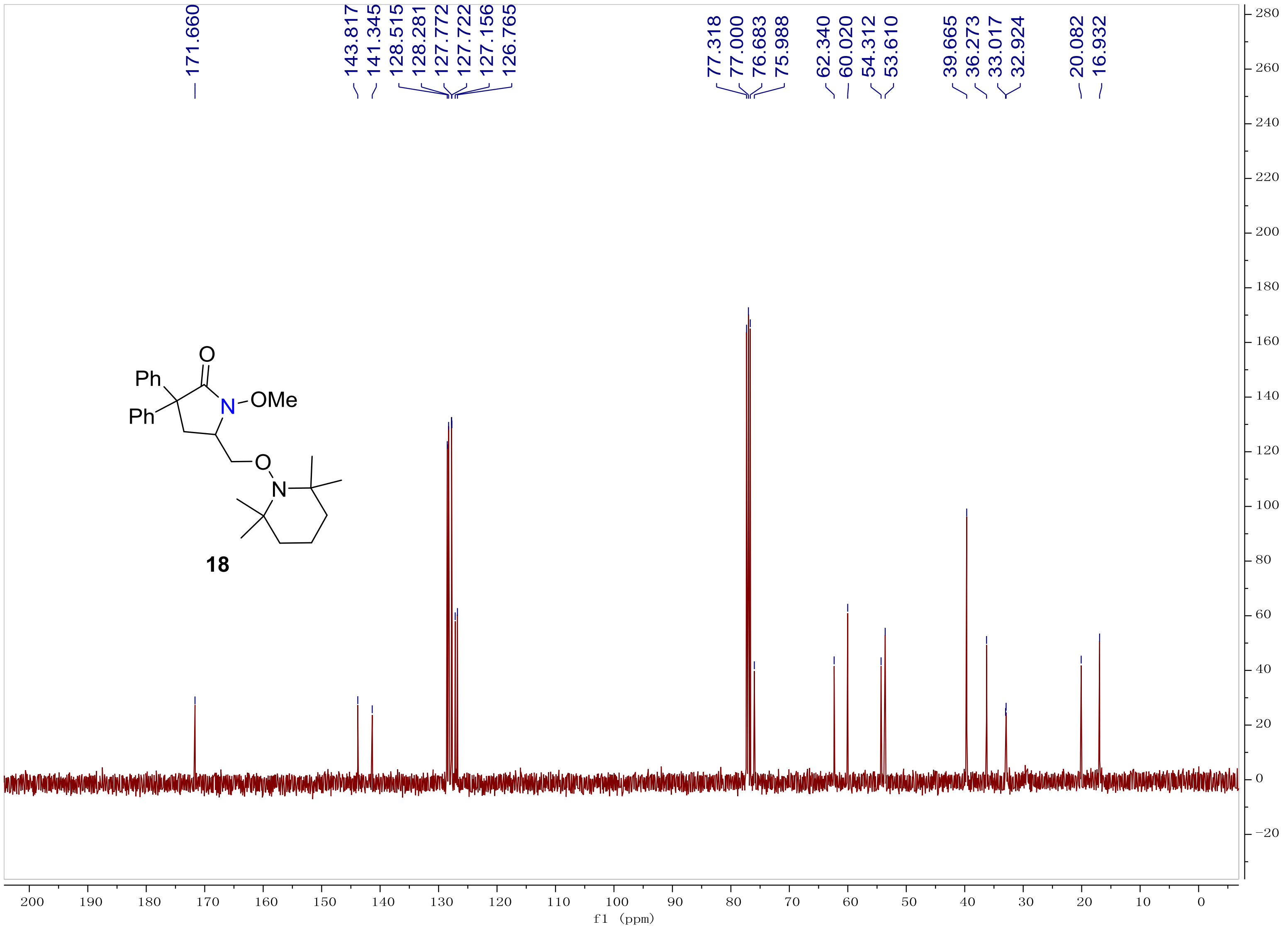
171.660
143.817
141.345
128.515
128.281
127.772
127.722
127.156
126.765
77.318
77.000
76.683
75.988
62.340
60.020
54.312
53.610
39.665
36.273
33.017
32.924
20.082
16.932
Ph
Ph
O
N
OMe
O
N
18
f1 (ppm)

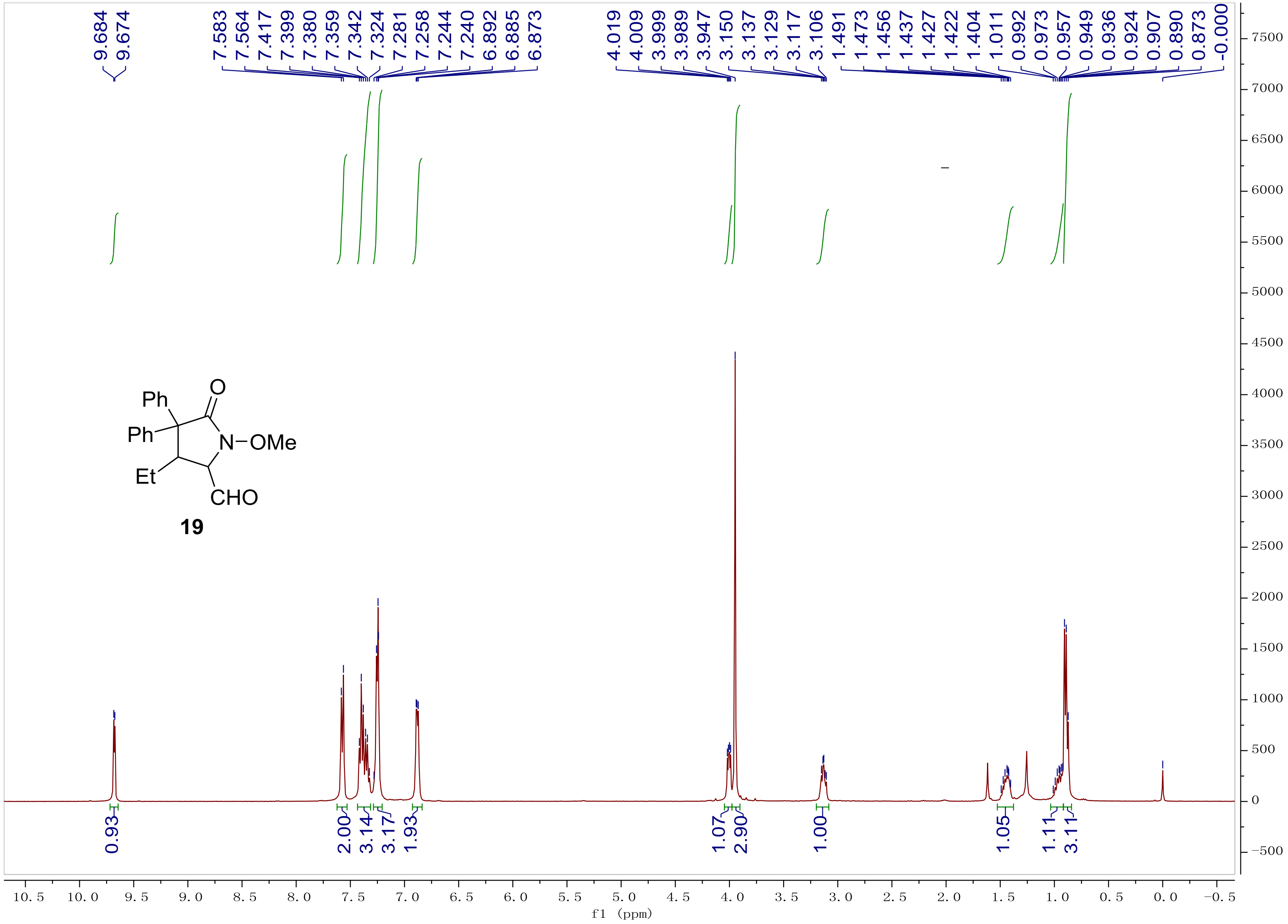
9.684
9.674
7.583
7.564
7.417
7.399
7.380
7.359
7.342
7.324
7.281
7.258
7.244
7.240
6.892
6.885
6.873
4.019
4.009
3.999
3.989
3.947
3.150
3.137
3.129
3.117
3.106
1.491
1.473
1.456
1.437
1.427
1.422
1.404
1.011
0.992
0.973
0.957
0.949
0.936
0.924
0.907
0.890
0.873
-0.000
Ph
Ph
O
N−OMe
Et
CHO
19
0.93
2.00
3.14
3.17
1.93
1.07
2.90
1.00
1.05
1.11
3.11
f1 (ppm)

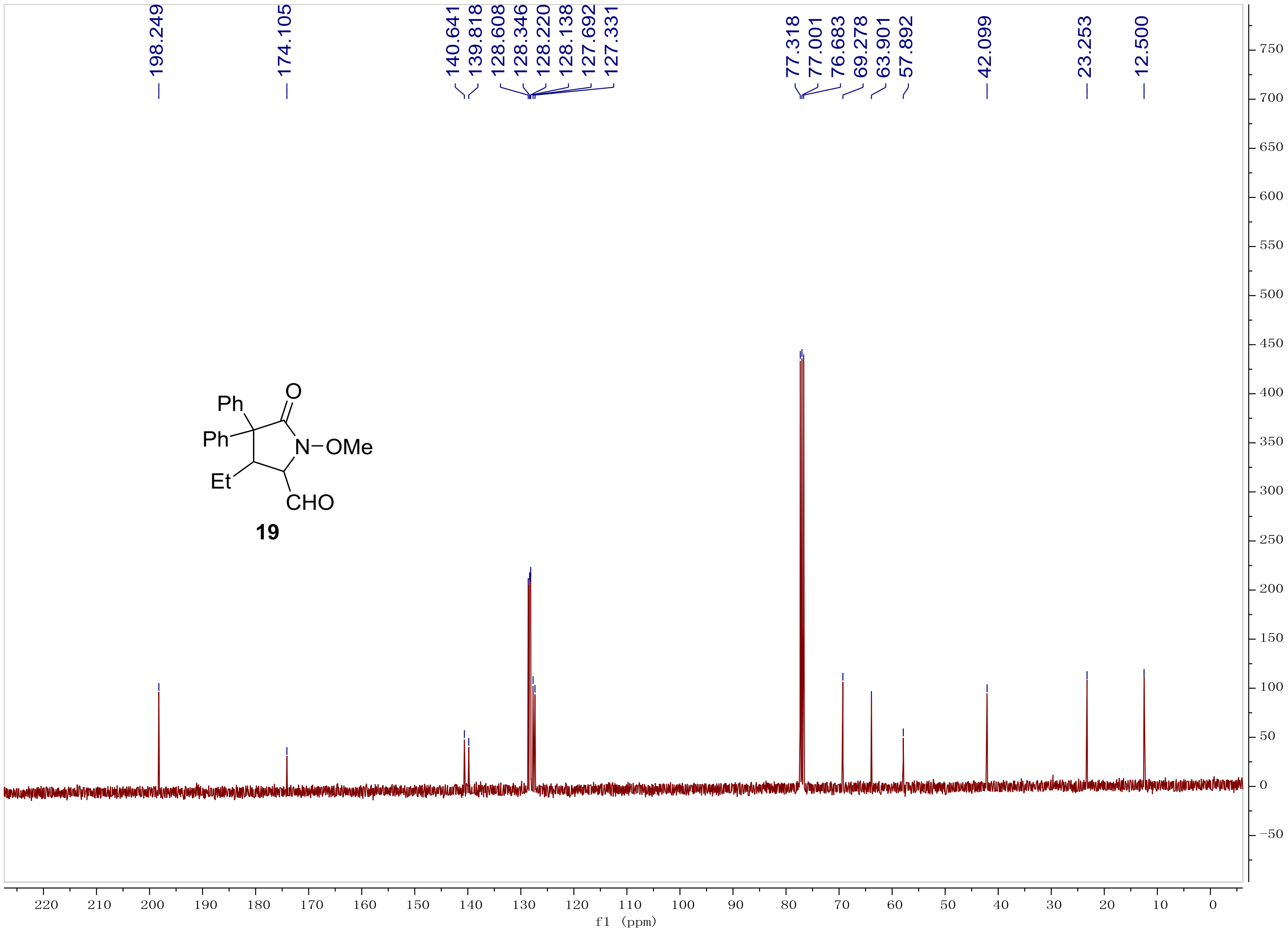
198.249
174.105
140.641
139.818
128.608
128.346
128.220
128.138
127.692
127.331
77.318
77.001
76.683
69.278
63.901
57.892
42.099
23.253
12.500
Ph
Ph
O
N−OMe
Et
CHO
19
750
700
650
600
550
500
450
400
350
300
250
200
150
100
50
0
−50
220 210 200 190 180 170 160 150 140 130 120 110 100 90 80 70 60 50 40 30 20 10 0
f1 (ppm)
